# Red flags for speech impairment: who should we refer to speech therapy?

**DOI:** 10.1136/archdischild-2025-329279

**Published:** 2026-02-25

**Authors:** Daisy Shepherd, Olivia van Reyk, Adam P Vogel, Simone Debono, Charlotte Boulton, Ashleigh Hill, Tully Coldrey, Francesca Coles, Angela Morgan

**Affiliations:** 1 Neurodevelopment, Murdoch Childrens Research Institute, Parkville, Victoria, Australia; 2 Department of Paediatrics, University of Melbourne, Melbourne, Victoria, Australia; 3 Speech and Language, Murdoch Childrens Research Institute, Parkville, Victoria, Australia; 4 Department of Audiology and Speech Pathology, The University of Melbourne - Parkville Campus, Melbourne, Victoria, Australia; 5 Redenlab, Melbourne, Victoria, Australia

**Keywords:** Child Development, Primary Health Care, Paediatrics, Child Health Services

## Abstract

**Background and objective:**

Speech disorders are a common presenting concern to paediatricians. Yet evidence to guide detection and speech sound therapy referral of cases at risk for persistent speech disorder is limited, with no normative English speech data published for over two decades. Here we describe speech development in a large, contemporaneous normative sample, to help clinicians identify errors of disordered speech from developmental errors (which are of lesser concern). We also examine differences in speech development over 20 years.

**Methods:**

Children aged 2 years 0 months–12 years 11 months were recruited from 24 sites (9 schools, 15 childcare/kindergarten settings) from New South Wales and Victoria in Australia. Speech sound acquisition and accuracy were documented, including whole word accuracy and type of sound error patterns categorised (developmental vs disordered).

**Results:**

1179 participants (53% male) were assessed. Speech performance was highly variable up to 6 years of age. By 7 years, 90% of participants could produce all sounds. There were negligible differences in word accuracy and error pattern performance between 8 and 12 years. Common disordered errors included transpositions (eg,‘efelant’ for elephant), vowel errors and backing (eg,‘glack’ for black). Compared with historical norms, sounds were acquired at a slower rate, and resolution of some common developmental errors was also slower.

**Conclusions:**

We found a high prevalence and variability of errors up to 6 years which explains why so many families seek support for speech development in the preschool years. Furthermore, our data suggest speech may be mastered more slowly nowadays compared with two decades ago. We provide an assessment tool with age-referenced normative cut-points and identify ‘red flag’ speech errors to guide data-driven referral of children at risk for persistent disorder to speech and language therapy.

**WHAT IS ALREADY KNOWN ON THIS TOPIC**

- Evidence to guide detection of speech disorder and targeted referral to speech sound therapy is limited. No large-scale normative English speech data have been published for over 20 years, despite indications that speech development has changed over this time.

**WHAT THIS STUDY ADDS**

- Speech errors are common in typical development until 6 years of age. We provide a tool to identify children at risk of speech disorder. Speech development was slower, but not more disordered, compared to historical data.

**HOW THIS STUDY MIGHT AFFECT RESEARCH, PRACTICE OR POLICY**

- We identify red flags for speech disorder to help guide appropriate referral to speech sound therapy.

## Introduction

 Speech and language impairments are some of the most common disorders of childhood, occurring in 8% of children.^1^ Speech impairments are perturbations of processing or production of speech sounds. By contrast, language impairments are deficits in expression or comprehension of vocabulary or grammar. These impairments are a common cause of parents presenting to primary care and paediatricians for guidance on communication development.^2^ Speech and language impairments can be debilitating in and of themselves, being associated with communication breakdown, psychosocial and mental health impacts.^3 4^ Here we focus on speech impairment, and specifically phonological disorder, which is the core predictor of poor language and literacy outcomes, with potential for deleterious impacts on education and longer-term employment.^5^ Yet we have limited data to guide practice on who will have a persistent speech sound disorder and require therapy, versus who will resolve error patterns without intervention.^1 6 7^

It is therefore challenging for healthcare professionals to reliably identify which children with speech production errors to refer to speech and language therapy (SALT). Specifically, it can be difficult to distinguish developmental speech errors from those indicative of disorder—and which should prompt referral to SALT. This forces a trial-and-error approach which results in potential under-servicing of those who need services most, and suboptimal use of critical resources.^8^,^10^

Developmental errors form predictable patterns known as ‘phonological processes’ which appear in typical speech development and resolve by specific ages.^11^ Given that developmental errors are common and expected in typically developing children, it is the *type* of error that is critical, not just the *number* of errors.^212^,^15^ The best validated speech diagnostic system in English is Dodd’s classification, which categorises errors based on type, into ‘developmental’ (age appropriate: used by more than 10% of children in an age group) or ‘disordered’ patterns (error pattern is not typical or expected: used by <10% of children at any age).^16^,^20^ The Dodd system was recently advocated for national use in the UK by the Royal College of Speech and Language Therapists.^20^ An example of a developmental error is the pattern of ‘cluster reduction’ which involves simplifying the number of consonants produced together, for example, ‘bue’ for ‘blue’ is seen in typical development up to 4 years of age.^16^ Whereas the substitution of posteriorly produced sounds for anterior sounds, a process known as ‘backing’, for example, saying ‘keddy’ for ‘teddy’, is not a pattern observed in typical development and is hence defined as a disordered error pattern.^18^ Disordered errors place the child at greater risk for persistent speech, language and literacy deficits.^2^ Hence, disordered errors are ‘red flags’ indicating a need for SALT referral and intervention. The other form of ‘red flag’ error is a delayed error form. That is, where a developmental pattern is persisting beyond the age of typical resolution, that is, the child is using an immature speech pattern relative to peers.^16^

Identifying children with speech disorders, not just developmental errors, requires individual speech sounds to be carefully transcribed and analysed to determine whether error forms are atypical from the norm and hence disordered. This deep characterisation is time-consuming and not possible for a busy healthcare professional, creating a barrier to evidence-based referral. Contemporary speech data are needed to better define ‘red flag’ error patterns, making them more recognisable to healthcare professionals, the critical conduits for SALT referral. Critically, there have been no community-based studies of English phonology for over 20 years. Further, previous studies encompassed relatively small samples of only a few hundred children or less and were not typically stratified for geographical or sociodemographic status.^21 22^ The lack of recent speech data is also concerning given suggestions that child phonological development is changing as the modern language environment has altered drastically with advancing technologies.^23 24^

Here we describe speech development in a normative sample of children aged 2 years–12 years 11 months. Our data will inform detection of speech disorder to guide referral and prioritisation of children needing therapy. We also determine whether there has been a change in speech development over the past 20 years.

## Methods

### Recruitment

Children aged 2–12 years were eligible. Children with medical, behavioural and attention disorders were flagged, but not excluded, to ensure data were representative of the general population. Children could have English as their primary or additional language. The exclusion criterion was being non-verbal. Children were recruited from 24 participating metropolitan and regional sites in greater Melbourne and regional New South Wales (15 childcare/kindergarten settings and 9 primary schools) and a range of socioeconomic areas, determined by Socio-Economic Indexes for Areas (SEIFA) using the Index of Relative Socio-economic Advantage and Disadvantage (IRSAD) (see online supplemental material page 4—*Recruitment and test administration* for further recruitment procedures).

### Test administration

Trained speech and language therapists assessed children individually using a picture naming task developed by the authors called the Assessment of Dysarthria Apraxia Articulation Phonology for planning Therapy (ADAAPT). Children were presented with each picture (eg, ‘helicopter’, ‘frog’) on an iPad and asked to say the name of the item in the picture. Responses were videorecorded and audiorecorded and phonetically transcribed using International Phonetic Alphabet (IPA) (see online supplemental material page 4—*Recruitment and test administration*, online supplemental figure S1 and tables S1–S5 for further test administration procedures).

### Statistical analyses and measures of speech performance

Analyses were conducted in R 4.3.1, with missing data quantified and reported. Key demographic and health characteristics are reported. To describe participation, the number of participants responding to all words was also reported (stratified by age group).

#### Sound (phonetic) acquisition

For each consonant, the number and percentage of participants (stratified by 12-month age bands) who produced the sound spontaneously (present within responses to the 55 word picture stimuli), produced it with prompting (stimulable, ie, sound was not produced within responses to the 55 picture stimuli but the child was able to subsequently produce the sound with prompting), did not produce it with prompting (not stimulable) or did not respond, was reported. Sound acquisition was also examined relative to history of SALT.

#### Sound (phonological) accuracy

##### Word-level accuracy

The median percentage of words correct (and IQR) was reported for each age group (12-month bands), with the distribution across age groups and gender visualised via boxplots. The difference in median percentage of words correct for (1) males compared with females, and (2) the first 6 months of age compared with the later 6 months (within the same age group) was estimated using linear quantile regression. Associated 95% CIs were estimated via the bootstrap (percentile) method. For each age band, a cut-point was defined corresponding to the 10% lowest performing participants. This cut-point represents the number of correctly produced words (ie, X words or less correct) that would classify the participant as falling into the lowest performing 10% for their age.

##### Sound level accuracy: developmental versus disordered error patterns

Speech was categorised into developmental and disordered (online supplemental table S6) error patterns. The number of opportunities children could make each of the developmental error patterns differed (eg, there were more opportunities to make a voicing error as this can occur across any devoiced sound, compared with a fronting velar error, which is limited to only one sound type (velars)). Number of errors for each error pattern was therefore reported relative to number of attempted opportunities. The percentage of developmental versus disordered error patterns was reported and visualised across age group and gender. For each error pattern and age band, a cut-point was defined based on the lowest performing 10% of participants at that age (ie, making X errors or more). The same analytic approach was used as described in the previous section.

### Comparison to previous normative data

We compared our data with previous publicly available normative data, by applying the same age-appropriate thresholds or expected age ranges for sound acquisition and use and resolution of developmental patterns from Dodd’s classification.^17^,^19^ A sound was considered acquired if more than 90% of the sample produced the sound with or without a prompt (within the same age band).^16^ An error pattern was deemed ‘age appropriate’ if more than 10% of children in an age group made the error pattern at least five times (or two times for weak syllable deletion as per previous norms), noting only a subset of seven error patterns was explored in the previous data.^17^ In addition, a narrower age range was described within the Dodd *et al*^17^ paper and therefore our comparison was restricted to a window of children of the same age in our sample (age 3–6 years).

## Results

### Study participants

A total of 1186 participants were recruited. Seven participants (aged 2–3 years) responded to none or only one stimulus and thus were removed from subsequent analysis (n=1179). Age at assessment was evenly distributed, with fewer children recruited at age 10 years and older (table 1). Participants were from predominantly English-speaking households (93.2%) with a range of socioeconomic backgrounds (IRSAD quintiles 1–5, where 1=most advantaged and 5=most disadvantaged; ranging 9.8%–26.1% across quintiles). The sample was representative of the general population (online supplemental table S7). Approximately 18% (210/1179) of participants had previously seen a speech and language therapist and 20% of those (43/210) had received a formal speech or language diagnosis (table 1). Around 8% (86/1179) of participants had received another cooccurring health or developmental diagnosis, most commonly autism spectrum disorder (n=19) or hearing loss (n=12). The majority of the sample attempted all test items (online supplemental tables S8 and S9).

**Table 1:** Demographic characteristics and health history of participants recruited within the study (who responded to at least one word within the assessment)

	Missing n (%)	Sample (n=1179) n (%)
Demographic characteristics		
Sex	0 (0)	
Female		552 (46.8)
Male		625 (53.0)
Another term		2 (0.2)
Age at time of assessment (years; months)	0 (0)	
2;0–2;11		99 (8.4)
3;0–3;11		107 (9.1)
4;0–4;11		153 (13.0)
5;0–5;11		137 (12.0)
6;0–6;11		127 (11.0)
7;0–7;11		138 (12.0)
8;0–8;11		122 (10.0)
9;0–9;11		105 (8.9)
10;0–10;11		85 (7.2)
11;0–11;11		69 (5.8)
12;0–12;11		37 (3.1)
Aboriginal or Torres Strait Island origin	48 (4.1)	59 (5.2)
Remoteness area	0 (0)	
Major cities		846 (71.8)
Inner regional		333 (28.2)
IRSAD* quintile	1 (0.08)	
1=most disadvantaged		336 (28.5)
2		308 (26.1)
3		116 (9.8)
4		115 (9.8)
5=most advantaged		303 (25.7)
School ICSEA†	47 (4.0)	
Lower middle (900–1000)		429 (37.9)
Upper middle (1000–1100)		1 (0.1)
Top (1100–1200)		348 (30.7)
Not applicable (child not yet attending school)		354 (31.3)
Main language spoken at home	41 (3.5)	
English		1060 (93.2)
Other		78 (6.8)
Speaks language other than English	177 (15.0)	204 (20.4)
Understands language other than English	178 (15.1)	241 (24.1)
Health history		
Previously seen speech and language therapist	221 (18.7)	210 (21.9)
Received diagnosis by speech and language therapist	12	43
Type of diagnosis		
Articulation disorder		13
Phonological disorder		12
Stuttering		8
Apraxia of speech		0
Dysarthria		0
Expressive language disorder		13
Receptive language disorder		4
Semantic disorder		2
Grammatical disorder		3
Reading disorder		3
Spelling disorder		2
Resonance disorder		1
Other diagnosis (not specified)		2
Received health or developmental diagnosis	43 (3.6)	86 (7.6)
Type of diagnosis		
Autism spectrum disorder		19
Hearing loss		12
Epilepsy		1
Intellectual disability		6
Chromosomal/genetic condition		1
Other condition		27

* Index of Relative Socioeconomic Advantage and Disadvantage (IRSAD).

† Index of Community Socioeducational Advantage (ICSEA)—reflects the socioeducational background of the students.

#### Sound (phonetic) acquisition

Sounds that are traditionally recognised as earlier-developing (eg, plosives: p, b, t, d, k, g and nasals: n, m, ŋ) were present in spontaneous productions for almost all participants from age 2 years (online supplemental figure S2 and table S10). Fricatives (f, v, s, z, ʃ, ʒ, θ, ð) and affricates (ʧ, ʤ) were less commonly acquired by 2 or 3 year olds, but were generally acquired (ie, present in spontaneous productions or stimulable) by 4 years of age or older. After the age of 7, 90% of participants were able to produce all sounds.

#### Sound (phonological) accuracy

##### Word level

Word accuracy improved with age (online supplemental table S11 and figure S3). For participants aged 5 years or younger, females outperformed males, with the median words correct ranging from 7.3 to 15.8 percentage points higher (online supplemental figure S4A), although performance was more varied for younger children. For participants aged 6 years or older, performance was similar between males and females.

For participants aged 5 years and younger, individuals in the later age band (months 6–11) produced more words correctly than younger participants (months 1–5) within the same 12-month age band (median difference of 2.3–12.7 percentage points; online supplemental figure S4). However, the difference in performance was highly variable within these younger age groups (indicated by wide CIs). From age 7 years and older, the 6-month difference was negligible.

Cut-points for number of words correct were determined (see online supplemental table S12) for exact cut-points stratified by age and incorporated into an abbreviated scoring form (online supplemental table S13) for physicians or other health or educational professionals to use alongside (online supplemental figure S1).

##### Sound level accuracy: developmental patterns

**Figure 1:**
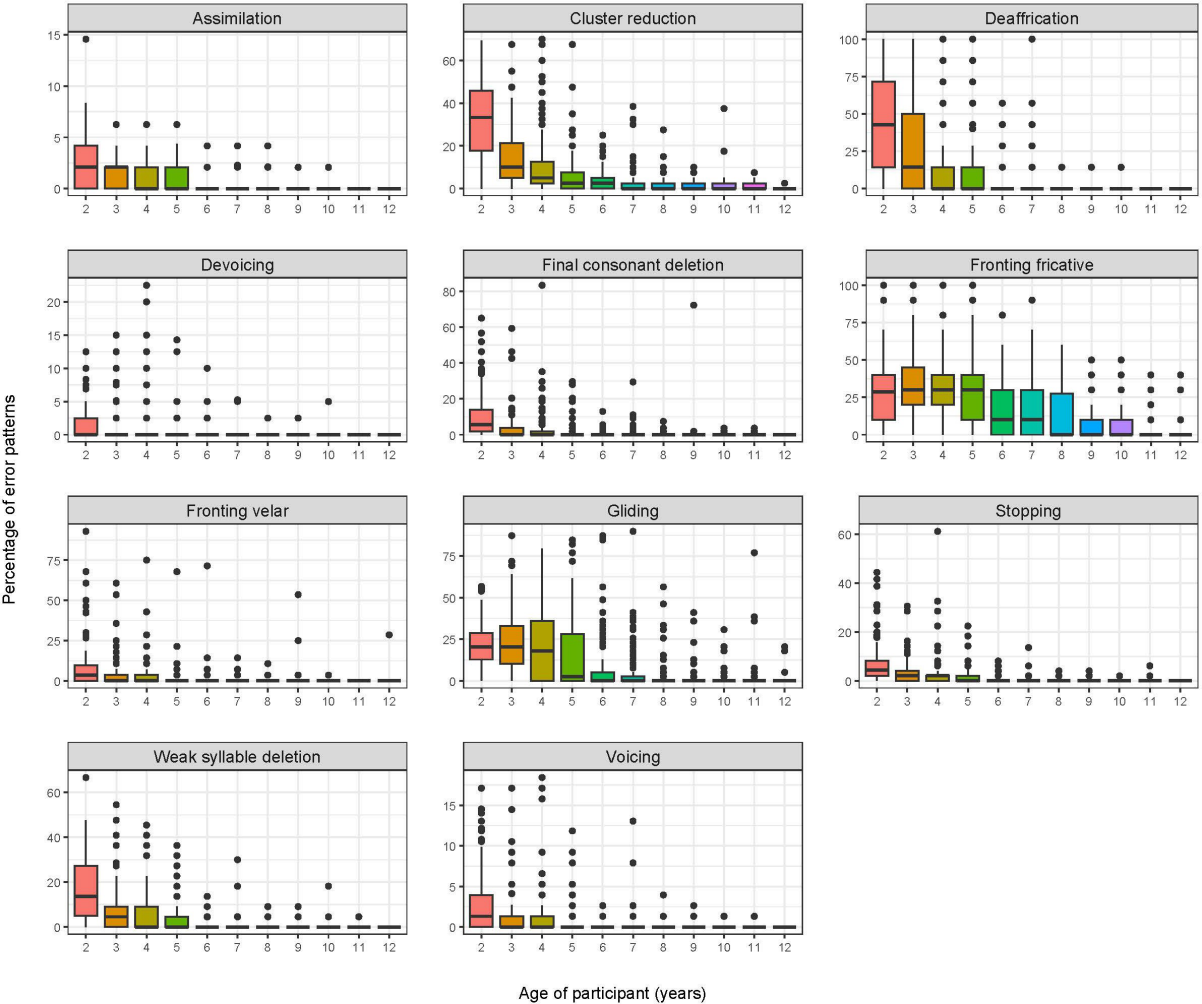
Percentage of developmental error patterns across age of participants stratified by type of error. The percentage of error patterns quantifies the number of errors made relative to the number of opportunities available and attempted.

The proportion of words attempted (and therefore error pattern opportunities) increased with age (online supplemental table S14). The percentage of error patterns decreased with age across all error types (figure 1). In general, the percentage of devoicing, final consonant deletion, fronting velars and stopping errors was low across all ages (figure 1). The overall percentage of errors was low (median percentage errors <10%), except for cluster reduction, deaffrication, fronting fricatives and gliding errors (figure 1; online supplemental tables S15 and S16). In descending order, many 2-year-old children made at least one developmental error (online supplemental table S15). For children aged 12 years (n=37), very few made any errors, and if so, at a low frequency (online supplemental table S15 and figures S5–S16).

When comparing male and female participants, the frequency of error patterns was similar for most patterns across all ages. Any differences occurred in younger ages (6 years or younger), with males making more errors than females (cluster reduction, fronting fricatives, weak syllable deletion, gliding, deaffrication; online supplemental figures S5–S16). Similar observations were made when comparing 6-month age bands, with younger participants (months 1–5) making more errors than older participants (months 6–11). However, after the age of 6 years, this difference was negligible (online supplemental figures S5–S16). As for sound accuracy at the word level, we have now also determined the cut-points for developmental errors (online supplemental table S17).

##### Sound level accuracy: disordered patterns

Most younger participants made at least one disordered error (94.9%–51.1% for ages 2–5 years, respectively; online supplemental table S18), and error frequency decreased with age (online supplemental table S18). The frequency of disordered errors was similar between females and males across all ages (online supplemental figures S17–S29). Two-year-old to 4-year-old children showed the greatest number of disordered errors, largely resolved by 6 years of age. The most common disordered error types were vowel errors (eg, /boːd/ (‘bord’) for /bɜːd/ (bird), /ʃɪp/ ‘ship’ for /ʃiːp/ (sheep)), backing (eg, /glæk/ (‘glack’) for /blæk/ (black)), transpositions (eg, /efələnt/ (‘efelant’) for /eləfənt/ (elephant)) and atypical sound substitutions (eg,/lʉː/ (‘loo’) for /zʉː/ (zoo), /hiːp/ ‘heep’ for /ʃiːp/ (sheep)). As for sound accuracy at the word level, we have now also determined the cut-points for disordered errors (online supplemental table S19).

### Comparison to previous normative data

Phonetic acquisition for plosive and nasal sounds was the same as in previous data (all present by 3 years; online supplemental tables S20 and S1). Mastery of fricative sounds /s/ and /z/ occurred later than previous norms when using the 90% criteria (online supplemental table S21); however, they were present in more than 80% of children in most age bands (online supplemental table S20). Children who had not acquired /s/ and /z/ from 6 years old were twice as likely to have previously seen a speech and language therapist compared with the total sample (online supplemental table S22). The frequency of developmental errors of fronting velars, stopping and voicing was consistent between our sample and past norms (table 2). By contrast, gliding, deaffrication, cluster reduction and weak syllable deletion errors persisted into older ages in our subgroup than previously noted in the historical dataset.^17^ There was no evidence of a higher number of disordered error patterns in the current generation.

**Table 2:** Percentage of cohort making phonological error patterns (assessed with ADAAPT) compared with outcomes reported by Dodd *et al*^17^ using the Diagnostic Evaluation of Articulation and Phonology

Age group (years;months)	Gliding		Deaffrication		Cluster reduction		Fronting velar		Weak syllable deletion		Stopping		Voicing	
	Current analysis	Dodd *et al* (2003)	Current analysis	Dodd *et al* (2003)	Current analysis	Dodd *et al* (2003)	Current analysis	Dodd *et al* (2003)	Current analysis	Dodd *et al* (2003)	Current analysis	Dodd *et al* (2003)	Current analysis	Dodd *et al* (2003)
3;0–3;5	**71.2**	*>10%*	**15.4**	*>10%*	**48.1**	*>10%*	9.6	*>10%*	**40.4**	*>10%*	3.8	*>10%*	1.9	
3;6–3;11	**61.8**	*>10%*	**18.2**	*>10%*	**43.6**	*>10%*	**10.9**	*>10%*	**36.4**	*>10%*	5.5		3.6	
4;0–4;5	**62.3**	*>10%*	**10.4**	*>10%*	**27.3**	>10%*	7.8		**32.5**		3.9		3.9	
4;6–4;11	**50.0**	*>10%*	5.3	*>10%*	**27.6**	>10%*	0.0		**23.7**		5.3		1.3	
5;0–5;5	**41.0**	*>10%*	8.2		**19.7**		1.6		**13.1**		0.0		3.3	
5;6–5;11	**30.3**	*>10%*	2.6		6.6		1.3		**14.5**		1.3		0.0	
6;0–6;5	**21.2**		0.0		**12.1**		3.0		**15.2**		0.0		0.0	
6;6–6;11	**13.8**		0.0		3.2		0.0		2.1		0.0		0.0	

Values in italics correspond to findings from Dodd *et al*, when >10% of the normative population were making at least five error patterns (or two for weak syllable deletion). Values in bold correspond to findings from this study when >10% of the sample made at least five error patterns (or two for weak syllable deletion). The contemporary ADAAPT cohort took longer to resolve some error patterns across age ranges.

* These figures pertain to reduction of triclusters (eg, /spl/) only, that is, reduction of biclusters (eg, /sp/) had resolved by 3;11, while reduction of triclusters resolved by 4;11 in the cohort in Dodd *et al*.^17^

ADAAPT, Assessment of Dysarthria Apraxia Articulation Phonology for planning Therapy.

## Discussion

For paediatricians and other professionals to guide clinical decision-making for referral to SALT, it is critical to understand the use and resolution of both speech and language domains. Here we focus on specific speech errors only. A data-driven approach to referral will lead to fewer children being incorrectly assigned to wait lists and would ensure fewer resources are wasted treating speech error patterns that are likely to resolve without resolution.

Here we provide the largest normative speech dataset for two decades, on a representative sample of English-speaking children aged 2–12 years. Findings confirm developmental speech errors are common and highly variable in typical speech development up to 6 years of age. Once children turn 7, speech sound acquisition plateaus and speech errors decrease and largely stabilise. The frequency of typical errors up to 6 years explains why so many families seek advice from paediatricians around speech development in the preschool years.

Our data also highlight why it is challenging for paediatricians to identify who to refer to SALT versus who to monitor. Almost all preschool children make speech errors. Speech and language therapists emphasise that speech must be classified based on *type* of error*,* that is, disordered versus typical speech errors, not just number of errors, based on our knowledge of longer-term speech, language and literacy outcomes for children with atypical error forms.^25^ Speech error characterisation and detection of children with disordered errors for referral ensures fewer ‘at risk’ children miss out on the treatment they require. Yet it is difficult for paediatricians or other health or educational professionals who do not have training in the IPA or speech analysis to provide a granular analysis of error *type* required to make referral decisions in one busy consult. Here we have also simplified speech assessment for non-SALT colleagues by providing age-based normative data. By administering our assessment (online supplemental figure S1) and consulting online supplemental table S13 to determine whether a child was in the bottom performing 10% for their age group, a paediatrician or other health or educational professional can make a data-driven decision regarding appropriateness of SALT referral. Into the future, it would be useful to have a more succinct tool with fewer stimuli items. We have also provided further detail in the way of red flag pointers for speech sound error types (table 3) for busy clinicians without time to administer a test. We also emphasise our focus on speech sound disorders and highlight that, of course, many children have cooccurring language support needs which may also influence decision-making for referral to SALT.^26^

**Table 3:** Speech error types used in the normative sample, with red flag indicators for speech disorder

Error patterns key: 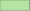 Age-appropriate: used by more than 10% of children in age band. 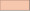 Delayed*; used by less than 10% of children in age band, but more than 10% of children of younger age bands. 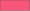 Disordered*; seen in less than 10% of children of any age.								
Developmental error pattern	Example(s) of error pattern	Age band (years;months)						
		2;0–2;5	2;6–2;11	3;0–3;5	3;6–3;11	4;0–4;11	5;0–5;11	6:0-6:11
Assimilation: sound influences production of another sound in word	‘lellow’ for yellow /leləʉː/ for /jeləʉː/	**Age-appropriate**	**Age-appropriate**	*Delayed*	*Delayed*	*Delayed*	*Delayed*	*Delayed*
Final consonant deletion: deletion of final consonant of word or syllable	‘pi’ for pig /pɪ/ for /pɪg/	**Age-appropriate**	**Age-appropriate**	*Delayed*	*Delayed*	*Delayed*	*Delayed*	*Delayed*
Stopping: replacement of fricatives with stops	‘doo’ for zoo /dʉː/ for /zʉː/	**Age-appropriate**	**Age-appropriate**	*Delayed*	*Delayed*	*Delayed*	*Delayed*	*Delayed*
Voicing: replacement of devoiced sound with voiced sound	‘bin’ for pin /bɪn/ for /pɪn/	**Age-appropriate**	**Age-appropriate**	*Delayed*	*Delayed*	*Delayed*	*Delayed*	*Delayed*
Fronting velars: place of articulation of velar moved to a more anterior position	‘tate’ for cake /tæɪt/ for /kæɪk/	**Age-appropriate**	**Age-appropriate**	**Age-appropriate**	**Age-appropriate**	*Delayed*	*Delayed*	*Delayed*
Deaffrication: replacement of affricate sound with a stop, fricative or stop+substituted fricative	‘wits’ for witch /wɪts/ for /wɪʧ/	**Age-appropriate**	**Age-appropriate**	**Age-appropriate**	**Age-appropriate**	**Age-appropriate**	*Delayed*	*Delayed*
Cluster reduction: deletion of consonant from a consonant cluster	‘boom’ for broom /bʉːm/ for /bɹʉːm/	**Age-appropriate**	**Age-appropriate**	**Age-appropriate**	**Age-appropriate**	**Age-appropriate**	**Age-appropriate**	*Delayed*
Fronting fricatives: place of articulation of fricative moved to a more anterior position	‘teef’ for teeth /tiːf/ for /tiːθ/	**Age-appropriate**	**Age-appropriate**	**Age-appropriate**	**Age-appropriate**	**Age-appropriate**	**Age-appropriate**	*Delayed*
Weak syllable deletion: deletion of unstressed syllable	‘di-saur’ for dinosaur /dɑesoː/ for /dɑenəsoː/	**Age-appropriate**	**Age-appropriate**	**Age-appropriate**	**Age-appropriate**	**Age-appropriate**	**Age-appropriate**	*Delayed*
Gliding: replacement of liquids with glides	‘wainbow’ for rainbow /wæɪnbəʉ/ for /ɹæɪnbəʉ/	**Age-appropriate**	**Age-appropriate**	**Age-appropriate**	**Age-appropriate**	**Age-appropriate**	**Age-appropriate**	**Age-appropriate†**

Disordered error pattern	Example(s)	2;0–2;5	2;6–2;11	3;0–3;5	3;6–3;11	4;0–4;11	5;0–5;11	6:0-6:11
Addition error: addition of sound/syllable to word	‘stards’ for stars /stɐːdz/ for /stɐːz/	***Disordered***	***Disordered***	***Disordered***	***Disordered***	***Disordered***	***Disordered***	***Disordered***
Frication/affrication: Replacement of sound/cluster with affricate or fricative	‘choes’ for shoes /ʧʉːz/ for /ʃʉːz/	***Disordered***	***Disordered***	***Disordered***	***Disordered***	***Disordered***	***Disordered***	***Disordered***
Atypical sound substitution: sound substitutions not otherwise categorised in any developmental error patterns	‘nitch’ for witch /nɪʧ/ for /wɪʧ/	***Disordered***	***Disordered***	***Disordered***	***Disordered***	***Disordered***	***Disordered***	***Disordered***
Backing: place of articulation moved to more posterior position	‘keggy’ for teddy/kegiː/ for /tediː/	***Disordered***	***Disordered***	***Disordered***	***Disordered***	***Disordered***	***Disordered***	***Disordered***
Transposition: swapping/inverting position of sound(s) in word	‘karangoo’ for kangaroo/kæɹæŋgʉː/ for /kæŋgəɹʉː/	***Disordered***	***Disordered***	***Disordered***	***Disordered***	***Disordered***	***Disordered***	***Disordered***
Initial consonant deletion: omission of initial sound/cluster of word/syllable	‘ar’ for star/ɐː/ for /stɐː/	***Disordered***	***Disordered***	***Disordered***	***Disordered***	***Disordered***	***Disordered***	***Disordered***
Vowel error: replacement of vowel with another vowel	‘bord’ for bird /boːd/ for /bɜːd/	***Disordered‡***	***Disordered‡***	***Disordered***	***Disordered***	***Disordered***	***Disordered***	***Disordered***

Speech sounds are denoted using the International Phonetic Alphabet (IPA) symbols as per convention in the field of speech and language therapy, with examples of sounds in real words provided in written English for broader audience.

* Speech therapy referral indicated for delayed and disordered error patterns alongside caregiver/healthcare professional concerns regarding ability for speech to be understood by others. A tool to measure speech intelligibility is the Intelligibility in Context Scale (https://www.csu.edu.au/research/multilingual-speech/speech-assessments/ics).

† Less than 10% of children use gliding beyond 7 years, 11 months.

‡ Vowel errors were seen in 20% of 2-year-old children but less than 10% of children from 3 years old.

A secondary aim was to compare present day speech performance to historical normative speech data. Preliminary reports suggest child speech performance has changed relative to past generations.^23^ When compared with previous norms, some aspects of speech development in our cohort were commensurate with past norms, while others showed a slower trajectory. Children acquired speech sounds in a similar *sequence* to previous data; whereby plosives and nasals were acquired first, followed by less commonly used and more motorically complex sounds of fricatives and affricates. Although, as regards the specific age of mastery, affricates (ʧ, ʤ) were mastered 6 months slower than past norms, and fricatives /s/ and /z/ were also 2–3 years later to consolidate when using a 90% criterion, but were mastered by at least 80% of children in most age bands. Our sample was also using developmental error patterns of gliding, cluster reduction and weak syllable deletion for longer time periods relative to previous norms.^17^ Whereas the resolution of other developmental patterns of deaffrication, fronting velars, stopping and voicing errors was consistent with previous norms.^17^ We did not find evidence of more disordered performance in our sample compared with past norms. We acknowledge that differences in assessment tools used between the historical norms and our study may limit direct comparison. Neither our study nor the historical normative study accounted for the influence of past speech therapy on outcomes. Contemporary differences in speech outcomes could have been influenced by differences in access and uptake of speech services now compared with those of the past.

Our analysis is limited by reliance on cross-sectional data, although past studies have confirmed the longer-term risk of adverse speech outcome in children who present with atypical errors at a younger age.^2 26^ To our knowledge, there are no longitudinal datasets to inform speech outcomes for a normative sample of children over time. As a result, existing clinical speech tests, including those recently advocated for in the Royal College of Speech and Language Therapists guidelines 2024 for child speech disorders, are based on cross-sectional data only.^16 20^ Further, the emphasis on single word testing rather than conversation or connected discourse in our analysis here is commensurate to existing clinical speech tests. Hence, there is a need to develop assessment tools and clinical referral/prioritisation algorithms based on longitudinal data, as well as incorporating outcome measures in conversation or connected discourse rather than single words alone.

We confirm that speech errors are common and variable in typical speech development up to 6 years. We highlight that the distinction between ‘developmental’ and ‘disordered’ speech errors remains clinically relevant.^16^,^19^ Our data support paediatricians and other health and educational professionals to improve detection of disordered speech errors and achieve more targeted referral for speech sound therapy.

## Supplementary material

10.1136/archdischild-2025-329279 online supplemental file 1

## Data Availability

Data are available upon reasonable request. All data relevant to the study are included in the article or uploaded as supplementary information.
