# Supplementary material for: Red flags for speech impairment: who should we refer to speech therapy?
Source: Arch Dis Child. 2026 Feb 25;111(7):e329279. doi: 10.1136/archdischild-2025-329279 (PMC13311972; doi:10.1136/archdischild-2025-329279)
Supplement: online supplemental file 1 [file archdischild-111-7-s001.pdf]

Red flags for speech impairment: who should we refer to speech therapy?

SUPPLEMENTARY MATERIAL

Daisy A Shepherd BSc MSc PhD[1,2], Olivia van Reyk BSc MSpPath[3], Adam P. Vogel PhD[4,5], Simone Debono BA MSpPath[3], Charlotte Boulton BA MSpPath[3], Ashleigh Hill BA MSpPath[3], Tully Coldrey BA MSpPath[3], Francesca Coles BA MSpPath[3], Angela T. Morgan BSpPath Aud Hons PhD[3,4]

[1]Department of Paediatrics, The University of Melbourne, Melbourne, Australia

[2]Clinical Epidemiology and Biostatistics Unit, Murdoch Children's Research Institute, Melbourne, Australia

[3]Speech and Language, Murdoch Children's Research Institute, Melbourne, Australia

[4]Department of Audiology and Speech Pathology, The University of Melbourne, Melbourne, Australia

[5] Redenlab Ltd. Melbourne, Australia

CONTENTS

SPEECH ASSESSMENT 4
  Recruitment and test administration 4
  Picture stimuli 4
    Figure S1. Picture stimuli (55 in total) used in the Assessment and Diagnosis of Articulation and Phonology with Treatment (ADAAPT). 4
  Consonant inventory 60
    Table S1. Frequency and distribution of consonants in the 55 picture stimuli. 60
  Consonant cluster inventory 61
    Table S2. Frequency and distribution of two- and three-consonant clusters in the 55 picture stimuli. 61
  Vowel inventory 62
    Table S3. Frequency and distribution of vowels of Australian English (transcribed in Harrington/Cox/Evans (HCE) system) in the 55 picture stimuli. 62
  Multisyllabic words and syllable shapes 64

Table S4. Frequency of monosyllabic and multisyllabic words in the 55 picture stimuli. 64
Table S5. Frequency and distribution of syllable shapes in the 55 picture stimuli. 64
Speech sound error patterns 66
Table *S6*. (a) Developmental and (b) disordered sound (phonological) error patterns 66
STATISTICAL ANALYSES: ADDITIONAL INFORMATION 68
Comparison between males and females 68
Comparison between 6-month age windows 68
ADDITIONAL RESULTS 69
Study Participants 69
Table S7. Demographic characteristics of participants recruited within the study (who responded to at least one word within the assessment) compared to Australian national census data. 69
Participation 69
Table S8. Number of participants who attempted all words during assessment stratified by age group. 70
Table S9. Top 25 words commonly not responded to across all age levels. [a]Note that sky is the first word on the assessment. 70
Figure S2. Percentage of participants responding to each sound stratified by age group. 71
Sound (phonetic) acquisition 72
Table S10. Proportion of participants (stratified by age group) who had a specific sound present, stimulable, not stimulable or did not respond (denoted as “pres”, “stim”, “not stim”, “no resp”, respectively). 72
Sound (phonological) accuracy 75
Word level 75
Sound level accuracy: developmental patterns 80
Sound level accuracy: disordered patterns 103
Comparison to previous normative data 125
Table S20. Percentage of participants with the sound present (with or without stimulus) broken down by 6-month age bands. The shaded cells correspond to when the percentage is equal to or greater than 90% (the threshold from Dodd et al. (2003) paper) 125

Table S21. Phonetic acquisition from our study compared to the previous study by Dodd et al. (2003). Under this threshold, a sound was considered present if 90% or more of that age band could produce the sound with or without stimulus. Plosive speech sounds are produced by using the lips, teeth, or palate to stop airflow before a sudden release of air. Nasal sounds are produced when air escapes via the nose rather than mouth. Fricative sounds are produced by releasing air through two articulators close together (e.g. upper teeth on lower lip to produce /f/). Affricates combine a plosive feature to start before releasing a fricative feature. Approximant sounds are produced by two articulators coming together but not as narrowly as fricatives, without turbulent airflow................. 126

Table S22. Proportion of participants with sounds not present (not stimulable or no response) stratified by age band (6 months) alongside the speech pathology history... 128

SPEECH ASSESSMENT

Recruitment and test administration

All children were invited to participate in an opt-in consent process; as required by respective Education ethics committees. Parents/caregivers provided written consent and completed a brief survey on age, sex, Aboriginal and/or Torres Strait Islander origin, primary language and additional languages, speech and language therapy services accessed, health/developmental diagnoses (i.e. autism spectrum disorder, hearing loss, epilepsy, intellectual disability, chromosomal/genetic condition or any other conditions.

Speech and language therapists tested children at each site using a picture naming task called the Assessment of Dysarthria Apraxia Articulation Phonology for planning Therapy (ADAAPT). Fifty-five single word picture stimuli elicited all English consonants in initial and final syllable positions (see Figure S1). Stimuli ranged from one to four syllables of carefully selected syllable shapes representative of English. Sounds were also elicited within clusters (e.g. ‘st’ ‘pl’). (Table S1-S5).

Picture stimuli

Figure S1. Picture stimuli (55 in total) used in the Assessment and Diagnosis of Articulation and Phonology with Treatment (ADAAPT).

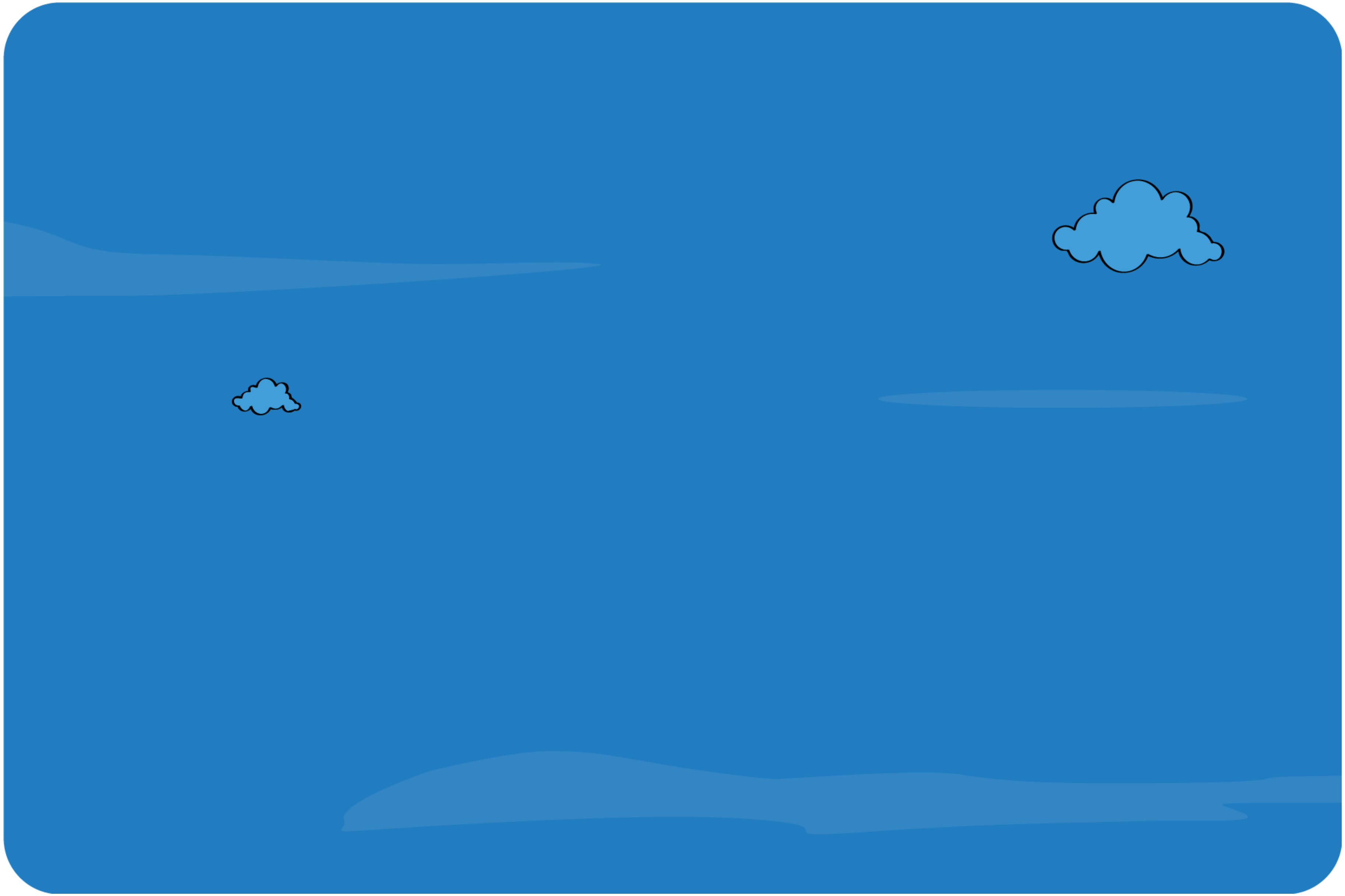

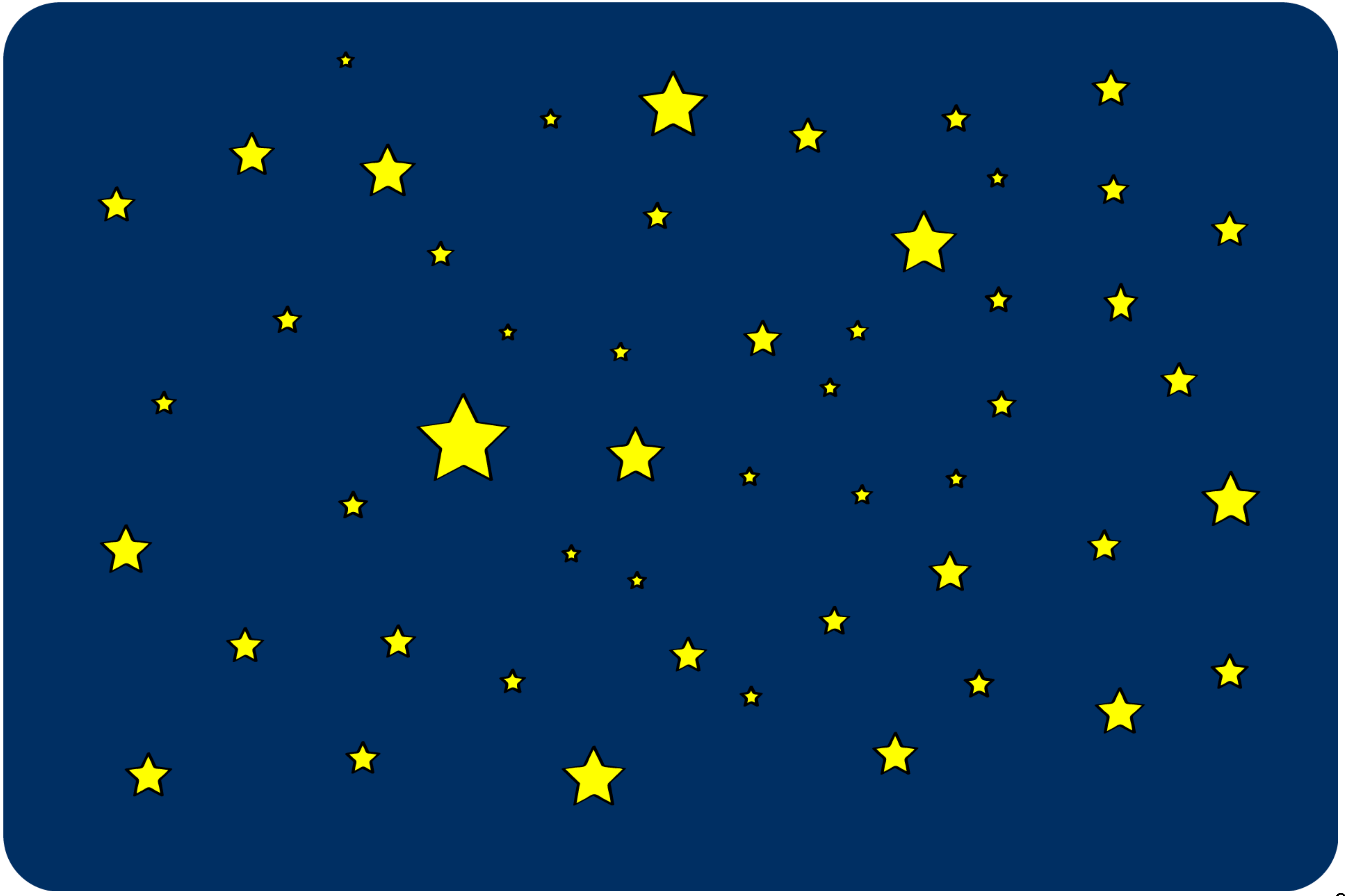

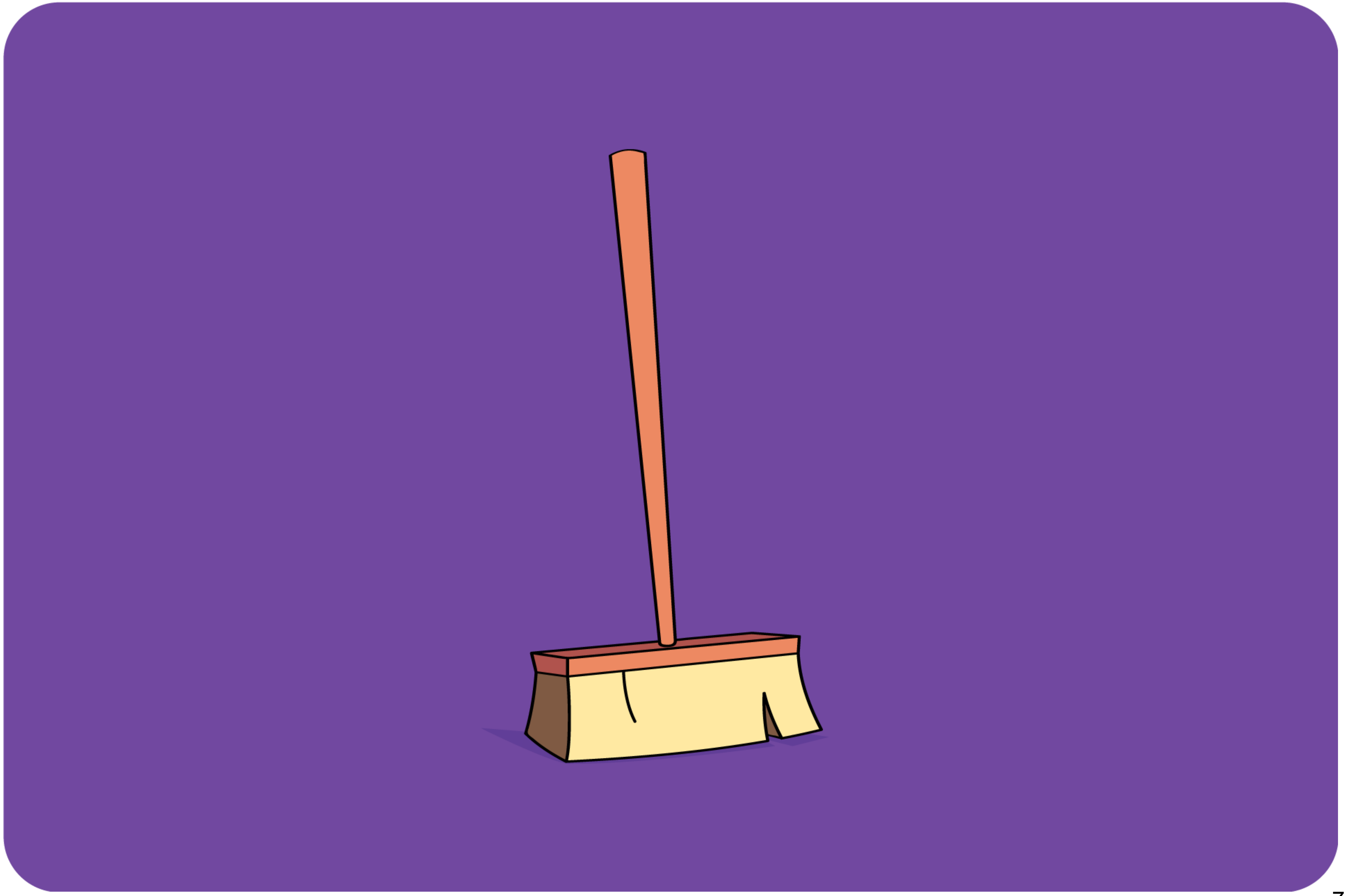

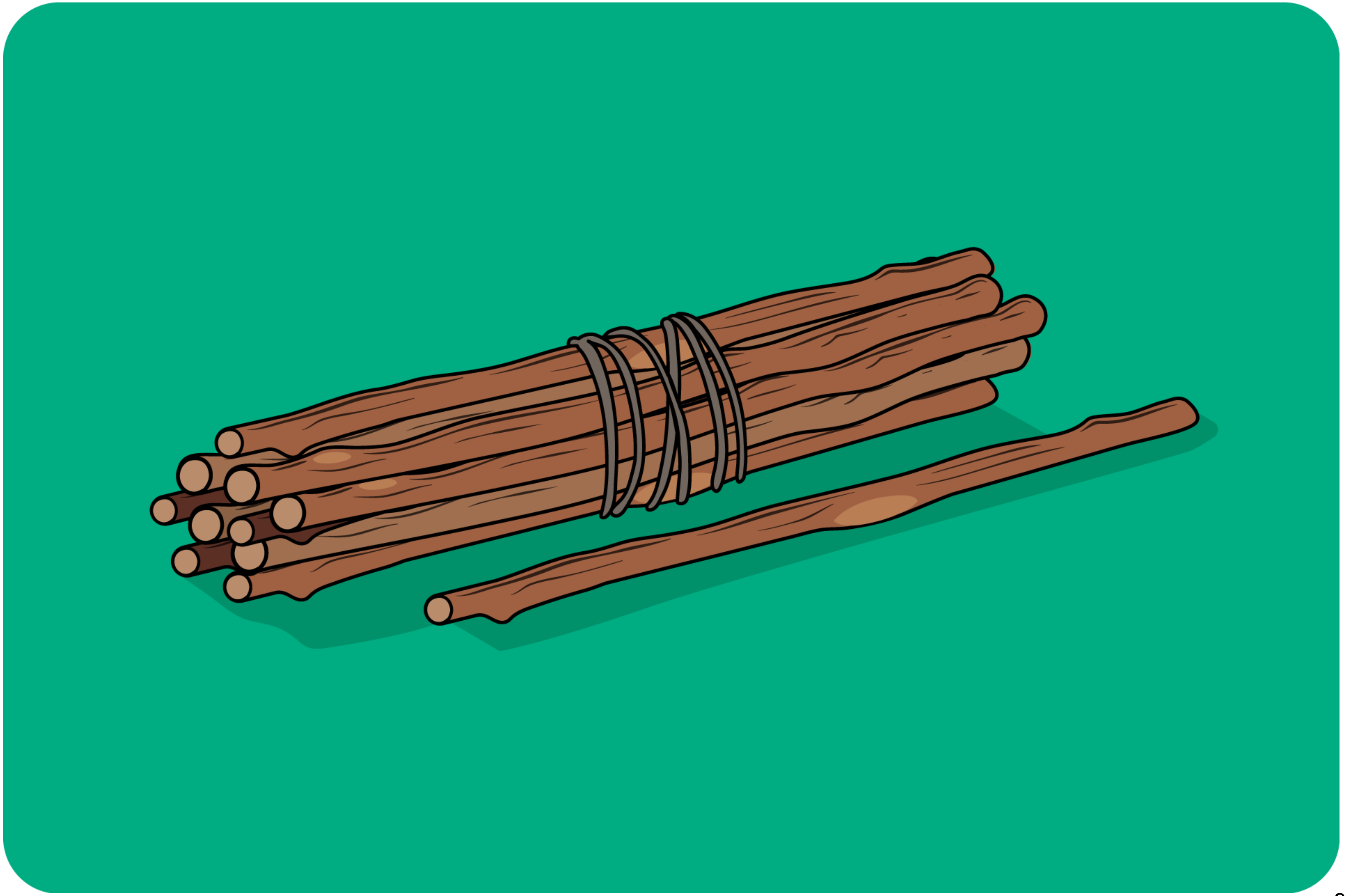

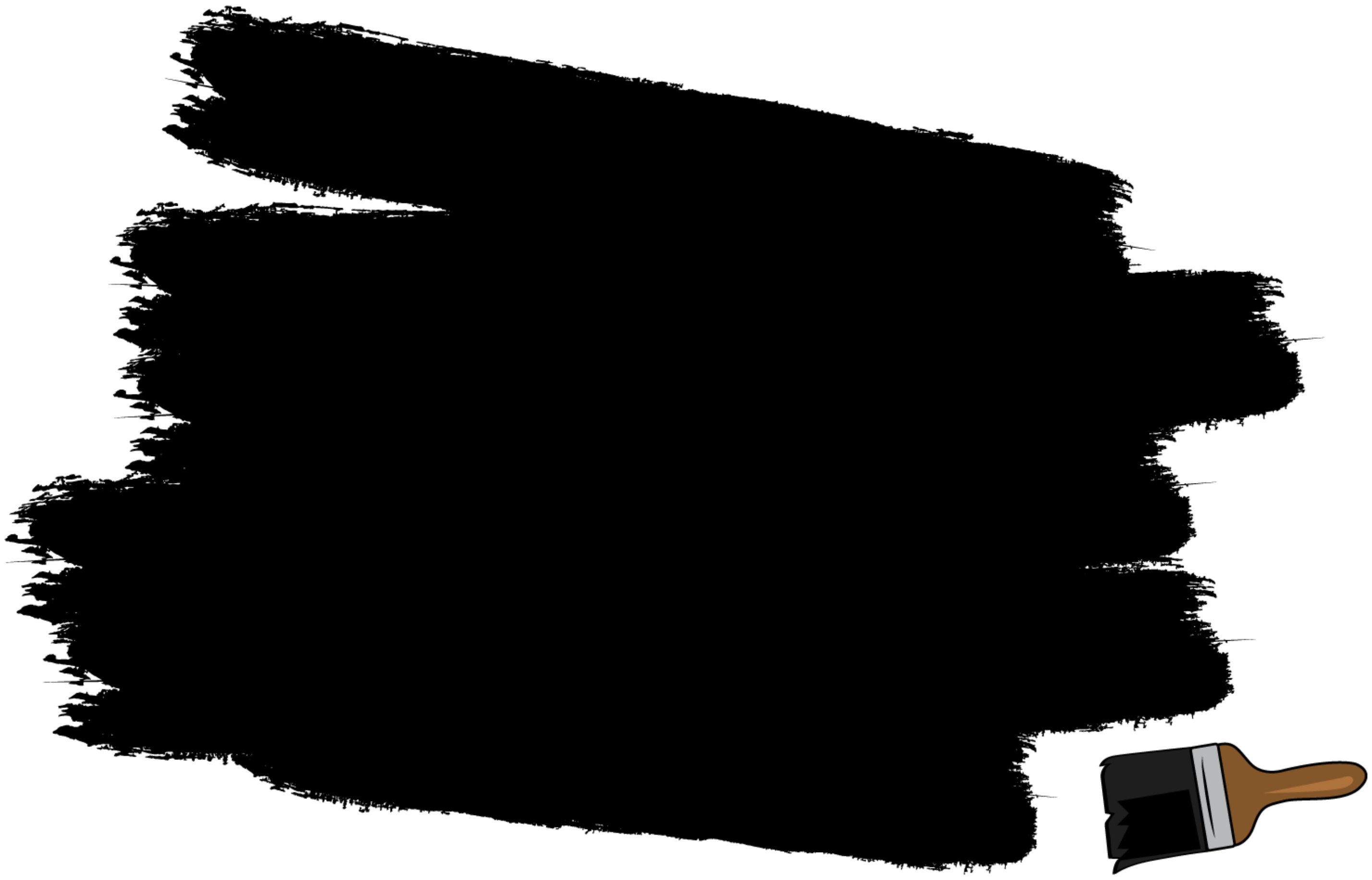

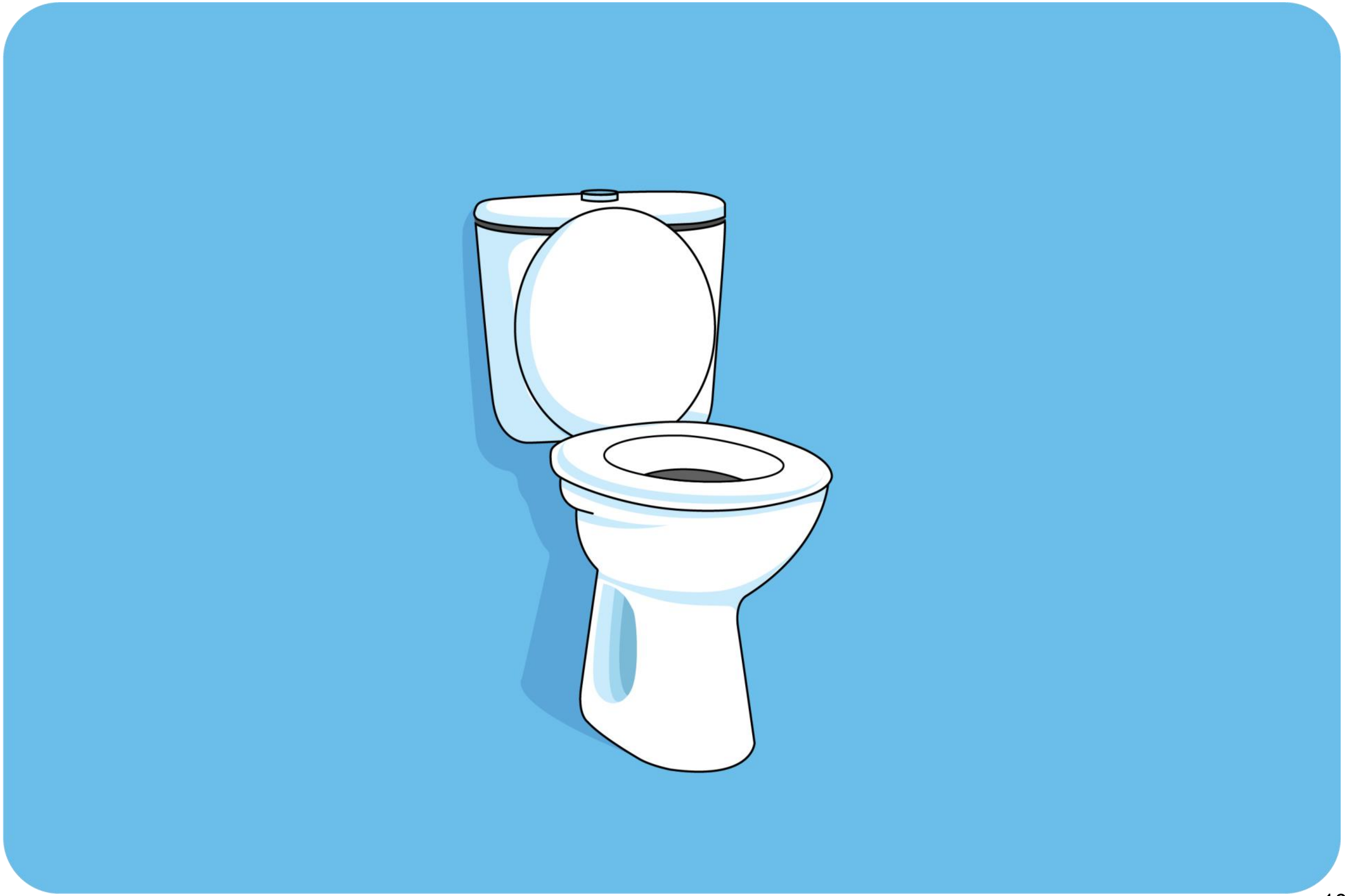

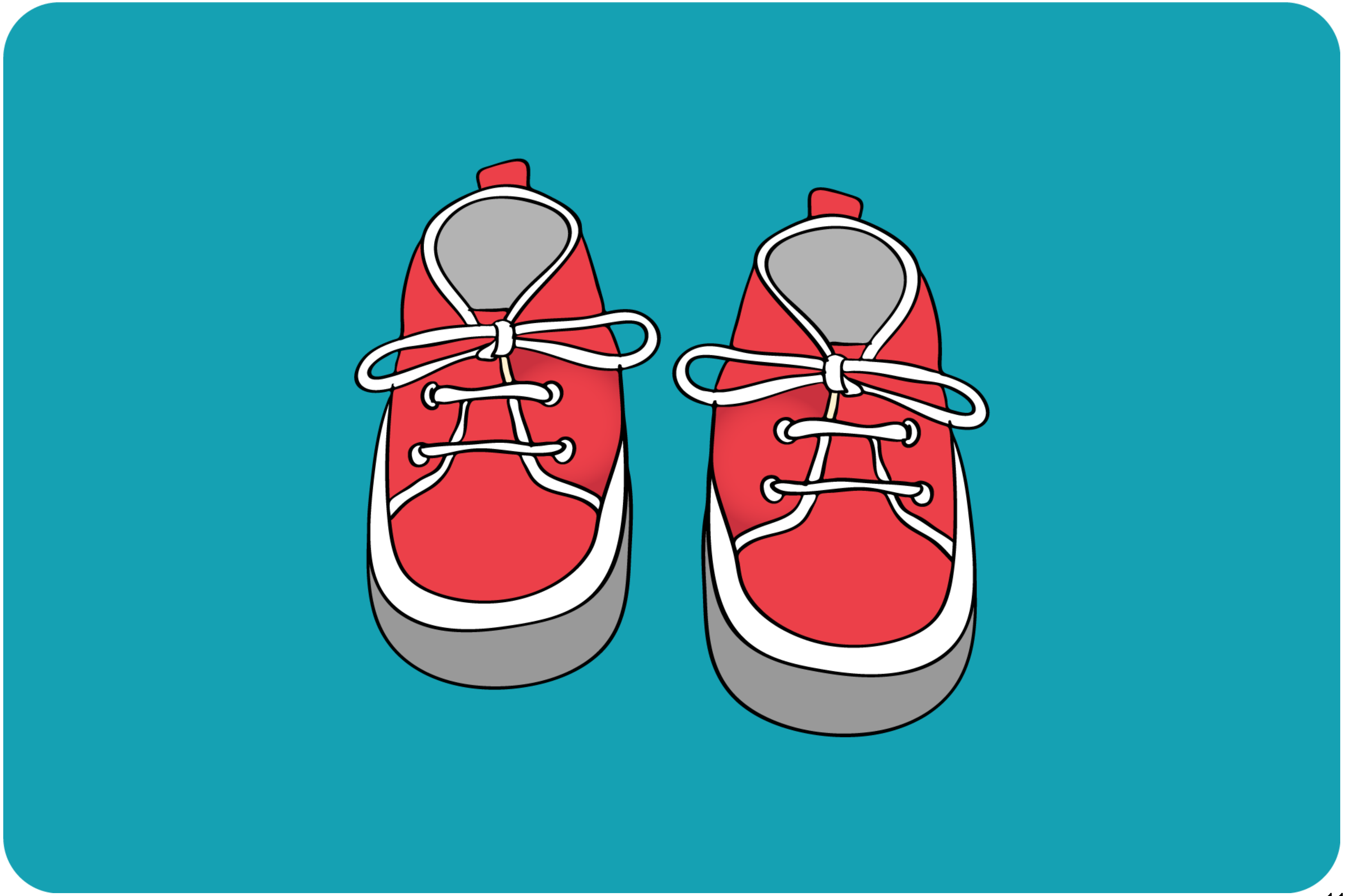

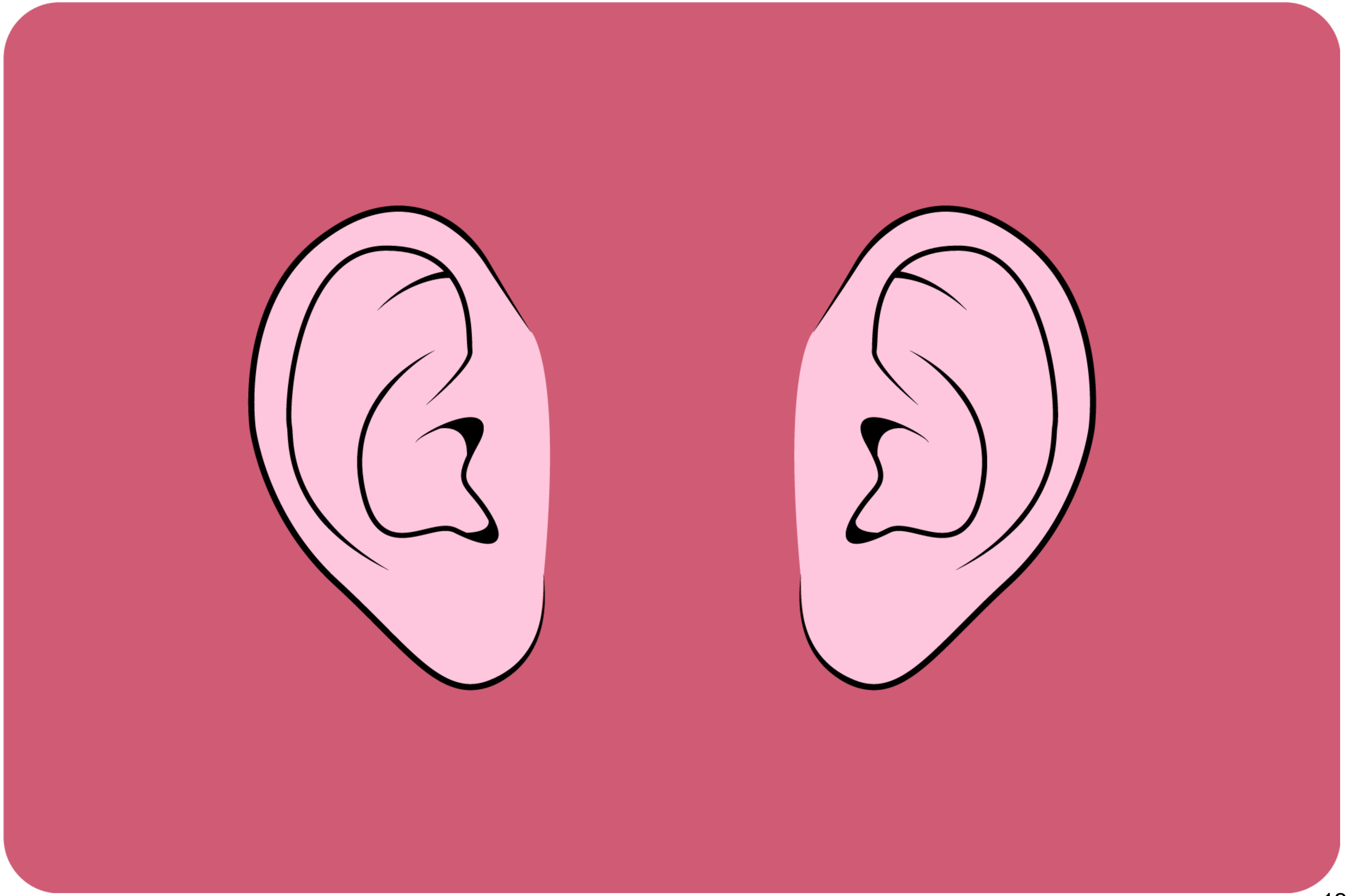

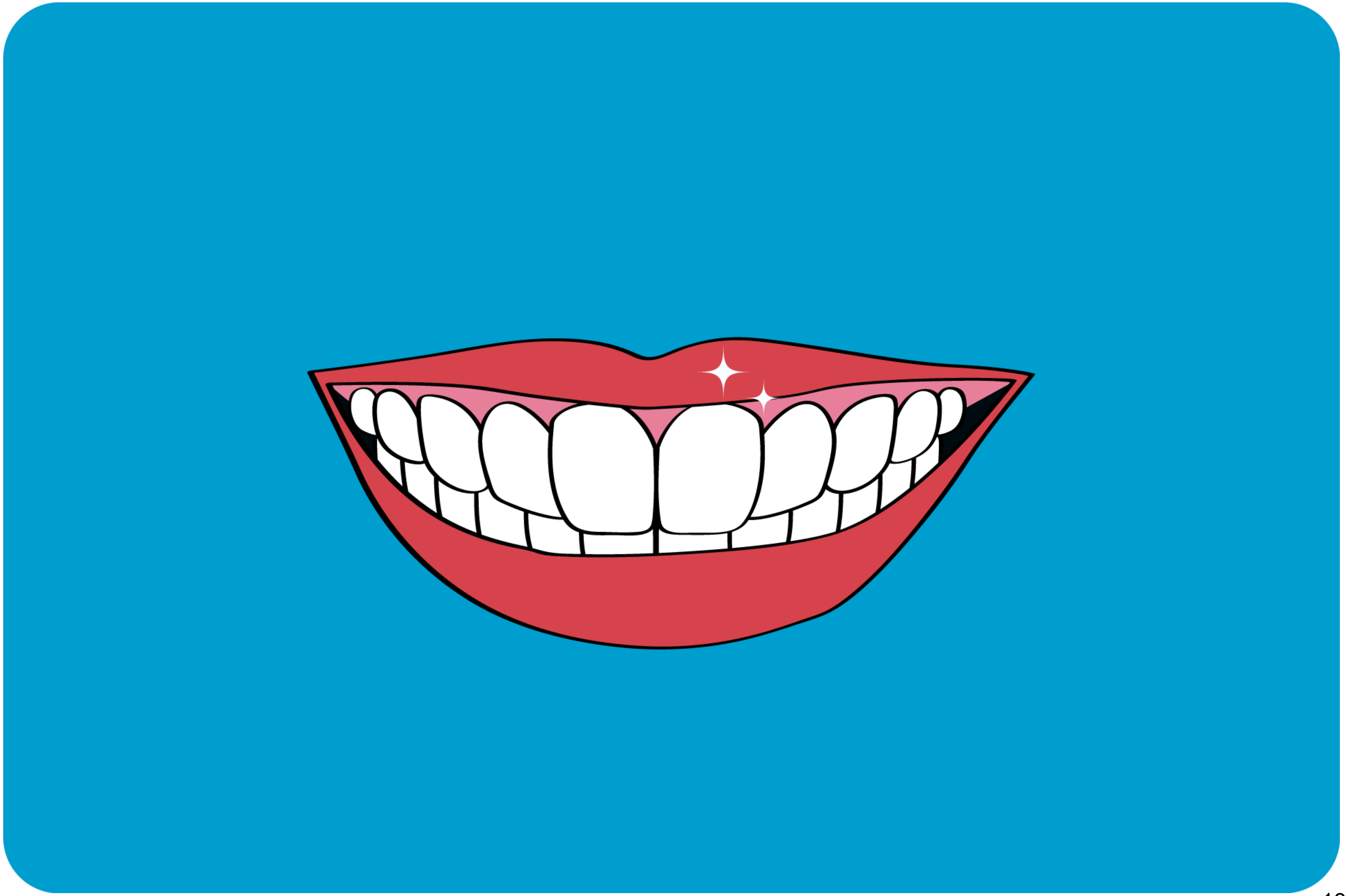

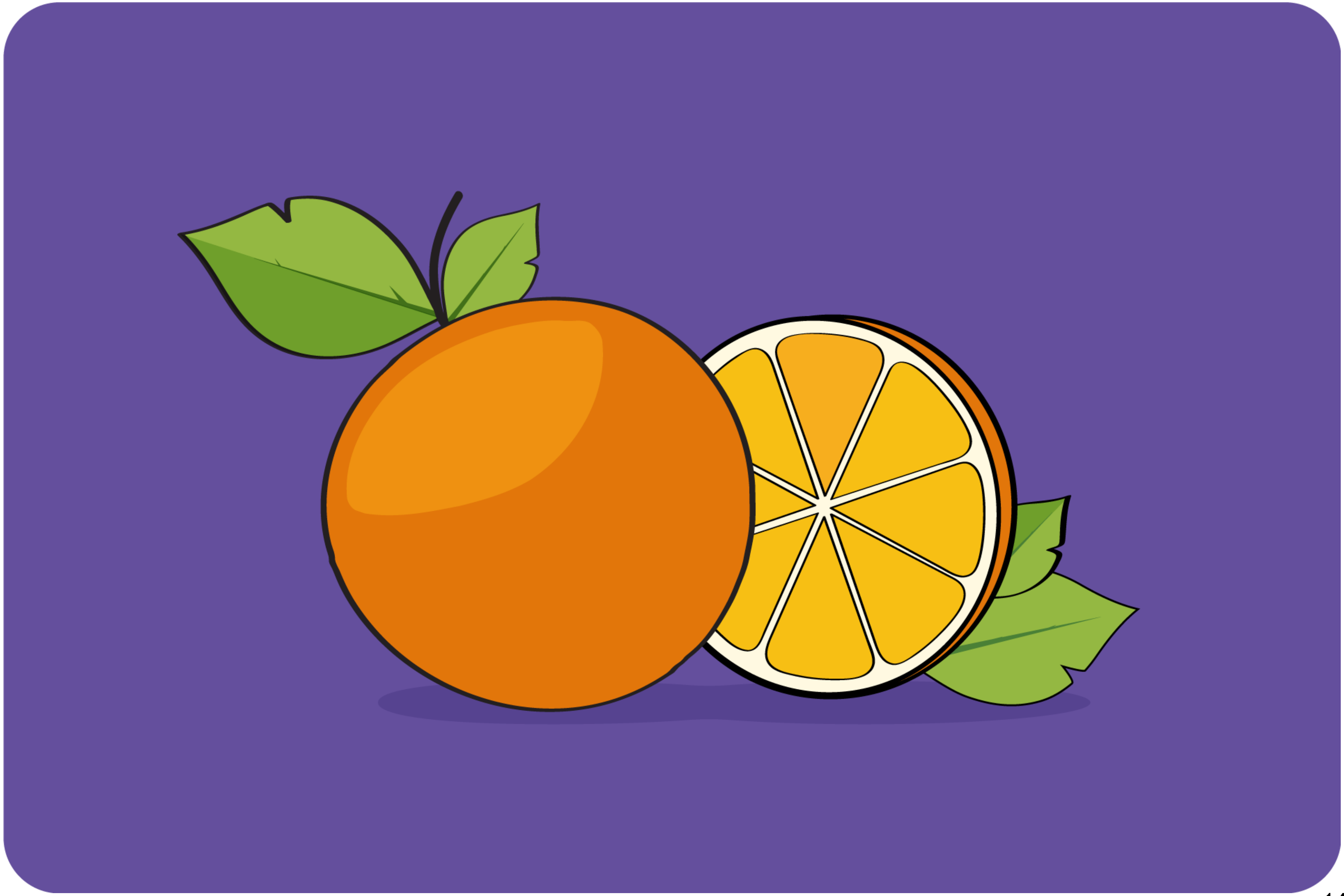

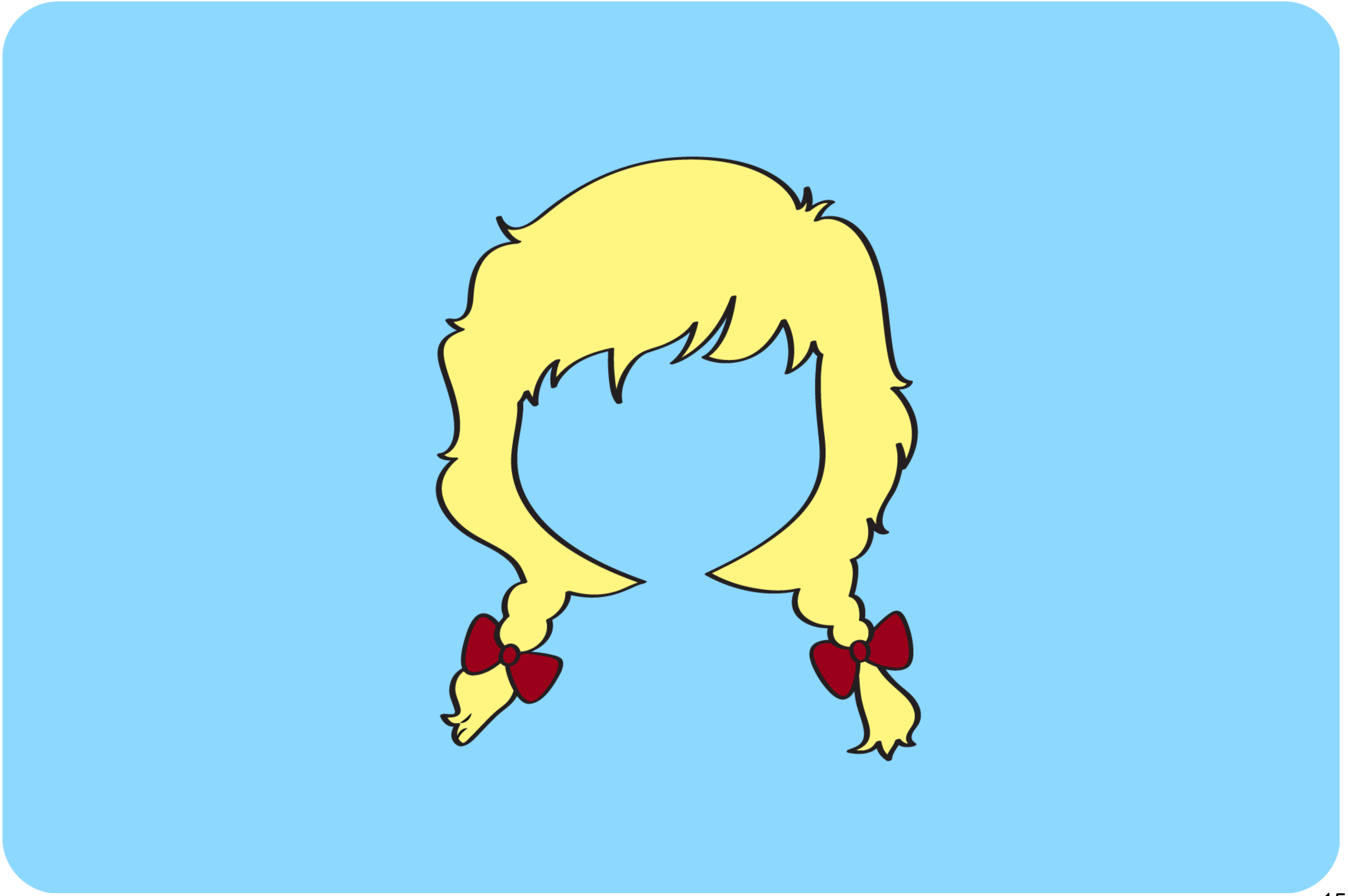

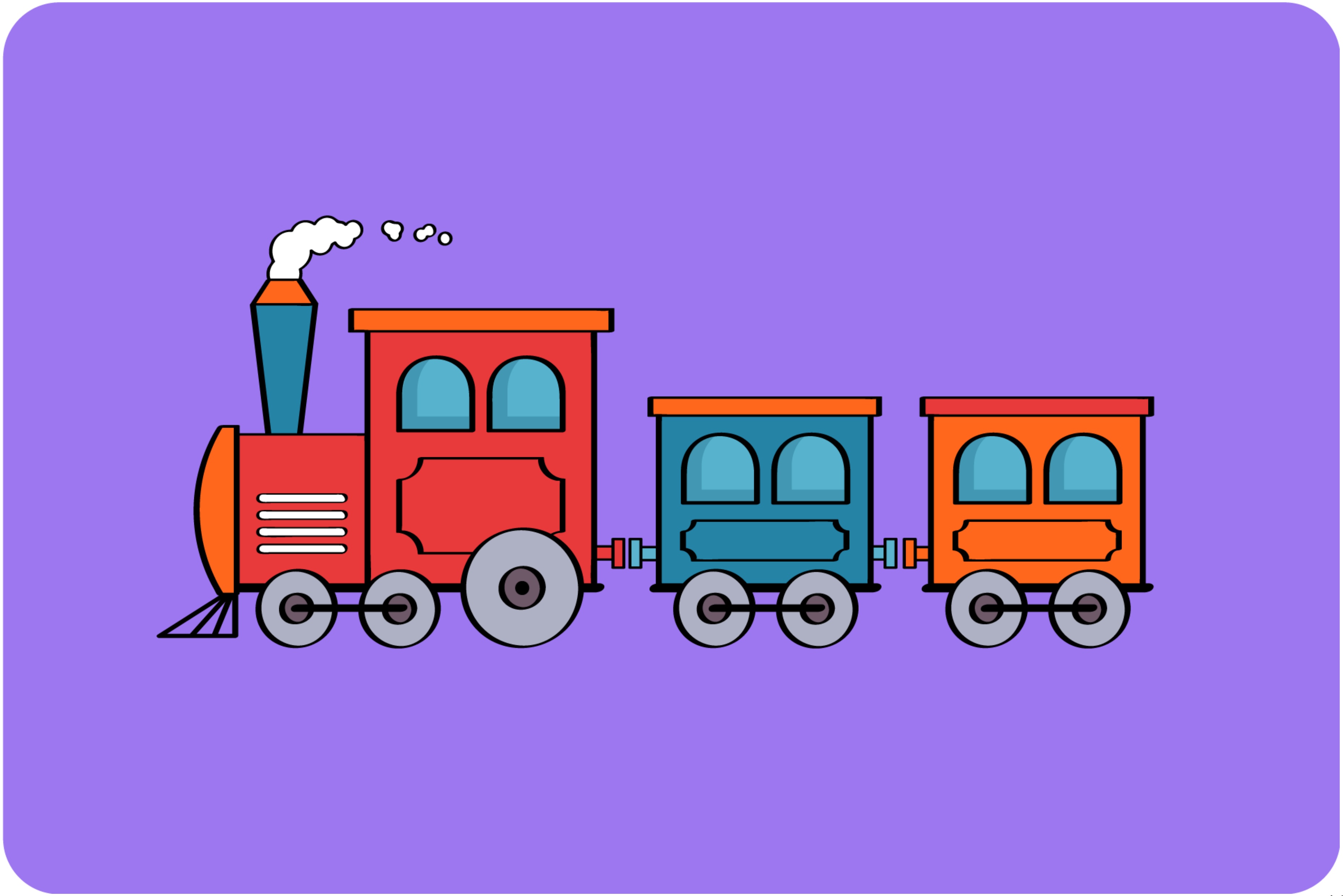

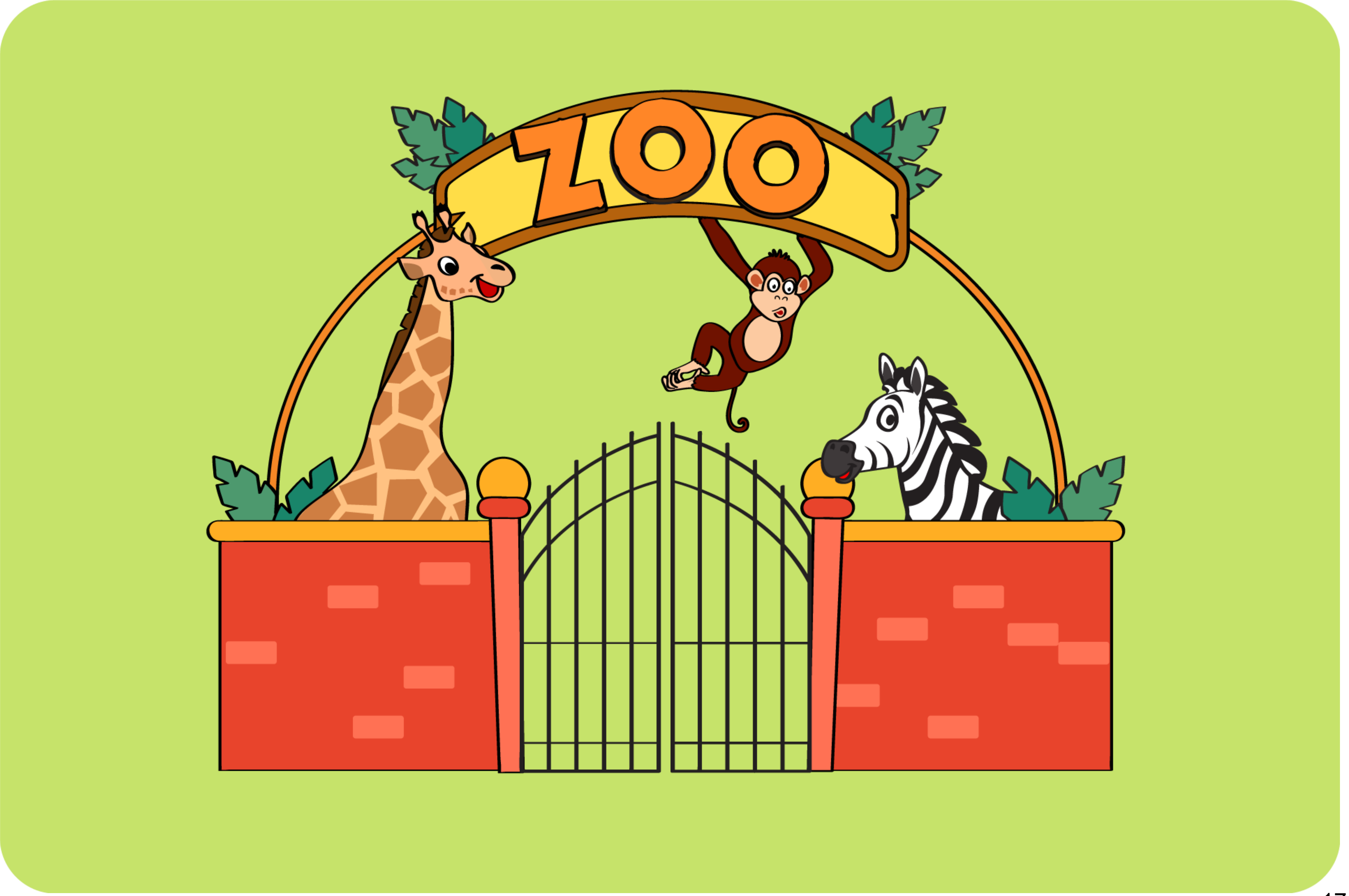
ZOO

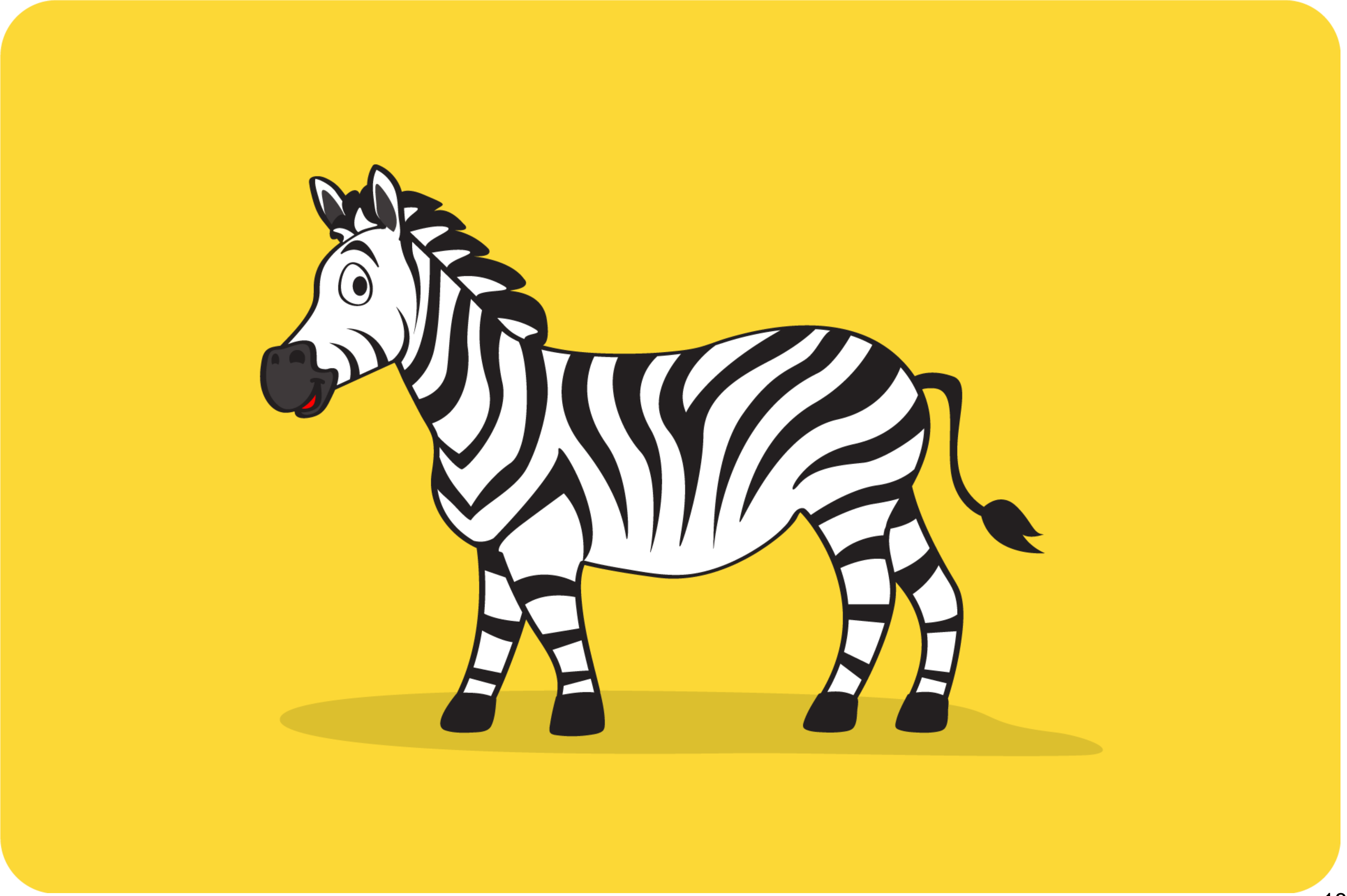

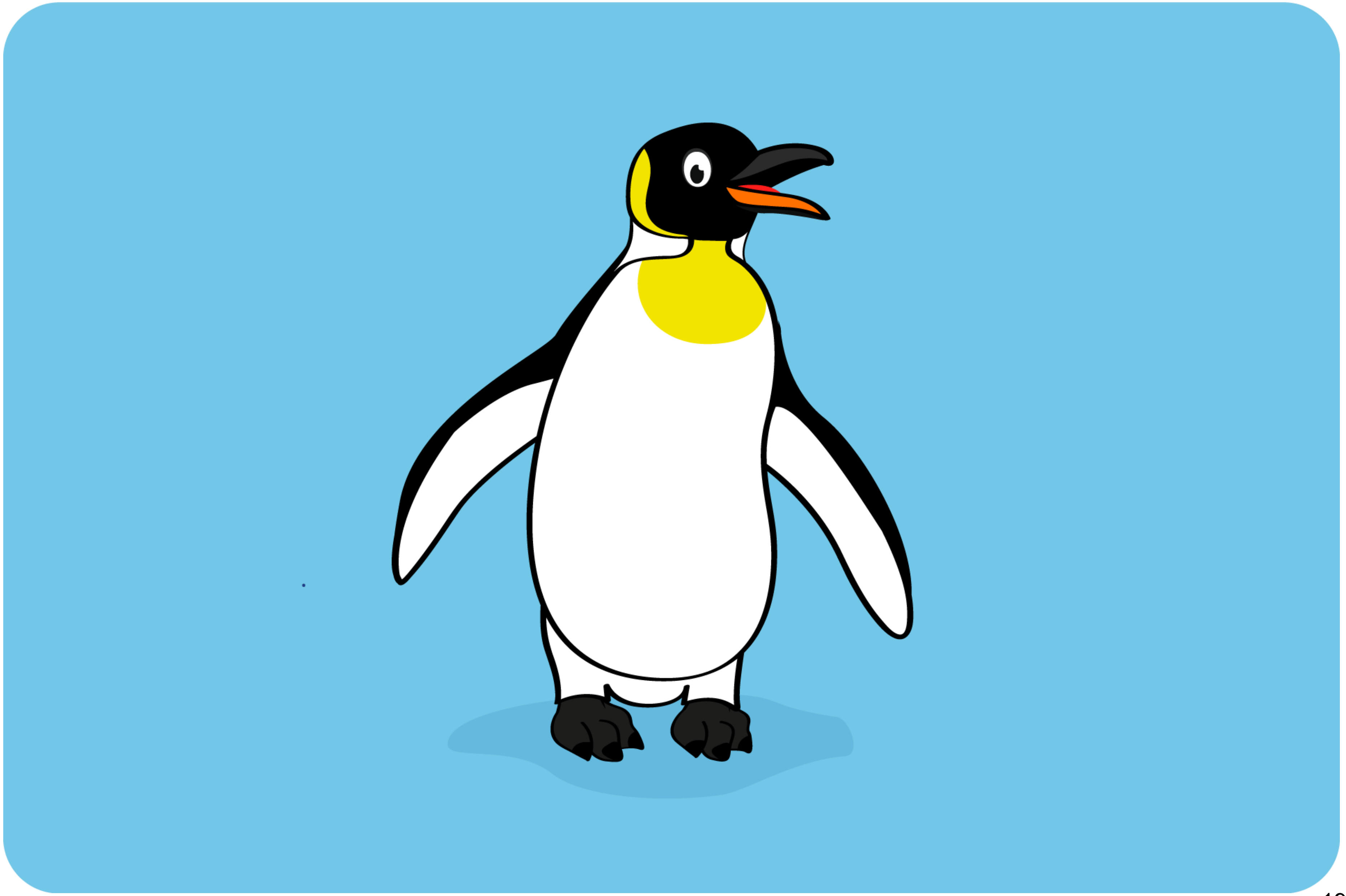

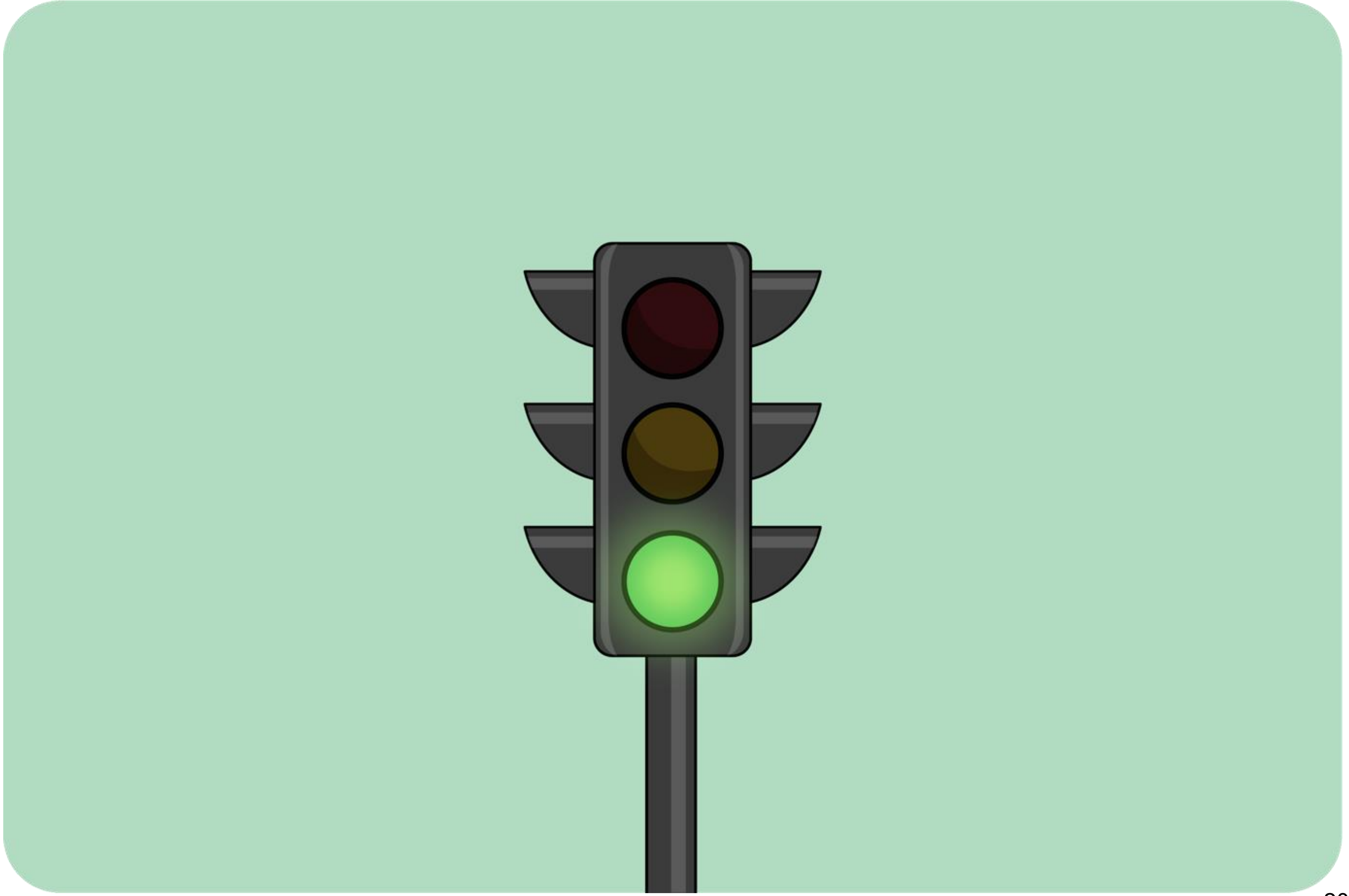

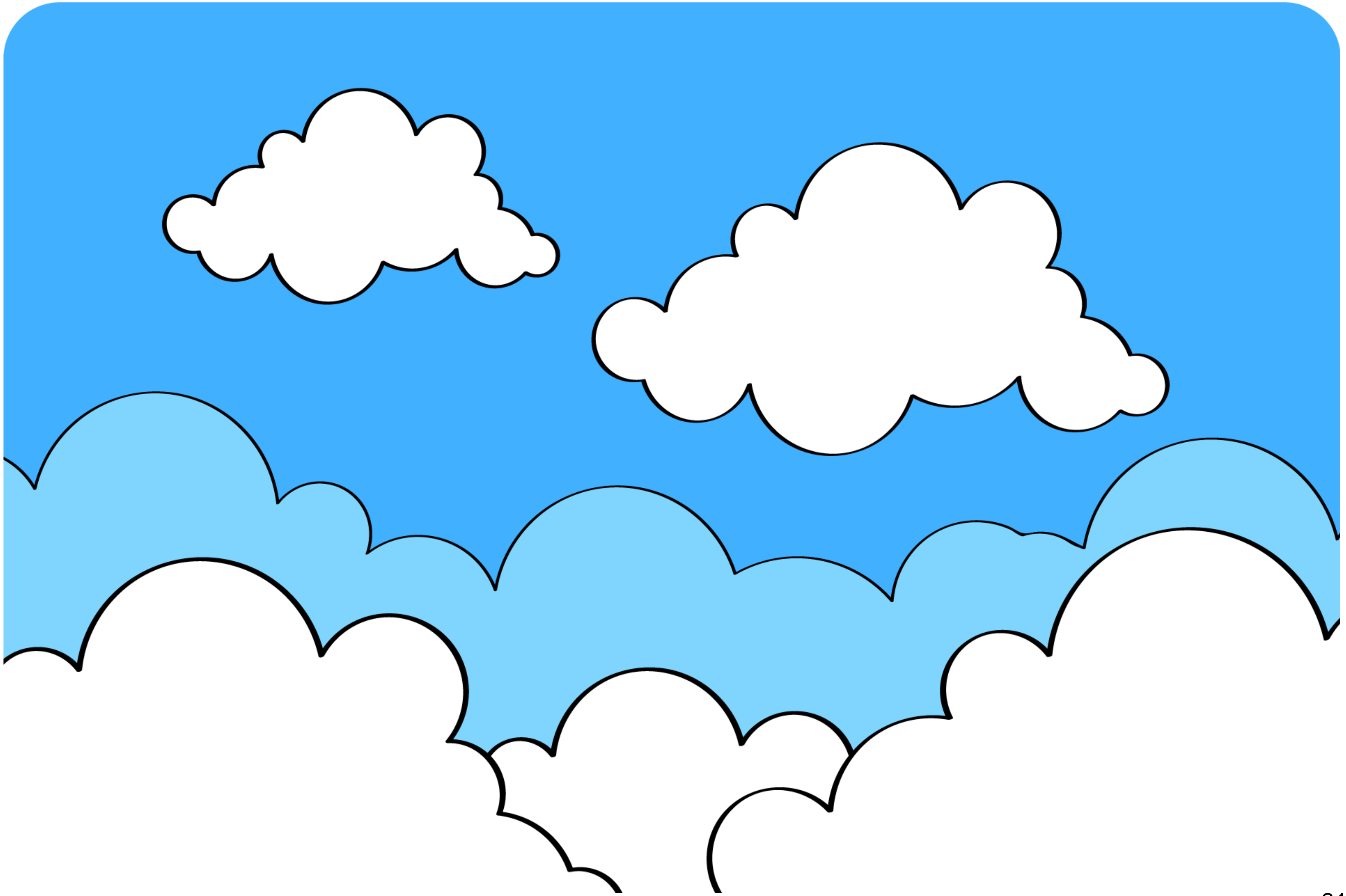

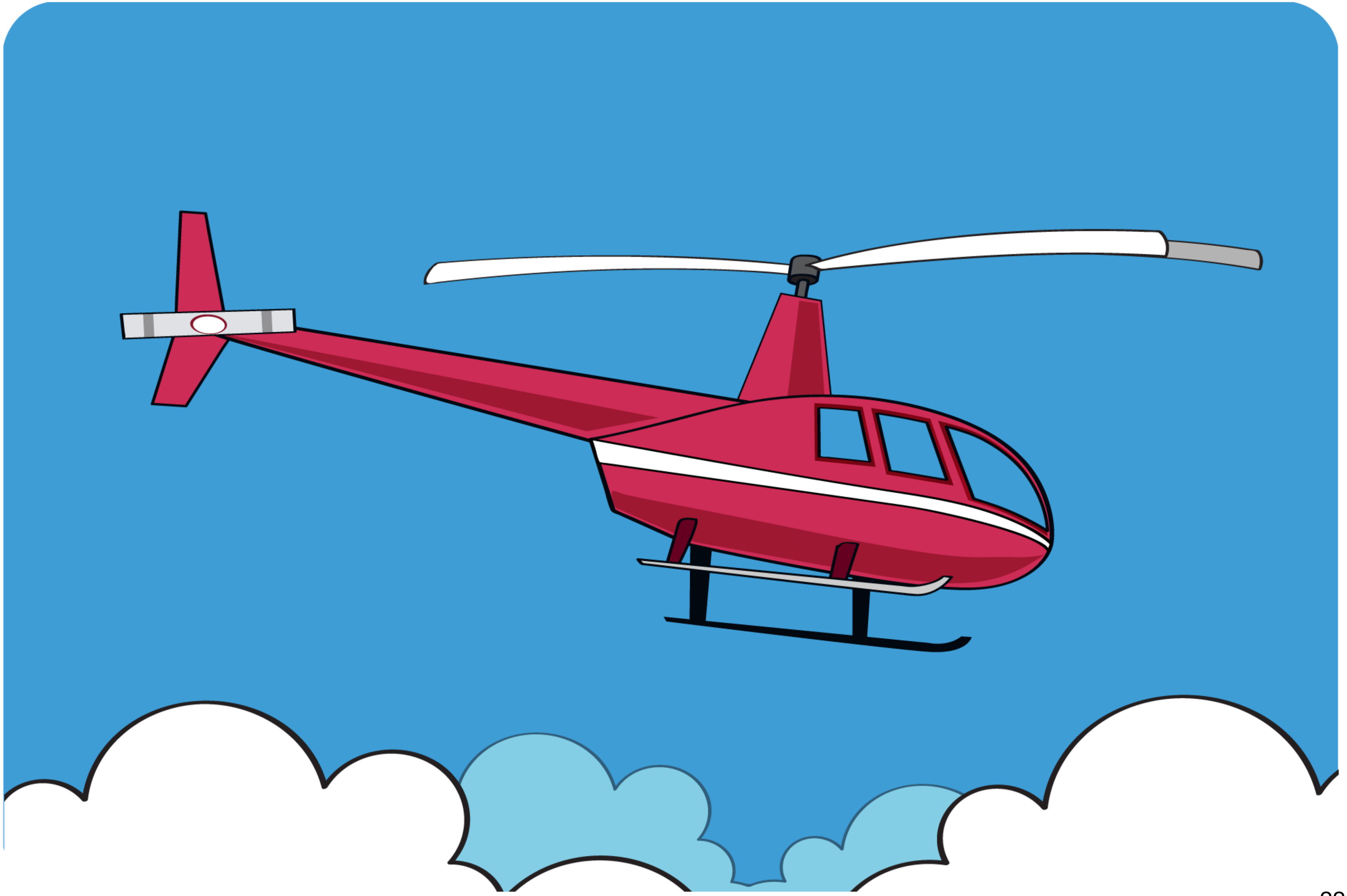

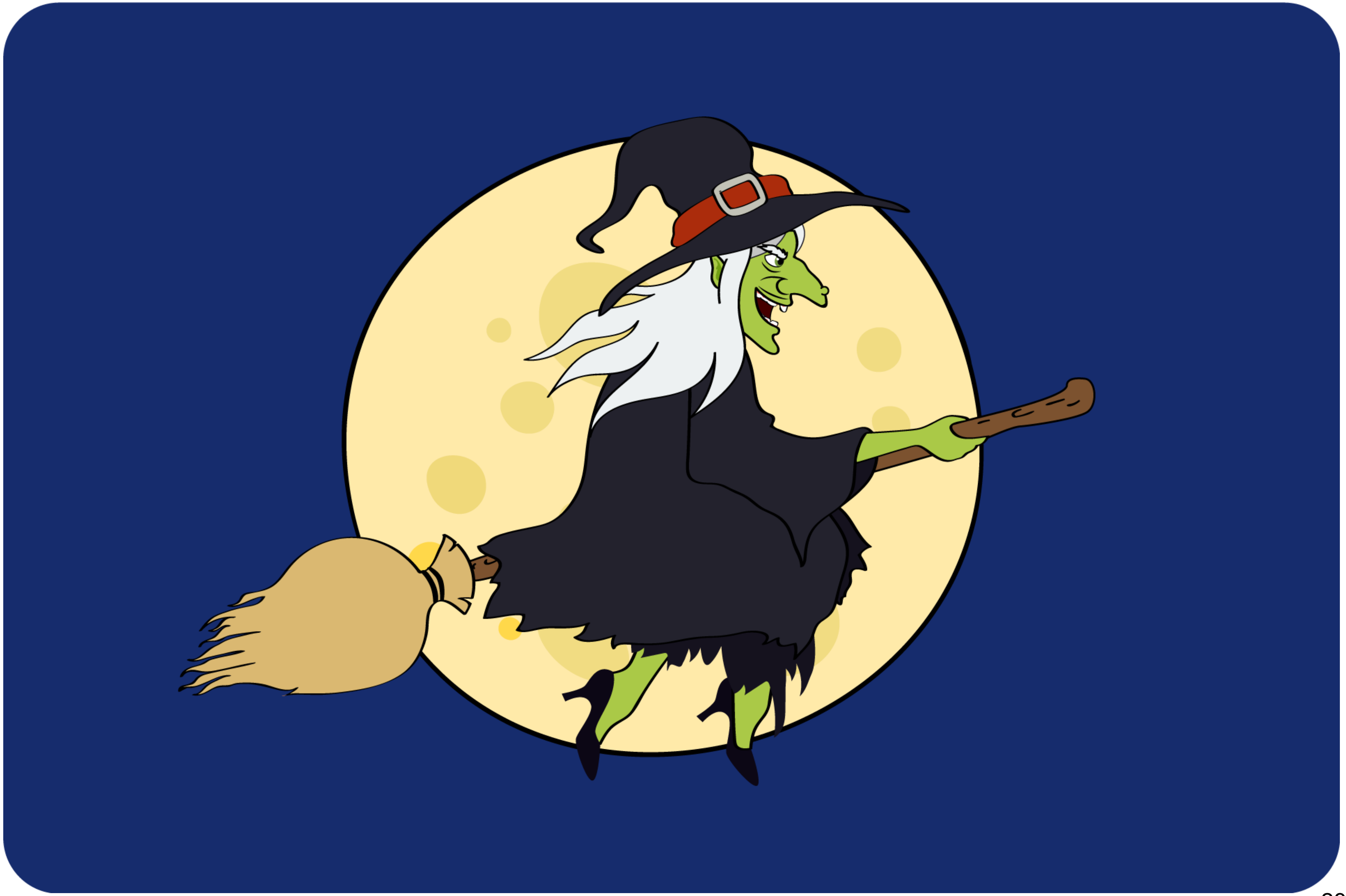

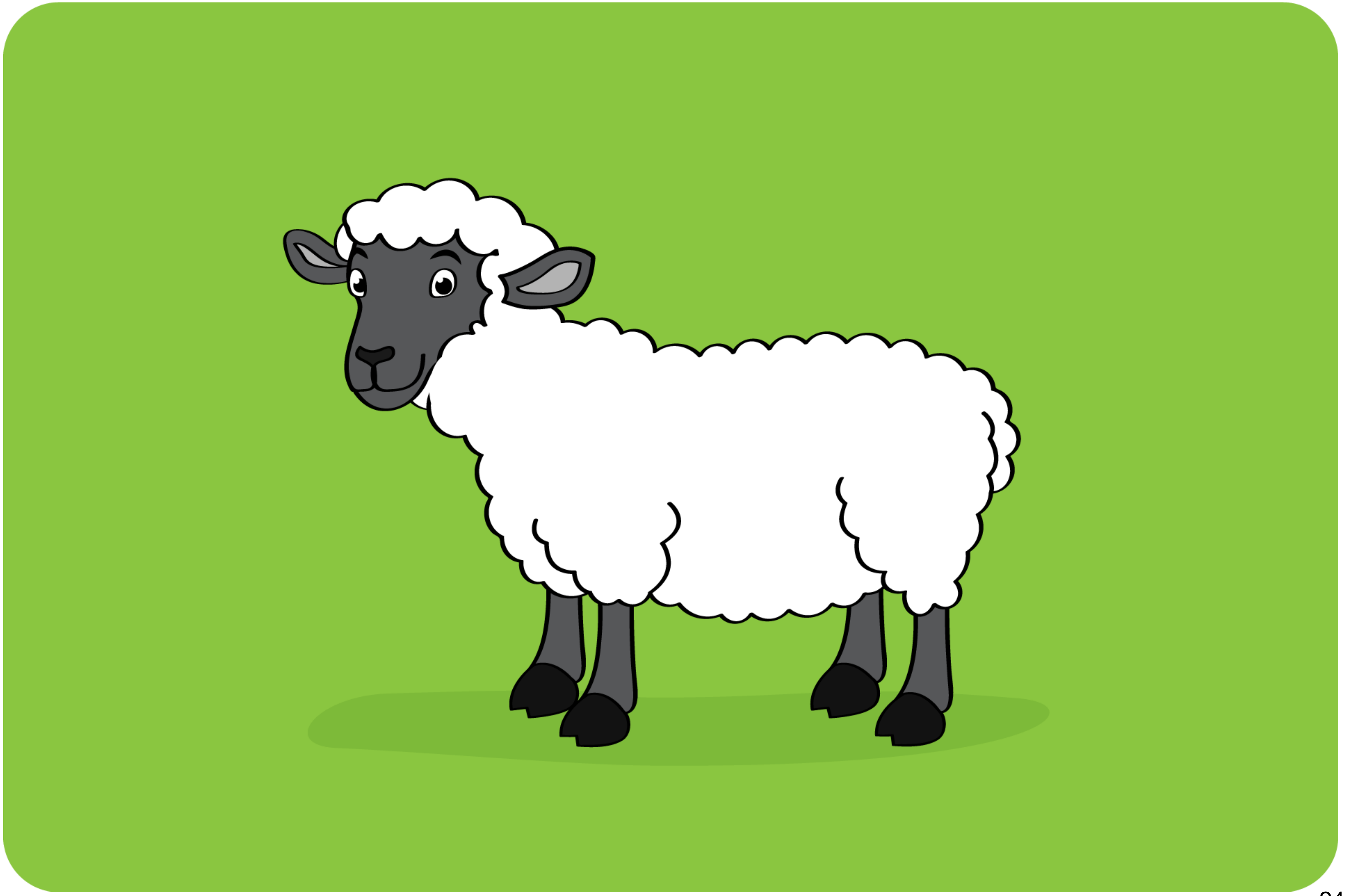

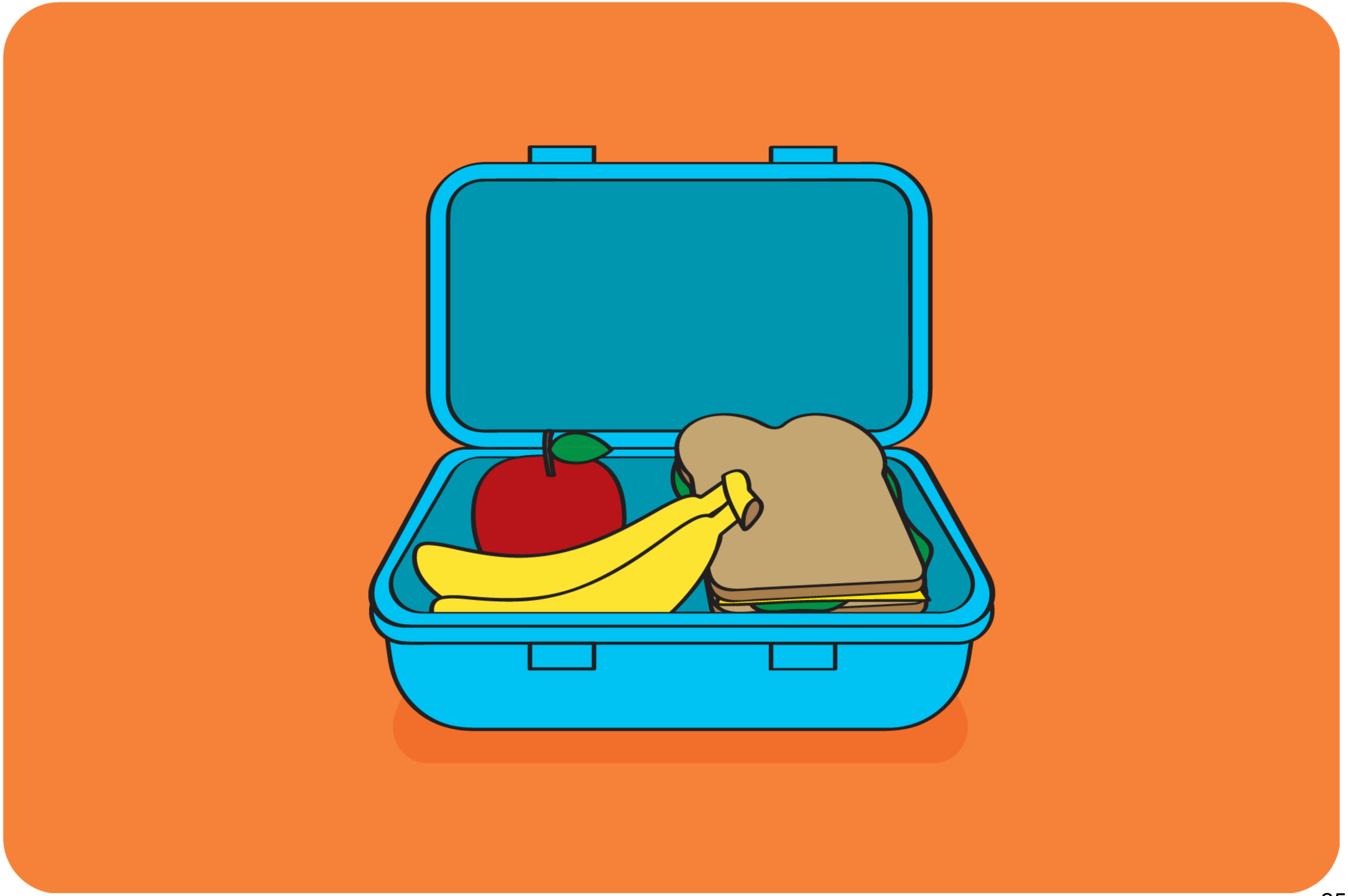

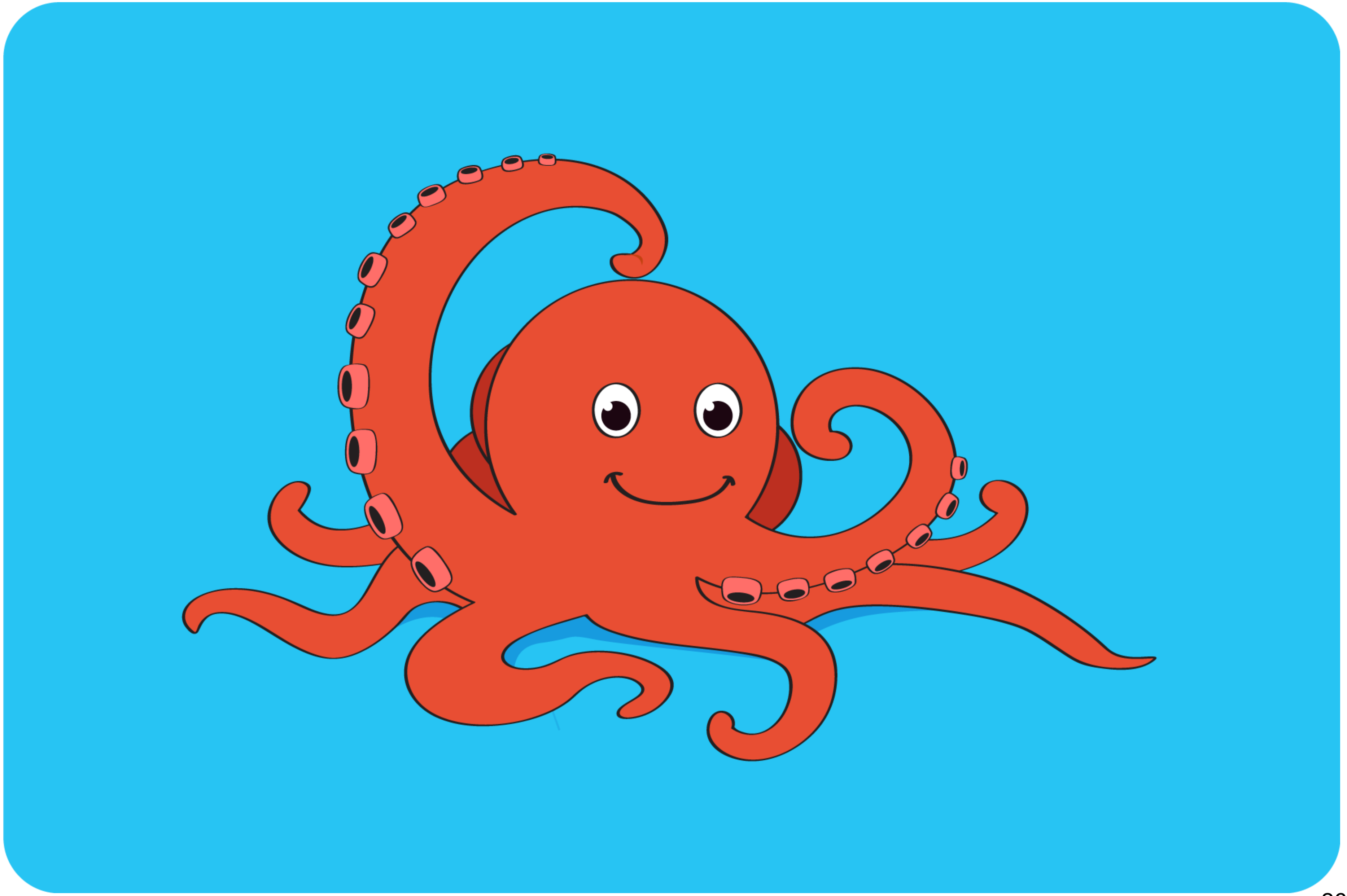

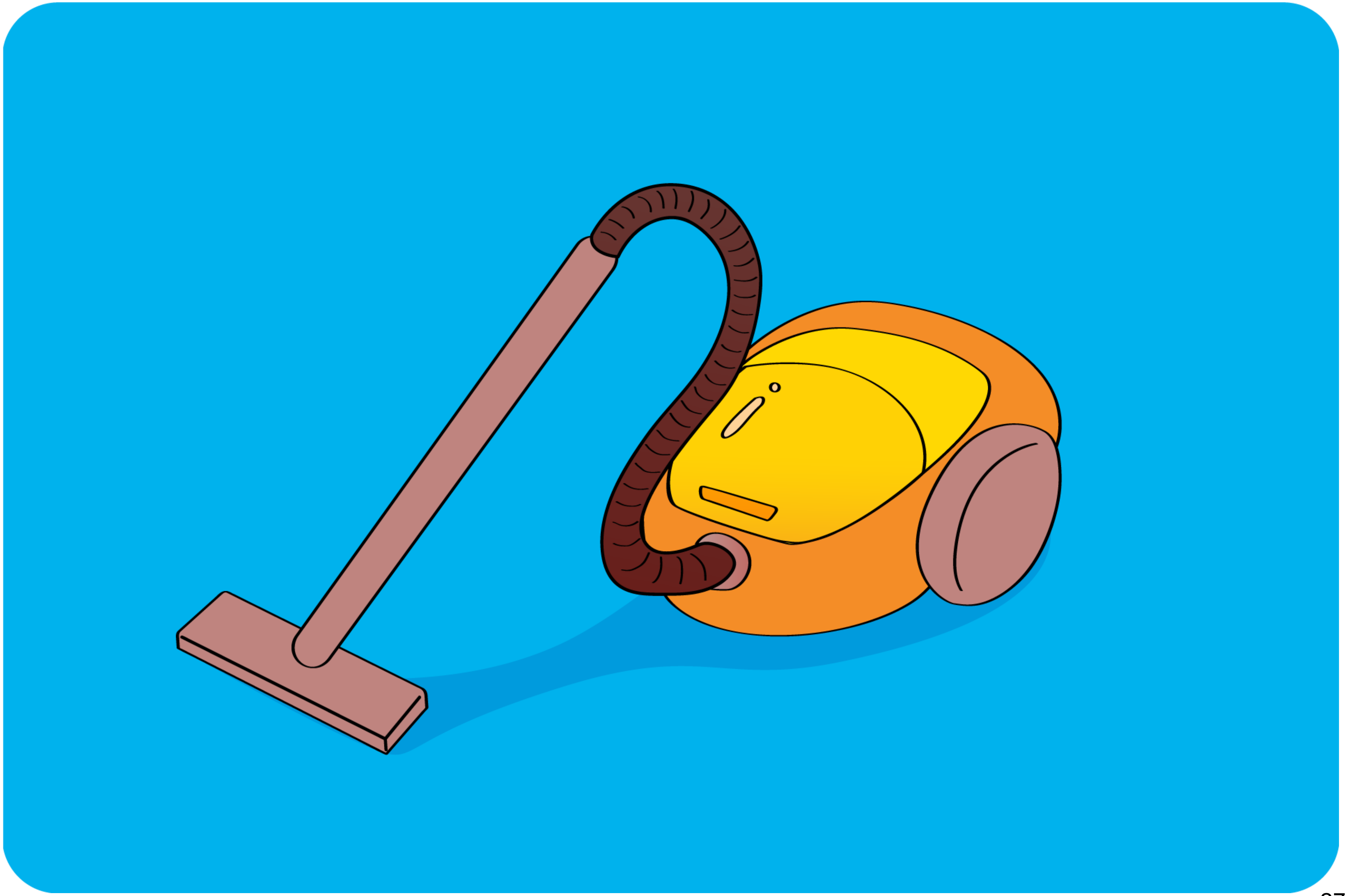

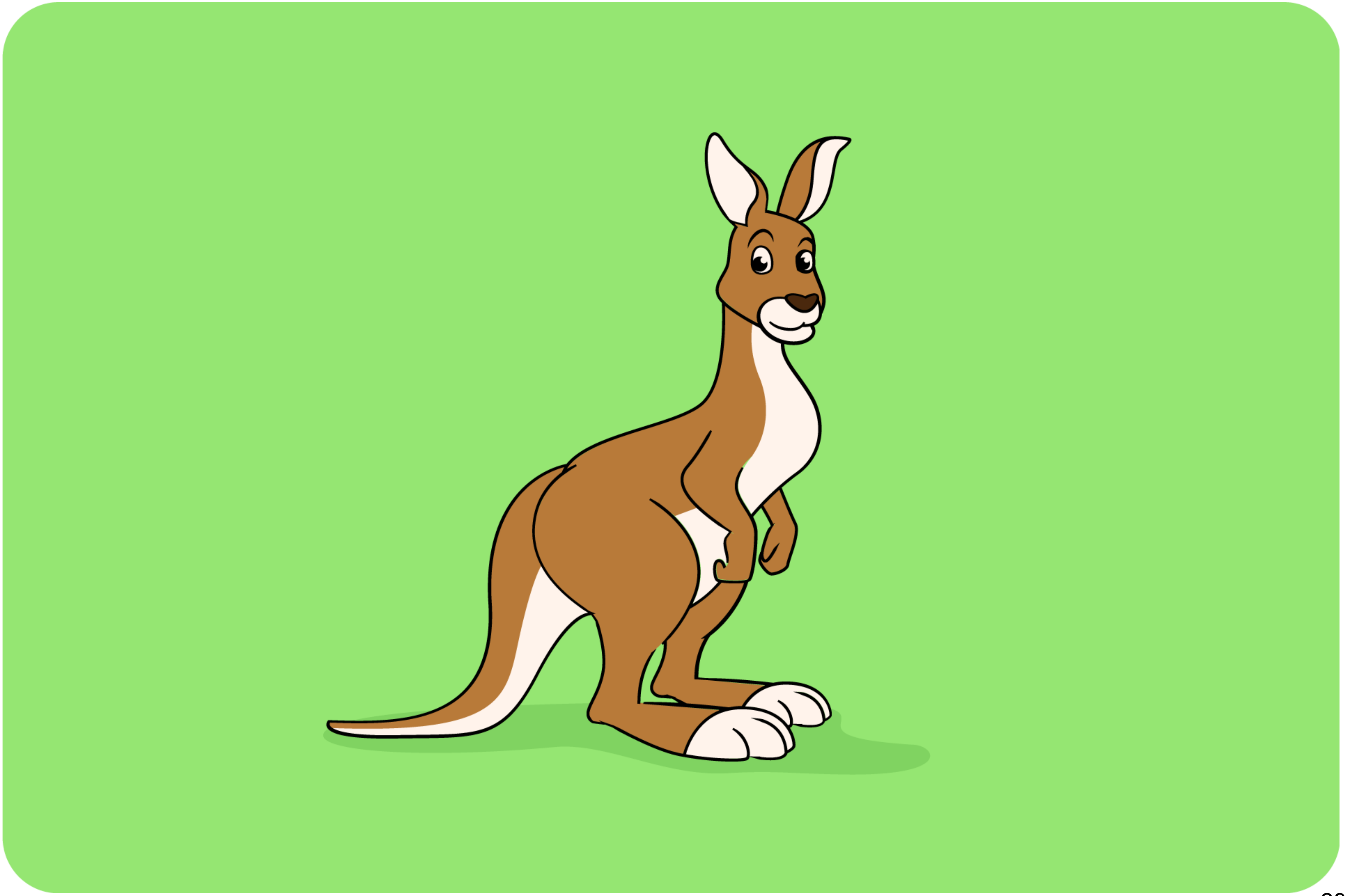

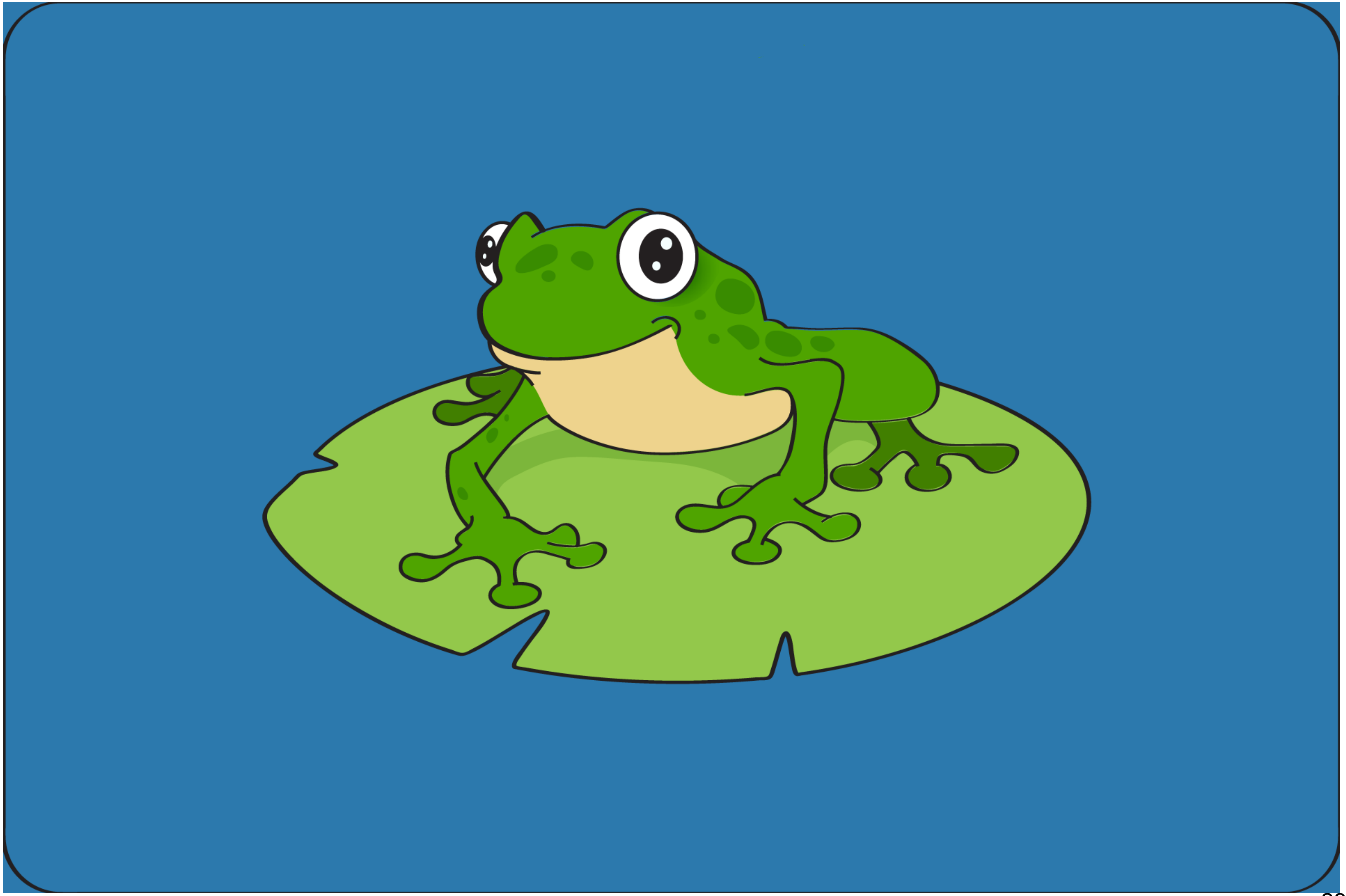

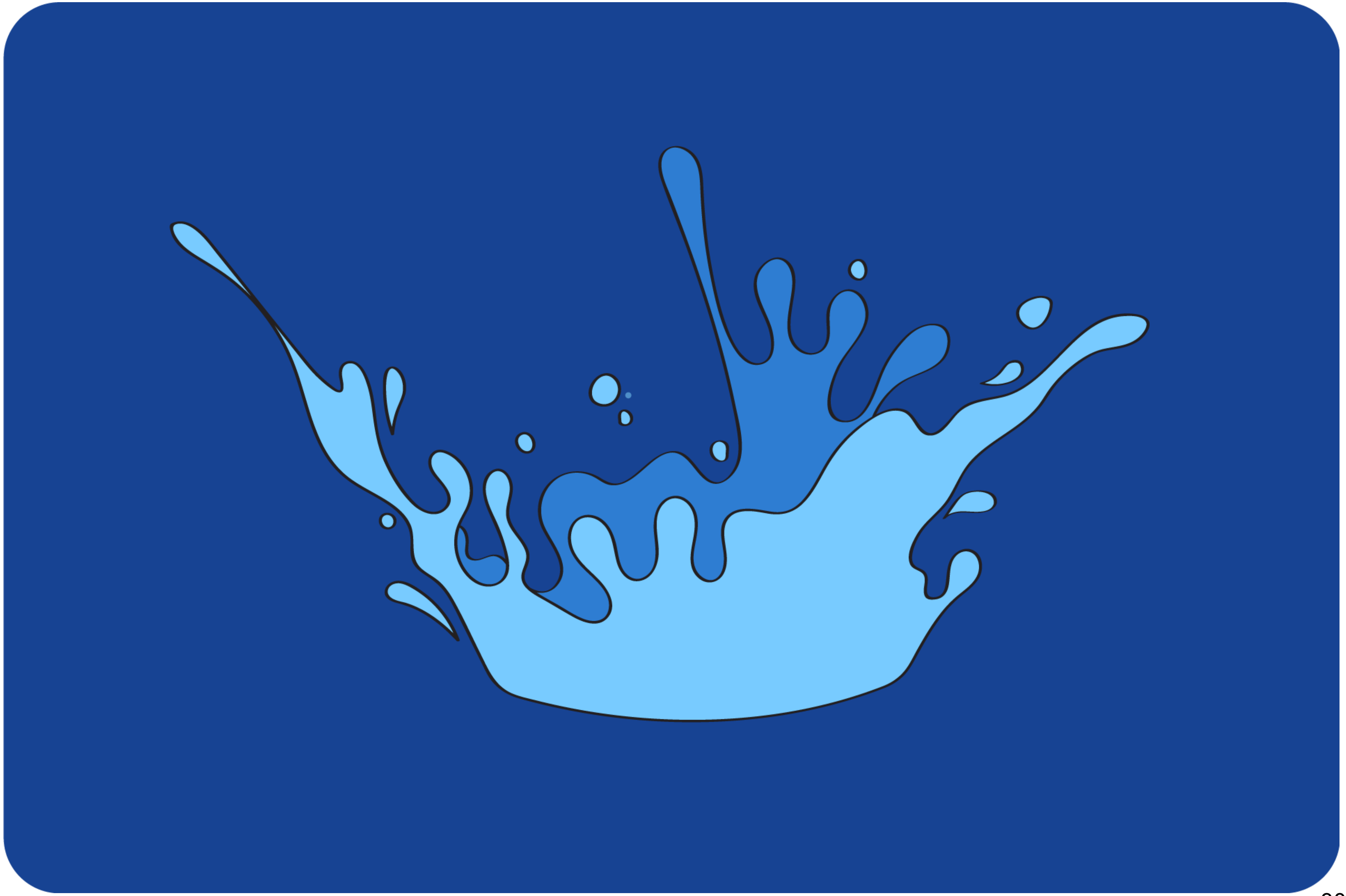

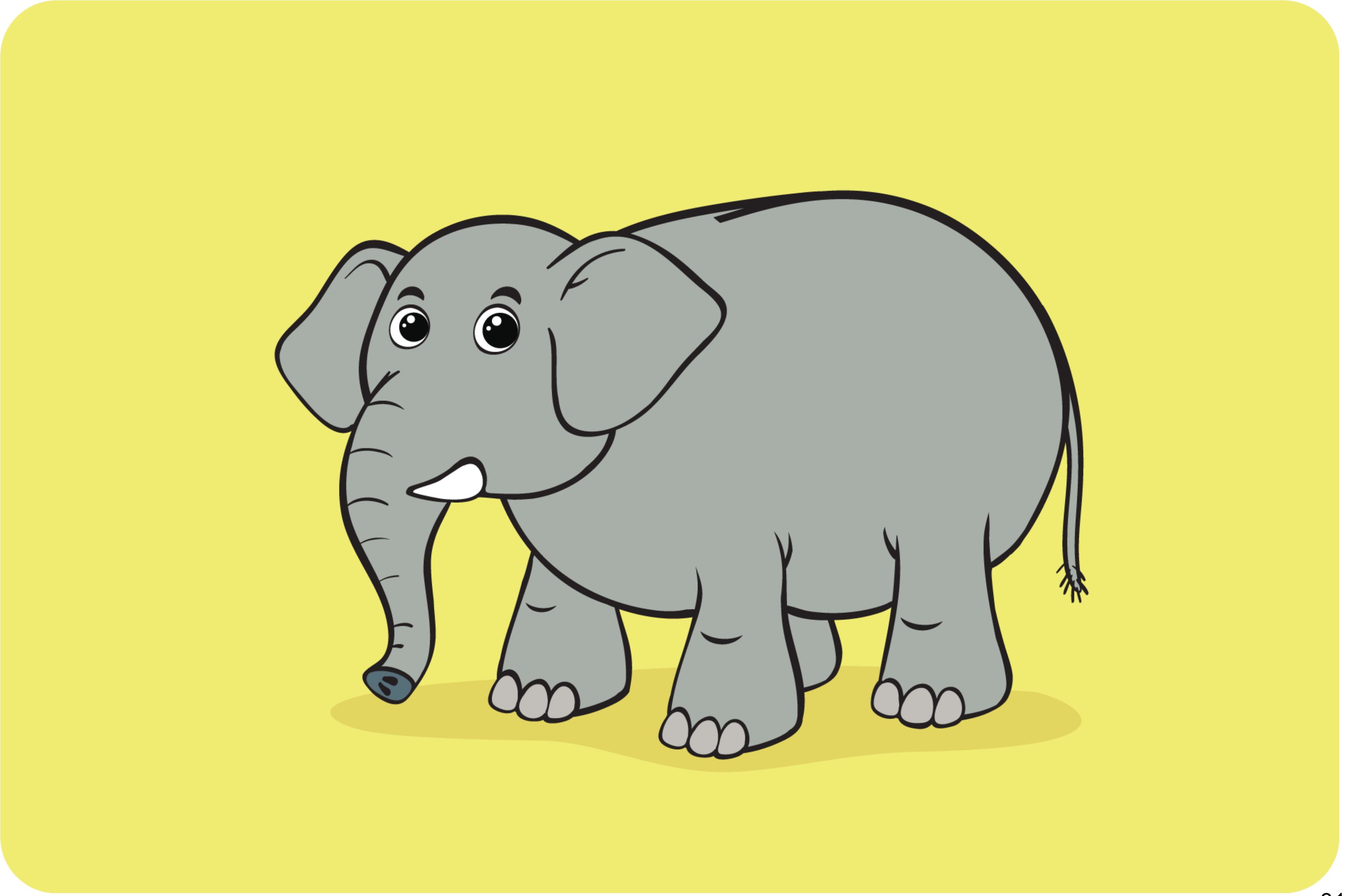

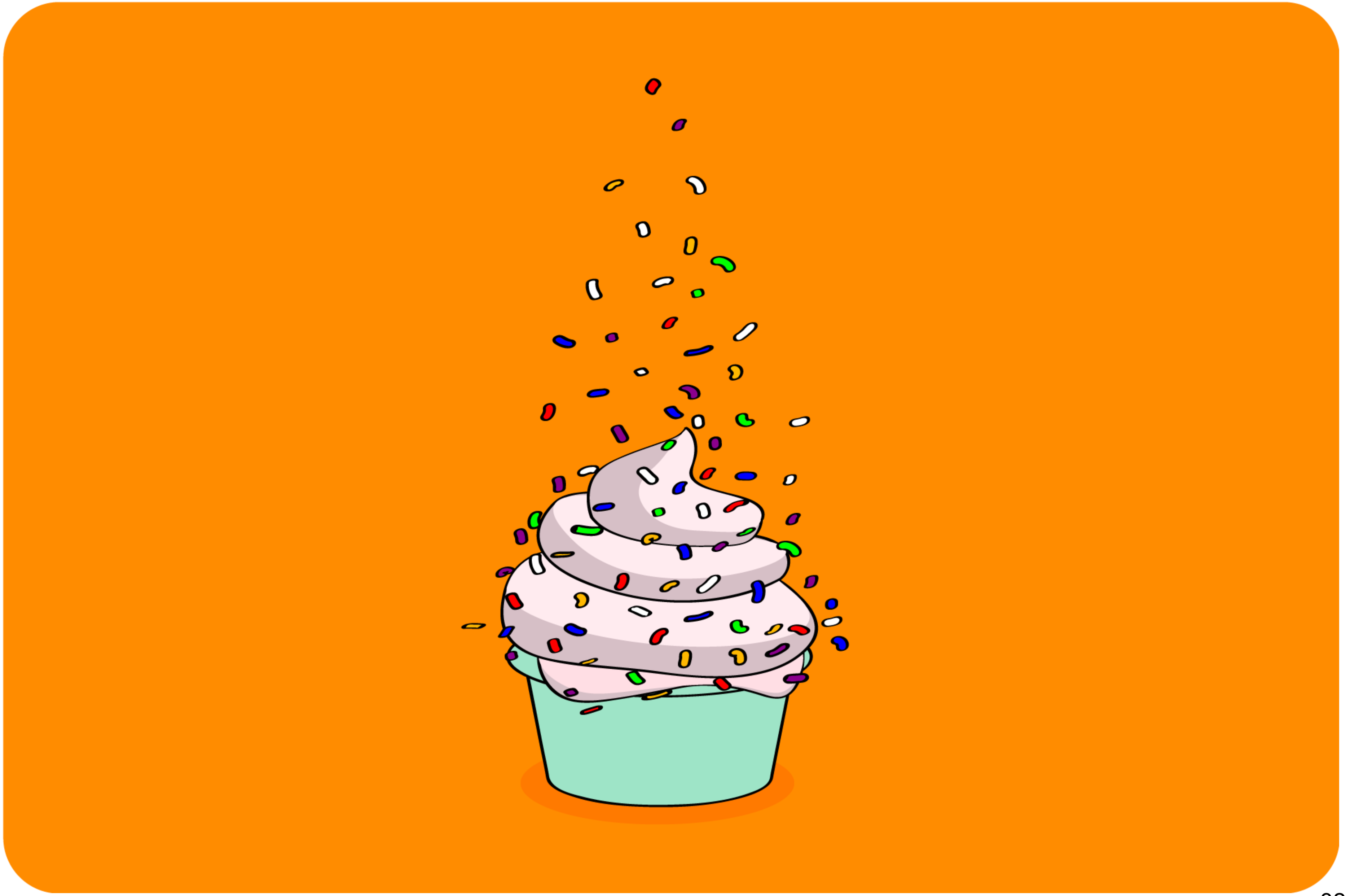

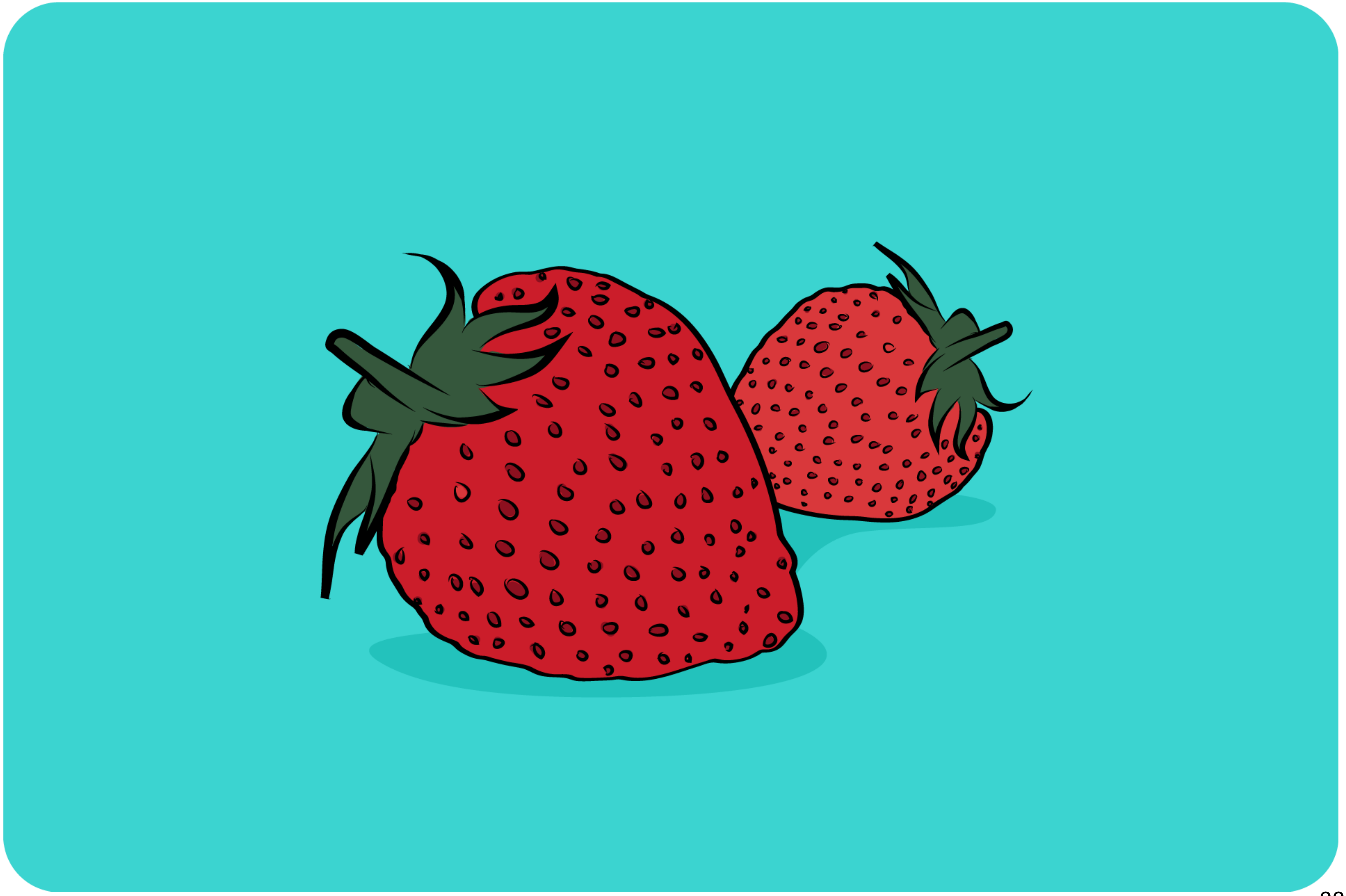

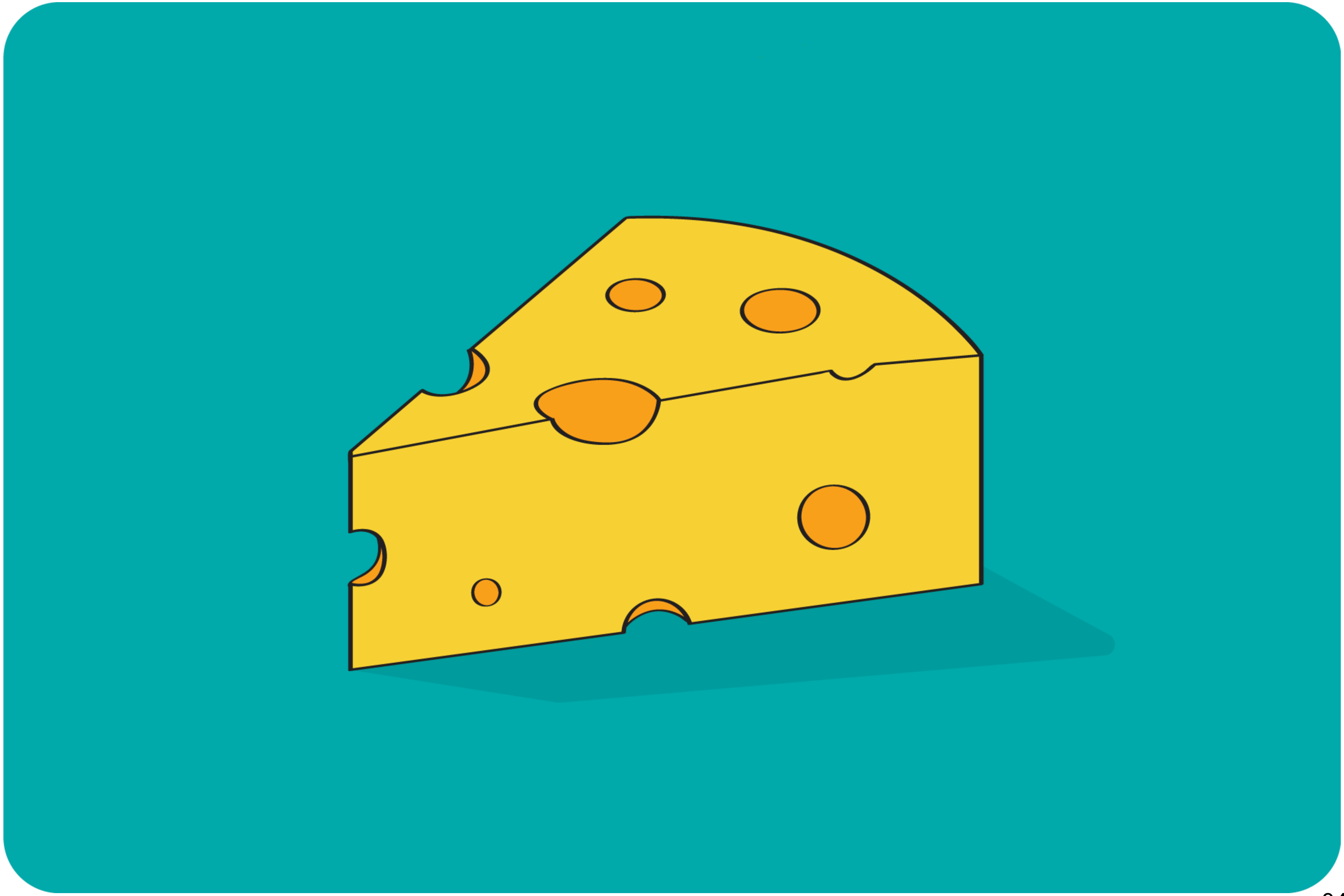

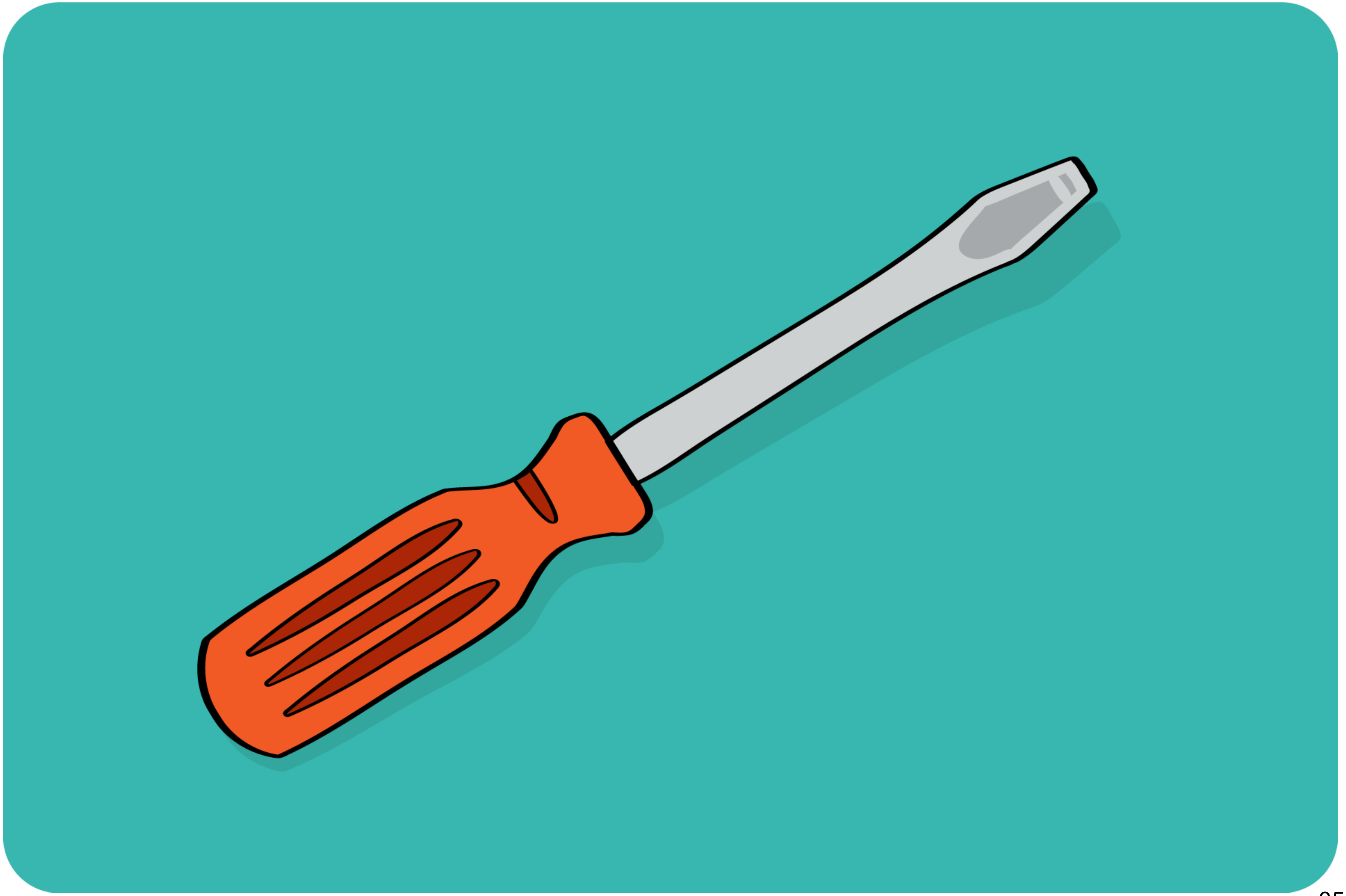

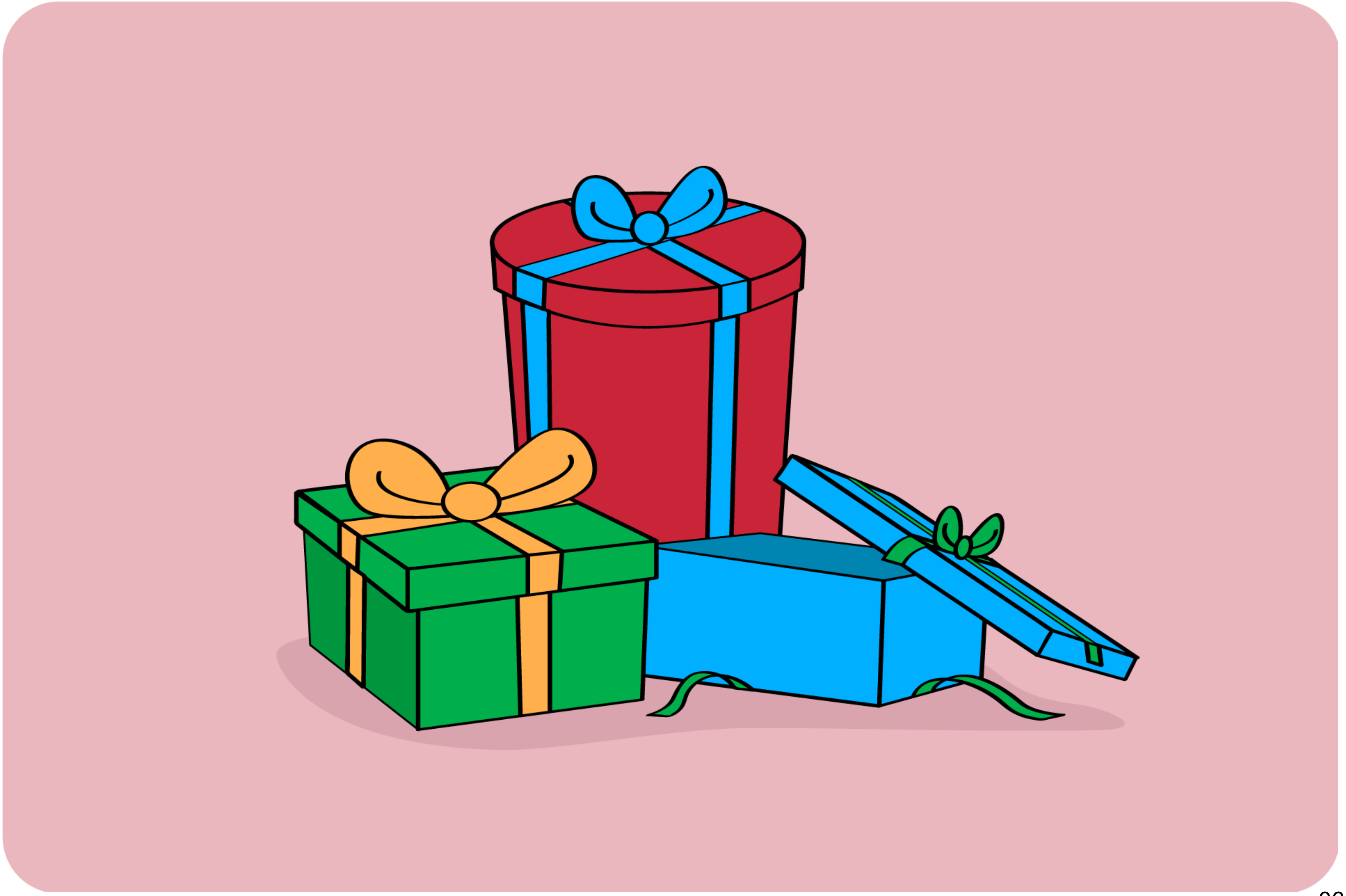

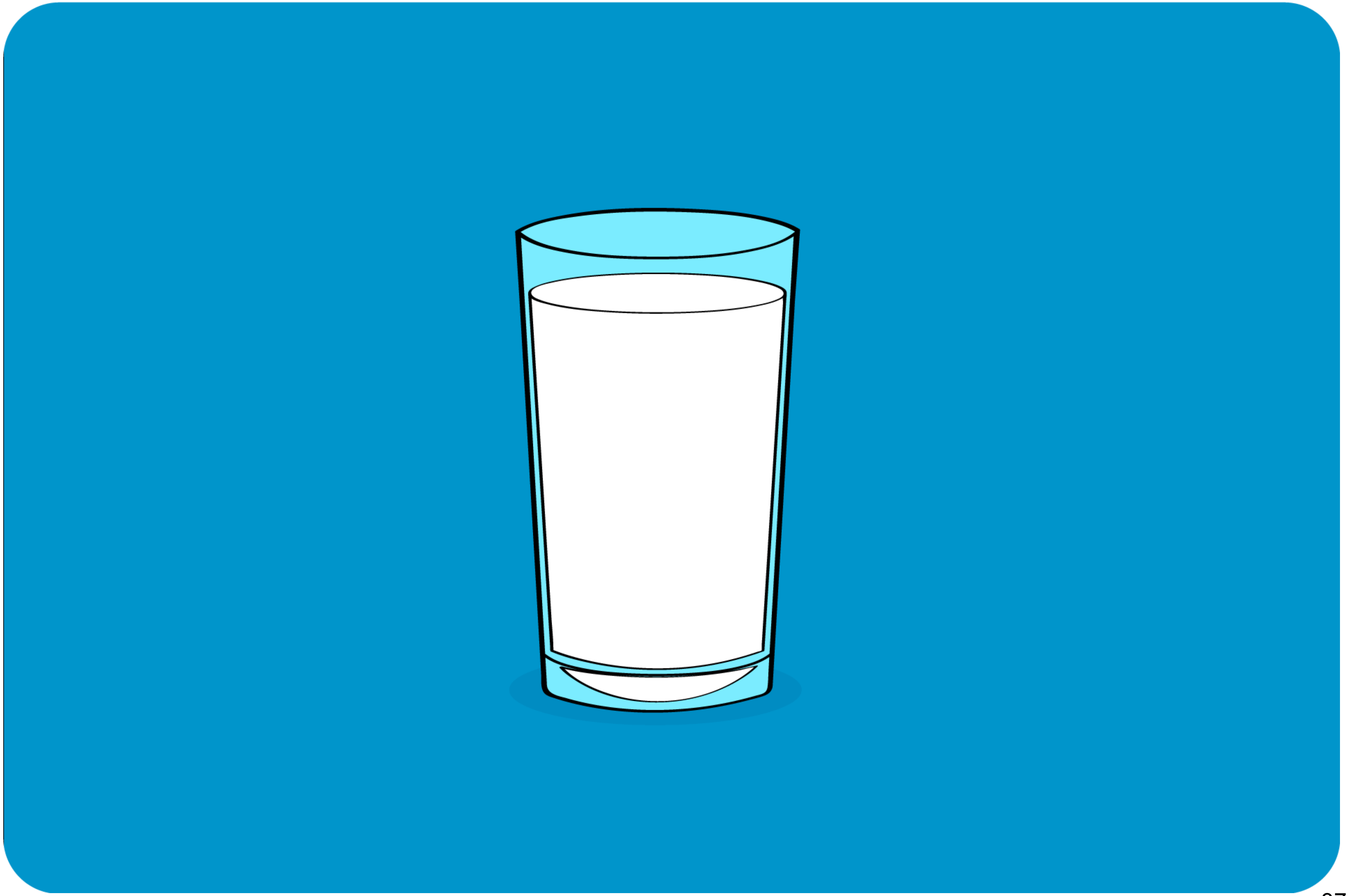

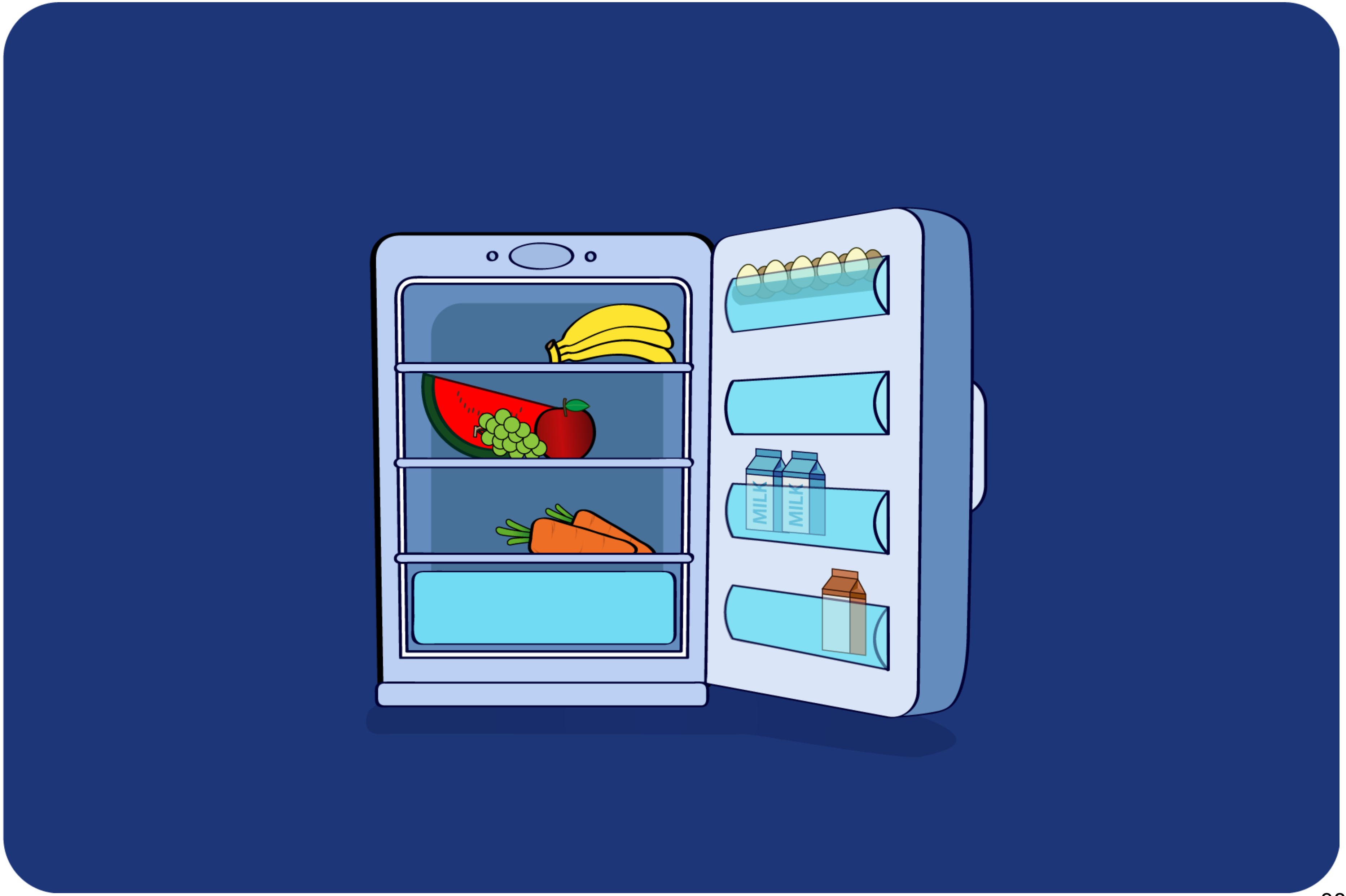
MILK
MILK

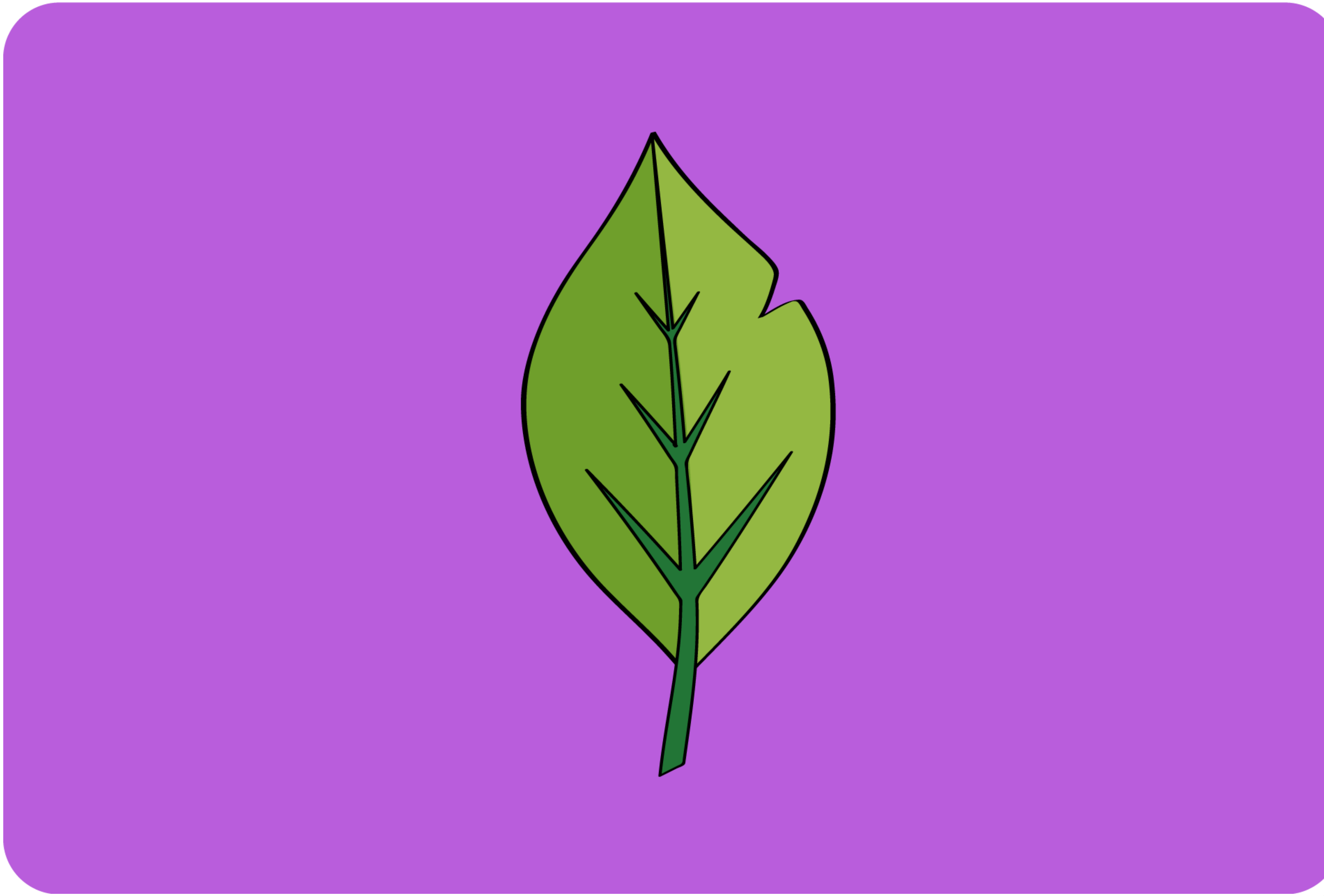

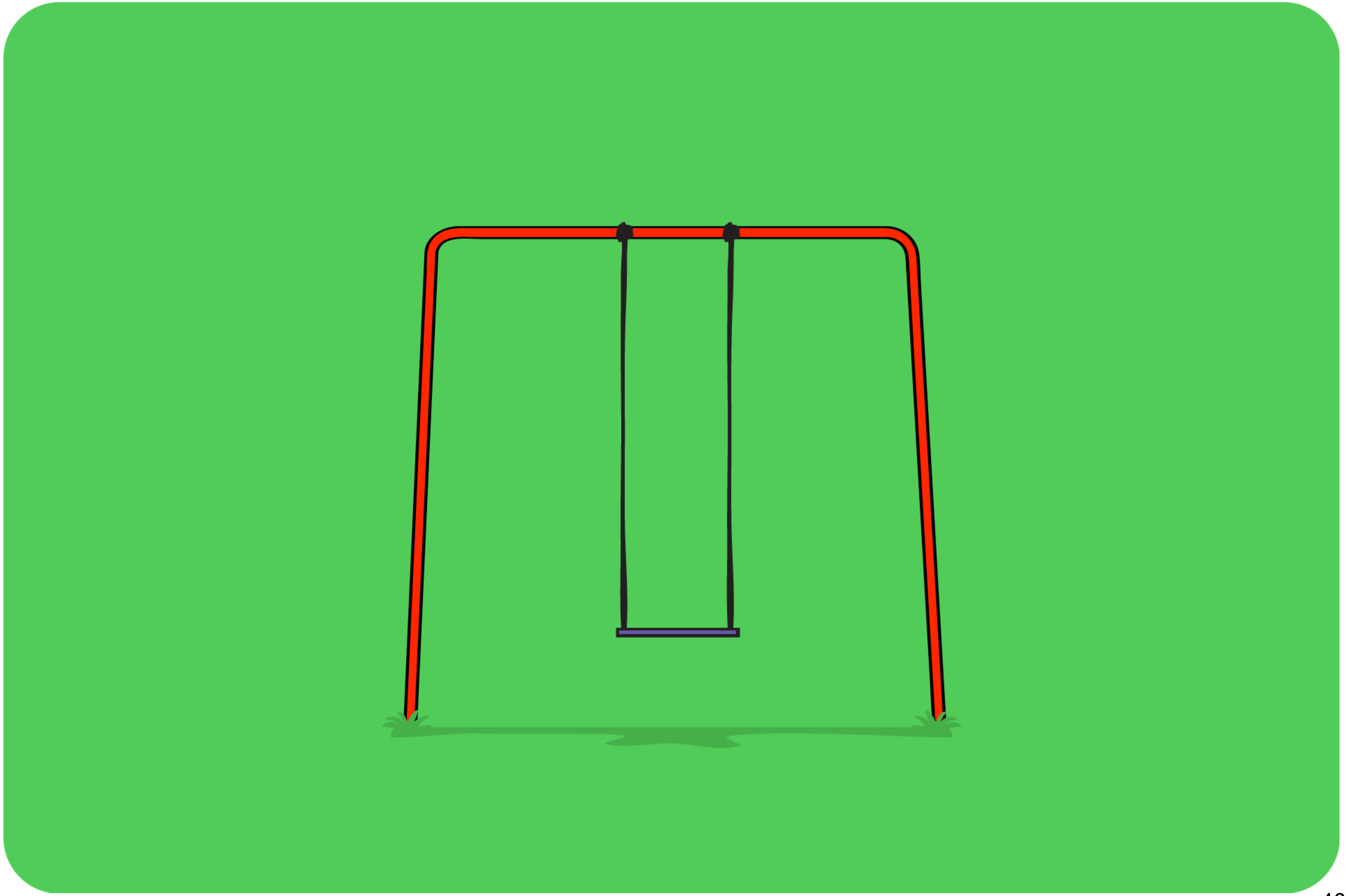

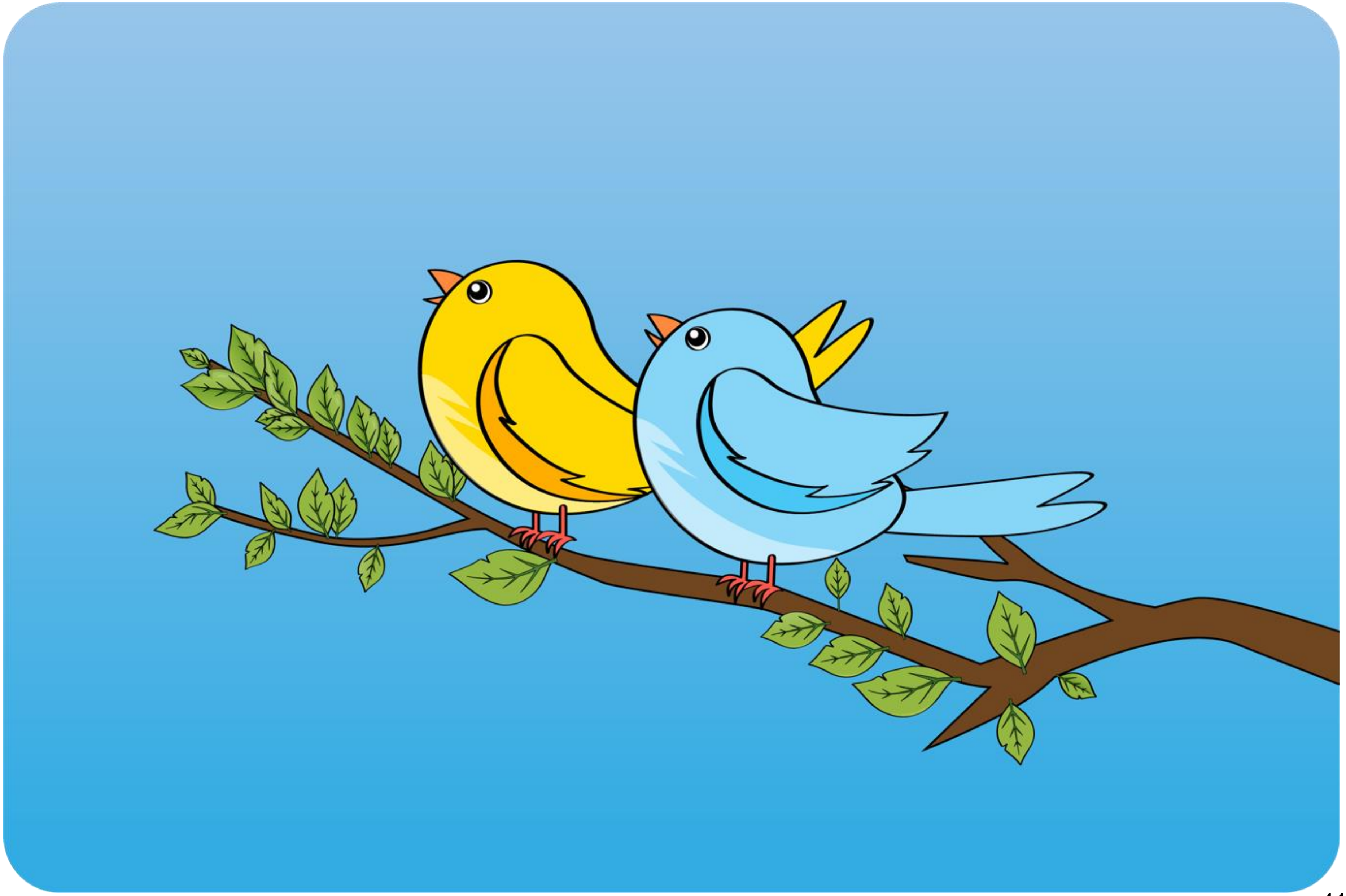

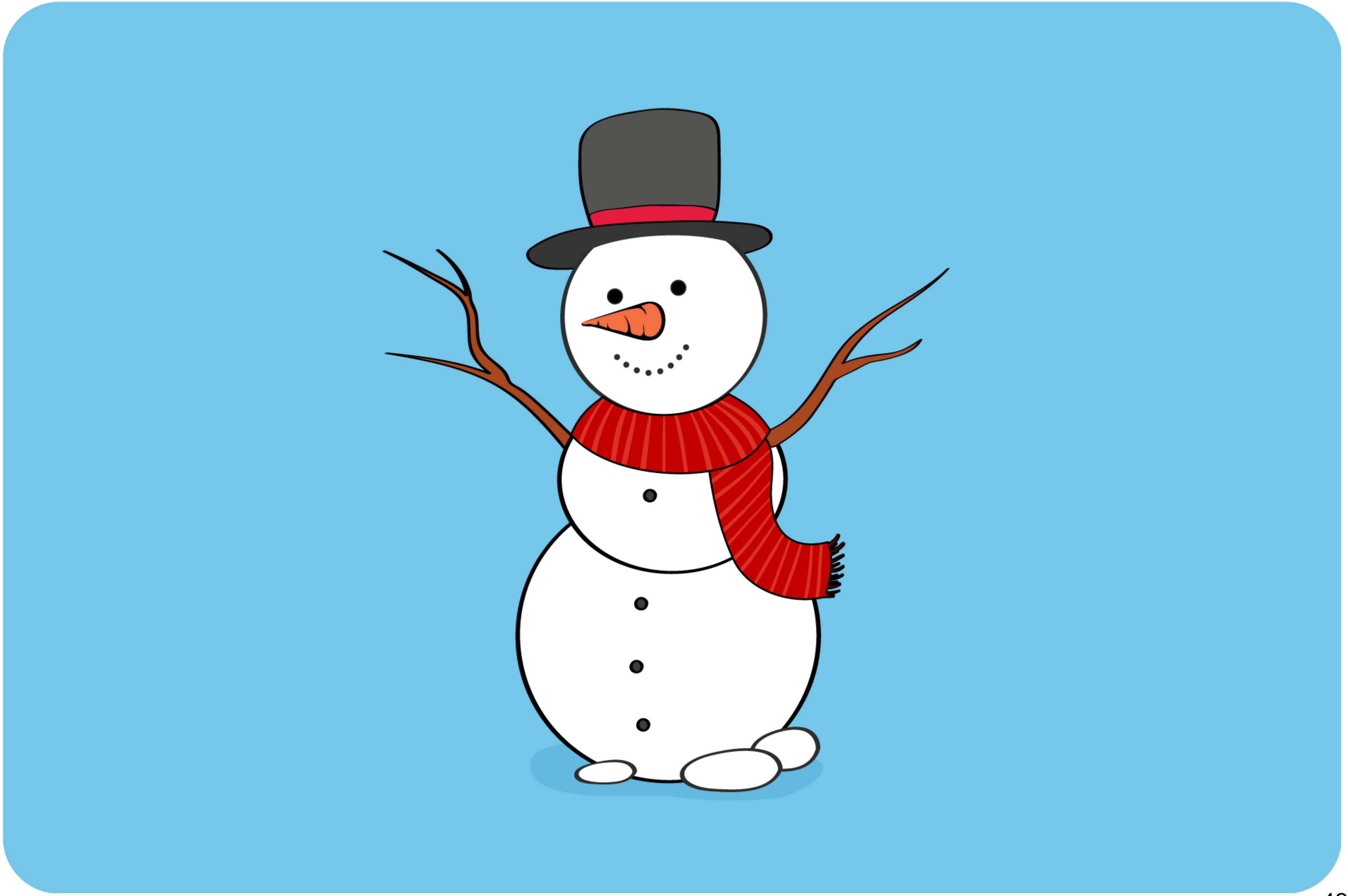

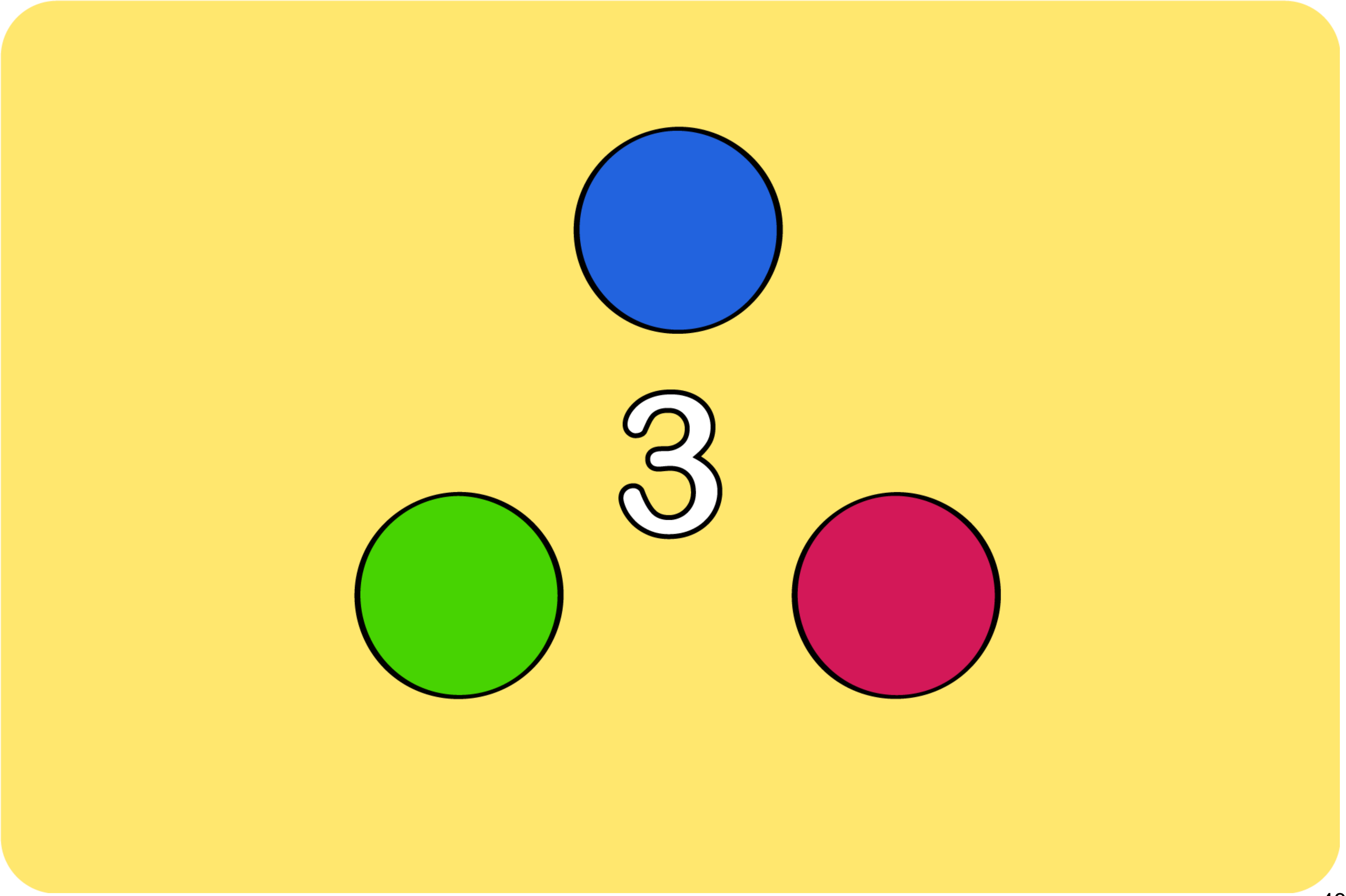
3

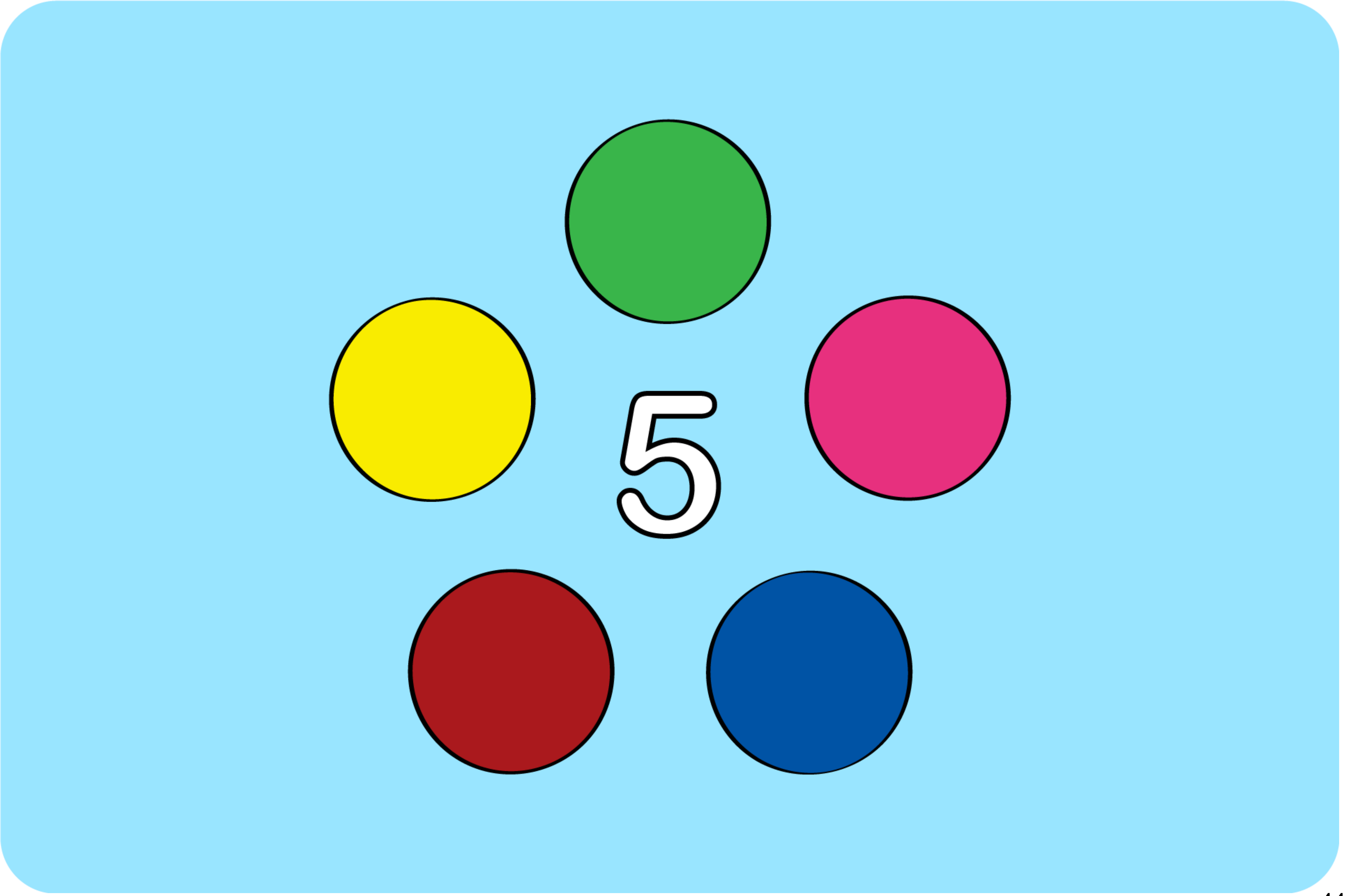
5

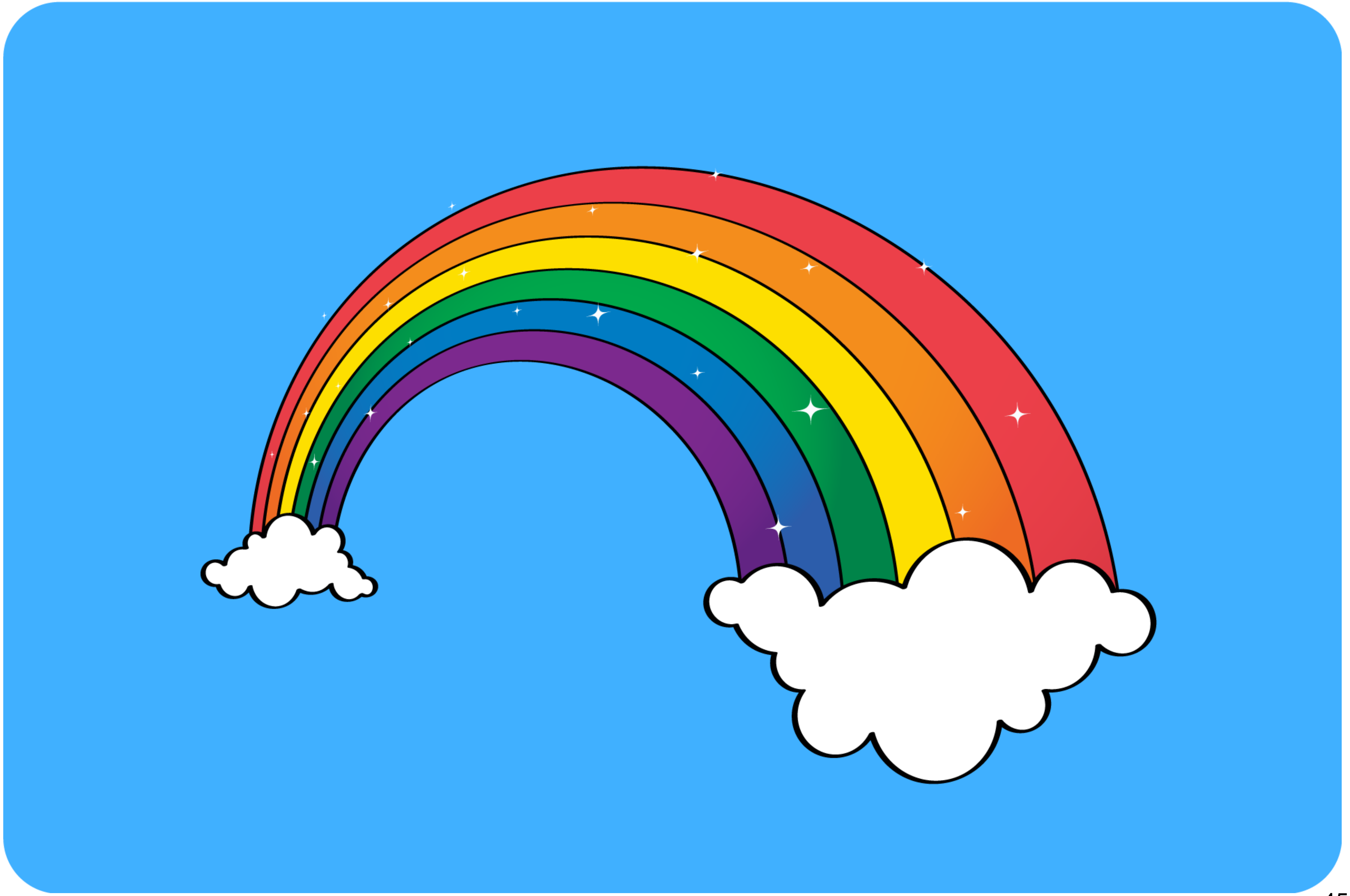

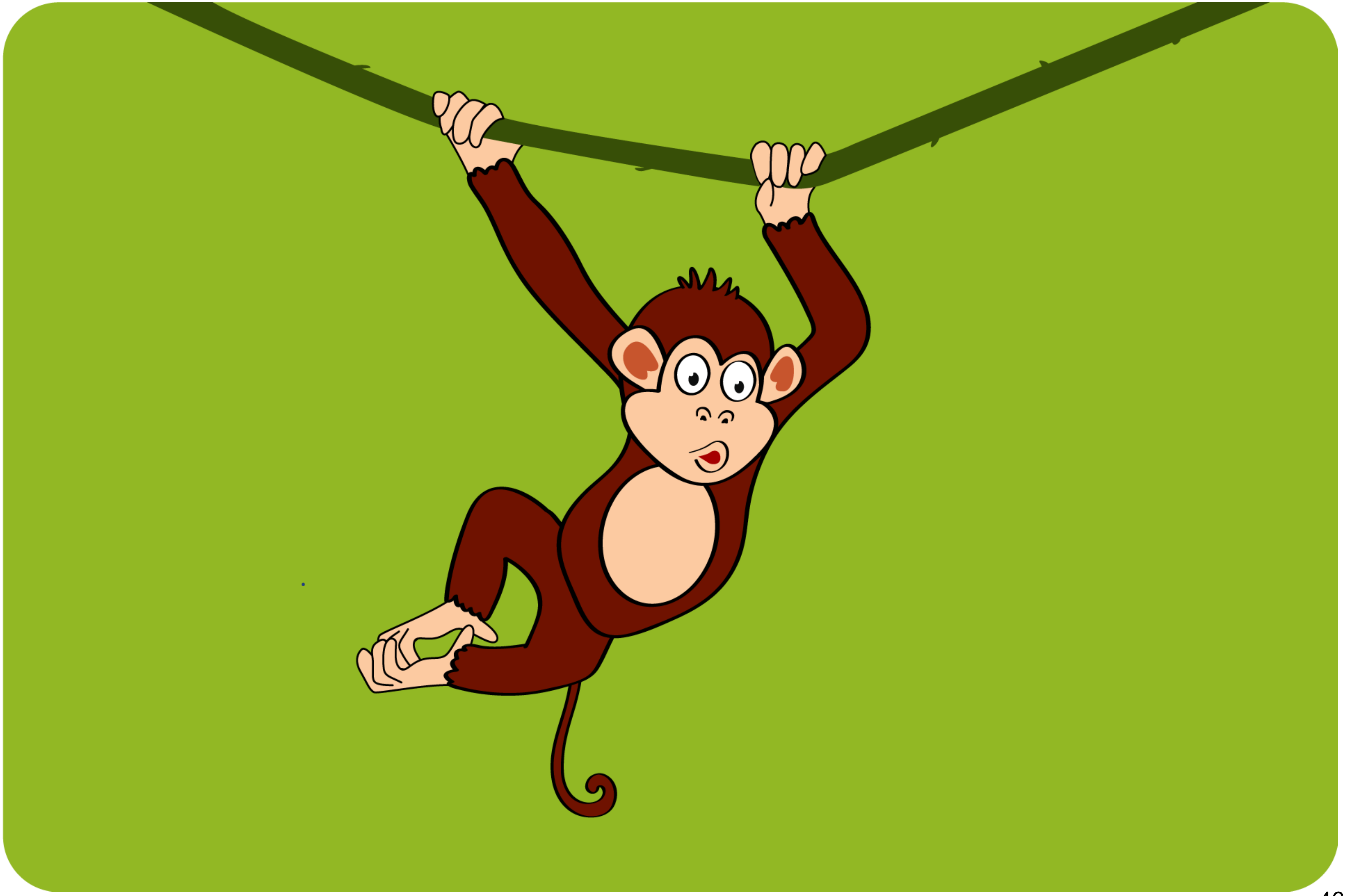

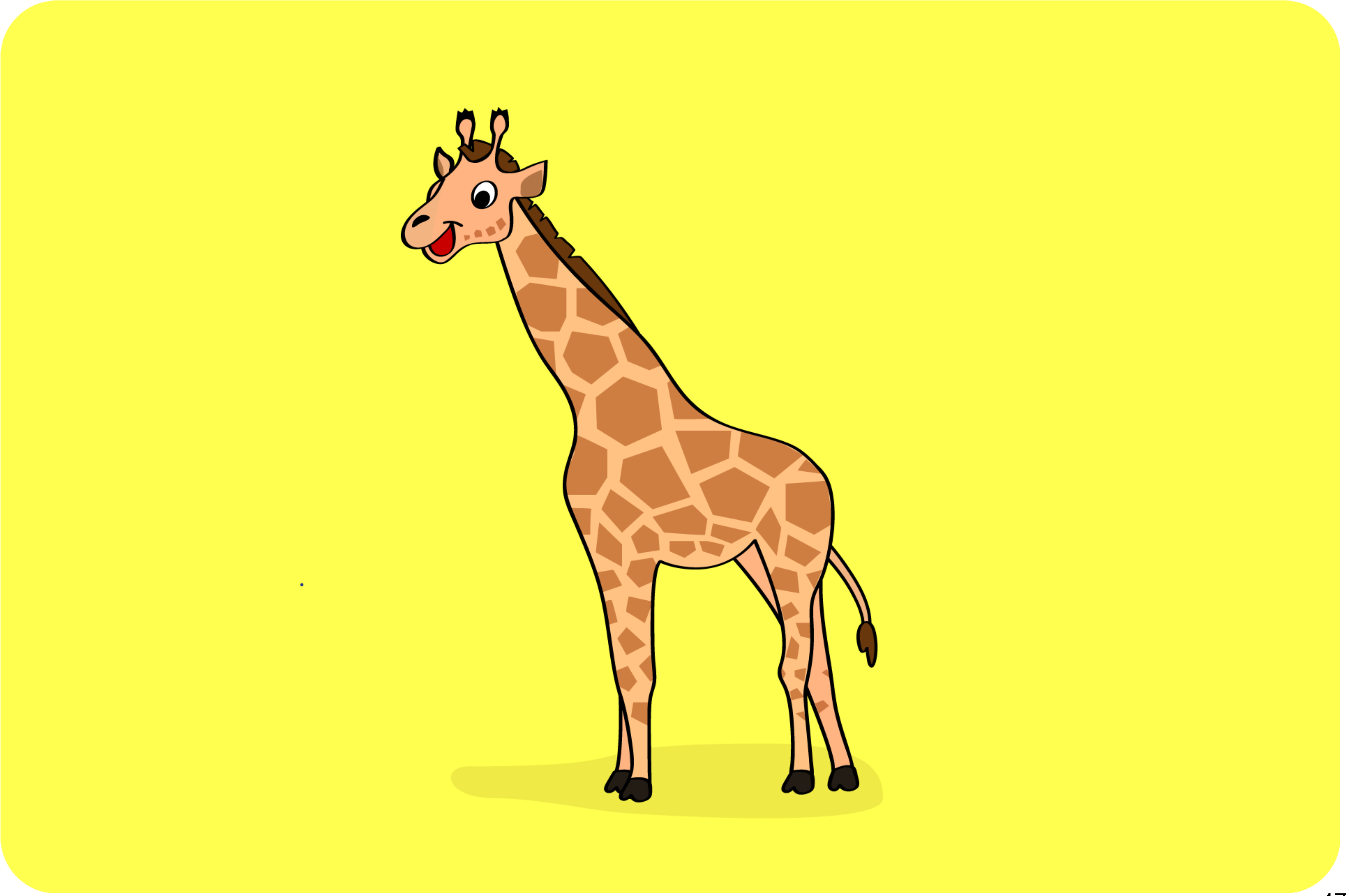

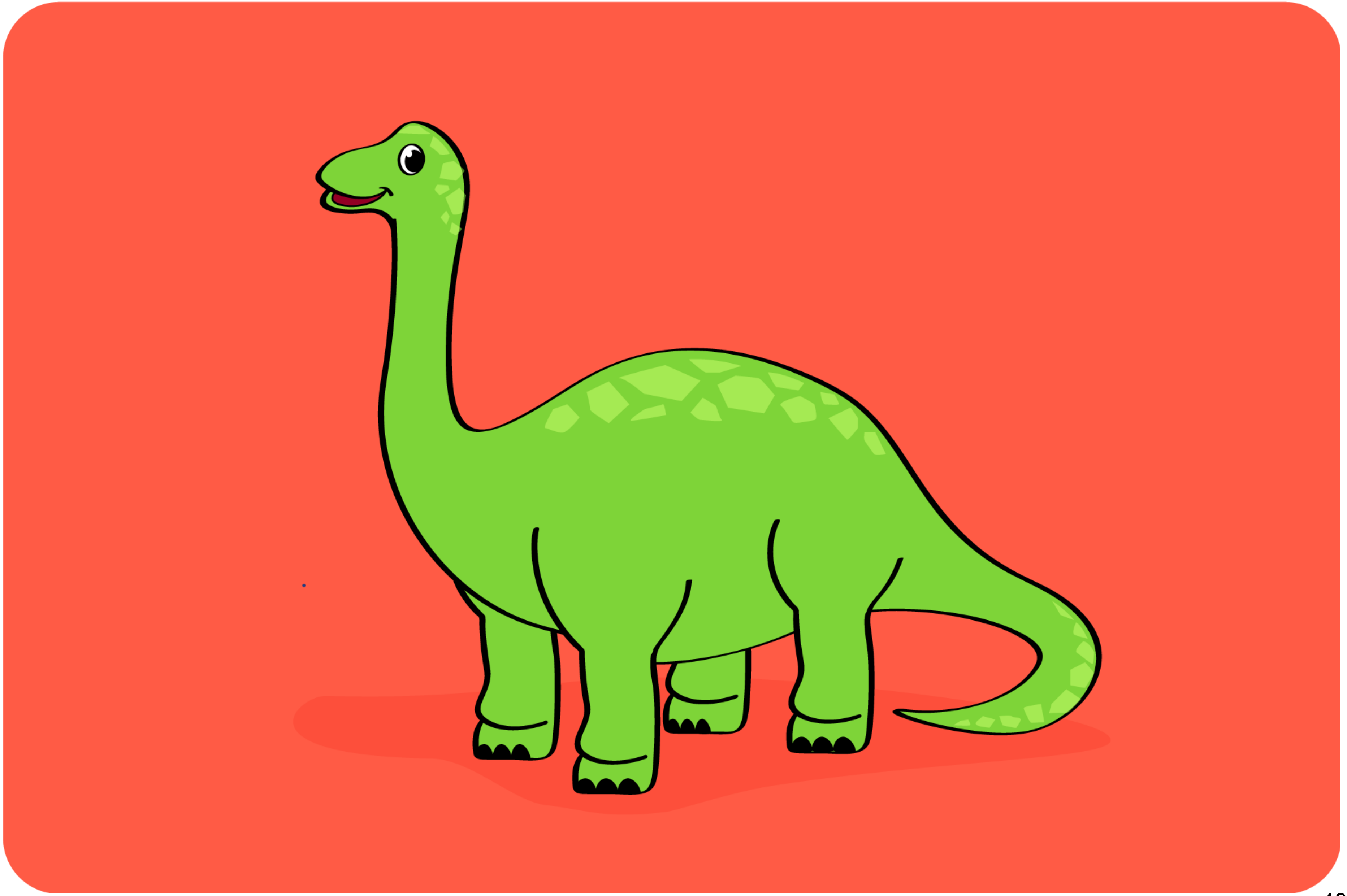

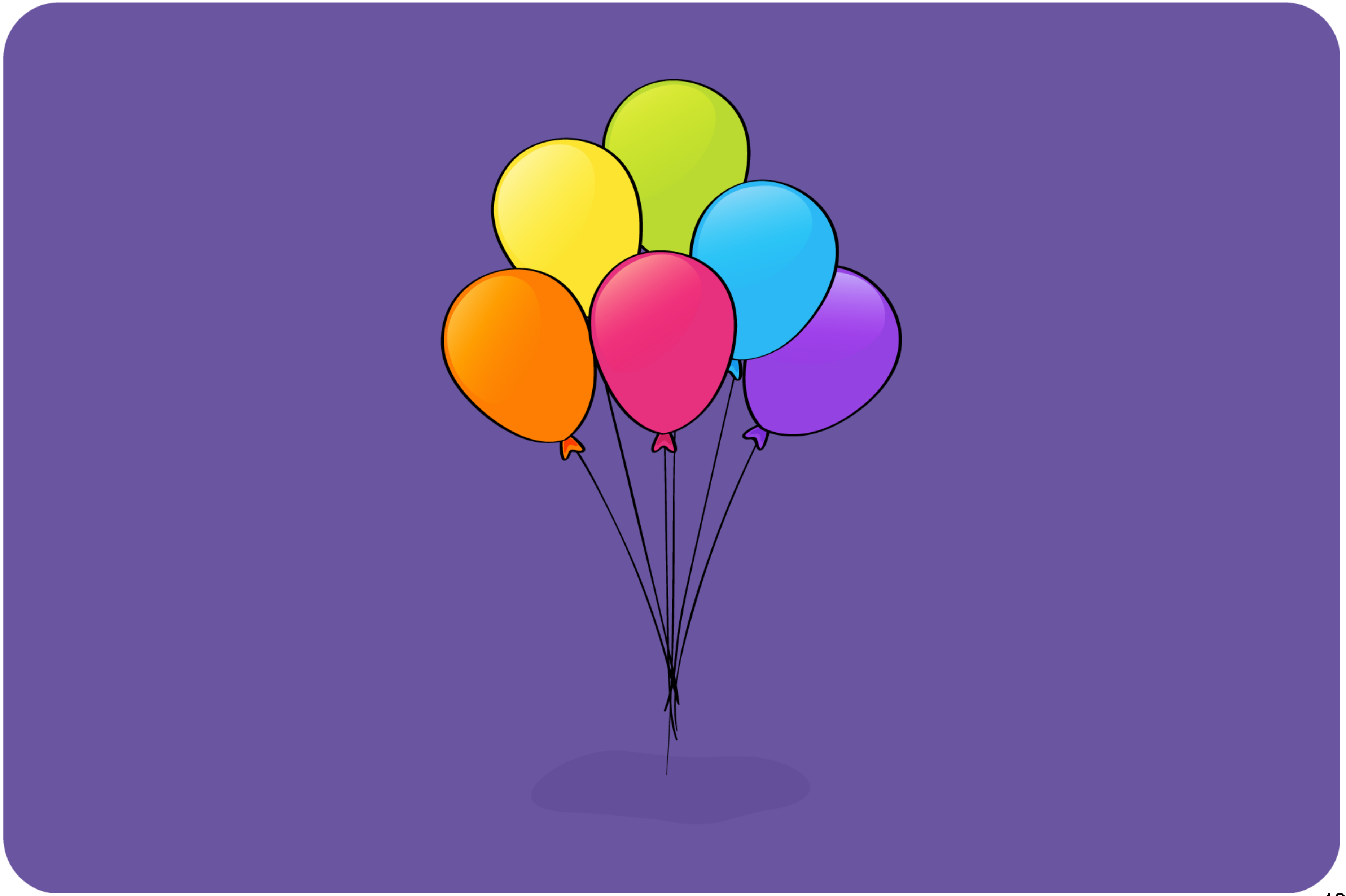

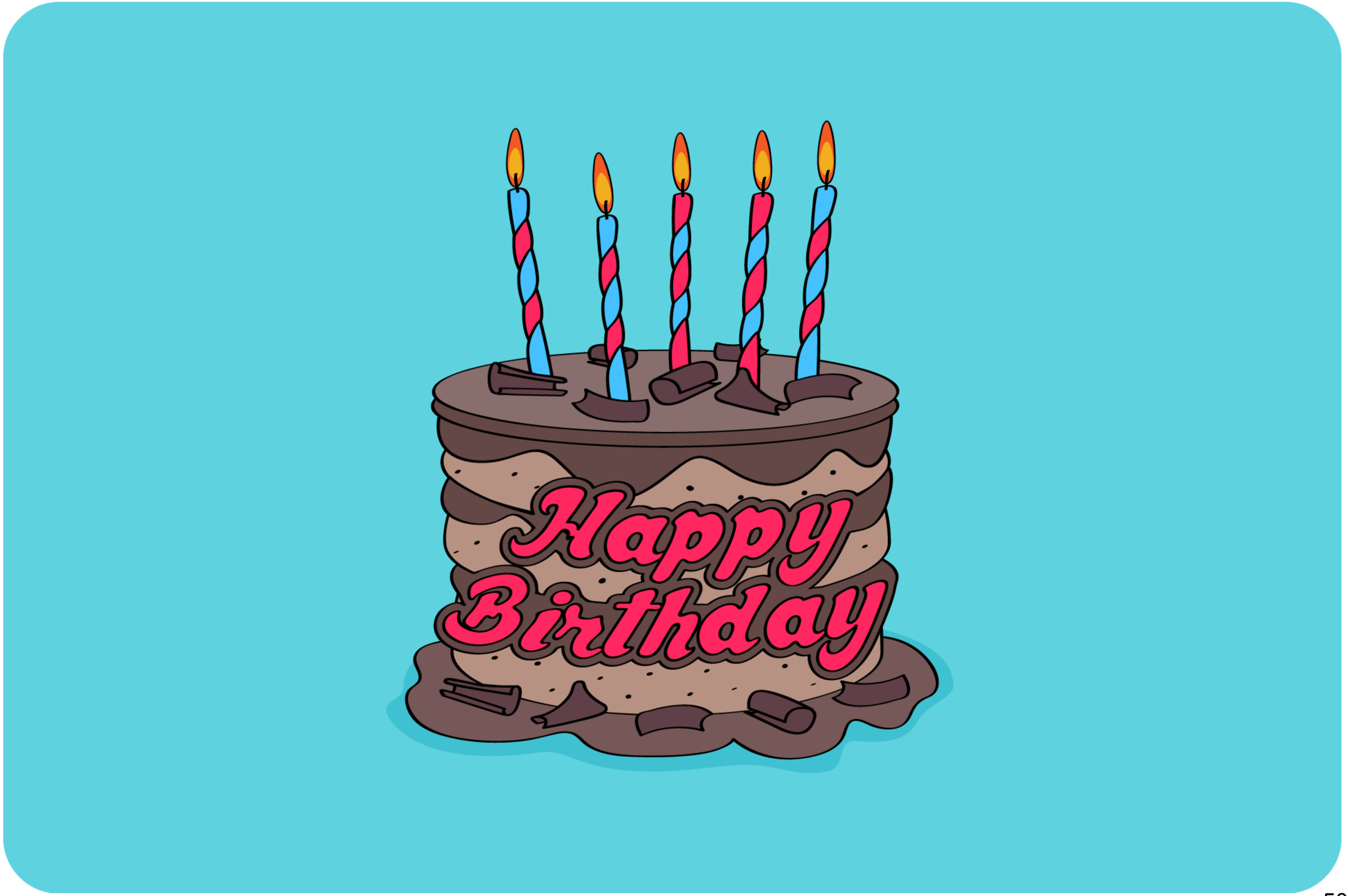
Happy Birthday

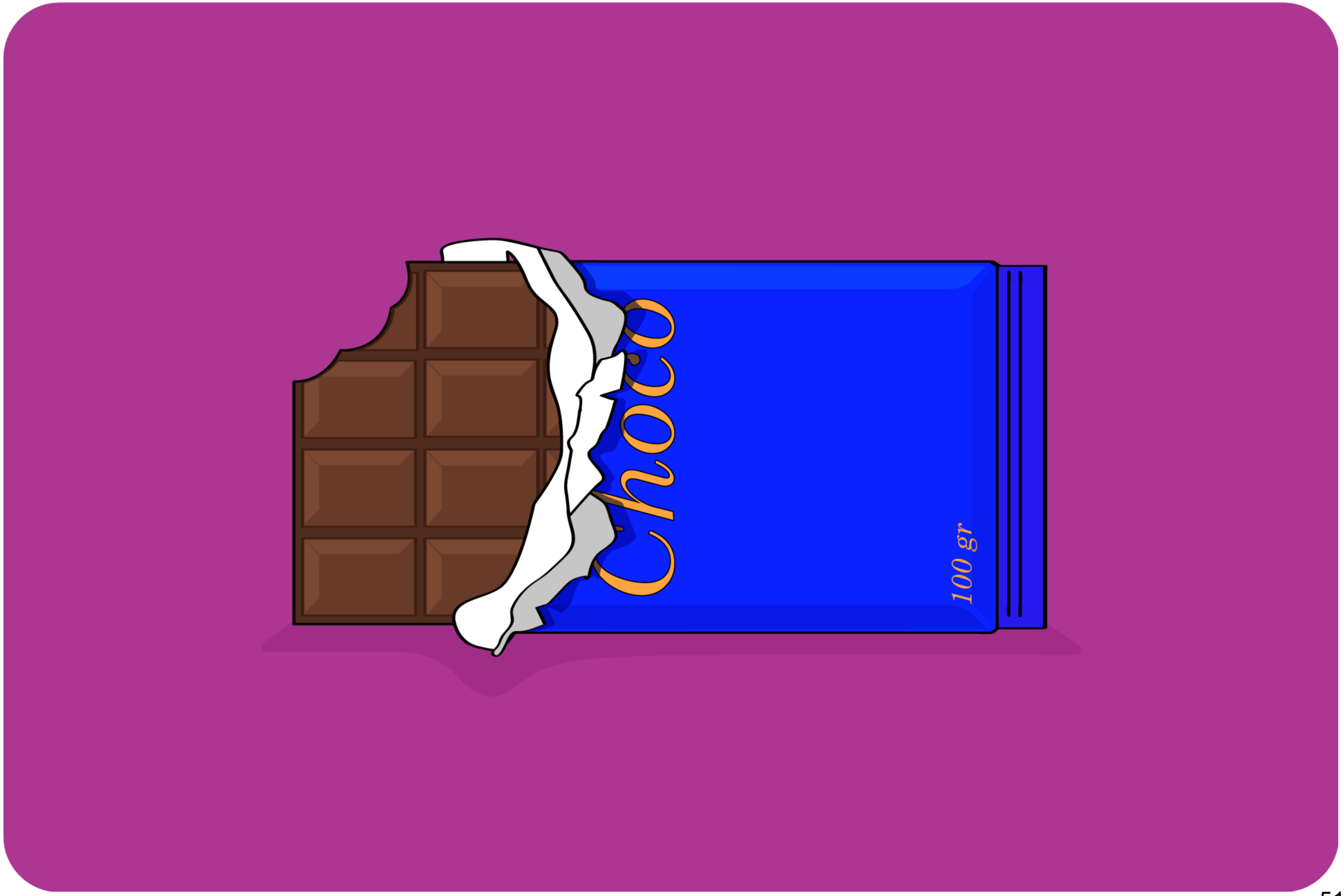
Choco
100 gr

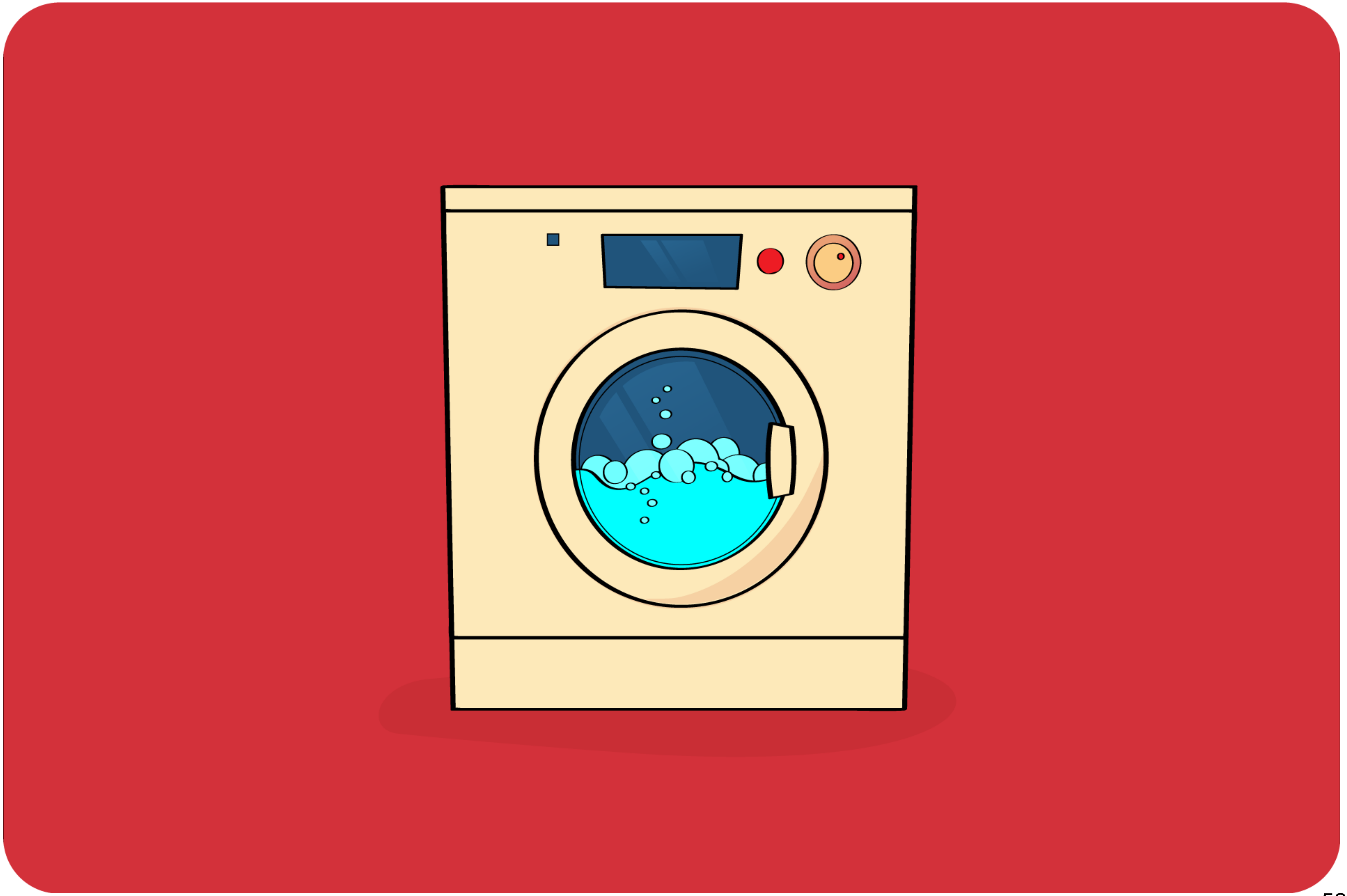

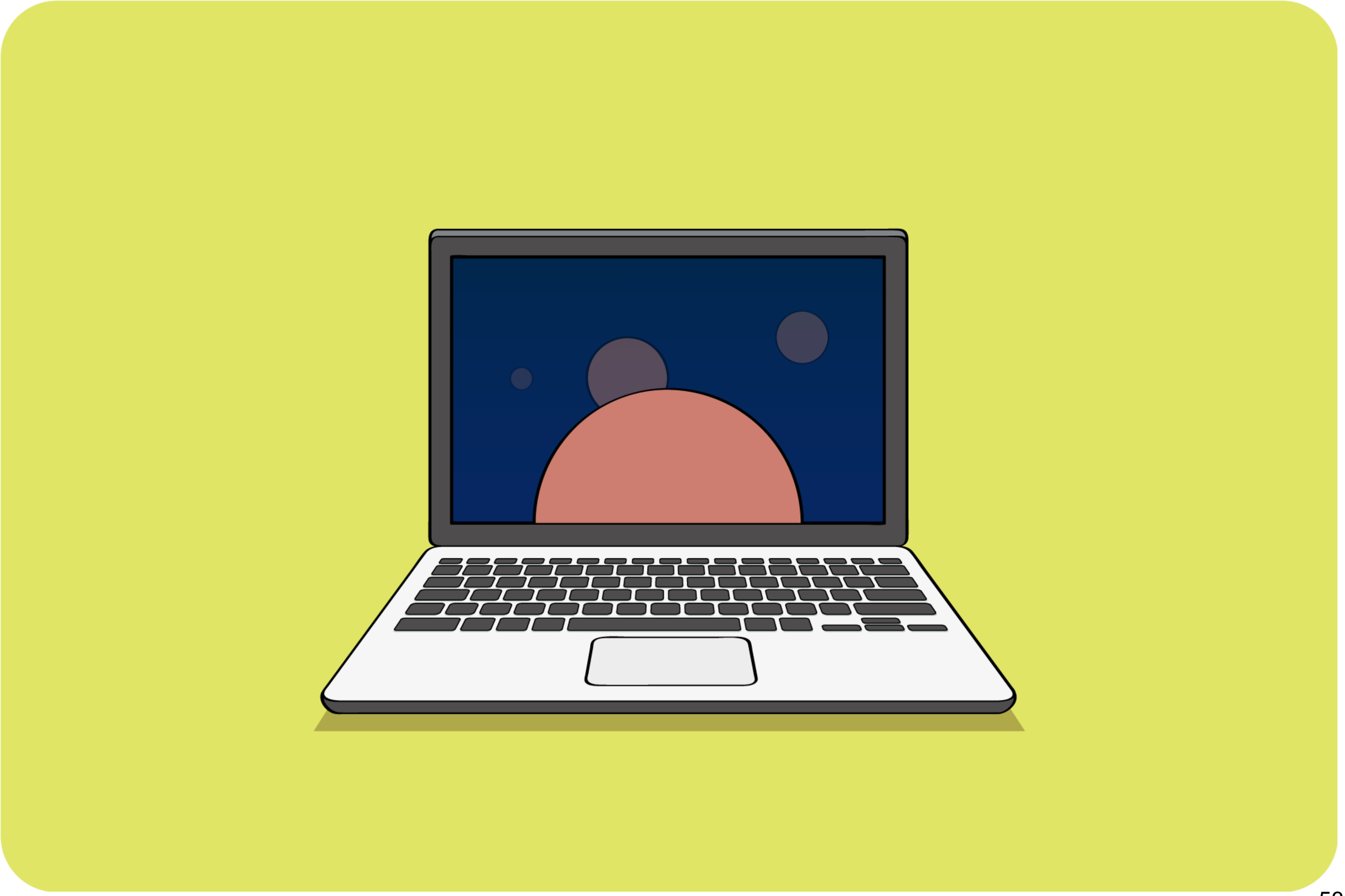

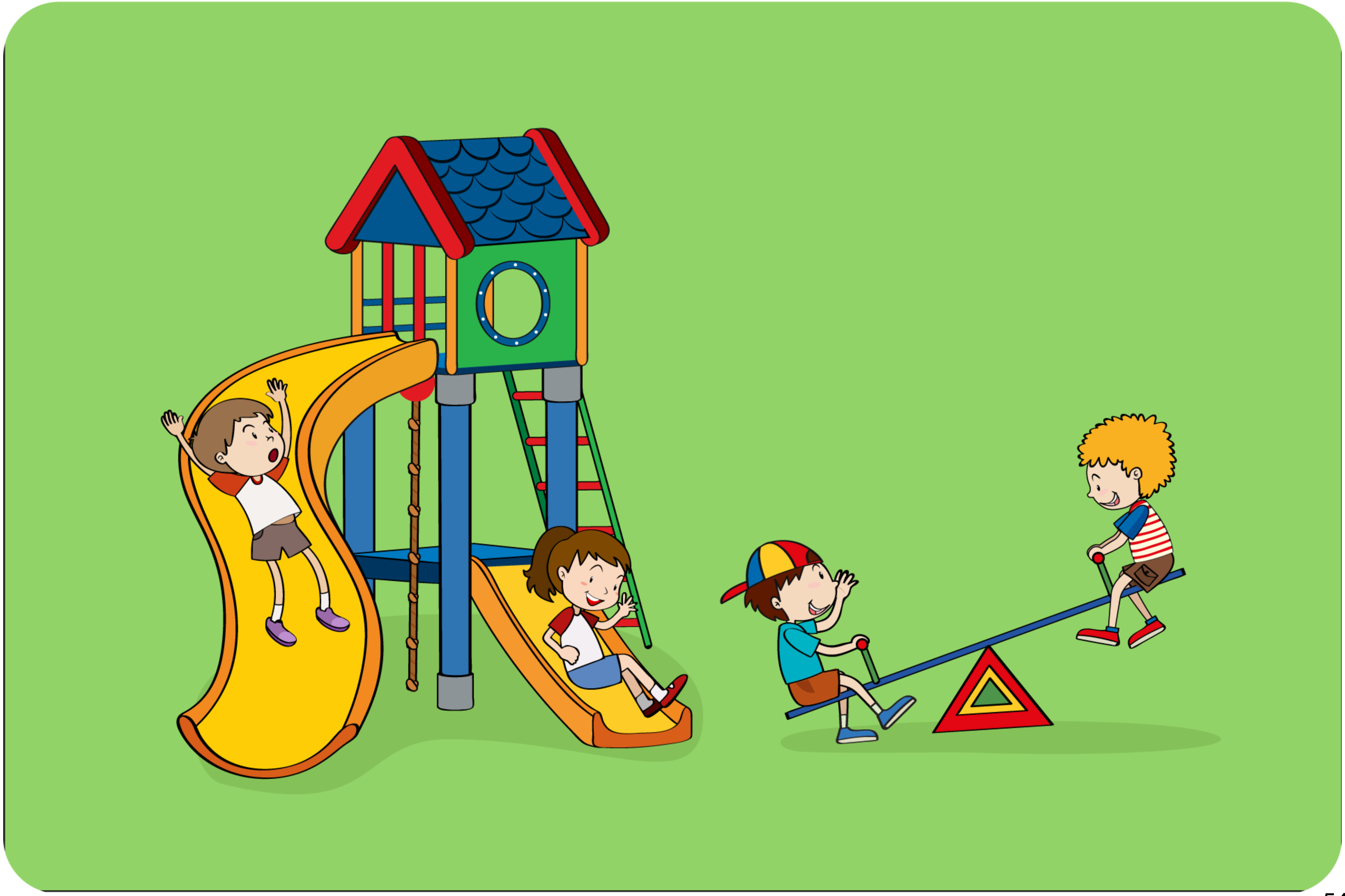

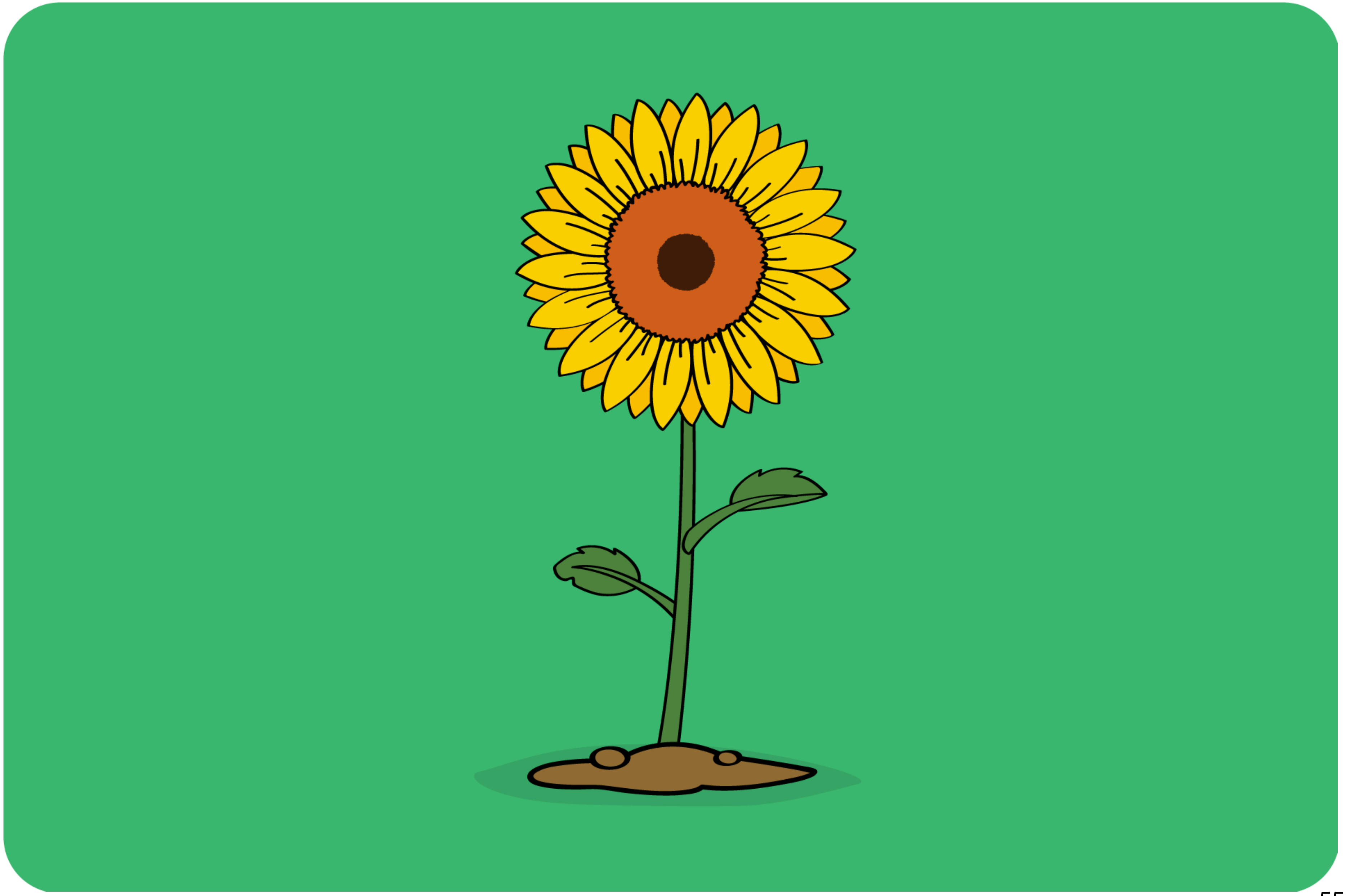

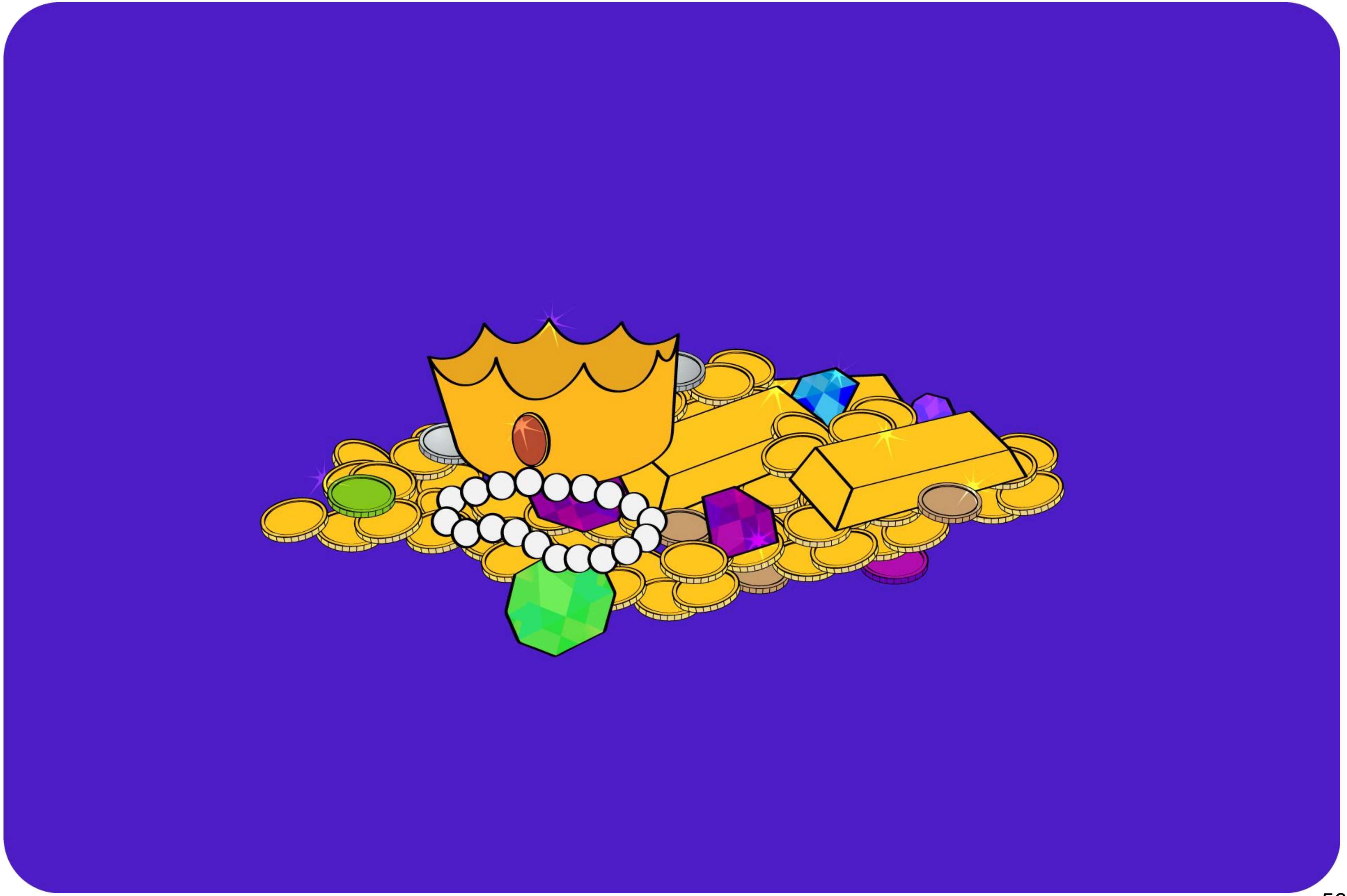

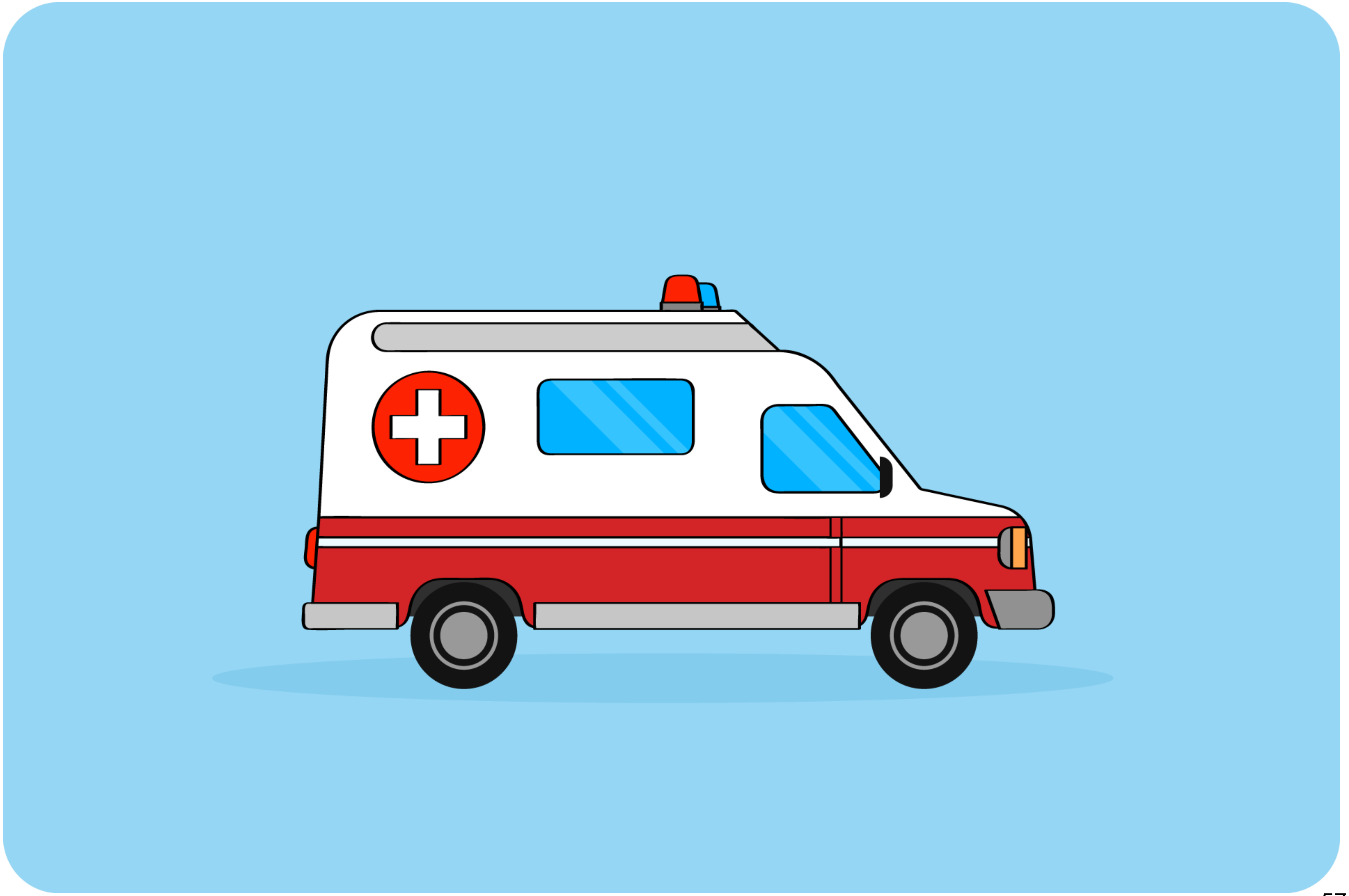

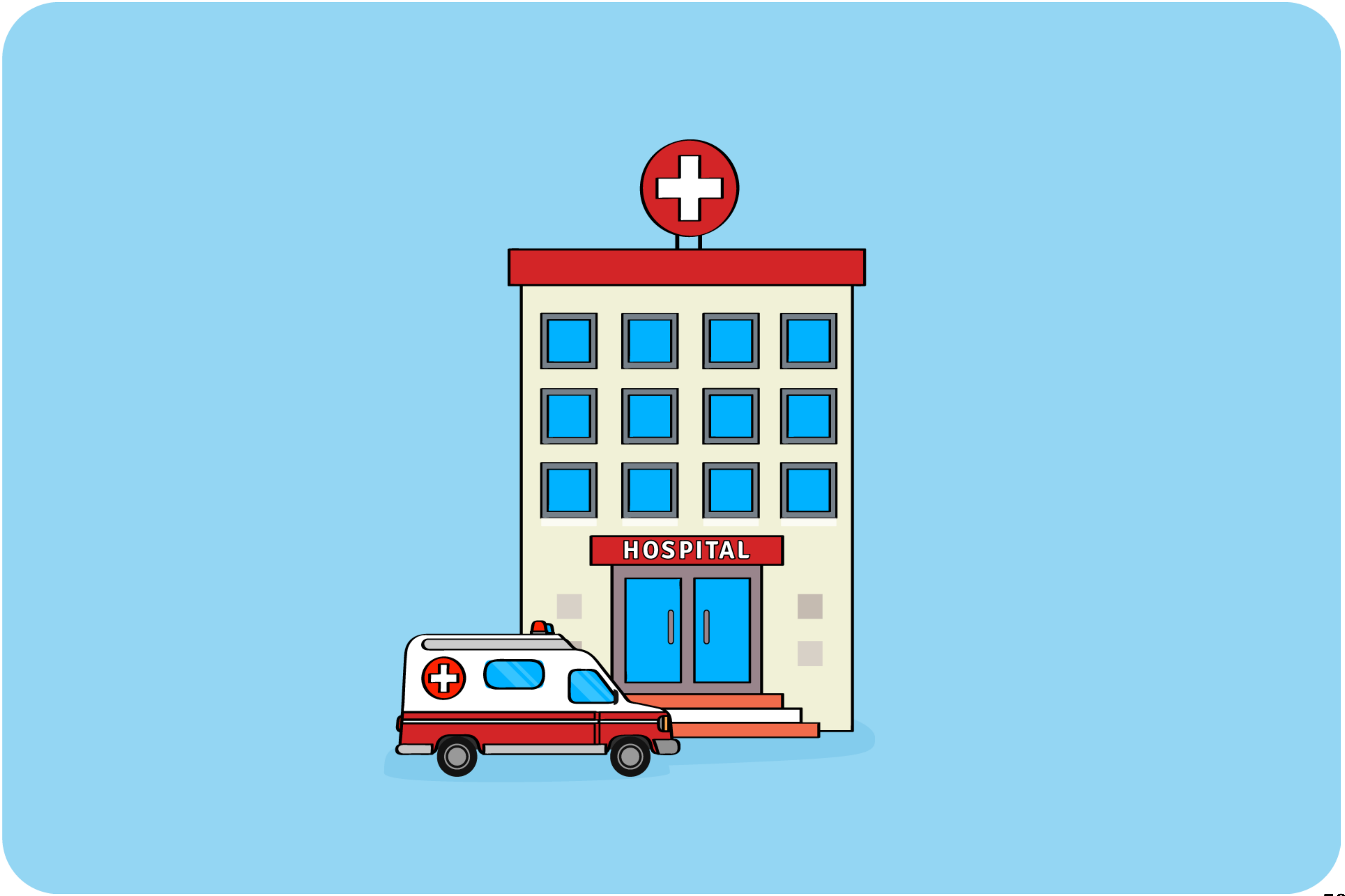
HOSPITAL

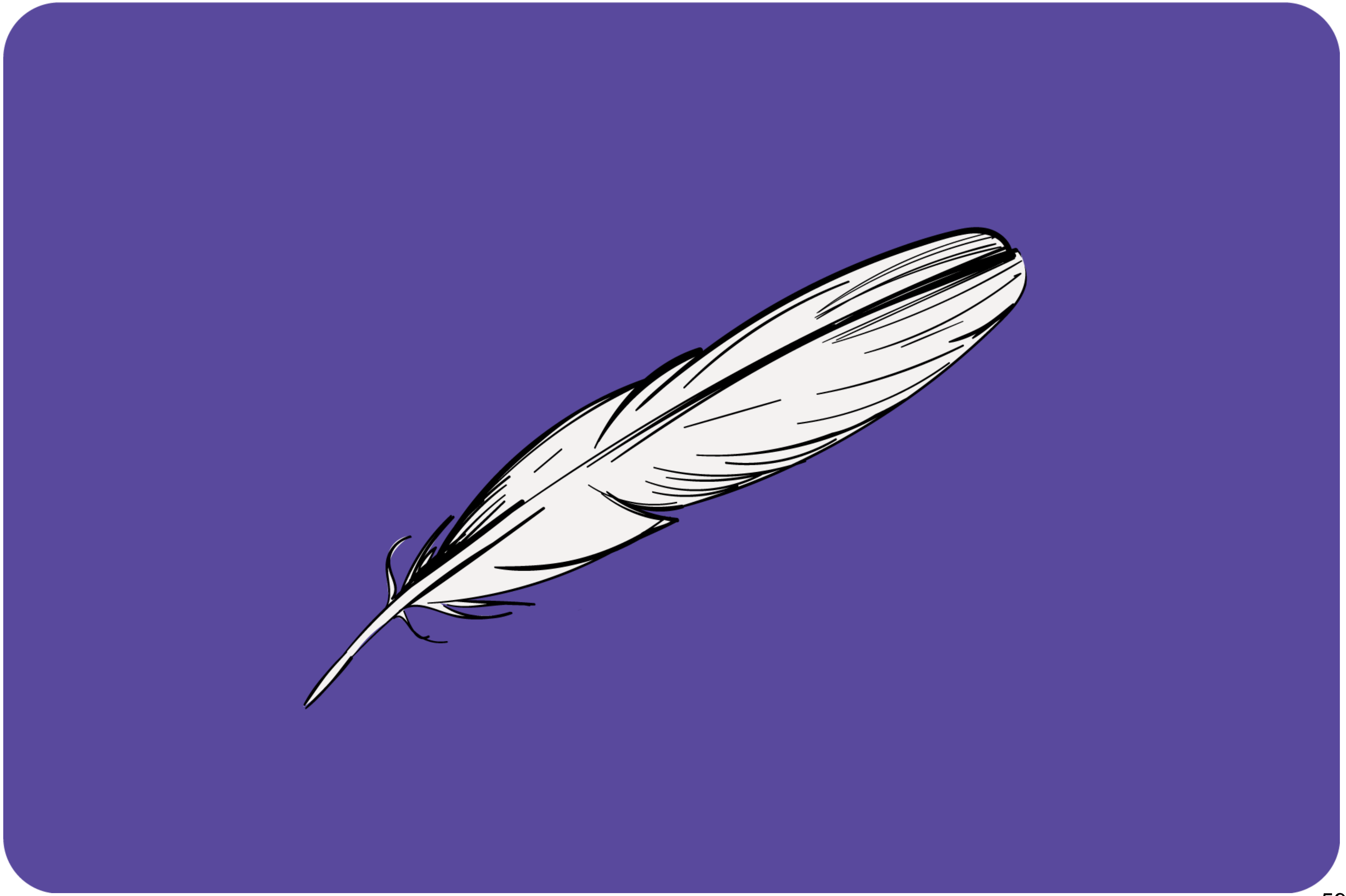

All International Phonetic Alphabet (IPA) symbols below are denoted using the Harrington/Cox/Evans (HCE) system.

Consonant inventory

Table S1. Frequency and distribution of consonants in the 55 picture stimuli.

Consonant	Syllable Initial, Word Initial	Syllable Initial, Within Word	Syllable Final, Word Final	Syllable Final, Within Word
**p**	1 /peŋgwən/	1 /ɔktəpʊs/	1 /ʃiːp/	1 /heliːkɔptə/
**b**	2 /bəlʉːnz/, /bɜːθdæɪkæɪk/	3 /lɐnʧbɔks/, /ɹæɪnbəʉ/, /jeləʉː bɜːd/	0	0
**t**	2 /toɪlət/, /tiːθ/	4 /heliːkɔptə/, /ɔktəpʊs/, /kəmpjʉːtə/, /hɔspɪtəl/	2 /toɪlət/, /ʧɔklət/	0
**d**	1 /dɑenəsoː/	1 /bɜːθdæɪkæɪk/	1 /jeləʉː bɜːd/	0
**k**	2 /kæŋgəɹʉː/, /kəmpjʉːtə/	4 /heliːkɔptə/, /spɹɪŋkəlz/, /mɐŋkiː/, /bɜːθdæɪkæɪk/	3 /stɪk/, /blæk/, /bɜːθdæɪkæɪk/	2 /ɔktəpʊs/, /ʧɔklət/
**g**	1 /gəʉ/	1 /kæŋgəɹʉː/	1 /fɹɔg/	0
**m**	2 /mɪlk/, /mɐŋkiː/	2 /snəʉmæn/, /wɔʃɪŋməʃiːn/	1 /bɹʉːm/	3 /vækjʉːmkliːnə/, /kəmpjʉːtə/, /æmbjələns/
**n**	0	2 /vækjʉːmkliːnə/, /dɑenəsoː/	4 /tɹæɪn/, /peŋgwən/, /snəʉmæn/, /wɔʃɪŋməʃiːn/	2 /ɹæɪnbəʉ/, /sɐnflæɔːə/
**ŋ**	-	-	1 /swɪŋ/	5 /peŋgwən/ /kæŋgəɹʉː/, /spɹɪŋkəlz/, /mɐŋkiː/, /wɔʃɪŋməʃiːn/
**s**	1 /sɐnflæɔːə/	1 /dɑenəsoː/	1 /ɔktəpʊs/	0
**z**	2 /zʉː/, /zebɹə/	1 /pɹezənts/	5 /steːz/, /ʃʉːz/, /ɪəz/, /stɹoːbɹiːz/, /ʧiːz/	0
**f**	2 /fɑev/, /feðə/	1 /eləfənt/	2 /liːf/, /ʤəɹeːf/	0
**v**	1 /vækjʉːmkliːnə/	1 /skɹʉːdɹɑevə/	1 /fɑev/	0

**θ**	0	0	1 /ti:θ/	1 /bɜ:θdæɪkæɪk/
**ð**	0	1 /feðə/	0	0
**ʃ**	2 /ʃʉ:z/, /ʃi:p/	2 /wɔʃɪŋməʃi:n/ (2)	1 /splæʃ/	0
**ʒ**	-	1 (tɹeʒə)	0	0
**tʃ**	2 /ʧi:z/, /ʧɔklət/	0	1 /wɪʧ/	1 /lenʧbɔks/
**dʒ**	1 /ʤəɹɐ:f/	0	1 (fɹɪʤ)	0
**h**	3 /he:/, /heli:kɔptə/, /hɔspɪtəl/	0	-	-
**w**	2 /wɪʧ/, /wɔʃɪŋməʃi:n/	0	-	-
**l**	2 /lenʧbɔks/, /li:f/	7 /toɪlət/, /heli:kɔptə/, /eləfənt/, /jeləʉ: bɜ:d/, /bəlʉ:nz/, /ʧɔklət/, /æmbjələns/	1 /hɔspɪtəl/	0
**j**	1 /jeləʉ: bɜ:d/	0	-	-
**ɹ**	1 /ɹæɪnbəʉ/	3 /ɔɹænʤ/, /kæŋgəɹʉ:/ /ʤəɹɐ:f/	-	-

Consonant cluster inventory

Table S2. Frequency and distribution of two- and three-consonant clusters in the 55 picture stimuli.

Syllable Initial, Word Initial	Frequency	Syllable Initial Within Word	Frequency	Syllable Final, Word Final	Frequency	Syllable Final, Within Word	Frequency
sn	1 /snəʉmæn/	sp	1 /hɔspɪtəl/	dz	1 /klæɔdz/	ntʃ	1 /lenʧbɔks/
sk	1 /skɑe/	br	2 /zebɹə/, /stɹo:bɹi:z/	ks	1 /lenʧbɔks/		
st	2 /stɐ:z/, /stɪk/	bj	1 /æmbjələns/	lk	1 /mɪlk/		

sw	1 /swɪŋ/	gr	1 /plæɪgɹæɔnd/	lz	1 /spɹɪŋkəlz/		
kl	1 /klæɔdz/	gw	1 /peŋgwən/	nd	1 /plæɪgɹæɔ nd/		
θr	1 /θɹiː/	kj	1 /vækjʉːmkliːnə/	ns	1 /æmbjələn s/		
br	1 /bɹʉːm/	kl	1 /vækjʉːmkliːnə/	nt	1 /eləfənt/		
bl	1 /blæk/	dr	1 /skɹʉːdɹɑevə/	ndʒ	1 /ɔɹændʒ/		
pr	1 /pɹezənts/	fl	1 /sɐnflæɔːə/	nz	1 /bəlʉːnz/		
pl	1 /plæɪgɹæɔnd/	pj	1 /kəmpjʉːtə/	nts	1 /pɹezənts/		
tr	2 /tɹæɪn/, /tɹeʒə/						
fr	2 /fɹɔg/, /fɹɪdʒ/						
skr	1 /skɹʉːdɹɑevə/						
spl	1 /splæʃ/						
spr	1 /spɹɪŋkəlz/						
str	1 /stɹoːbɹiːz/						

Vowel inventory

Table S3. Frequency and distribution of vowels of Australian English (transcribed in Harrington/Cox/Evans (HCE) system) in the 55 picture stimuli.

**Vowel**	**Frequency**	**Stimuli**	**Vowel**	**Frequency**	**Stimuli**

iː	**10**	/tiːθ/, /heliːkɔptə/, /ʃiːp/, /vækjʉːmkliːnə/, /stɹoːbɹiːz/, /ʧiːz/, /liːf/, /θɹiː/, /mɐŋkiː/ /wɔʃɪŋməʃiːn/	ɜː	**2**	/jeləʉː bɜːd/, /bɜːθdæɪkæɪk/
ɪ	**8**	/stɪk/, /wɪʧ/, /spɹɪŋkəlz/, /mɪlk/, /fɹɪʤ/, /swɪŋ/, /wɔʃɪŋməʃiːn/, /hɔspɪtəl/	ə	**23**	/zebɹə/, /peŋgwən/, /heliːkɔptə/, /ɔktəpʊs/, /kæŋgəɹʉː/, /eləfənt/ (2), /spɹɪŋkəlz/, /skɹʉːdɹɑevə/, /pɹezənts/, /ʤəɹɐːf/, /dɑenəsoː/, /bəlʉːnz/, /ʧɔklət/, /wɔʃɪŋməʃiːn/, /kəmpjʉːtə/ (2), /sɐnflæɔːə/, /tɹeʒə/, /æmbjələns/ (2), /hɔspɪtəl/, /feðə/
e	**9**	/zebɹə/, /peŋgwən/, /heliːkɔptə/, /vækjʉːmkliːnə/, /eləfənt/, /pɹezənts/, /jeləʉː bɜːd/, /tɹeʒə/, /feðə/	æɪ	**5**	/tɹæɪn/, /ɹæɪnbəʉ/, /bɜːθdæɪkæɪk/ (2), /plæɪgɹæɔnd/
æ	**7**	/blæk/, /ɔɹænʤ/, /vækjʉːmkliːnə/, /kæŋgəɹʉː/, /splæʃ/, /snəʉmæn/, /æmbjələns/	ɑe	**4**	/skɑe/, /skɹʉːdɹɑevə/, /fɑev/, /dɑenəsoː/
ɐː	**2**	/stɐːz/, /ʤəɹɐːf/	oɪ	**1**	/toɪlət/
ɐ	**3**	/lɐnʧbɔks/, /mɐŋkiː/, /sɐnflæɔːə/	æɔ	**3**	/klæɔdz/, /plæɪgɹæɔnd/, /sɐnflæɔːə/
ɔ	**8**	/ɔɹænʤ/, /heliːkɔptə/, /lɐnʧbɔks/, /ɔktəpʊs/, /fɹɔg/, /ʧɔklət/, /wɔʃɪŋməʃiːn/, /hɔspɪtəl/	əʉ	**4**	/gəʉ/, /jeləʉ bɜːd/, /snəʉmæn/, /ɹæɪnbəʉ/
oː	**2**	/stɹoːbɹiːz/, /dɑenəsoː/	ɪə	**1**	/ɪəz/
ʊ	**1**	/ɔktəpʊs/	eː	**1**	/heː/
ʉː	**8**	/bɹʉːm/, /ʃʉːz/, /zʉː/, /vækjʉːmkliːnə/, /kæŋgəɹʉː/, /skɹʉːdɹɑevə/, /bəlʉːnz/, /kəmpjʉːtə/	ʊə	**0**	

Multisyllabic words and syllable shapes

Table S4. Frequency of monosyllabic and multisyllabic words in the 55 picture stimuli.

Word type	Frequency	Stimuli
1 syllable	24	Sky, stars, broom, stick, black, shoes, ears, teeth, hair, train, zoo, go, clouds, witch, sheep, frog, splash, cheese, milk, fridge, leaf, swing, three, five,
2 syllable	16*	Toilet, orange, zebra, penguin, lunchbox, sprinkles, strawberries*, presents, snowman, rainbow, monkey, giraffe, balloons, chocolate*, playground, treasure, feather
3 syllable	13*	Octopus, kangaroo, elephant, strawberries*, screwdriver, yellow bird, dinosaur, birthday cake, chocolate*, computer, sunflower, ambulance, hospital,
4 syllable	3	Helicopter, vacuum cleaner, washing machine

*words appear in both 2- and 3-syllable count

Table S5. Frequency and distribution of syllable shapes in the 55 picture stimuli.

Syllable shape	Total frequency	Stimuli			
		**1 syllable words**	**2 syllable words**	**3 syllable words**	**4 syllable words**
V			**ɔ-**ɹændʒ	el-**ə-**fənt, sɐn-flæɔː**-ə, **ʧɔk-**ə-**lət###	
CV		gəʉ, **heː, zʉː**	**toɪ**-lət,**ze**-bɹə, ɹæɪn-**bəʉ,** meŋ-**kiː, ʤə**-ɹɐːf, **bə**-lʉːnz, tɹe-**ʒə, fe-ðə** (2)	ɔk-**tə**-pʊs, kæŋ-**gə-ɹʉː** (2), skɹʉː-dɹɑe-**və, je-ləʉː** bɜːd (2), **dɑe-nə-soː** (3), bɜːθ-**dæɪ-**kæɪk, kəm-pjʉː**-tə,** hɔs-**pɪ**-təl, stɹoː**-be-**ɹiːz***	**he-liː**-kɔp-**tə** (3), væk-jʉːm-kliː-**nə, wɔ**-ʃɪŋ-**mə**-ʃiːn (2)
VC		**ɪəz**		**ɔk**-tə-pʊs, **el**-ə-fənt, **æm**-bjə-ləns	
CVC		**ʃʉːz, tiːθ, wɪʧ, ʃiːp, ʧiːz, liːf, fɑev,**	toɪ-**lət, peŋ**-gwən, snəʉ-**mæn, ɹæɪn**-bəʉ, **mɐŋ**-kiː, ʤəɹ**ɐːf, ʧɔk-lət**## (2)	ɔk-tə-**pʊs, kæŋ**-gə-ɹʉː, je-ləʉː **bɜːd, bɜːθ**-dæɪ-**kæɪk** (2), **kəm**-pjʉː-tə, **hɔs**-pɪ-**təl** (2),	he-liː**-kɔp**-tə, **væk-jʉːm**-kliː-nə (2), wɔ-**ʃɪŋ**-mə-**ʃiːn** (2)

				**sɐn**-flæɔː-ə, stɹoː-be-**ɹiːz*****, **ʧɔk**-ə-**lət**[###](2)	
CCV		**skɑe, θɹiː**	ze-**bɹə, snəʉ-**mæn, **plæɪ**-gɹæɔnd, **tɹe**-ʒə,	skɹʉː-**dɹɑe**-və, kəm-**pjʉː**-tə, æm-**bjə**-ləns, sɐn-**flæɔː**-ə	væk-jʉːm-**kliː**-nə
CCVC		**stɐːz, bɹʉːm, stɪk, blæk, tɹæɪn, fɹɔg, fɹɪʤ, swɪŋ**	peŋ-**gwən, pɹez**-ənts, stɹoː-**bɹiːz******		
CVCC		**mɪlk**	ɔ-**ɹænʤ, lɐnʧ-bɔks** (2), spɹɪŋ-**kəlz,** bə-**lʉːnz,**	el-ə-**fənt,** æm-bjə-**ləns**	
CCVCC		**klæɔdz**	plæɪ-**gɹæɔnd**		
CCCV			**stɹoː**-bɹiːz**	**skɹʉː**-dɹɑe-və, **stɹoː**-be-ɹiːz***	
VCCC			pɹez-**ənts**		
CCCVC		**splæʃ**	**spɹɪŋ**-kəlz		

** "Strawberries” with 2 syllables and *** 3 syllables, [##] “Chocolate” with 2 syllables and [###] 3 syllables

Speech sound error patterns

Table *S6*. (a) Developmental and (b) disordered sound (phonological) error patterns

Sound Error Pattern	Description	Example(s)	Number of opportunities
**(a) Developmental**			
Assimilation	Phoneme influences production of another phoneme in target word	• 'lellow' for yellow	48
Cluster reduction	Deletion of:<br>a) one consonant from 2-consonant cluster (bi-cluster reduction), or<br>b) one or two consonants from 3-consonant cluster (tri-cluster reduction)	• 'boom' for broom<br>• 'spinkle' for sprinkle	40[#]
Deaffrication	Replacement of affricate sound with:<br>a) stop sound only<br>b) fricative sound only<br>c) stop sound + substituted fricative sound	• 'wit' for witch<br>• 'wish' for witch<br>• 'wits' for witch	7
Devoicing	Replacement of voiced phoneme with a devoiced phoneme	• 'pik' for pig	40
Final consonant deletion	Deletion of:<br>a) word final consonant(s) or<br>b) syllable final consonant(s)	• 'lea' for leaf<br>• 'bir-day' for birthday	54
Fronting fricatives	Place of articulation of fricative moved to a more anterior position	• 'seep' for sheep	10
Fronting velars	Place of articulation of velar moved to a more anterior position	• 'tar' for 'car	28
Gliding	Replacement of liquids with glides	• 'wainbow' for rainbow<br>• 'yeaf' for leaf	39
Stopping	Replacement of fricatives with stops	• 'doo' for zoo	49
Voicing	Replacement of devoiced phoneme with a voiced phoneme	• 'deeth' for teeth	76
Weak syllable deletion	Deletion of unstressed syllable	• 'di-saur' for dinosaur	22

(b) Disordered

Addition error	addition of sound/syllable to word	• 'star**d**s' for stars
Frication/Affrication	replacement of a sound/cluster with affricate or fricative	• 'vroom' for broom<br>• 'choes' for shoes
Atypical sound substitution	sound substitutions not otherwise categorised in any developmental phonological error patterns	• 'nitch' for witch
Atypical gliding	May also be called liquid replacement i.e. /r/ to /l/, ?glide replacement? i.e. /j/ to /w/, or a combination of each e.g. /w/ to /ɹ/ (the reverse of typical gliding)	• 'bloom' for broom<br>• 'zebya' for zebra<br>• ritch' for witch
Backing	place of articulation moved to more posterior position	• 'kresents' for presents (plosive)<br>• 'leas'for leaf (fricative)<br>• 'nilk' for milk (nasal)
Denasalisation	replacement of a nasal sound with a non-nasal sound	• 'stowman' for snowman
Vowel error	replacement of vowel with another vowel	• 'bord' for bird
Fronting - other	fronting of sounds not encompassed in fricative fronting or velar fronting	• 'prain' for train
Transposition	swapping/inverting position of sound(s) in word	• 'hostipal' for hospital
Reduplication	'complete or partial duplication of a stressed syllable' (DEAP manual)	• 'wash wash' for washing
Backing fricative	'th' to /s/ or /z/	• 'tees' for teeth
Word initial consonant deletion	omission of initial sound/cluster of **word**	• '-ive' for five
Syllable initial consonant deletion	omission of initial sound/cluster of **syllable**	• 'birth-ay' for birthday

bi-cluster reduction = 35, tri-cluster reduction = 5

STATISTICAL ANALYSES: ADDITIONAL INFORMATION

Due to the skewed nature of performance (participants tended to perform better; negatively skewed), the difference in the median percentage of words correct between groups was used to quantify the difference in performance between a) males and females, and b) 6-month age bands. Linear quantile regression (LQR) was used to estimate the difference in median PWC between these groups. The *quantreg* package was used to implement the LQR models.

Comparison between males and females

When comparing males and females, a LQR model was specified with the percentage of words correct as the outcome and a binary variable for *sex* (male or female) included as a covariate (as a main effect only). The coefficient for *sex* in the fitted model provides an estimate for the difference in median percentage of words correct between males and females. Standard errors and associated 95% confidence intervals were obtained via bootstrap, using the inbuilt "boot" feature in the "summary.rq" function (as part of the *quantreg* package). A subgroup analysis was performed for each 12-month age band separately (i.e., a regression model fitted to each subgroup separately) to compare males and females of the same age.

Comparison between 6-month age windows

Within the same age group (12-month bands), the percentage of words correct was compared between those in the first 6 months to those in the last 6 months (i.e., 2;1-2;5 compared to 2;6-2;11). The same analytic approach as above was applied. The LQR model was specified in the same way, with a binary variable for *6-month band* (months 1-5 vs. months 6-12) included as the only covariate (as a main effect). The coefficient for *6-month band* provided an estimate of the median PWC between 6-month windows, with 95% confidence intervals obtained in the same manner as before.

ADDITIONAL RESULTS

Below we present several additional results to support the findings reported in the main manuscript. Results are nested under the same subsection headings as the main manuscript for ease of navigation: participation; phonological accuracy; phonological error patterns; comparison to previous normative data.

Study Participants

Table S7. Demographic characteristics of participants recruited within the study (who responded to at least one word within the assessment) compared to Australian national census data.

	Missing	Sample (n=1179)	ABS Census 2016	ABS Census 2021
	n (%)	n (%)	%	%
**Demographic characteristics**				
Sex	0 (0)			
Female		552 (46.8)	50.7%	50.7%
Male		625 (53.0)	49.3%	49.3%
Another term		2 (0.2)		
Aboriginal or Torres Strait Islander origin	48 (4.1)	59 (5.2)	2.8%	3.2%
Remoteness Area	0 (0)			
Major Cities		846 (71.8)	71.1%	72.0%
Inner Regional		333 (28.2)	18.2%	17.8%
IRSAD Quintile	1 (0.08)			
1 = Most disadvantaged		336 (28.5)	20%	20%
2		308 (26.1)	20%	20%
3		116 (9.8)	20%	20%
4		115 (9.8)	20%	20%
5 = Most advantaged		303 (25.7)	20%	20%
Main language spoken at home	41 (3.5)			
English		1060 (93.2)	72.7%	72.0%
Other		78 (6.8)	22.2%	24.8%
Speaks language other than English	177 (15.0)	204 (20.4)	22.2%	24.8%
Understands language other than English	178 (15.1)	241 (24.1)	22.2%	24.8%

Participation

Two-year-old children were less likely to respond to all 55 words (48.0% attempted all 55 words; Table S8). Words not attempted were typically more phonetically and/or motorically complex multi-syllabic words (e.g., ho-spi-tal (3 syllables), va-cuum clea-ner (4 syllables; Table S9)). From age three years, 86.9% attempted all words, with a majority (> 97%) of participants aged four years or older attempting all words. Seven speech and language therapists collected data (OV, SD, CB, AH, TC, FC, AM).

Table S8. Number of participants who attempted all words during assessment stratified by age group.

**Age group** (years; months)	**n**	**Attempted all 55 words**	
		n	%
2;0 - 2;11	99	48	48.5
3;0 - 3;11	107	93	86.9
4;0 - 4;11	153	151	98.7
5;0 - 5;11	137	134	97.8
6;0 - 6;11	127	127	100.0
7;0 - 7;11	138	137	99.3
8;0 - 8;11	122	121	99.2
9;0 - 9;11	105	105	100.0
10;0 - 10;11	85	85	100.0
11;0 - 11;11	69	69	100.0
12;0 - 12;11	37	37	100.0

Table S9. Top 25 words commonly not responded to across all age levels. [a]Note that sky is the first word on the assessment.

**Word**	**Frequency**
hospital	27
sky[a]	27
ambulance	25
vacuum cleaner	24
lunchbox	21
screwdriver	20
helicopter	19
playground	18
treasure	18
washing machine	17
birthday cake	16
feather	16
computer	14
fridge	14
splash	14
sprinkles	14
sunflower	14
teeth	14
snowman	13
hair	12
kangaroo	12
octopus	12
broom	11
milk	11
presents	11

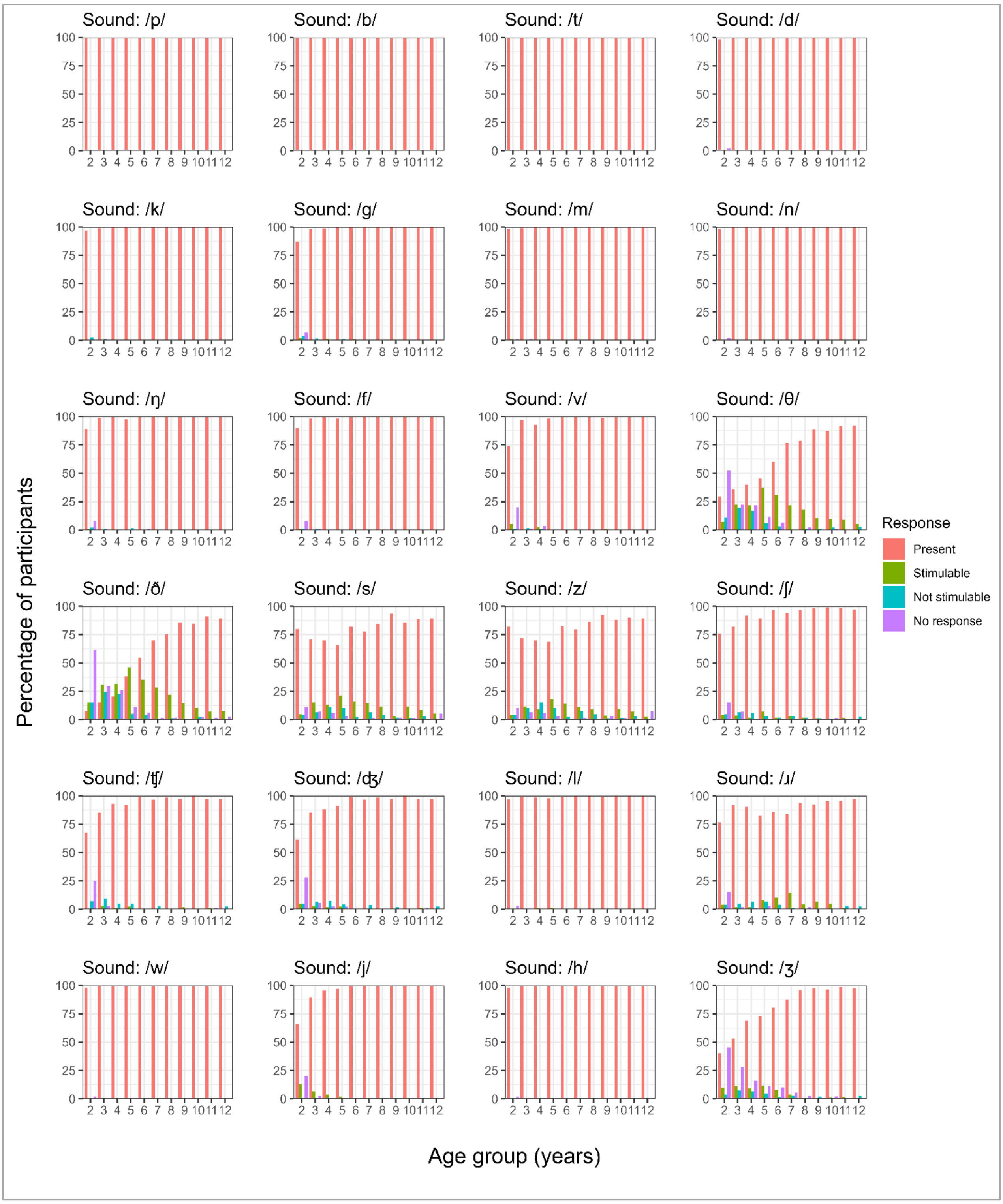

Figure S2. Percentage of participants responding to each sound stratified by age group.

Sound (phonetic) acquisition

Table S10. Proportion of participants (stratified by age group) who had a specific sound present, stimulable, not stimulable or did not respond (denoted as “pres”, “stim”, “not stim”, “no resp”, respectively).

Age band (years; months)	N	Sound															
		/p/				/b/				/t/				/d/			
		Pre	Stim	Not stim	No resp	Pres	Stim	Not stim	No resp	Pres	Stim	Not stim	No resp	Pres	Stim	Not stim	No resp
2;0 - 2;11	99	99.0	0	0	1.0	100	0	0	0	99.0	0	0	1.0	98.0	0	0	2.0
3;0 - 3;11	107	99.1	0	0	0.9	100	0	0	0	100	0	0	0	100	0	0	0
4;0 - 4;11	153	100	0	0	0	100	0	0	0	100	0	0	0	99.3	0.7	0	0
5;0 - 5;11	137	100	0	0	0	100	0	0	0	100	0	0	0	100	0	0	0
6;0 - 6;11	127	100	0	0	0	100	0	0	0	100	0	0	0	100	0	0	0
7;0 - 7;11	138	100	0	0	0	100	0	0	0	100	0	0	0	100	0	0	0
8;0 - 8;11	122	100	0	0	0	100	0	0	0	100	0	0	0	100	0	0	0
9;0 - 9;11	105	99.0	0.1	0	0	100	0	0	0	99.0	0	0	1.0	99.0	0	0	1.0
10;0 - 10;11	85	100	0	0	0	100	0	0	0	100	0	0	0	100	0	0	0
11;0 - 11;11	69	100	0	0	0	100	0	0	0	100	0	0	0	100	0	0	0
12;0 - 12;11	37	100	0	0	0	100	0	0	0	100	0	0	0	100	0	0	0
		/k/				/g/				/m/				/n/			
		Pre	Stim	Not stim	No resp	Pres	Stim	Not stim	No resp	Pres	Stim	Not stim	No resp	Pres	Stim	Not stim	No resp
2;0 - 2;11	99	97	0	3	0	86.9	2	4	7.1	98	1	0	1	98	0	0	2
3;0 - 3;11	107	99.1	0	0.9	0	98.1	0	1.9	0	99.1	0	0	0.9	100	0	0	0
4;0 - 4;11	153	100	0	0	0	98.7	1.3	0	0	100	0	0	0	100	0	0	0
5;0 - 5;11	137	100	0	0	0	99.3	0	0.7	0	100	0	0	0	100	0	0	0
6;0 - 6;11	127	100	0	0	0	99.2	0.8	0	0	100	0	0	0	100	0	0	0
7;0 - 7;11	138	100	0	0	0	100	0	0	0	100	0	0	0	100	0	0	0
8;0 - 8;11	122	100	0	0	0	100	0	0	0	100	0	0	0	100	0	0	0

9;0 - 9;11	105	100	0	0	0	100	0	0	0	100	0	0	0	100	0	0	0
10;0 - 10;11	85	100	0	0	0	100	0	0	0	100	0	0	0	100	0	0	0
11;0 - 11;11	69	100	0	0	0	100	0	0	0	100	0	0	0	100	0	0	0
12;0 - 12;11	37	100	0	0	0	100	0	0	0	100	0	0	0	100	0	0	0
		**/ŋ/**				**/f/**				**/v/**				**/θ/**			
		Pre	Stim	Not stim	No resp	Pres	Stim	Not stim	No resp	Pres	Stim	Not stim	No resp	Pres	Stim	Not stim	No resp
2;0 - 2;11	99	88.9	1	2	8.1	89.9	1	1	8.1	73.7	5.1	1	20.2	29.3	7.1	11.1	52.5
3;0 - 3;11	107	99.1	0	0.9	0	98.1	0	0.9	0.9	97.2	0	1.9	0.9	35.5	22.4	19.6	22.4
4;0 - 4;11	153	99.3	0	0.7	0	99.3	0	0	0.7	92.8	2.6	1.3	3.3	39.9	21.6	17	21.6
5;0 - 5;11	137	97.8	0	1.5	0.7	98.5	0.7	0	0.7	98.5	0	0.7	0.7	45.3	37.2	5.8	11.7
6;0 - 6;11	127	99.2	0	0	0.8	100	0	0	0	100	0	0	0	59.8	30.7	3.1	6.3
7;0 - 7;11	138	99.3	0	0.7	0	99.3	0	0.7	0	99.3	0	0.7	0	76.8	21.7	0.7	0.7
8;0 - 8;11	122	100	0	0	0	100	0	0	0	100	0	0	0	78.7	18	0.8	2.5
9;0 - 9;11	105	100	0	0	0	100	0	0	0	99	1	0	0	88.6	10.5	1	0
10;0 - 10;11	85	100	0	0	0	100	0	0	0	100	0	0	0	87.1	9.4	2.4	1.2
11;0 - 11;11	69	100	0	0	0	100	0	0	0	100	0	0	0	91.3	8.7	0	0
12;0 - 12;11	37	100	0	0	0	100	0	0	0	100	0	0	0	91.9	5.4	2.7	0
		**/ð/**				**/s/**				**/z/**				**/ʃ/**			
		Pres	Stim	Not stim	No resp	Pres	Stim	Not stim	No resp	Pres	Stim	Not stim	No resp	Pres	Stim	Not stim	No resp
2;0 - 2;11	99	8.1	15.2	15.2	61.6	79.8	5.1	4	11.1	81.8	4	4	10.1	75.8	4	5.1	15.2
3;0 - 3;11	107	15	30.8	24.3	29.9	71	15	6.5	7.5	72	11.2	10.3	6.5	82.2	3.7	6.5	7.5
4;0 - 4;11	153	20.3	31.4	22.2	26.1	69.9	13.1	11.1	5.9	69.9	9.2	15	5.9	91.5	2	5.9	0.7
5;0 - 5;11	137	38	46	5.1	10.9	65.7	21.2	10.2	2.9	68.6	18.2	10.2	2.9	89.1	7.3	2.9	0.7
6;0 - 6;11	127	54.3	35.4	3.9	6.3	81.9	15.7	2.4	0	82.7	14.2	2.4	0.8	96.9	1.6	1.6	0
7;0 - 7;11	138	69.6	28.3	0.7	1.4	77.5	14.5	6.5	1.4	79.7	10.9	8	1.4	94.2	2.9	2.9	0
8;0 - 8;11	122	75.4	22.1	0.8	1.6	84.4	11.5	4.1	0	86.1	9	4.9	0	96.7	1.6	1.6	0
9;0 - 9;11	105	85.7	14.3	0	0	93.3	2.9	1.9	1.9	92.4	3.8	1	2.9	98.1	1	1	0
10;0 - 10;11	85	84.7	10.6	2.4	2.4	85.9	11.8	1.2	1.2	88.2	9.4	1.2	1.2	98.8	0	0	1.2
11;0 - 11;11	69	91.3	7.2	0	1.4	88.4	8.7	2.9	0	89.9	7.2	2.9	0	98.6	1.4	0	0
12;0 - 12;11	37	89.2	8.1	0	2.7	89.2	5.4	0	5.4	89.2	2.7	0	8.1	97.3	0	2.7	0
		**/ʧ/**				**/ʤ/**				**/l/**				**/ɹ/**			

		Pre	Stim	Not stim	No resp	Pres	Stim	Not stim	No resp	Pres	Stim	Not stim	No resp	Pres	Stim	Not stim	No resp
2;0 - 2;11	99	67.7	0	7.1	25.3	61.6	5.1	5.1	28.3	97	0	0	3	76.8	4	4	15.2
3;0 - 3;11	107	85	2.8	9.3	2.8	85	2.8	6.5	5.6	99.1	0	0.9	0	91.6	1.9	4.7	1.9
4;0 - 4;11	153	92.8	1.3	5.2	0.7	88.2	2	7.2	2.6	98.7	1.3	0	0	90.2	2	6.5	1.3
5;0 - 5;11	137	92	2.2	5.1	0.7	91.2	2.2	4.4	2.2	97.8	1.5	0.7	0	82.5	8	6.6	2.9
6;0 - 6;11	127	99.2	0.8	0	0	99.2	0.8	0	0	99.2	0.8	0	0	85.8	10.2	3.9	0
7;0 - 7;11	138	96.4	0.7	2.9	0	96.4	0	3.6	0	100	0	0	0	84.1	14.5	1.4	0
8;0 - 8;11	122	98.4	0.8	0.8	0	98.4	0.8	0.8	0	100	0	0	0	93.4	4.1	0.8	1.6
9;0 - 9;11	105	97.1	1.9	1	0	97.1	1	1.9	0	99	0	1	0	92.4	6.7	0	1
10;0 - 10;11	85	100	0	0	0	100	0	0	0	100	0	0	0	95.3	4.7	0	0
11;0 - 11;11	69	97.1	1.4	0	1.4	97.1	1.4	0	1.4	100	0	0	0	95.7	1.4	2.9	0
12;0 - 12;11	37	97.3	0	2.7	0	97.3	0	2.7	0	100	0	0	0	97.3	0	2.7	0

		**/w/**				**/j/**				**/h/**				**/ʒ/**			
		Pres	Stim	Not stim	No Resp	Pres	Stim	Not stim	No Resp	Pres	Stim	Not stim	No Resp	Pres	Stim	Not stim	No Resp
2;0 - 2;11	99	98	0	0	2	65.7	13.1	1	20.2	98	0	0	2	40.4	10.1	4	45.5
3;0 - 3;11	107	100	0	0	0	89.7	6.5	0.9	2.8	99.1	0	0	0.9	53.3	11.2	7.5	28
4;0 - 4;11	153	100	0	0	0	95.4	3.9	0	0.7	99.3	0	0	0.7	68.6	9.2	6.5	15.7
5;0 - 5;11	137	100	0	0	0	97.1	2.2	0	0.7	100	0	0	0	73	11.7	4.4	10.9
6;0 - 6;11	127	100	0	0	0	100	0	0	0	100	0	0	0	80.3	7.9	1.6	10.2
7;0 - 7;11	138	100	0	0	0	100	0	0	0	100	0	0	0	87.7	3.6	2.9	5.8
8;0 - 8;11	122	100	0	0	0	100	0	0	0	100	0	0	0	95.9	0.8	0.8	2.5
9;0 - 9;11	105	99	1	0	0	100	0	0	0	100	0	0	0	97.1	0	1.9	1
10;0 - 10;11	85	100	0	0	0	100	0	0	0	100	0	0	0	96.5	1.2	0	2.4
11;0 - 11;11	69	100	0	0	0	100	0	0	0	100	0	0	0	98.6	1.4	0	0
12;0 - 12;11	37	100	0	0	0	100	0	0	0	100	0	0	0	97.3	0	2.7	0

Sound (phonological) accuracy

Word level

Table S11. Percentage of words correct stratified by age group.

Age group (years; months)	n	All words correct*		Percentage of words correct (PWC)		
		n	%	Median	IQR	Range (min, max)
2;0 - 2;11	99	1	1.0	25.0	(12.1, 44.0)	(0, 78.2)
3;0 - 3;11	107	1	0.9	56.3	(38.2, 73.4)	(0, 100)
4;0 - 4;11	153	3	2.0	67.3	(45.5, 85.5)	(1.8, 100)
5;0 - 5;11	137	8	5.8	83.6	(58.2, 92.7)	(0, 100)
6;0 - 6;11	127	15	11.8	90.9	(84.5, 96.4)	(27.3, 100)
7;0 - 7;11	138	35	25.4	94.5	(87.3, 99.5)	(3.9, 100)
8;0 - 8;11	122	42	34.4	96.4	(92.7, 100)	(32.7, 100)
9;0 - 9;11	105	49	46.7	98.2	(94.5, 100)	(1.8, 100)
10;0 - 10;11	85	43	50.6	100	(94.5, 100)	(21.8, 100)
11;0 - 11;11	69	39	56.5	100	(94.5, 100)	(49.1, 100)
12;0 - 12;11	37	26	70.3	100	(98.2, 100)	(76.4, 100)

*Out of words attempted

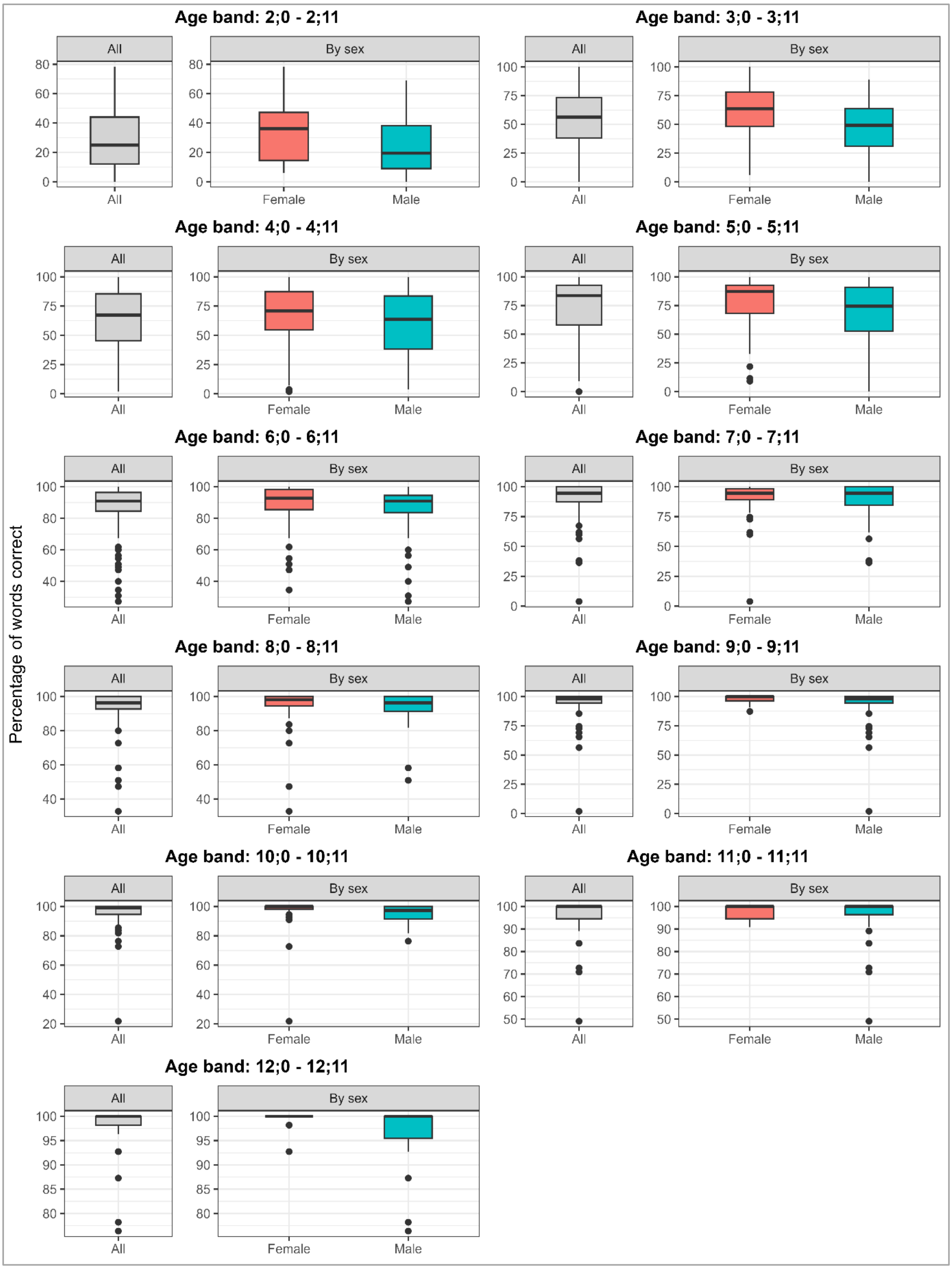

Figure S3. Percentage of words correct (out of attempted words; maximum 55 words) stratified by age group and sex.

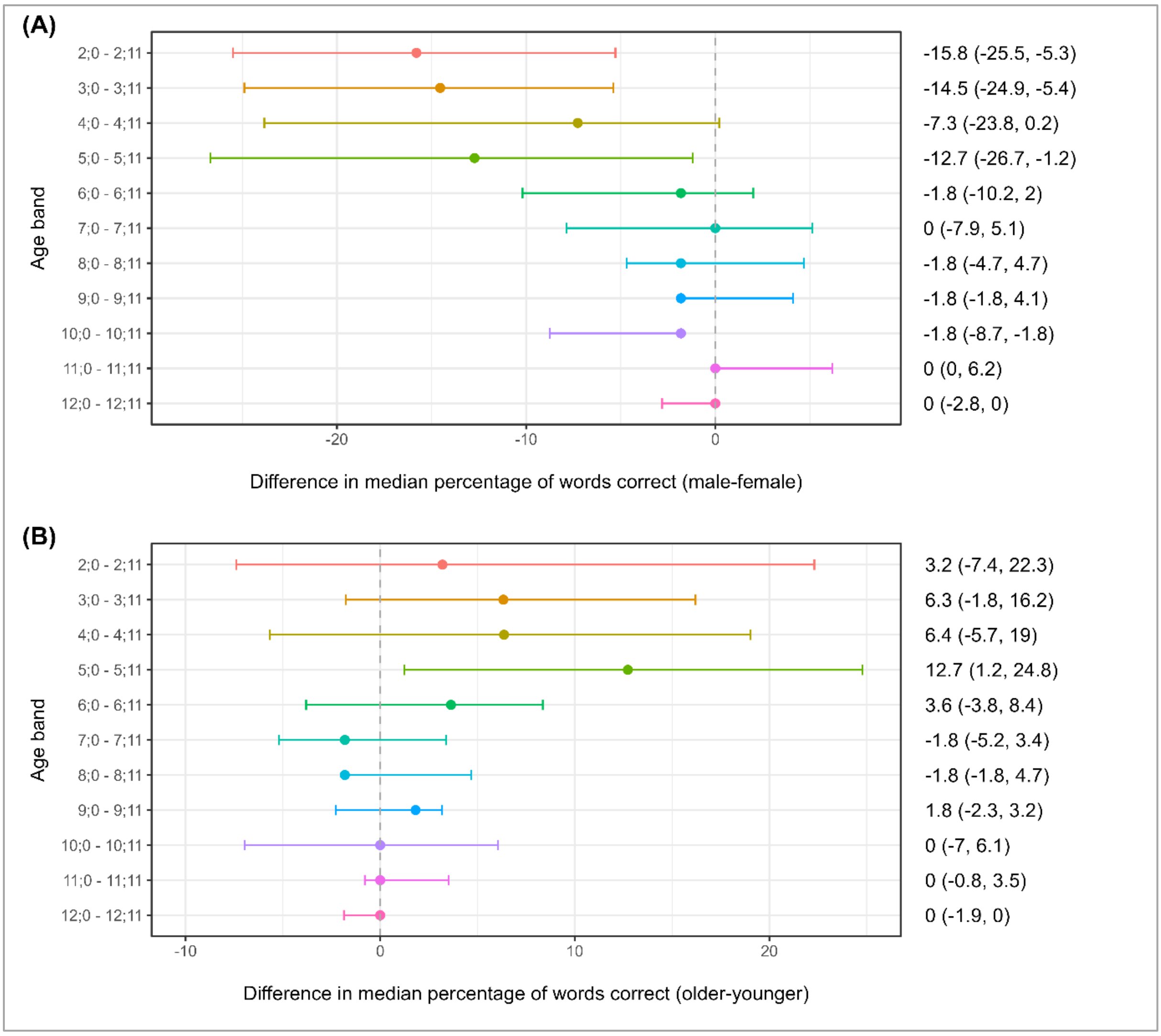

Figure S4. Difference in the median percentage of words correct (with 95% confidence intervals) between (A) males and females within the same age band, and (B) first 6 months of age to the later 6 months. Positive values indicate (A) the males were outperforming the females on average and (B) the older participants (months 6-11) were outperforming the younger participants (months 1-5).

Table S12. Individual cut-points (stratified by 12-month age band) defined corresponding to the lowest performing 10% of the sample at that age. The cut-point represents the number of correctly produced words (i.e., X words or less correct) that would classify the participant as falling into the lowest performing 10% of the sample; this assumed a participant attempted all 55 words.

**Age group**	**n**	**Percentage of words correct (10th percentile)**	**Cut-point: Number of words correct (or lower) to be in bottom 10th percentile of sample**		
(years; months)		%	Exact	Conservative rounding	Inclusive rounding
2;0 - 2;11	99	6.8	3.8	3	4
3;0 - 3;11	107	20.5	11.2	11	12^
4;0 - 4;11	153	17.5	9.6	9	10^
5;0 - 5;11	137	35.6	19.6	19	20
6;0 - 6;11	127	67.3	37	36	37
7;0 - 7;11	138	74	40.7	40	41
8;0 - 8;11	122	87.3	48	48	48
9;0 - 9;11	105	89.8	49.4	49	50*
10;0 - 10;11	85	88.7	48.8	48	49*
11;0 - 11;11	69	92.4	50.8	50	51
12;0 - 12;11	37	92.7	51	51	51

^cut-points differ by two words only therefore cut-points represented on Table S13 are listed as equal for clinical use to ensure possible at risk children are captured *cut-points differ by one word therefore cut-points represented on Table S13 are listed as equal for clinical use to ensure possible at risk children are captured

Table S13. Abbreviated score form to calculate numbers of words correct for 55 picture stimuli of ADAAPT (see Figure S1) with cut point references for number of words correct provided (as per Table S12).

Instructions:

1. Mark child's production of each word from 55 picture stimuli (Figure S1) "Correct" or "Incorrect":
2. Sum the total number of words Correct
3. Refer to cut points for number of words correct based on age (below)

Target word	Correct (✓)	Incorrect (x)	Target word	Correct (✓)	Incorrect (x)
1. sky			30. cheese		
2. stars			31. screwdriver		
3. broom			32. presents		
4. stick			33. milk		
5. black			34. fridge		
6. toilet			35. leaf		
7. shoes			36. swing		
8. ears			37. yellow bird		
9. teeth			38. snowman		
10. orange			39. three		
11. hair			40. five		
12. train			41. rainbow		
13. zoo			42. monkey		
14. zebra			43. giraffe		
15. penguin			44. dinosaur		
16. go			45. balloons		
17. clouds			46. birthday cake		
18. helicopter			47. chocolate		
19. witch			48. washing machine		
20. sheep			49. computer		
21. lunchbox			50. playground		
22. octopus			51. sunflower		
23. vacuum cleaner			52. treasure		
24. kangaroo			53. ambulance		
25. frog			54. hospital		
26. splash			55. feather		
27. elephant			**TOTAL**	**___/55 words correct**	___/55 words incorrect
28. sprinkles					
29. strawberries					
			Age band *(years;months)*	Number of words Correct cut-points*	
			2;0 – 2;11	≤4	*scores listed indicate lowest performing 10% of children per age band
			3;0 – 3;11	≤12	
			4;0 – 4;11	≤12	
			5;0 – 5;11	≤20	
			6;0 – 6;11	≤37	
			7;0 – 7;11	≤41	
			8;0 – 8;11	≤48	
			9;0 – 9;11	≤50	
			10;0 – 10;11	≤50	
			11;0 – 11;11	≤51	
			12;0 – 12;11	≤51	

Sound level accuracy: developmental patterns

Table S14. Proportion of participants responding to all developmental error pattern opportunities by age group (12-month band).

Age group	n	Assimilation	Cluster reduction	Fronting Fricative	Fronting Velar	Weak syllable deletion	Stopping	Gliding	Voicing	Final consonant deletion	Deaffrication	Devoicing
2;0 - 2;11	100	55	52	67	56	61	49	58	49	55	74	61
3;0 - 3;11	107	92.5	90.7	97.2	91.6	92.5	88.8	92.5	89.7	91.6	98.1	92.5
4;0 - 4;11	153	98.7	99.3	99.3	99.3	98.7	98.7	99.3	98.7	98.7	100	98.7
5;0 - 5;11	137	97.8	98.5	98.5	99.3	98.5	97.8	98.5	97.8	97.8	99.3	98.5
6;0 - 6;11	127	100	100	100	100	100	100	100	100	100	100	100
7;0 - 7;11	138	99.3	99.3	99.3	100	99.3	99.3	99.3	99.3	99.3	100	99.3
8;0 - 8;11	122	99.2	99.2	99.2	100	99.2	99.2	99.2	99.2	100	100	99.2
9;0 - 9;11	105	100	100	100	100	100	100	100	100	100	100	100
10;0 - 10;11	85	100	100	100	100	100	100	100	100	100	100	100
11;0 - 11;11	69	100	100	100	100	100	100	100	100	100	100	100
12;0 - 12;11	37	100	100	100	100	100	100	100	100	100	100	100

Table S15. Frequency* of *developmental* error patterns (stratified by type of error) across age groups.

Error pattern	Age group (years; months)	n	≥ 1 error pattern		≥ 5 error patterns[a]		Percentage of error patterns		Ever seen a speech and language therapist (%[b])		
			n	%	n	%	Median	IQR	Yes	No	Missing
Assimilation	2;0 - 2;11	99	84	84.8	14	14.1	4.2	(2.1, 8.33)	14.3	85.7	0
	3;0 - 3;11	107	74	69.2	5	4.7	2.1	(0, 4.17)	0	80	20
	4;0 - 4;11	153	81	52.9	7	4.6	2.1	(0, 2.08)	28.6	28.6	42.9
	5;0 - 5;11	137	54	39.4	1	0.7	0	(0, 2.08)	100	0	0
	6;0 - 6;11	127	22	17.3	0	0	0	(0, 0)	—	—	—
	7;0 - 7;11	138	17	12.3	0	0	0	(0, 0)	—	—	—
	8;0 - 8;11	122	6	4.9	1	0.8	0	(0, 0)	100	0	0
	9;0 - 9;11	105	5	4.8	0	0	0	(0, 0)	—	—	—
	10;0 - 10;11	85	1	1.2	0	0	0	(0, 0)	—	—	—
	11;0 - 11;11	69	0	0	0	0	0	(0, 0)	—	—	—
	12;0 - 12;11	37	0	0	0	0	0	(0, 0)	—	—	—
Cluster reduction	2;0 - 2;11	99	99	100	91	91.9	47.4	(27.5, 65)	7.7	86.8	5.5
	3;0 - 3;11	107	101	94.4	55	51.4	12.5	(5, 28.02)	1.8	67.3	30.9
	4;0 - 4;11	153	136	88.9	50	32.7	5	(2.5, 15)	30	48.0	22.0
	5;0 - 5;11	137	104	75.9	26	19.0	2.5	(2.5, 7.5)	42.3	42.3	15.4
	6;0 - 6;11	127	81	63.8	8	6.3	2.5	(0, 5)	25.0	50	25.0
	7;0 - 7;11	138	68	49.3	7	5.1	0	(0, 2.5)	57.1	28.6	14.3
	8;0 - 8;11	122	45	36.9	2	1.6	0	(0, 2.5)	100	0	0
	9;0 - 9;11	105	29	27.6	1	1.0	0	(0, 2.5)	100	0	0
	10;0 - 10;11	85	23	27.1	2	2.4	0	(0, 2.5)	50	50	0
	11;0 - 11;11	69	18	26.1	0	0	0	(0, 2.5)	—	—	—
	12;0 - 12;11	37	4	10.8	0	0	0	(0, 0)	—	—	—
Deaffrication	2;0 - 2;11	99	81	81.8	28	28.3	42.9	(14.3, 71.43)	10.7	82.1	7.1
	3;0 - 3;11	107	61	57	21	19.6	14.3	(0, 50)	0	76.2	23.8
	4;0 - 4;11	153	53	34.6	16	10.5	0	(0, 14.29)	31.2	43.8	25.0
	5;0 - 5;11	137	38	27.7	11	8	0	(0, 14.29)	45.5	27.3	27.3
	6;0 - 6;11	127	11	8.7	0	0	0	(0, 0)	—	—	—
	7;0 - 7;11	138	12	8.7	2	1.4	0	(0, 0)	50	50	0
	8;0 - 8;11	122	3	2.5	0	0	0	(0, 0)	—	—	—

Error pattern	Age group (years; months)	n	≥ 1 error pattern		≥ 5 error patterns[a]		Percentage of error patterns		Ever seen a speech and language therapist (%[b])		
			n	%	n	%	Median	IQR	Yes	No	Missing
	9;0 - 9;11	105	3	2.9	0	0	0	(0, 0)	—	—	—
	10;0 - 10;11	85	5	5.9	0	0	0	(0, 0)	—	—	—
	11;0 - 11;11	69	0	0	0	0	0	(0, 0)	—	—	—
	12;0 - 12;11	37	0	0	0	0	0	(0, 0)	—	—	—
Devoicing	2;0 - 2;11	99	26	26.3	1	1	0	(0, 2.5)	0	100	0
	3;0 - 3;11	107	23	21.5	2	1.9	0	(0, 0)	0	50	50
	4;0 - 4;11	153	25	16.3	5	3.3	0	(0, 0)	60	20	20
	5;0 - 5;11	137	9	6.6	1	0.7	0	(0, 0)	0	100	0
	6;0 - 6;11	127	4	3.1	0	0	0	(0, 0)	—	—	—
	7;0 - 7;11	138	2	1.4	0	0	0	(0, 0)	—	—	—
	8;0 - 8;11	122	2	1.6	0	0	0	(0, 0)	—	—	—
	9;0 - 9;11	105	1	1	0	0	0	(0, 0)	—	—	—
	10;0 - 10;11	85	1	1.2	0	0	0	(0, 0)	—	—	—
	11;0 - 11;11	69	0	0	0	0	0	(0, 0)	—	—	—
	12;0 - 12;11	37	0	0	0	0	0	(0, 0)	—	—	—
Final consonant deletion	2;0 - 2;11	99	84	84.8	34	34.3	5.6	(1.9, 14.01)	14.7	82.4	2.9
	3;0 - 3;11	107	52	48.6	9	8.4	0	(0, 3.7)	0	88.9	11.1
	4;0 - 4;11	153	61	39.9	10	6.5	0	(0, 1.85)	50	20	30
	5;0 - 5;11	137	34	24.8	7	5.1	0	(0, 0)	28.6	57.1	14.3
	6;0 - 6;11	127	18	14.2	1	0.8	0	(0, 0)	0	100	0
	7;0 - 7;11	138	17	12.3	3	2.2	0	(0, 0)	33.3	66.7	0
	8;0 - 8;11	122	9	7.4	0	0	0	(0, 0)	—	—	—
	9;0 - 9;11	105	4	3.8	1	1	0	(0, 0)	100	0	0
	10;0 - 10;11	85	3	3.5	0	0	0	(0, 0)	—	—	—
	11;0 - 11;11	69	3	4.3	0	0	0	(0, 0)	—	—	—
	12;0 - 12;11	37	0	0	0	0	0	(0, 0)	—	—	—
Fronting fricative	2;0 - 2;11	99	90	90.9	30	30.3	30	(20, 60)	6.7	86.7	6.7
	3;0 - 3;11	107	99	92.5	40	37.4	40	(30, 60)	2.5	75.0	22.5
	4;0 - 4;11	153	137	89.5	33	21.6	30	(20, 40)	24.2	51.5	24.2
	5;0 - 5;11	137	106	77.4	24	17.5	30	(10, 40)	37.5	33.3	29.2
	6;0 - 6;11	127	83	65.4	7	5.5	10	(0, 30)	14.3	57.1	28.6

Error pattern	Age group (years; months)	n	≥ 1 error pattern		≥ 5 error patterns[a]		Percentage of error patterns		Ever seen a speech and language therapist (%[b])		
			n	%	n	%	Median	IQR	Yes	No	Missing
	7;0 - 7;11	138	73	52.9	5	3.6	10	(0, 30)	60	20	20
	8;0 - 8;11	122	47	38.5	1	0.8	0	(0, 27.5)	100	0	0
	9;0 - 9;11	105	32	30.5	1	1	0	(0, 10)	100	0	0
	10;0 - 10;11	85	24	28.2	2	2.4	0	(0, 10)	100	0	0
	11;0 - 11;11	69	11	15.9	0	0	0	(0, 0)	—	—	—
	12;0 - 12;11	37	6	16.2	0	0	0	(0, 0)	—	—	—
Fronting velar	2;0 - 2;11	99	68	68.7	20	20.2	3.7	(0, 14.91)	0	95	5
	3;0 - 3;11	107	50	46.7	12	11.2	0	(0, 3.57)	0	75	25
	4;0 - 4;11	153	54	35.3	11	7.2	0	(0, 3.57)	18.2	36.4	45.5
	5;0 - 5;11	137	26	19	2	1.5	0	(0, 0)	50	0	50
	6;0 - 6;11	127	21	16.5	1	0.8	0	(0, 0)	0	100	0
	7;0 - 7;11	138	18	13	1	0.7	0	(0, 0)	100	0	0
	8;0 - 8;11	122	4	3.3	0	0	0	(0, 0)	—	—	—
	9;0 - 9;11	105	5	4.8	2	1.9	0	(0, 0)	100	0	0
	10;0 - 10;11	85	8	9.4	0	0	0	(0, 0)	—	—	—
	11;0 - 11;11	69	0	0	0	0	0	(0, 0)	—	—	—
	12;0 - 12;11	37	1	2.7	1	2.7	0	(0, 0)	100	0	0
Gliding	2;0 - 2;11	99	95	96	71	71.7	20.5	(12.8, 28.57)	7.0	90.1	2.8
	3;0 - 3;11	107	99	92.5	76	71	20.5	(10.3, 32.88)	2.6	77.6	19.7
	4;0 - 4;11	153	113	73.9	89	58.2	17.9	(0, 35.9)	18.0	52.8	29.2
	5;0 - 5;11	137	82	59.9	49	35.8	2.6	(0, 28.21)	34.7	49.0	16.3
	6;0 - 6;11	127	44	34.6	20	15.7	0	(0, 5.13)	20	50	30
	7;0 - 7;11	138	44	31.9	19	13.8	0	(0, 2.56)	42.1	47.4	10.5
	8;0 - 8;11	122	24	19.7	9	7.4	0	(0, 0)	66.7	33.3	0
	9;0 - 9;11	105	18	17.1	4	3.8	0	(0, 0)	50	25.0	25.0
	10;0 - 10;11	85	12	14.1	3	3.5	0	(0, 0)	66.7	33.3	0
	11;0 - 11;11	69	14	20.3	3	4.3	0	(0, 0)	66.7	33.3	0
	12;0 - 12;11	37	3	8.1	2	5.4	0	(0, 0)	0	100	0
Stopping	2;0 - 2;11	99	79	79.8	23	23.2	4.3	(2, 8.25)	13.0	82.6	4.3
	3;0 - 3;11	107	69	64.5	10	9.3	2	(0, 4.08)	0	60	40
	4;0 - 4;11	153	92	60.1	9	5.9	2	(0, 2.04)	44.4	33.3	22.2

Error pattern	Age group (years; months)	n	≥ 1 error pattern		≥ 5 error patterns[a]		Percentage of error patterns		Ever seen a speech and language therapist (%[b])		
			n	%	n	%	Median	IQR	Yes	No	Missing
	5;0 - 5;11	137	48	35	3	2.2	0	(0, 2.04)	100	0	0
	6;0 - 6;11	127	31	24.4	0	0	0	(0, 0)	—	—	—
	7;0 - 7;11	138	17	12.3	1	0.7	0	(0, 0)	100	0	0
	8;0 - 8;11	122	6	4.9	0	0	0	(0, 0)	13.0	82.6	4.3
	9;0 - 9;11	105	5	4.8	0	0	0	(0, 0)	—	—	—
	10;0 - 10;11	85	3	3.5	0	0	0	(0, 0)	—	—	—
	11;0 - 11;11	69	4	5.8	0	0	0	(0, 0)	—	—	—
	12;0 - 12;11	37	0	0	0	0	0	(0, 0)	—	—	—
Voicing	2;0 - 2;11	99	68	68.7	15	15.2	1.3	(0, 3.95)	6.7	93.3	0
	3;0 - 3;11	107	52	48.6	6	5.6	0	(0, 1.32)	0	83.3	16.7
	4;0 - 4;11	153	74	48.4	6	3.9	0	(0, 1.32)	50	16.7	33.3
	5;0 - 5;11	137	32	23.4	5	3.6	0	(0, 0)	80	20	0
	6;0 - 6;11	127	24	18.9	0	0	0	(0, 0)	—	—	—
	7;0 - 7;11	138	19	13.8	2	1.4	0	(0, 0)	100	0	0
	8;0 - 8;11	122	10	8.2	0	0	0	(0, 0)	—	—	—
	9;0 - 9;11	105	4	3.8	0	0	0	(0, 0)	—	—	—
	10;0 - 10;11	85	4	4.7	0	0	0	(0, 0)	—	—	—
	11;0 - 11;11	69	1	1.4	0	0	0	(0, 0)	—	—	—
	12;0 - 12;11	37	0	0	0	0	0	(0, 0)	—	—	—
Weak syllable deletion	2;0 - 2;11	99	84	84.8	71	71.7	13.6	(5, 27.27)	8.5	87.3	4.2
	3;0 - 3;11	107	62	57.9	41	38.3	4.5	(0, 9.09)	2.4	70.7	26.8
	4;0 - 4;11	153	71	46.4	43	28.1	0	(0, 9.09)	27.9	44.2	27.9
	5;0 - 5;11	137	35	25.5	19	13.9	0	(0, 4.55)	42.1	47.4	10.5
	6;0 - 6;11	127	18	14.2	7	5.5	0	(0, 0)	42.9	57.1	0
	7;0 - 7;11	138	8	5.8	2	1.4	0	(0, 0)	100	0	0
	8;0 - 8;11	122	4	3.3	1	0.8	0	(0, 0)	100	0	0
	9;0 - 9;11	105	4	3.8	2	1.9	0	(0, 0)	100	0	0
	10;0 - 10;11	85	2	2.4	1	1.2	0	(0, 0)	100	0	0
	11;0 - 11;11	69	1	1.4	0	0	0	(0, 0)	—	—	—
	12;0 - 12;11	37	0	0	0	0	0	(0, 0)	—	—	—
Other	2;0 - 2;11	99	94	94.9	68	68.7	16.4	(6.6, 32.61)	8.8	85.3	5.9

Error pattern	Age group (years; months)	n	≥ 1 error pattern		≥ 5 error patterns[a]		Percentage of error patterns		Ever seen a speech and language therapist (%[b])		
			n	%	n	%	Median	IQR	Yes	No	Missing
	3;0 - 3;11	107	90	84.1	39	36.4	5.5	(1.8, 11.92)	0	64.1	35.9
	4;0 - 4;11	153	108	70.6	38	24.8	3.6	(0, 7.27)	26.3	42.1	31.6
	5;0 - 5;11	137	70	51.1	22	16.1	1.8	(0, 3.7)	50.0	27.3	22.7
	6;0 - 6;11	127	60	47.2	3	2.4	0	(0, 1.82)	33.3	66.7	0
	7;0 - 7;11	138	38	27.5	3	2.2	0	(0, 1.82)	100.0	0	0
	8;0 - 8;11	122	19	15.6	2	1.6	0	(0, 0)	100.0	0	0
	9;0 - 9;11	105	13	12.4	1	1.0	0	(0, 0)	100.0	0	0
	10;0 - 10;11	85	12	14.1	1	1.2	0	(0, 0)	100.0	0	0
	11;0 - 11;11	69	3	4.3	0	0	0	(0, 0)	—	—	—
	12;0 - 12;11	37	2	5.4	0	0	0	(0, 0)	—	—	—

*The percentage of participants for each age group exhibiting each error pattern at least once which may denote minor non-clinically significant variations in performance, compared to at least five times as per the clinical cut-point suggestion of Dodd (16) was reported.[a]For weak syllable deletion, reported ≥2 error patterns; [b]Of those participants making ≥ 5 error patterns

Table S16. Frequency of *cluster reduction* errors (stratified by bi-cluster or tri-cluster) across age groups.

**Cluster Reduction** (no. of opportunities)	**Age group (years; months)**	**n**	**≥ 1 error pattern**		**≥ 5 error patterns**		**Percentage of error patterns**	
			n	%	n	%	Median	IQR
Bi-cluster (35)	2;0 - 2;11	99	99	100	84	84.8	47.1	(25.7, 65.7)
	3;0 - 3;11	107	101	94.4	41	38.3	11.4	(5.7, 20.6)
	4;0 - 4;11	153	131	85.6	37	24.2	5.7	(2.9, 11.4)
	5;0 - 5;11	137	97	70.8	17	12.4	2.9	(0, 5.7)
	6;0 - 6;11	127	74	58.3	6	4.7	2.9	(0, 5.7)
	7;0 - 7;11	138	67	48.6	6	4.3	0	(0, 2.9)
	8;0 - 8;11	122	41	33.6	2	1.6	0	(0, 2.9)
	9;0 - 9;11	105	28	26.7	1	1	0	(0, 2.9)
	10;0 - 10;11	85	21	24.7	2	2.4	0	(0, 0)
	11;0 - 11;11	69	15	21.7	0	0	0	(0, 0)
	12;0 - 12;11	37	3	8.1	0	0	0	(0, 0)
Tri-cluster (5)	2;0 - 2;11	98	94	95.9	10	10.2	80	(40, 80)
	3;0 - 3;11	106	77	72.6	4	3.8	30	(0, 60)
	4;0 - 4;11	153	82	53.6	5	3.3	20	(0, 60)
	5;0 - 5;11	137	53	38.7	1	0.7	0	(0, 20)
	6;0 - 6;11	127	26	20.5	0	0	0	(0, 0)
	7;0 - 7;11	138	28	20.3	1	0.7	0	(0, 0)
	8;0 - 8;11	122	9	7.4	0	0	0	(0, 0)
	9;0 - 9;11	105	5	4.8	0	0	0	(0, 0)
	10;0 - 10;11	85	5	5.9	0	0	0	(0, 0)
	11;0 - 11;11	69	5	7.2	0	0	0	(0, 0)
	12;0 - 12;11	37	1	2.7	0	0	0	(0, 0)

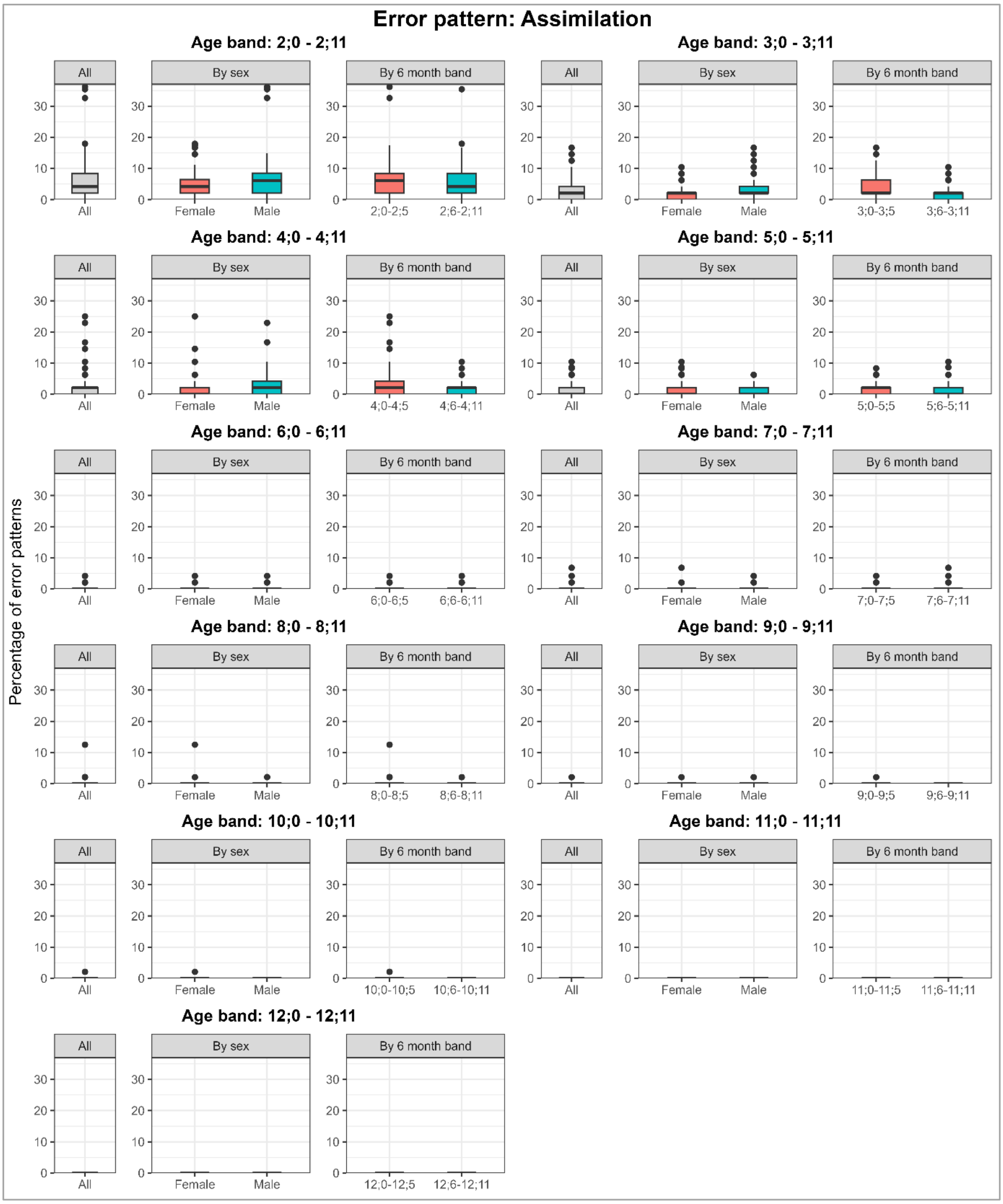

Figure S5. Percentage of assimilation errors stratified by age, sex, and six-month age band.

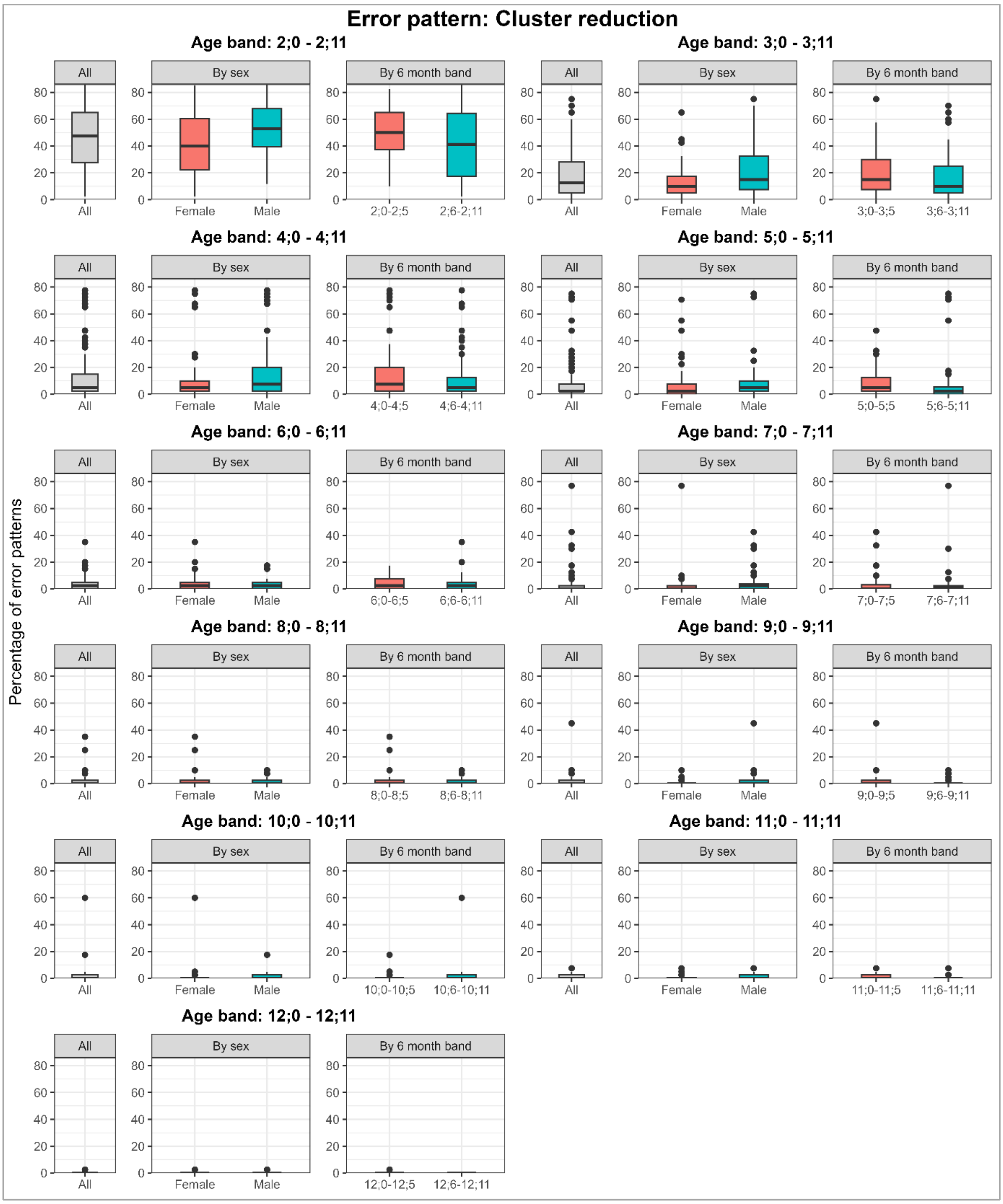

Figure S6. Percentage of cluster reduction errors stratified by age, sex and six-month age band.

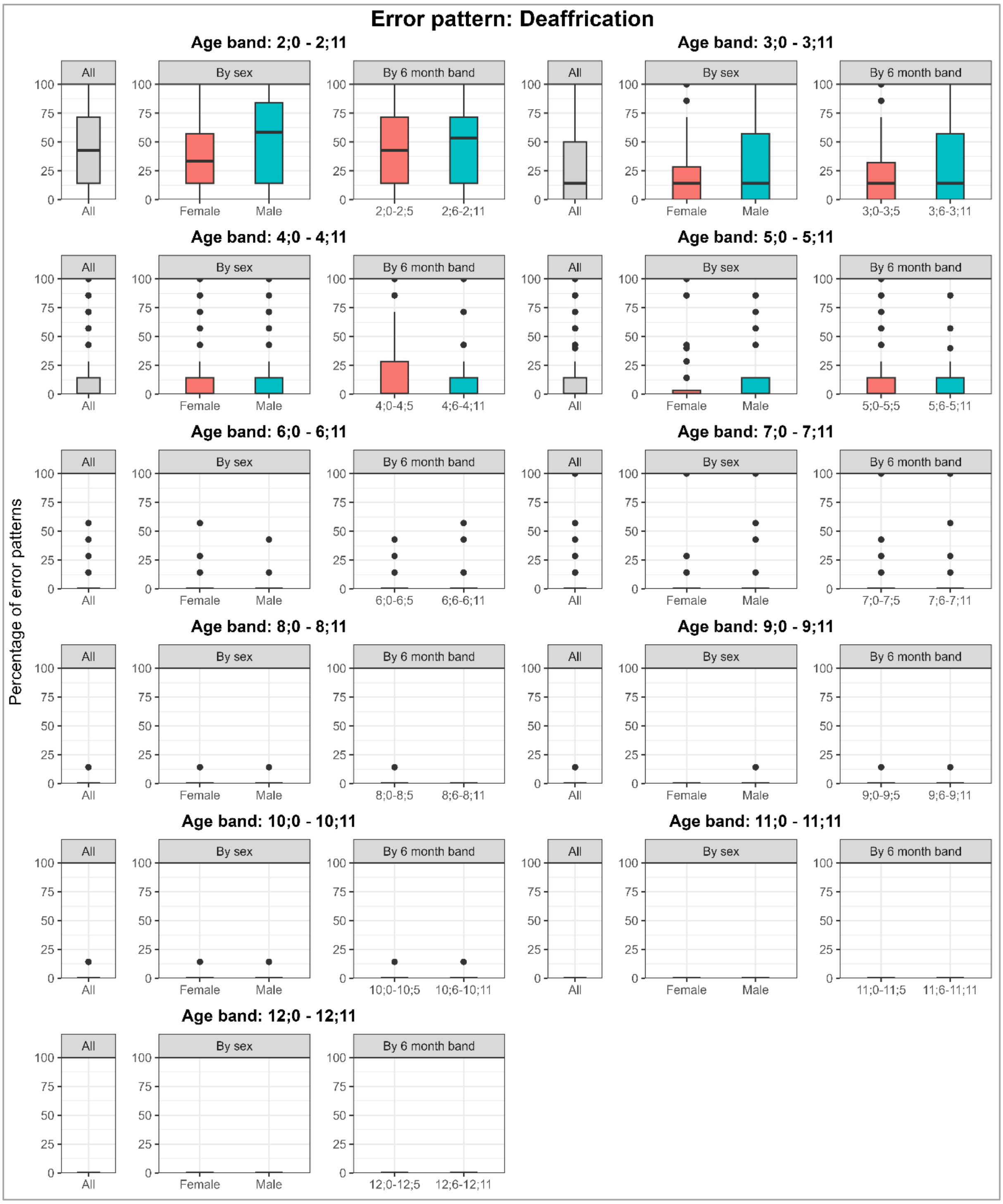

Figure S7. Percentage of deaffrication errors stratified by age, sex and six-month age band.

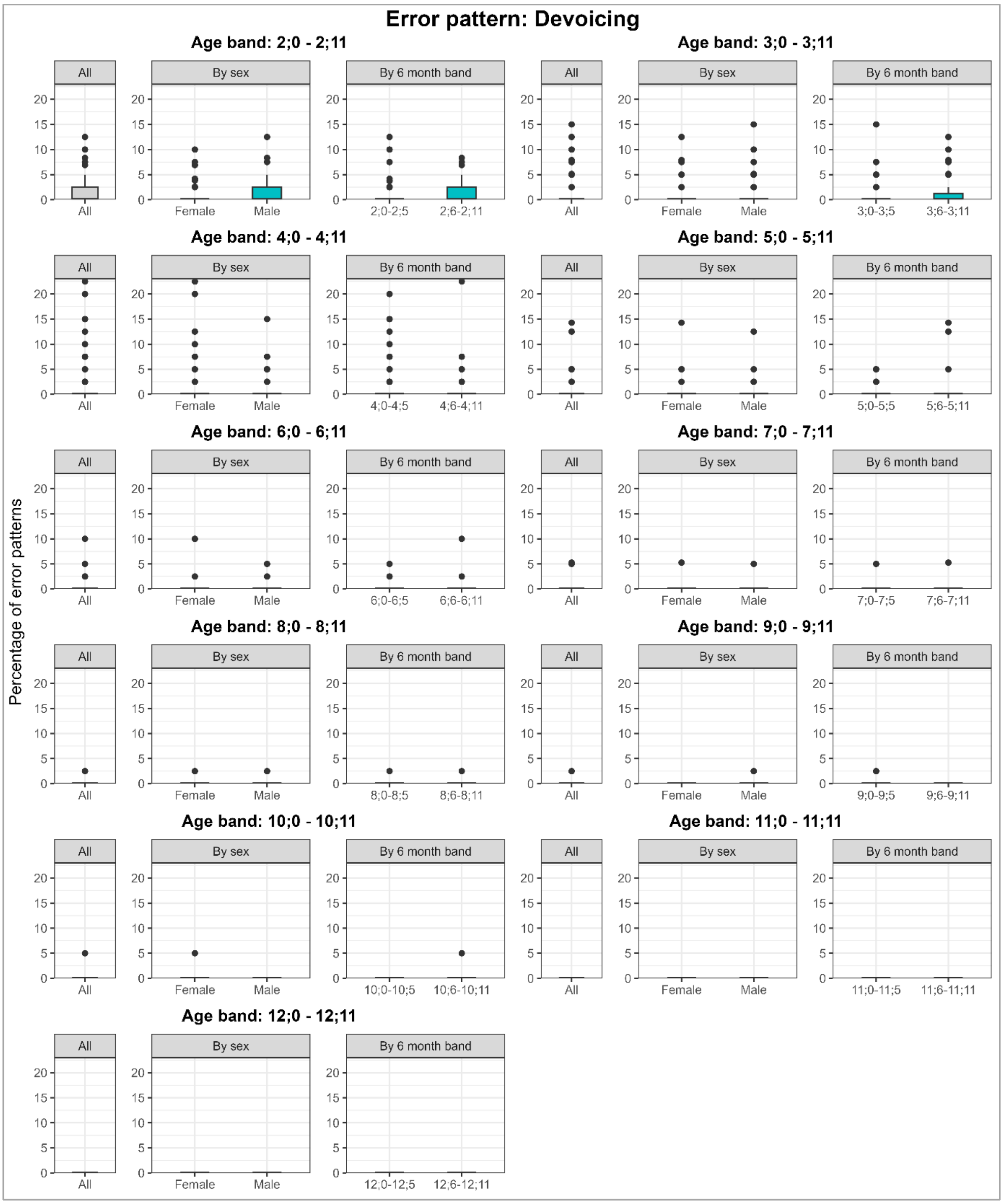

Figure S8. Percentage of devoicing errors stratified by age, sex and six-month age band.

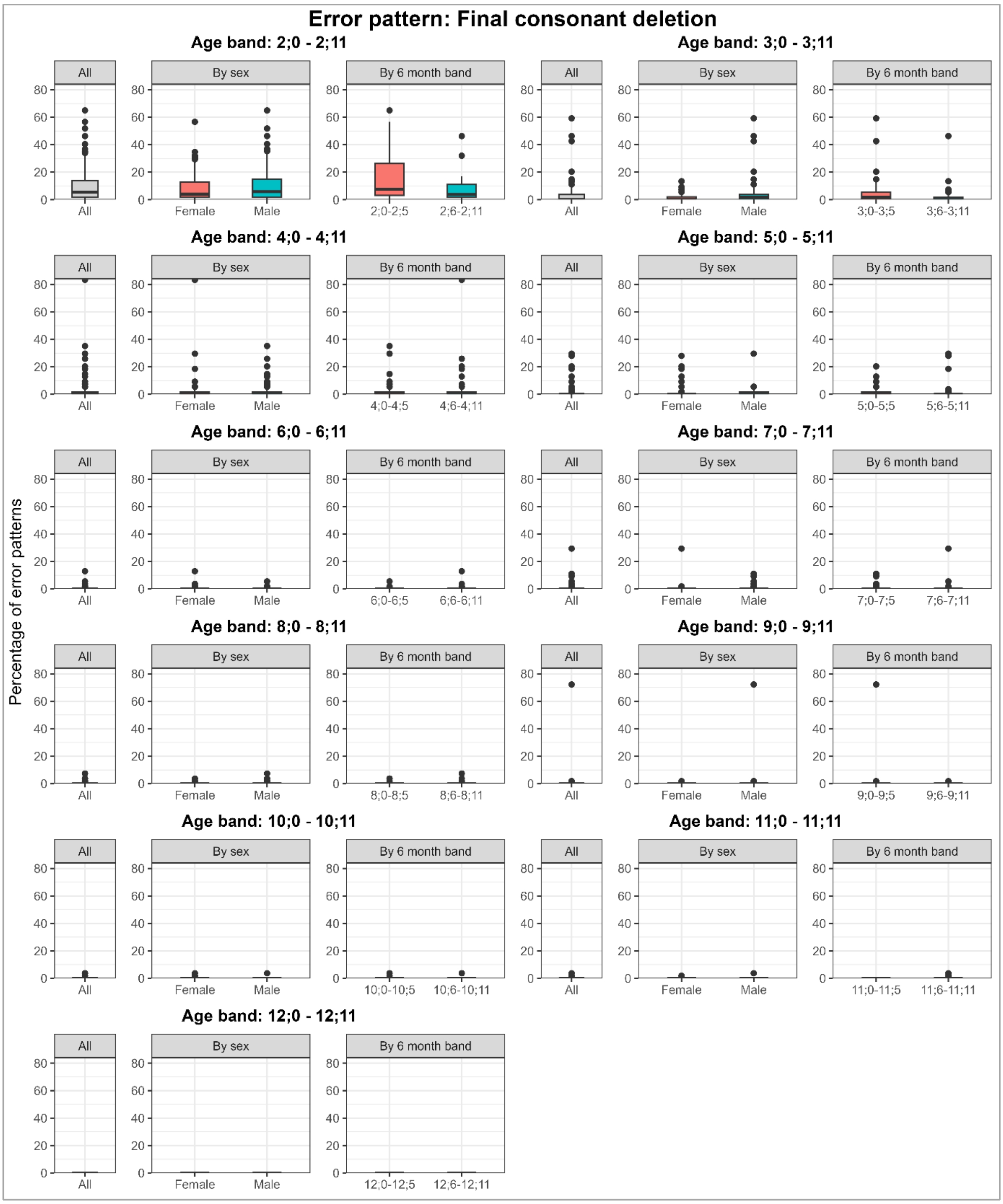

Figure S9. Percentage of final consonant deletion errors stratified by age, sex and six-month age band.

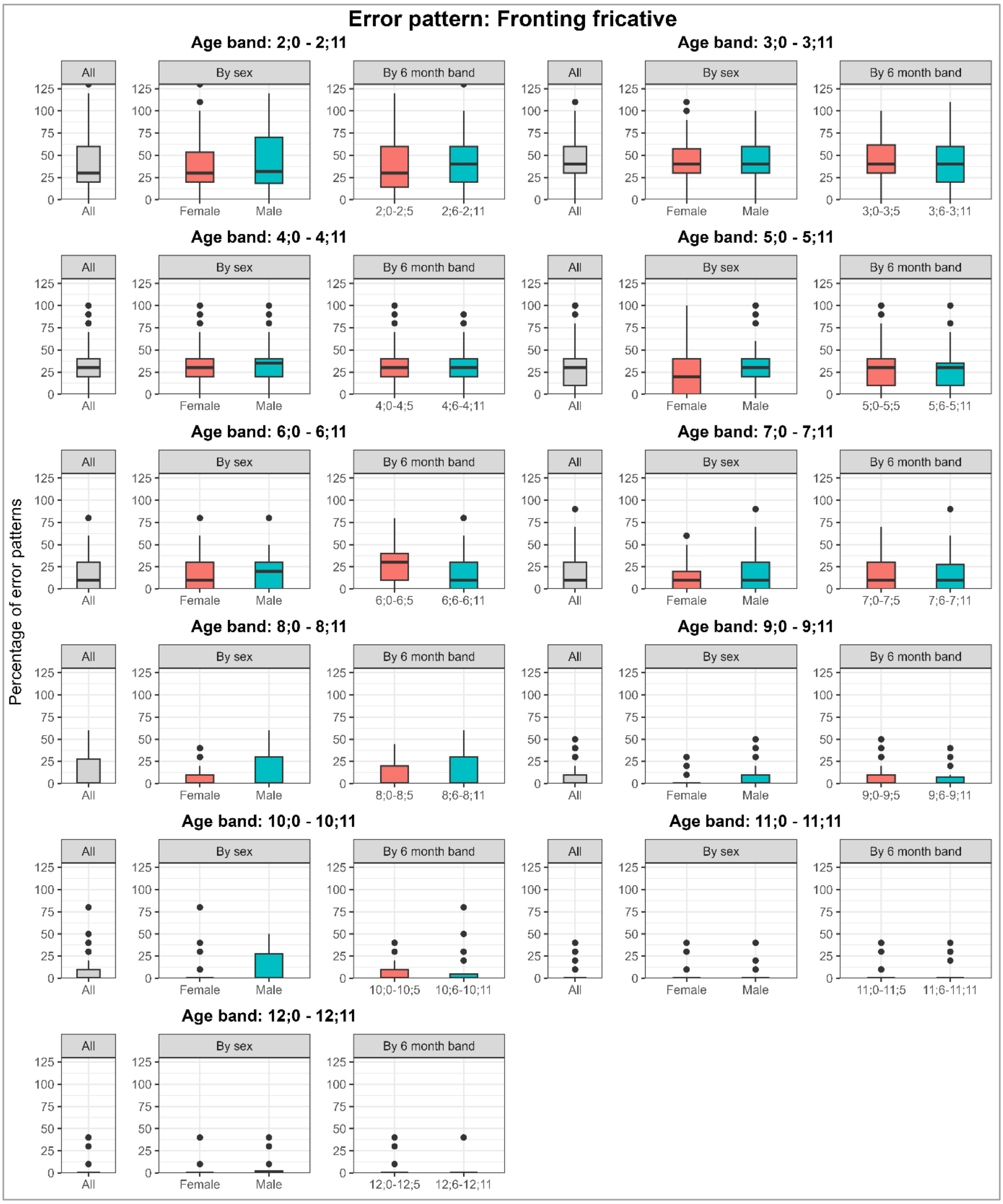

Figure S10. Percentage of fronting fricative errors stratified by age, sex and six-month age band.

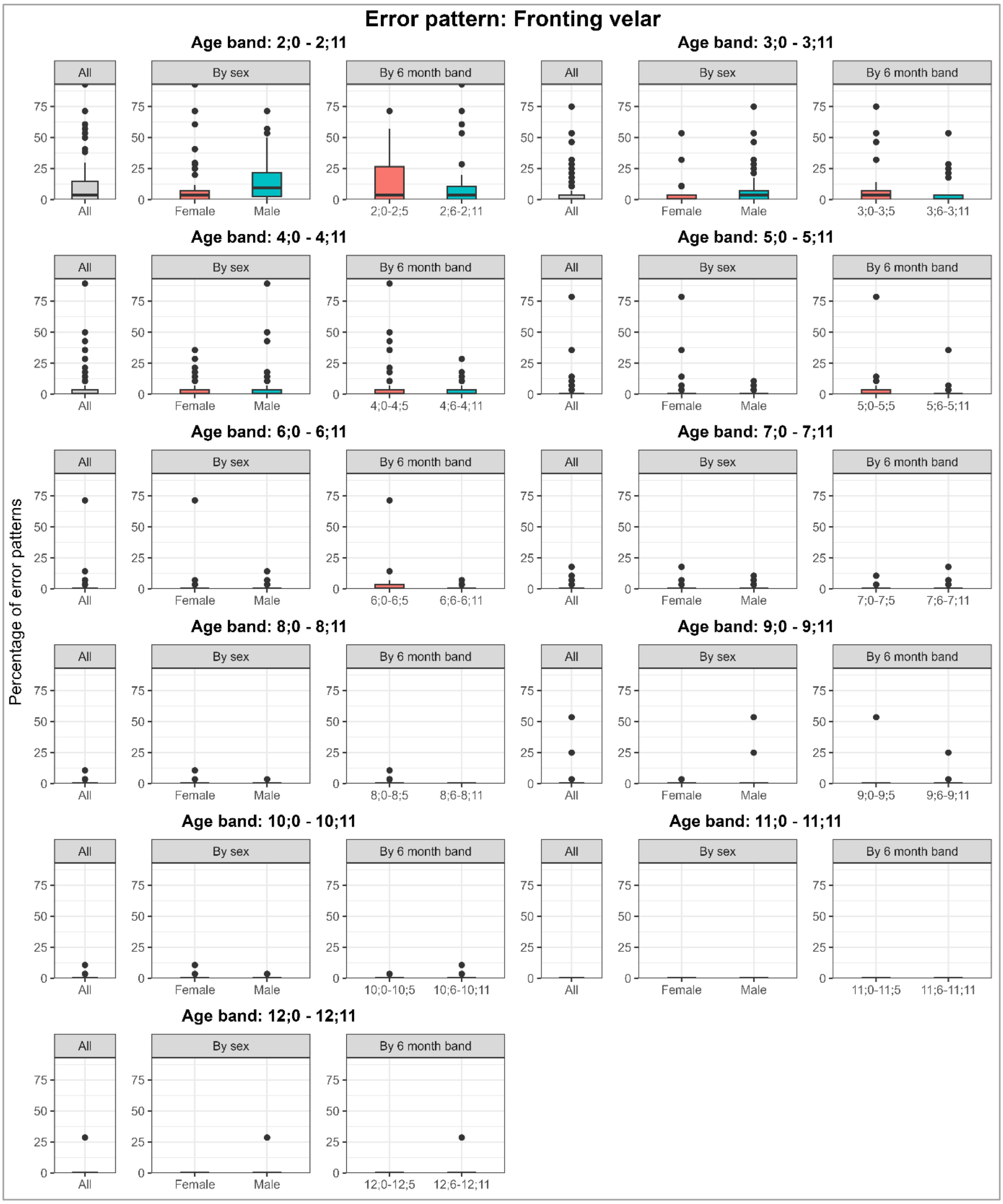

Figure S11. Percentage of fronting velar errors stratified by age, sex and six-month age band.

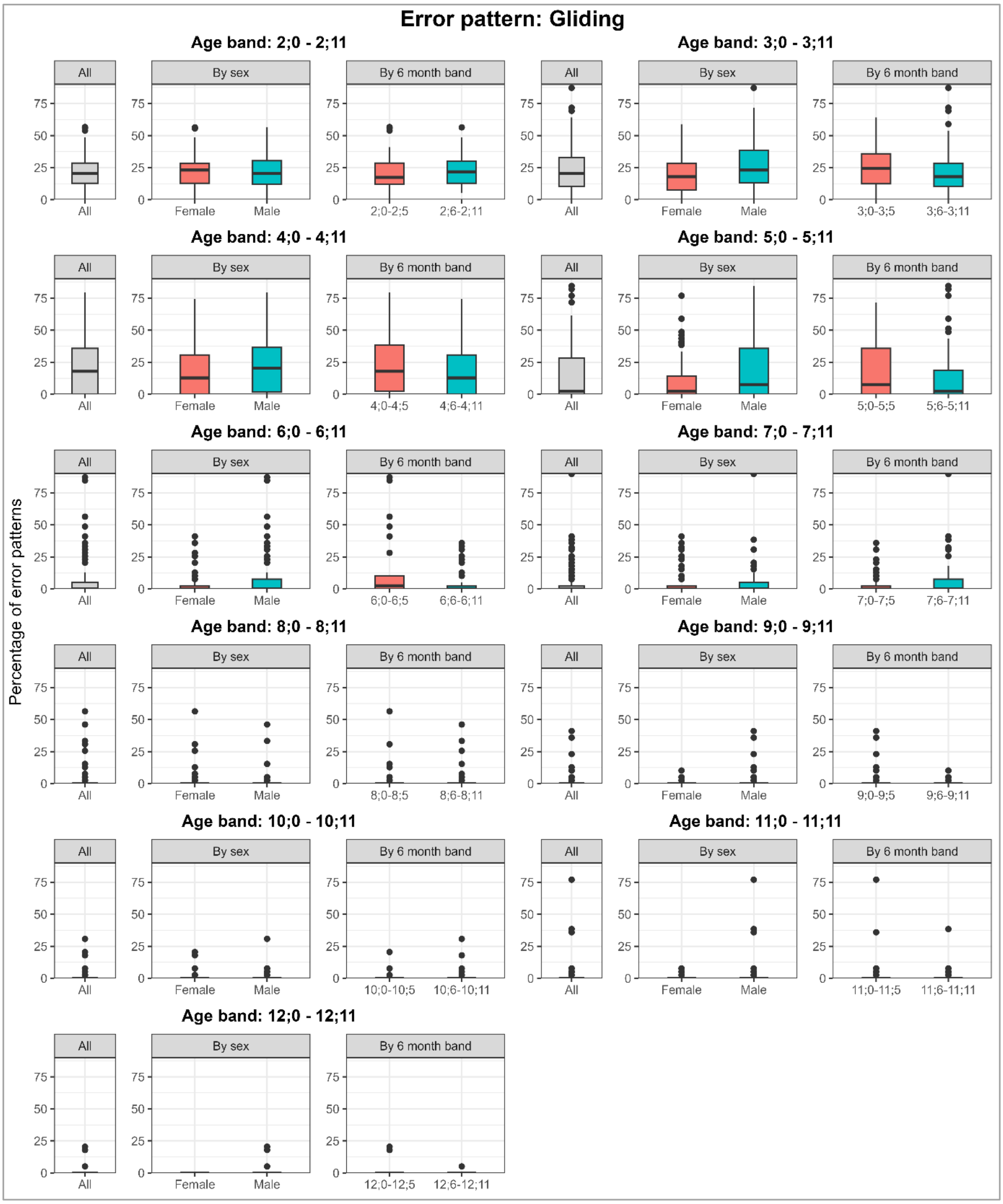

Figure S12. Percentage of gliding errors stratified by age, sex and six-month age band.

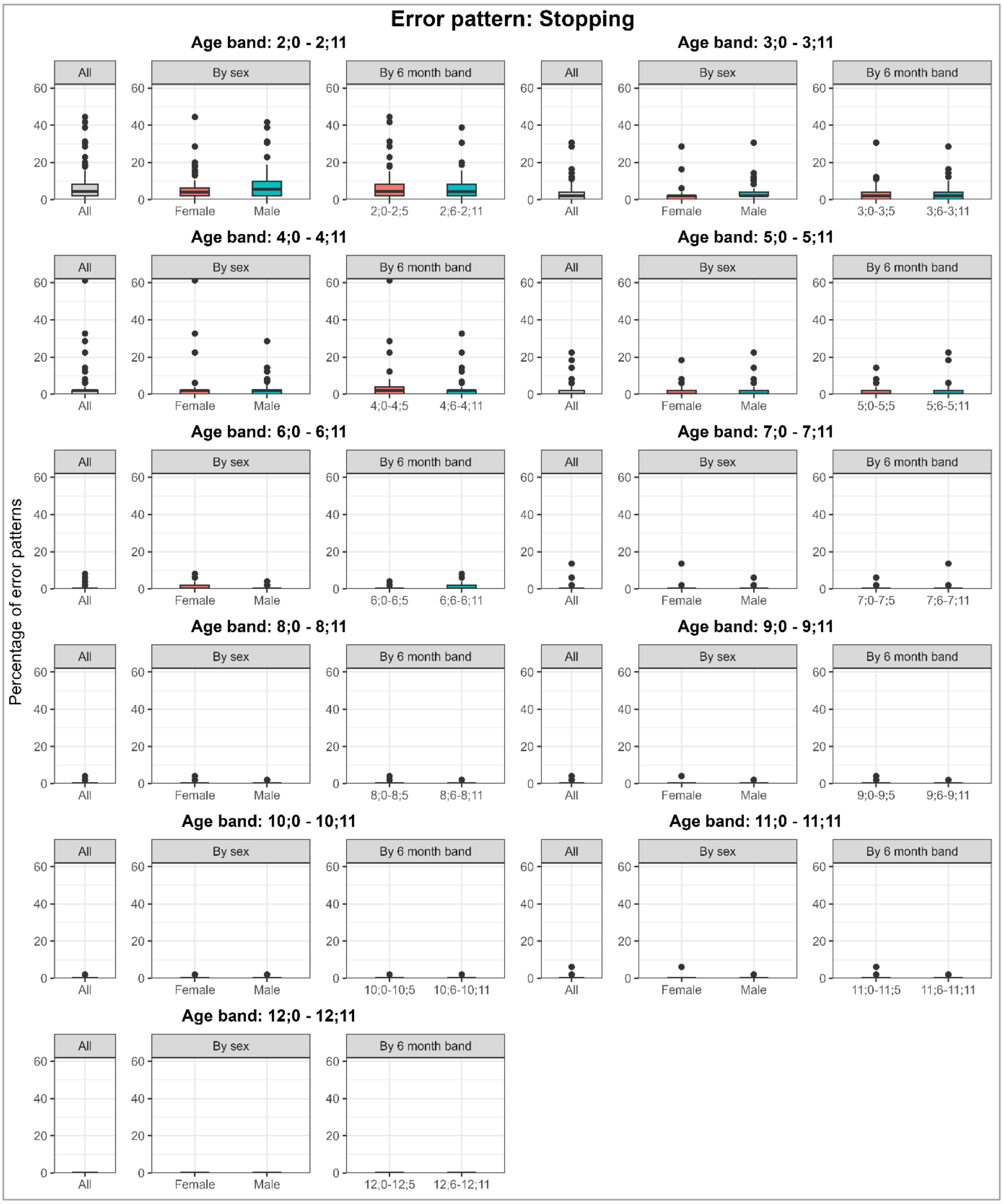

Figure S13. Percentage of stopping errors stratified by age, sex and six-month age bands.

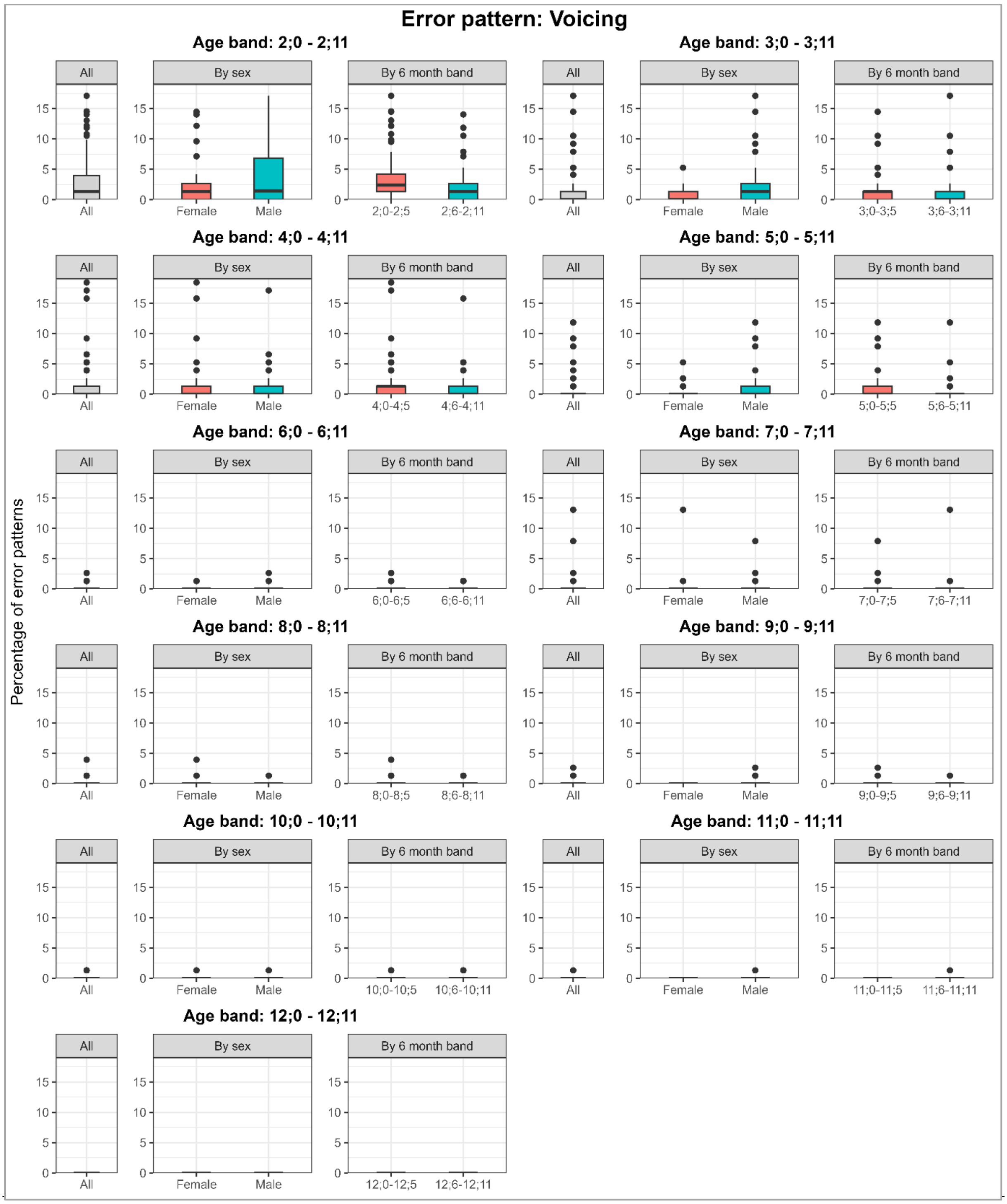

Figure S14. Percentage of voicing errors stratified by age, sex and six-month age bands.

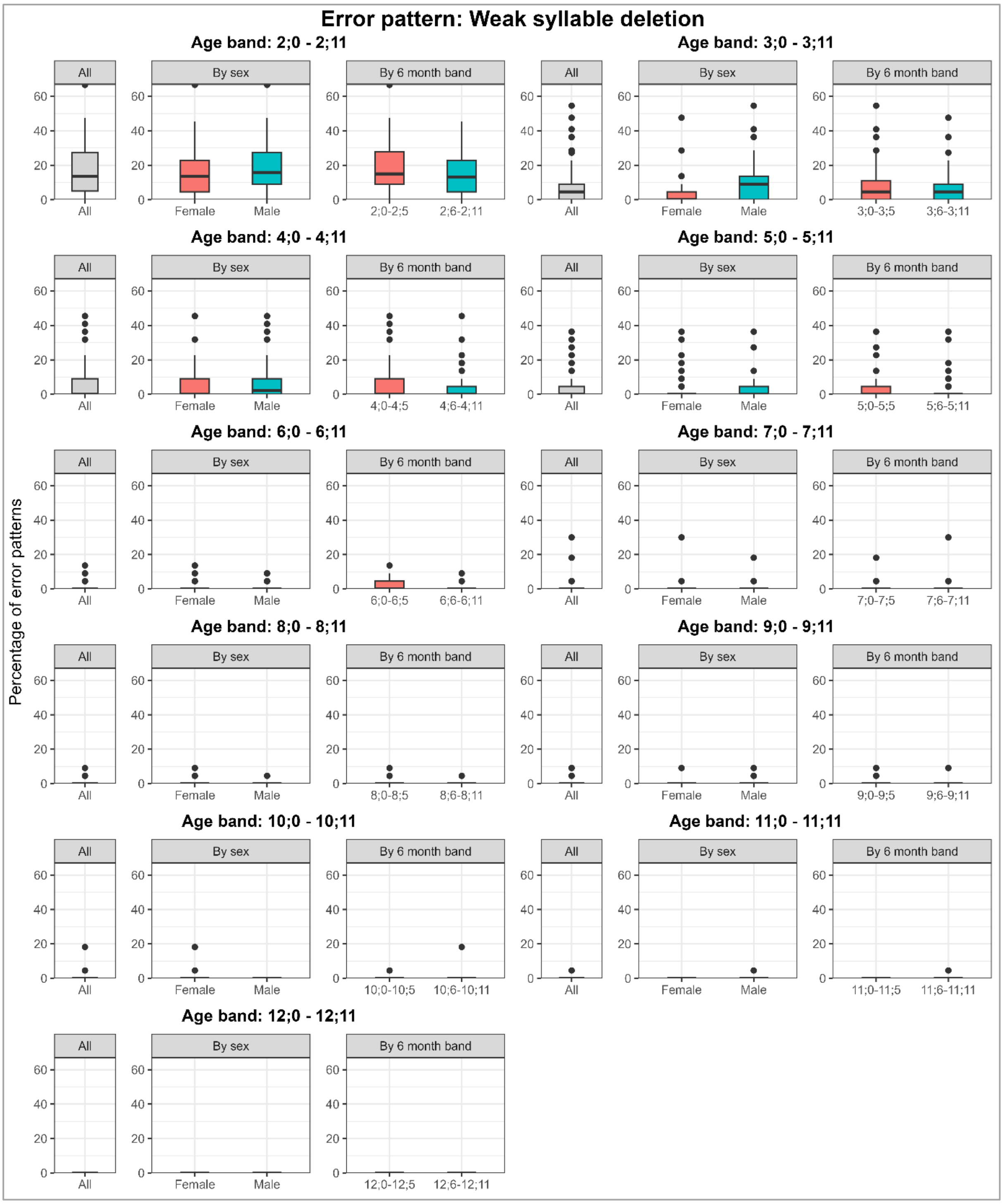

Figure S15. Percentage of weak syllable deletion errors stratified by age, sex and six-month age bands.

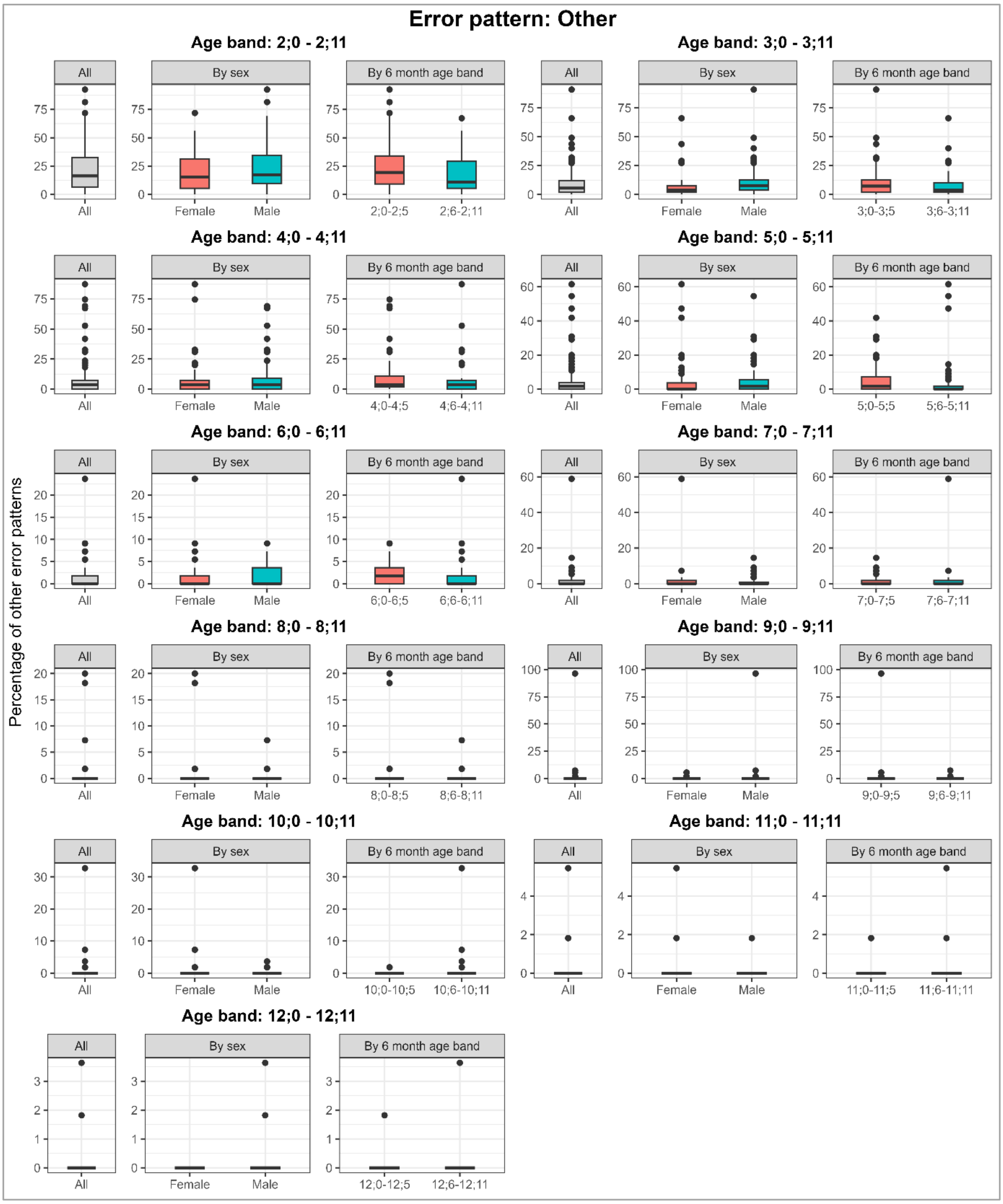

Figure S16. Percentage of other errors stratified by age, sex and six-month age band.

Table S17. Individual cut-points (stratified by developmental error pattern and 12-month age band) defined corresponding to the lowest performing 10% of the sample at that age (i.e., 90th percentile of number of errors). The cut-point represents the number of errors (i.e., X errors or more) that would classify the participant as falling into the lowest performing 10% of the sample; this assumed a participant attempted all opportunities for that error pattern.

**Developmental error pattern (no. of opportunities)**	**Age group**	**n**	**Percentage of errors (90th percentile)**	**Cut-point: Number of errors (or more) to be in lowest performing 10% of sample[a]**		
	(years; months)		%	Exact	Conservative rounding	Inclusive rounding
Assimilation (48)	2;0 - 2;11	99	6.3	3	3	3
	3;0 - 3;11	107	3	1.4	2	1
	4;0 - 4;11	153	2.1	1	1	1
	5;0 - 5;11	137	2.1	1	1	1
	6;0 - 6;11	127	2.1	1	1	1
	7;0 - 7;11	138	2.1	1	1	1
	8;0 - 8;11	122	0	0	0	0
	9;0 - 9;11	105	0	0	0	0
	10;0 - 10;11	85	0	0	0	0
	11;0 - 11;11	69	0	0	0	0
	12;0 - 12;11	37	0	0	0	0
Cluster reduction (40) *Bi-cluster and tri-cluster also presented separately*	2;0 - 2;11	99	56.6	22.6	23	22
	3;0 - 3;11	107	32.5	13	13	13
	4;0 - 4;11	153	25	10	10	10
	5;0 - 5;11	137	15	6	6	6
	6;0 - 6;11	127	7.5	3	3	3
	7;0 - 7;11	138	7.5	3	3	3
	8;0 - 8;11	122	5	2	2	2
	9;0 - 9;11	105	2.5	1	1	1
	10;0 - 10;11	85	2.5	1	1	1
	11;0 - 11;11	69	2.5	1	1	1
	12;0 - 12;11	37	1	0.4	1	0
Deaffrication (7)	2;0 - 2;11	99	85.7	6	6	6
	3;0 - 3;11	107	85.7	6	6	6
	4;0 - 4;11	153	68.6	4.8	5	4
	5;0 - 5;11	137	48.6	3.4	4	3
	6;0 - 6;11	127	0	0	0	0
	7;0 - 7;11	138	0	0	0	0
	8;0 - 8;11	122	0	0	0	0
	9;0 - 9;11	105	0	0	0	0
	10;0 - 10;11	85	0	0	0	0
	11;0 - 11;11	69	0	0	0	0
	12;0 - 12;11	37	0	0	0	0
Devoicing (40)	2;0 - 2;11	99	3	1.2	2	1
	3;0 - 3;11	107	5	2	2	2
	4;0 - 4;11	153	4.5	1.8	2	1
	5;0 - 5;11	137	0	0	0	0
	6;0 - 6;11	127	0	0	0	0

Developmental error pattern (no. of opportunities)	Age group	n	Percentage of errors (90th percentile)	Cut-point: Number of errors (or more) to be in lowest performing 10% of sample[a]		
	(years; months)		%	Exact	Conservative rounding	Inclusive rounding
	7;0 - 7;11	138	0	0	0	0
	8;0 - 8;11	122	0	0	0	0
	9;0 - 9;11	105	0	0	0	0
	10;0 - 10;11	85	0	0	0	0
	11;0 - 11;11	69	0	0	0	0
	12;0 - 12;11	37	0	0	0	0
Final consonant deletion (54)	2;0 - 2;11	99	31.7	17.1	18	17
	3;0 - 3;11	107	7.5	4.1	5	4
	4;0 - 4;11	153	5.6	3	3	3
	5;0 - 5;11	137	1.9	1	1	1
	6;0 - 6;11	127	1.9	1	1	1
	7;0 - 7;11	138	1.9	1	1	1
	8;0 - 8;11	122	0	0	0	0
	9;0 - 9;11	105	0	0	0	0
	10;0 - 10;11	85	0	0	0	0
	11;0 - 11;11	69	0	0	0	0
	12;0 - 12;11	37	0	0	0	0
Fronting fricative (10)	2;0 - 2;11	99	60	6.0	6	6
	3;0 - 3;11	107	70	7.0	7	7
	4;0 - 4;11	153	60	6.0	6	6
	5;0 - 5;11	137	50	5.0	5	5
	6;0 - 6;11	127	40	4.0	4	4
	7;0 - 7;11	138	40	4.0	4	4
	8;0 - 8;11	122	40	4.0	4	4
	9;0 - 9;11	105	30	3.0	3	3
	10;0 - 10;11	85	30	3.0	3	3
	11;0 - 11;11	69	12	1.2	2	1
	12;0 - 12;11	37	18	1.8	2	1
Fronting velar (28)	2;0 - 2;11	99	28.6	8.0	9	8
	3;0 - 3;11	107	15.7	4.4	5	4
	4;0 - 4;11	153	10	2.8	3	2
	5;0 - 5;11	137	3.6	1.0	2	1
	6;0 - 6;11	127	3.6	1.0	2	1
	7;0 - 7;11	138	3.6	1.0	2	1
	8;0 - 8;11	122	0	0.0	0	0
	9;0 - 9;11	105	0	0.0	0	0
	10;0 - 10;11	85	0	0.0	0	0
	11;0 - 11;11	69	0	0.0	0	0
	12;0 - 12;11	37	0	0.0	0	0
Gliding (39)	2;0 - 2;11	99	39.7	15.5	16	15
	3;0 - 3;11	107	44.6	17.4	18	17
	4;0 - 4;11	153	48.7	19	19	19
	5;0 - 5;11	137	48.7	19	19	19
	6;0 - 6;11	127	25.6	10	11	10

Developmental error pattern (no. of opportunities)	Age group	n	Percentage of errors (90th percentile)	Cut-point: Number of errors (or more) to be in lowest performing 10% of sample[a]		
	(years; months)		%	Exact	Conservative rounding	Inclusive rounding
	7;0 - 7;11	138	15.4	6	6	6
	8;0 - 8;11	122	5.1	2	2	2
	9;0 - 9;11	105	5.1	2	2	2
	10;0 - 10;11	85	2.6	1	1	1
	11;0 - 11;11	69	5.1	2	2	2
	12;0 - 12;11	37	0	0	0	0
Stopping (49)	2;0 - 2;11	99	19	9.3	10	9
	3;0 - 3;11	107	8.3	4.1	5	4
	4;0 - 4;11	153	4.1	2	2	2
	5;0 - 5;11	137	4.1	2	2	2
	6;0 - 6;11	127	2	1	1	1
	7;0 - 7;11	138	2	1	1	1
	8;0 - 8;11	122	0	0	0	0
	9;0 - 9;11	105	0	0	0	0
	10;0 - 10;11	85	0	0	0	0
	11;0 - 11;11	69	0	0	0	0
	12;0 - 12;11	37	0	0	0	0
Voicing (76)	2;0 - 2;11	99	10	7.6	8	7
	3;0 - 3;11	107	5.3	4	4	4
	4;0 - 4;11	153	3.9	3	3	3
	5;0 - 5;11	137	1.3	1	1	1
	6;0 - 6;11	127	1.3	1	1	1
	7;0 - 7;11	138	1.3	1	1	1
	8;0 - 8;11	122	0	0	0	0
	9;0 - 9;11	105	0	0	0	0
	10;0 - 10;11	85	0	0	0	0
	11;0 - 11;11	69	0	0	0	0
	12;0 - 12;11	37	0	0	0	0
Weak syllable deletion (22)	2;0 - 2;11	99	37.7	8.3	9	8
	3;0 - 3;11	107	22.7	5	5	5
	4;0 - 4;11	153	18.2	4	4	4
	5;0 - 5;11	137	9.1	2	2	2
	6;0 - 6;11	127	4.5	1	1	1
	7;0 - 7;11	138	0	0	0	0
	8;0 - 8;11	122	0	0	0	0
	9;0 - 9;11	105	0	0	0	0
	10;0 - 10;11	85	0	0	0	0
	11;0 - 11;11	69	0	0	0	0
	12;0 - 12;11	37	0	0	0	0
Bi-cluster reduction (35)	2;0 - 2;11	99	73.5	25.7	26	25
	3;0 - 3;11	107	41.1	14.4	15	14
	4;0 - 4;11	153	39.4	13.8	14	13
	5;0 - 5;11	137	14.3	5	5	5
	6;0 - 6;11	127	6.9	2.4	3	2

Developmental error pattern (no. of opportunities)	Age group	n	Percentage of errors (90th percentile)	Cut-point: Number of errors (or more) to be in lowest performing 10% of sample[a]		
	(years; months)		%	Exact	Conservative rounding	Inclusive rounding
	7;0 - 7;11	138	5.7	2	2	2
	8;0 - 8;11	122	2.9	1	1	1
	9;0 - 9;11	105	2.9	1	1	1
	10;0 - 10;11	85	2.9	1	1	1
	11;0 - 11;11	69	2.9	1	1	1
	12;0 - 12;11	37	0	0	0	0
Tri-cluster reduction (5)	2;0 - 2;11	98	100	5	5	5
	3;0 - 3;11	106	80	4	4	4
	4;0 - 4;11	153	80	4	4	4
	5;0 - 5;11	137	60	3	3	3
	6;0 - 6;11	127	20	1	1	1
	7;0 - 7;11	138	20	1	1	1
	8;0 - 8;11	122	0	0	0	0
	9;0 - 9;11	105	0	0	0	0
	10;0 - 10;11	85	0	0	0	0
	11;0 - 11;11	69	0	0	0	0
	12;0 - 12;11	37	0	0	0	0

[a]When 0 is presented as the cut point, any participants making at least one error would be considered as underperforming (even lower than 10% of the sample).

Sound level accuracy: disordered patterns

Table S18. Frequency and percentage of participants (stratified by age group) exhibiting a disordered error pattern at least once and at least five times during their assessment, alongside the mean, median and maximum number of errors exhibited by participants.

Disordered error	Age band	n	≥ 1 error		≥ 5 errors		Number of errors			Ever seen speech and language therapist (%)		
	(years;months)		n	%	n	%	Mean	Median	Max	Yes	No	Missing
**Addition**	2;0 - 2;11	99	34	34.34	1	1.01	0.52	0	5	0	100	0
	3;0 - 3;11	107	36	33.64	0	0	0.53	0	4	—	—	—
	4;0 - 4;11	153	35	22.88	1	0.65	0.34	0	6	0	100	0
	5;0 - 5;11	137	18	13.14	0	0	0.20	0	3	—	—	—
	6;0 - 6;11	127	12	9.45	0	0	0.12	0	3	—	—	—
	7;0 - 7;11	138	5	3.62	0	0	06	0	4	—	—	—
	8;0 - 8;11	122	3	2.46	0	0	02	0	1	—	—	—
	9;0 - 9;11	105	2	1.90	1	0.95	0.10	0	9	100	0	0
	10;0 - 10;11	85	3	3.53	0	0	05	0	2	—	—	—
	11;0 - 11;11	69	0	00	0	0	00	0	0	—	—	—
	12;0 - 12;11	37	0	00	0	0	00	0	0	—	—	—
**Frication/ Affrication**	2;0 - 2;11	99	40	40.40	2	2.02	0.74	0	8	0	100	0
	3;0 - 3;11	107	28	26.17	3	2.80	0.57	0	7	0	66.7	33.3
	4;0 - 4;11	153	39	25.49	3	1.96	0.55	0	9	0	33.3	66.7
	5;0 - 5;11	137	24	17.52	1	0.73	0.33	0	8	100	0	0
	6;0 - 6;11	127	21	16.54	0	0	0.20	0	3	—	—	—
	7;0 - 7;11	138	10	7.25	0	0	08	0	2	—	—	—
	8;0 - 8;11	122	1	0.82	0	0	03	0	4	—	—	—
	9;0 - 9;11	105	3	2.86	0	0	03	0	1	—	—	—
	10;0 - 10;11	85	1	1.18	0	0	01	0	1	—	—	—
	11;0 - 11;11	69	1	1.45	0	0	01	0	1	—	—	—
	12;0 - 12;11	37	0	00	0	0	00	0	0	—	—	—
**Atypical sound substitution**	2;0 - 2;11	99	37	37.37	4	4.04	0.91	0	12	50	50	0
	3;0 - 3;11	107	14	13.08	4	3.74	0.49	0	14	0	50	50
	4;0 - 4;11	153	15	9.80	2	1.31	0.32	0	18	50	0	50
	5;0 - 5;11	137	8	5.84	1	0.73	0.15	0	5	100	0	0
	6;0 - 6;11	127	1	0.79	0	0	02	0	2	—	—	—

	7;0 - 7;11	138	2	1.45	1	0.72	09	0	11	100	0	0
	8;0 - 8;11	122	2	1.64	0	0	02	0	1	—	—	—
	9;0 - 9;11	105	0	00	0	0	00	0	0	—	—	—
	10;0 - 10;11	85	1	1.18	0	0	01	0	1	—	—	—
	11;0 - 11;11	69	0	00	0	0	00	0	0	—	—	—
	12;0 - 12;11	37	0	00	0	0	00	0	0	—	—	—
**Atypical gliding**	2;0 - 2;11	99	17	17.17	1	1.01	0.27	0	7	0	100	0
	3;0 - 3;11	107	17	15.89	1	0.93	0.22	0	6	0	100	0
	4;0 - 4;11	153	20	13.07	1	0.65	0.29	0	13	0	0	100
	5;0 - 5;11	137	11	8.03	3	2.19	0.20	0	8	33.3	66.7	0
	6;0 - 6;11	127	6	4.72	0	0	07	0	3	—	—	—
	7;0 - 7;11	138	0	00	0	0	00	0	0	—	—	—
	8;0 - 8;11	122	1	0.82	0	0	01	0	1	—	—	—
	9;0 - 9;11	105	1	0.95	0	0	01	0	1	—	—	—
	10;0 - 10;11	85	1	1.18	0	0	01	0	1	—	—	—
	11;0 - 11;11	69	0	00	0	0	00	0	0	—	—	—
	12;0 - 12;11	37	0	00	0	0	00	0	0	—	—	—
**Backing**	2;0 - 2;11	99	50	50.51	7	7.07	1.25	1	11	0	100	0
	3;0 - 3;11	107	23	21.50	1	0.93	0.40	0	12	0	100	0
	4;0 - 4;11	153	31	20.26	5	3.27	0.69	0	16	40	20	40
	5;0 - 5;11	137	15	10.95	2	1.46	0.23	0	6	50	50	0
	6;0 - 6;11	127	5	3.94	0	0	05	0	2	—	—	—
	7;0 - 7;11	138	4	2.90	0	0	04	0	2	—	—	—
	8;0 - 8;11	122	3	2.46	0	0	06	0	4	—	—	—
	9;0 - 9;11	105	1	0.95	1	0.95	0.87	0	91[a]	100	0	0
	10;0 - 10;11	85	1	1.18	0	0	01	0	1	—	—	—
	11;0 - 11;11	69	0	00	0	0	00	0	0	—	—	—
	12;0 - 12;11	37	0	00	0	0	00	0	0	—	—	—
**Denasalisation**	2;0 - 2;11	99	0	00	0	0	00	0	0	—	—	—
	3;0 - 3;11	107	3	2.80	0	0	03	0	1	—	—	—
	4;0 - 4;11	153	1	0.65	0	0	01	0	1	—	—	—
	5;0 - 5;11	137	0	00	0	0	00	0	0	—	—	—
	6;0 - 6;11	127	0	00	0	0	00	0	0	—	—	—
	7;0 - 7;11	138	0	00	0	0	00	0	0	—	—	—
	8;0 - 8;11	122	0	00	0	0	00	0	0	—	—	—

	9;0 - 9;11	105	0	00	0	0	00	0	0	—	—	—
	10;0 - 10;11	85	0	00	0	0	00	0	0	—	—	—
	11;0 - 11;11	69	0	00	0	0	00	0	0	—	—	—
	12;0 - 12;11	37	0	00	0	0	00	0	0	—	—	—
**Vowel error**	2;0 - 2;11	99	63	63.64	20	20.20	2.91	1	25	15.0	85.0	0
	3;0 - 3;11	107	50	46.73	6	5.61	1.34	0	35	0	66.7	33.3
	4;0 - 4;11	153	56	36.60	7	4.58	0.96	0	34	57.1	0	42.9
	5;0 - 5;11	137	42	30.66	6	4.38	0.82	0	28	50.0	33.3	16.7
	6;0 - 6;11	127	19	14.96	0	0	0.20	0	3	—	—	—
	7;0 - 7;11	138	21	15.22	1	0.72	0.27	0	14	100	0	0
	8;0 - 8;11	122	13	10.66	0	0	0.14	0	4	—	—	—
	9;0 - 9;11	105	5	4.76	1	0.95	0.45	0	43	100	0	0
	10;0 - 10;11	85	9	10.59	0	0	0.13	0	3	—	—	—
	11;0 - 11;11	69	1	1.45	0	0	01	0	1	—	—	—
	12;0 - 12;11	37	0	00	0	0	00	0	0	—	—	—
**Transposition**	2;0 - 2;11	99	42	42.42	2	2.02	0.90	0	5	50.0	50.0	0
	3;0 - 3;11	107	29	27.10	0	0	0.37	0	4	—	—	—
	4;0 - 4;11	153	27	17.65	2	1.31	0.33	0	8	0	0	100
	5;0 - 5;11	137	25	18.25	0	0	0.23	0	3	—	—	—
	6;0 - 6;11	127	10	7.87	0	0	0.10	0	2	—	—	—
	7;0 - 7;11	138	12	8.70	0	0	0.11	0	4	—	—	—
	8;0 - 8;11	122	11	9.02	0	0	09	0	1	—	—	—
	9;0 - 9;11	105	4	3.81	0	0	05	0	2	—	—	—
	10;0 - 10;11	85	2	2.35	1	1.18	08	0	6	100	0	0
	11;0 - 11;11	69	1	1.45	0	0	01	0	1	—	—	—
	12;0 - 12;11	37	0	00	0	0	00	0	0	—	—	—
**Th to SZ**	2;0 - 2;11	99	23	23.23	0	0	0.26	0	2	—	—	—
	3;0 - 3;11	107	14	13.08	0	0	0.17	0	2	—	—	—
	4;0 - 4;11	153	10	6.54	0	0	08	0	2	—	—	—
	5;0 - 5;11	137	7	5.11	0	0	07	0	2	—	—	—
	6;0 - 6;11	127	2	1.57	0	0	02	0	2	—	—	—
	7;0 - 7;11	138	3	2.17	0	0	02	0	1	—	—	—
	8;0 - 8;11	122	0	00	0	0	00	0	0	—	—	—
	9;0 - 9;11	105	0	00	0	0	00	0	0	—	—	—
	10;0 - 10;11	85	1	1.18	0	0	01	0	1	—	—	—

	11;0 - 11;11	69	0	00	0	0	00	0	0	—	—	—
	12;0 - 12;11	37	0	00	0	0	00	0	0	—	—	—
**Word initial deletion**	2;0 - 2;11	99	28	28.28	2	2.02	0.59	0	8	50.0	50.0	0
	3;0 - 3;11	107	17	15.89	1	0.93	0.29	0	10	0	100	0
	4;0 - 4;11	153	11	7.19	0	0	0.12	0	4	—	—	—
	5;0 - 5;11	137	12	8.76	0	0	0.10	0	3	—	—	—
	6;0 - 6;11	127	1	0.79	0	0	01	0	1	—	—	—
	7;0 - 7;11	138	1	0.72	0	0	03	0	4	—	—	—
	8;0 - 8;11	122	2	1.64	0	0	02	0	1	—	—	—
	9;0 - 9;11	105	0	00	0	0	00	0	0	—	—	—
	10;0 - 10;11	85	0	00	0	0	00	0	0	—	—	—
	11;0 - 11;11	69	0	00	0	0	00	0	0	—	—	—
	12;0 - 12;11	37	0	00	0	0	00	0	0	—	—	—
**Syllable initial deletion**	2;0 - 2;11	99	32	32.32	2	2.02	0.59	0	5	50.0	50.0	0
	3;0 - 3;11	107	14	13.08	1	0.93	0.27	0	6	0	100	0
	4;0 - 4;11	153	15	9.80	0	0	0.18	0	4	—	—	—
	5;0 - 5;11	137	7	5.11	0	0	06	0	2	—	—	—
	6;0 - 6;11	127	2	1.57	0	0	02	0	2	—	—	—
	7;0 - 7;11	138	1	0.72	0	0	01	0	2	—	—	—
	8;0 - 8;11	122	3	2.46	0	0	06	0	3	—	—	—
	9;0 - 9;11	105	1	0.95	0	0	03	0	3	—	—	—
	10;0 - 10;11	85	1	1.18	0	0	02	0	2	—	—	—
	11;0 - 11;11	69	0	00	0	0	00	0	0	—	—	—
	12;0 - 12;11	37	0	00	0	0	00	0	0	—	—	—
**Fronting other**	2;0 - 2;11	99	6	6.06	0	0	06	0	1	—	—	—
	3;0 - 3;11	107	8	7.48	0	0	08	0	2	—	—	—
	4;0 - 4;11	153	6	3.92	0	0	06	0	4	—	—	—
	5;0 - 5;11	137	2	1.46	0	0	01	0	1	—	—	—
	6;0 - 6;11	127	0	00	0	0	00	0	0	—	—	—
	7;0 - 7;11	138	0	00	0	0	00	0	0	—	—	—
	8;0 - 8;11	122	1	0.82	0	0	01	0	1	—	—	—
	9;0 - 9;11	105	0	00	0	0	00	0	0	—	—	—
	10;0 - 10;11	85	0	00	0	0	00	0	0	—	—	—
	11;0 - 11;11	69	0	00	0	0	00	0	0	—	—	—
	12;0 - 12;11	37	0	00	0	0	00	0	0	—	—	—

**Reduplication**	2;0 - 2;11	99	1	1.01	0	0	01	0	1	—	—	—
	3;0 - 3;11	107	0	00	0	0	00	0	0	—	—	—
	4;0 - 4;11	153	0	00	0	0	00	0	0	—	—	—
	5;0 - 5;11	137	0	00	0	0	00	0	0	—	—	—
	6;0 - 6;11	127	0	00	0	0	00	0	0	—	—	—
	7;0 - 7;11	138	0	00	0	0	00	0	0	—	—	—
	8;0 - 8;11	122	0	00	0	0	00	0	0	—	—	—
	9;0 - 9;11	105	0	00	0	0	00	0	0	—	—	—
	10;0 - 10;11	85	0	00	0	0	00	0	0	—	—	—
	11;0 - 11;11	69	0	00	0	0	00	0	0	—	—	—
	12;0 - 12;11	37	0	00	0	0	00	0	0	—	—	—

[a]child had history of unilateral cleft lip and palate with extensive use of backing; second highest number of errors was 16.

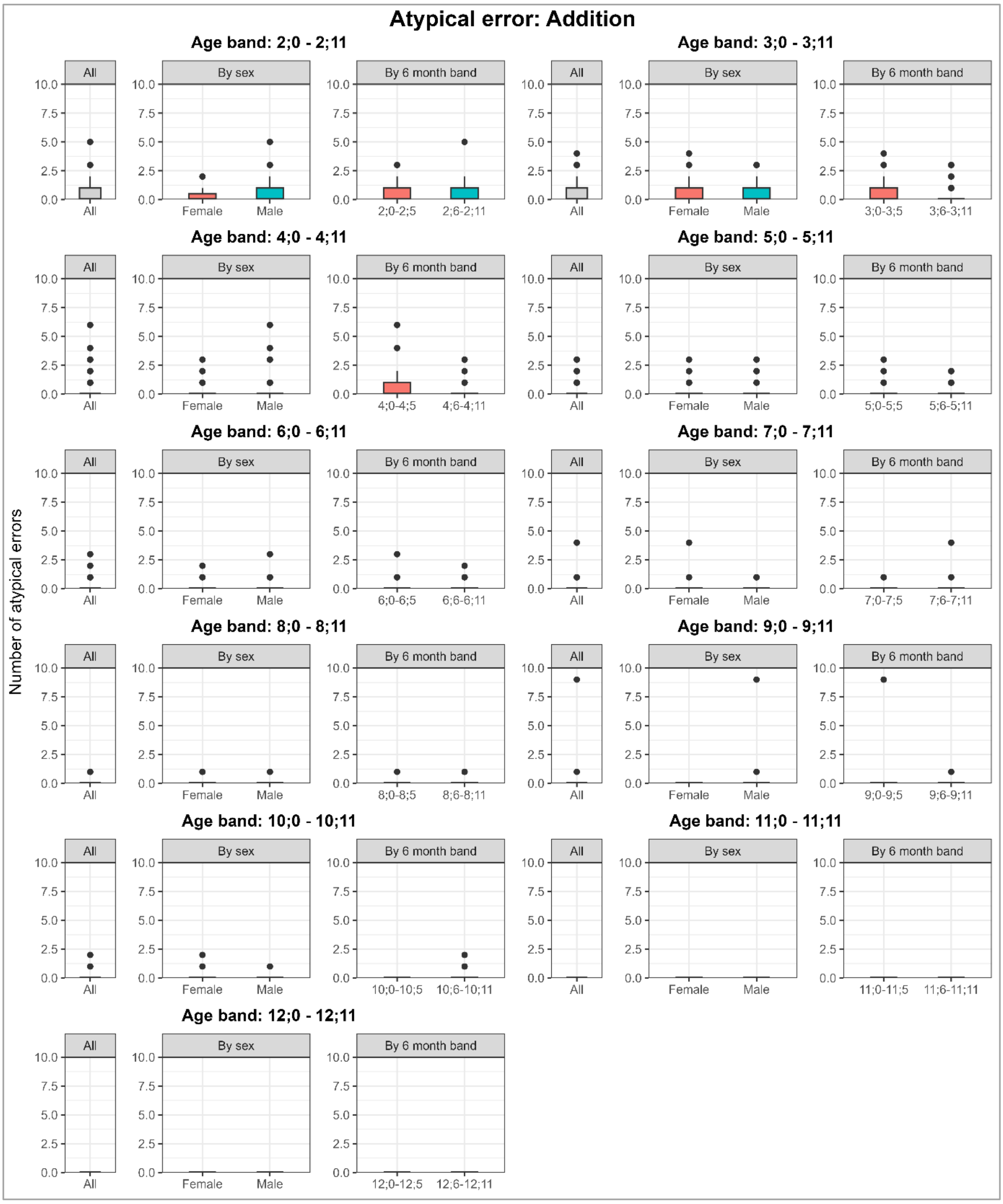

Figure S17. Frequency of addition errors stratified by age, sex, and six-month age band.

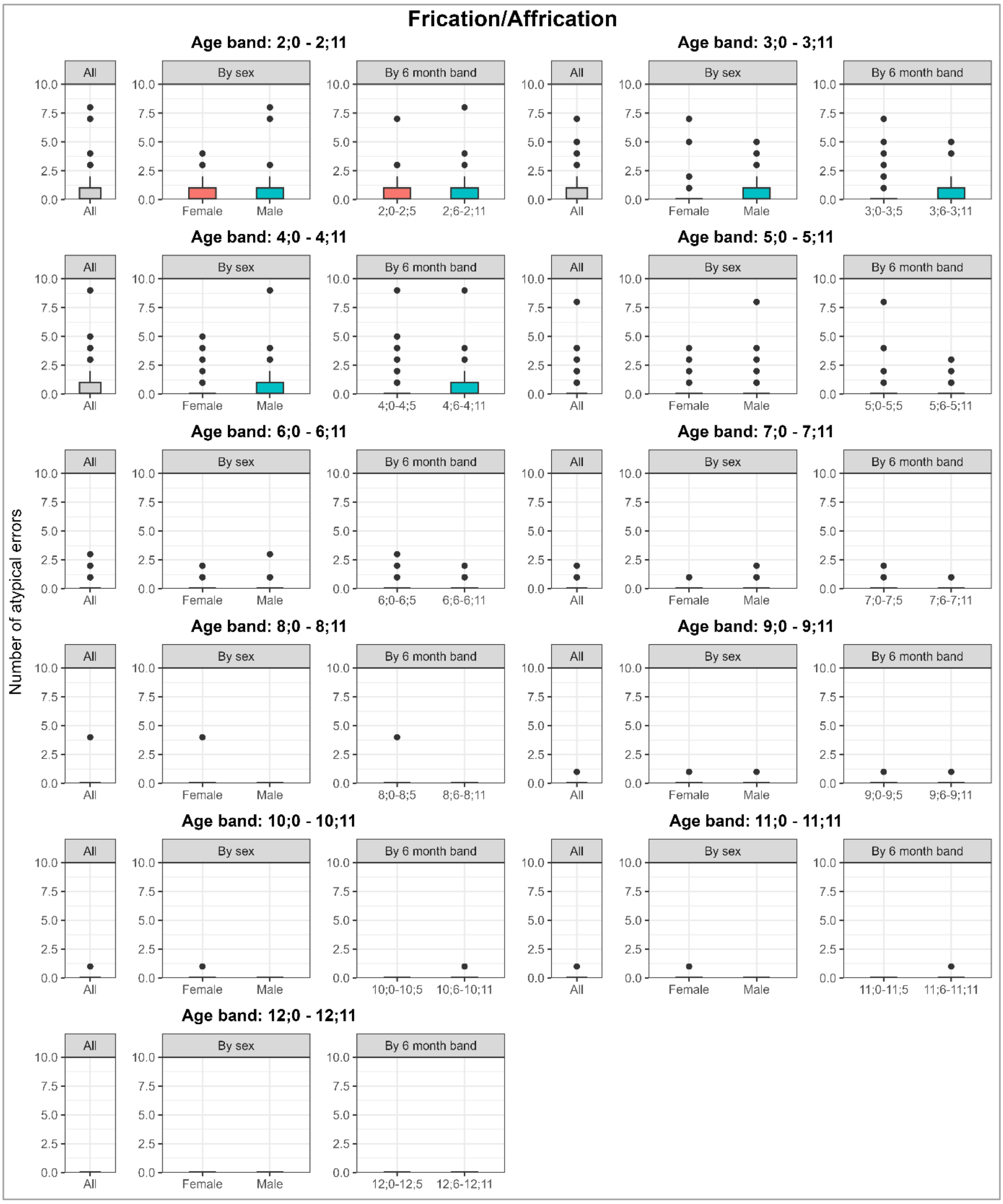

Figure S18. Frequency of frication/affrication errors stratified by age, sex, and six-month age band.

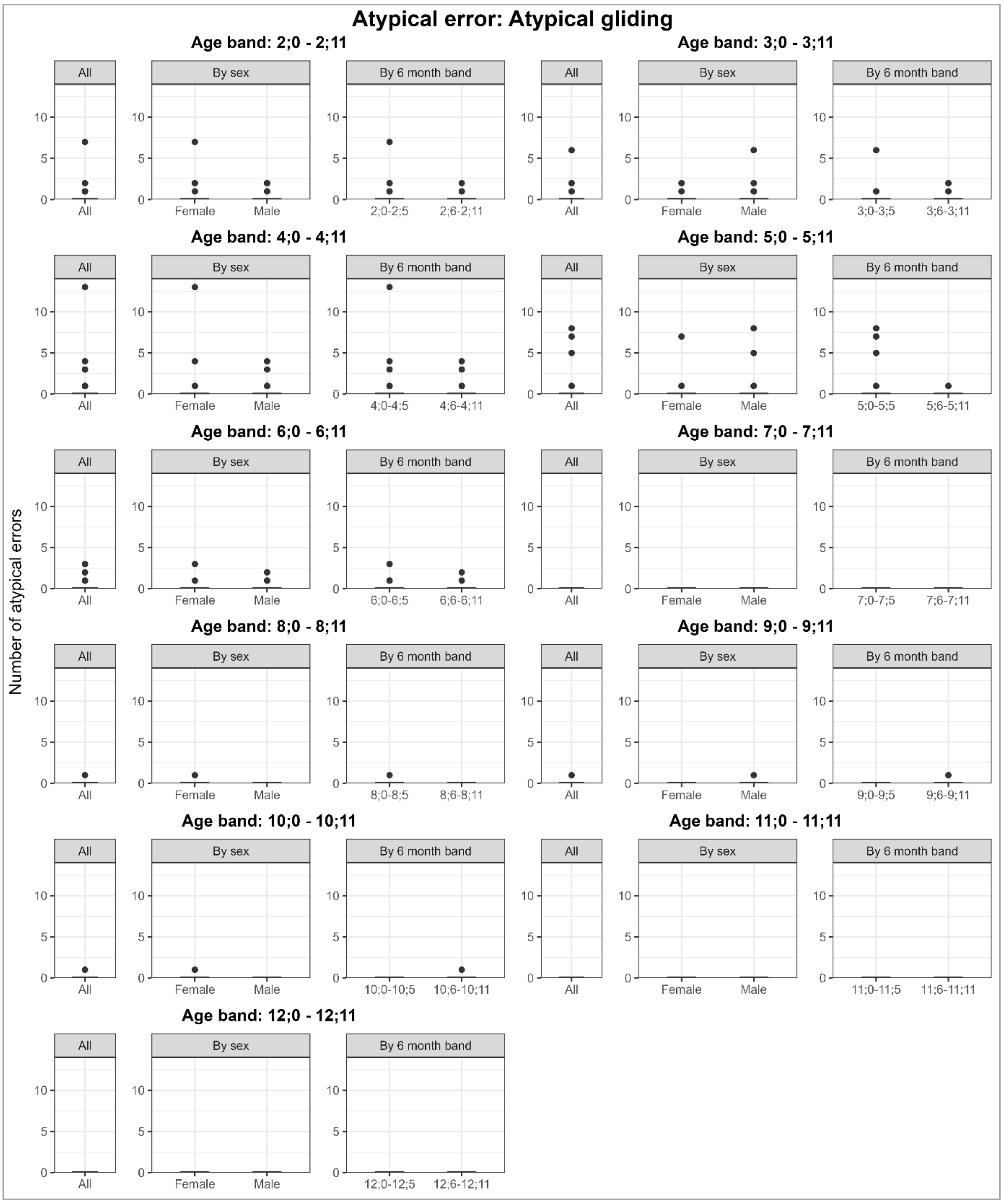

Figure S19. Frequency of atypical gliding errors stratified by age, sex, and six-month age band.

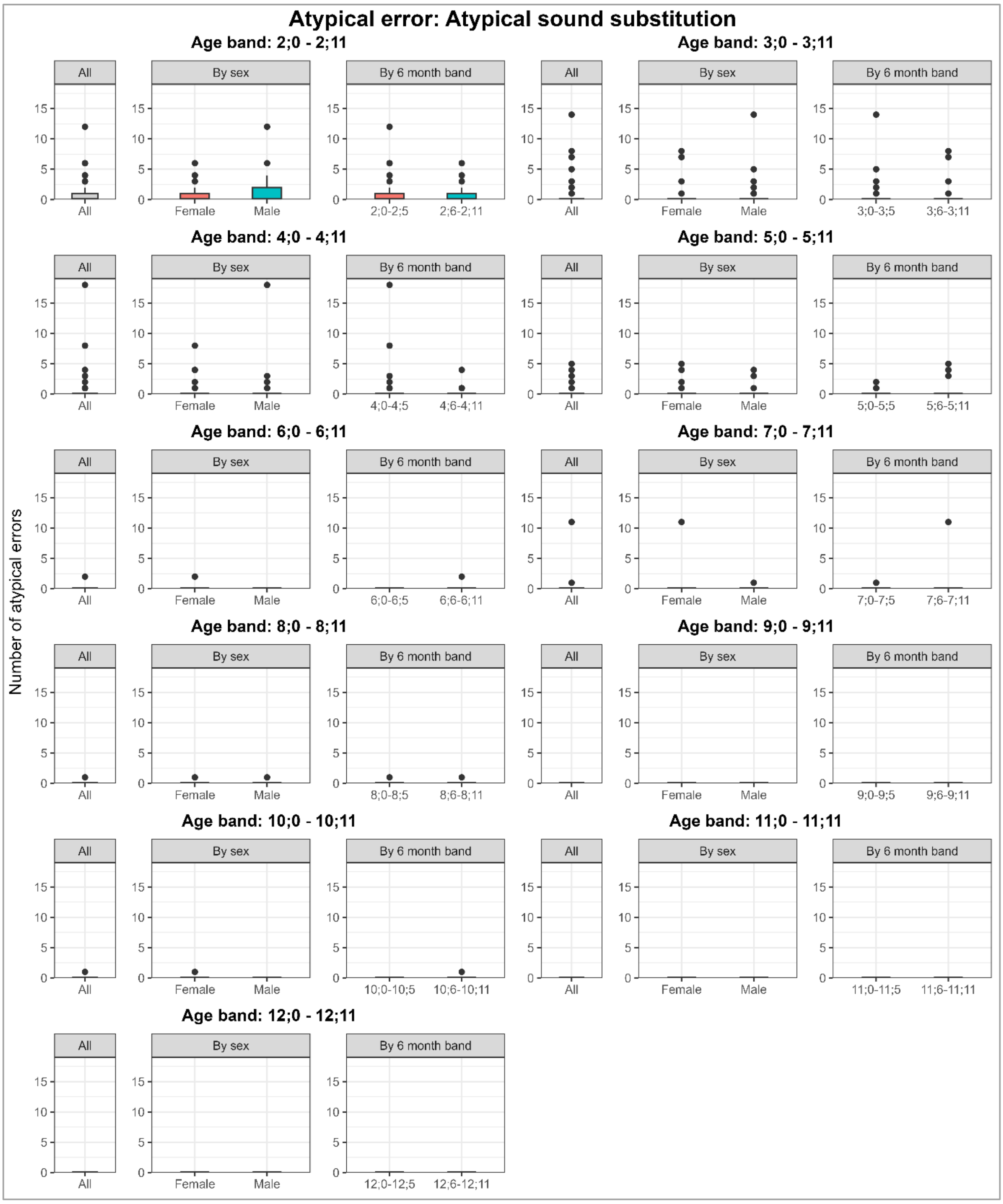

Figure S20. Frequency of atypical sound substitution errors stratified by age, sex, and six-month age band.

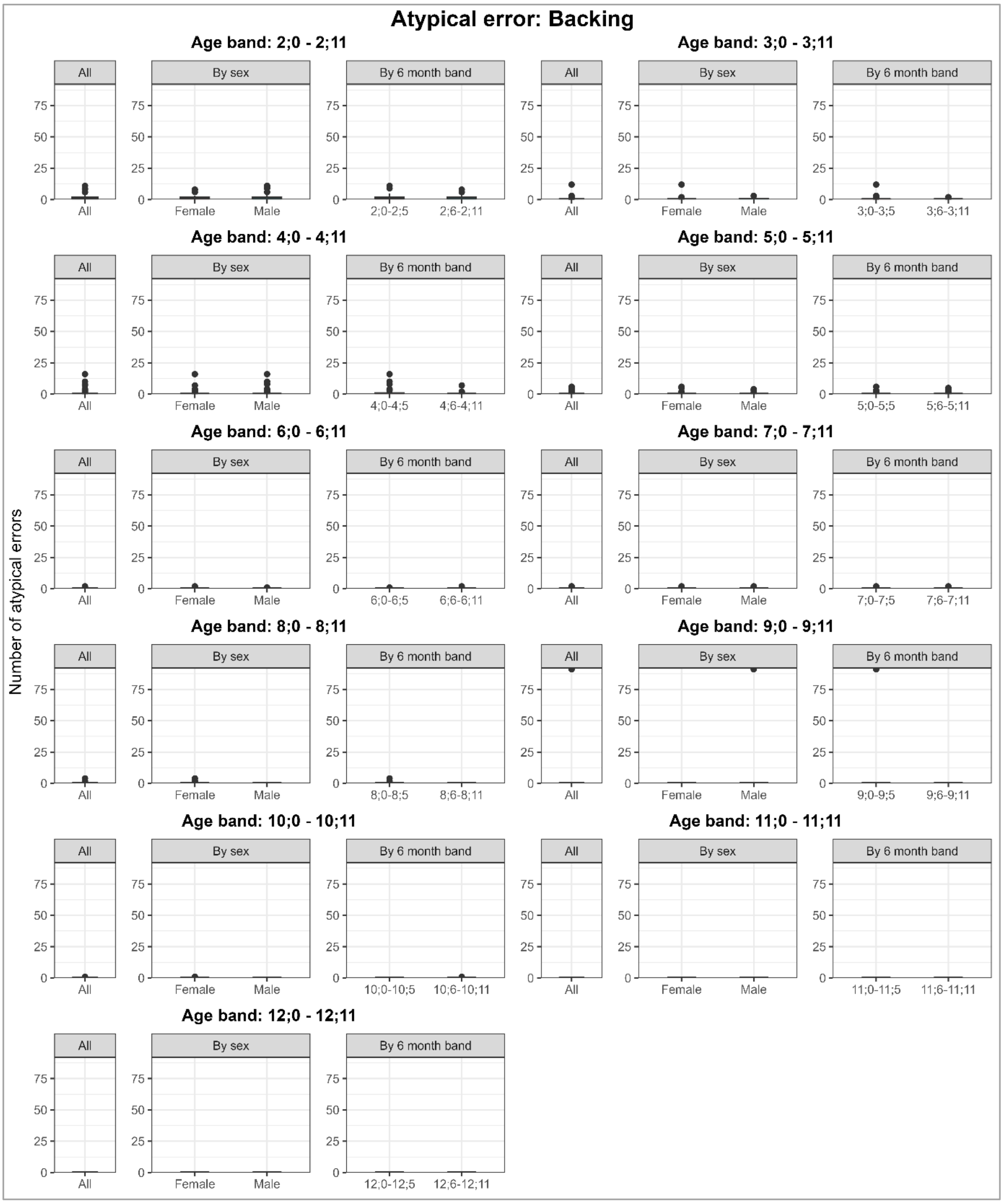

Figure S21. Frequency of backing errors stratified by age, sex, and six-month age band.

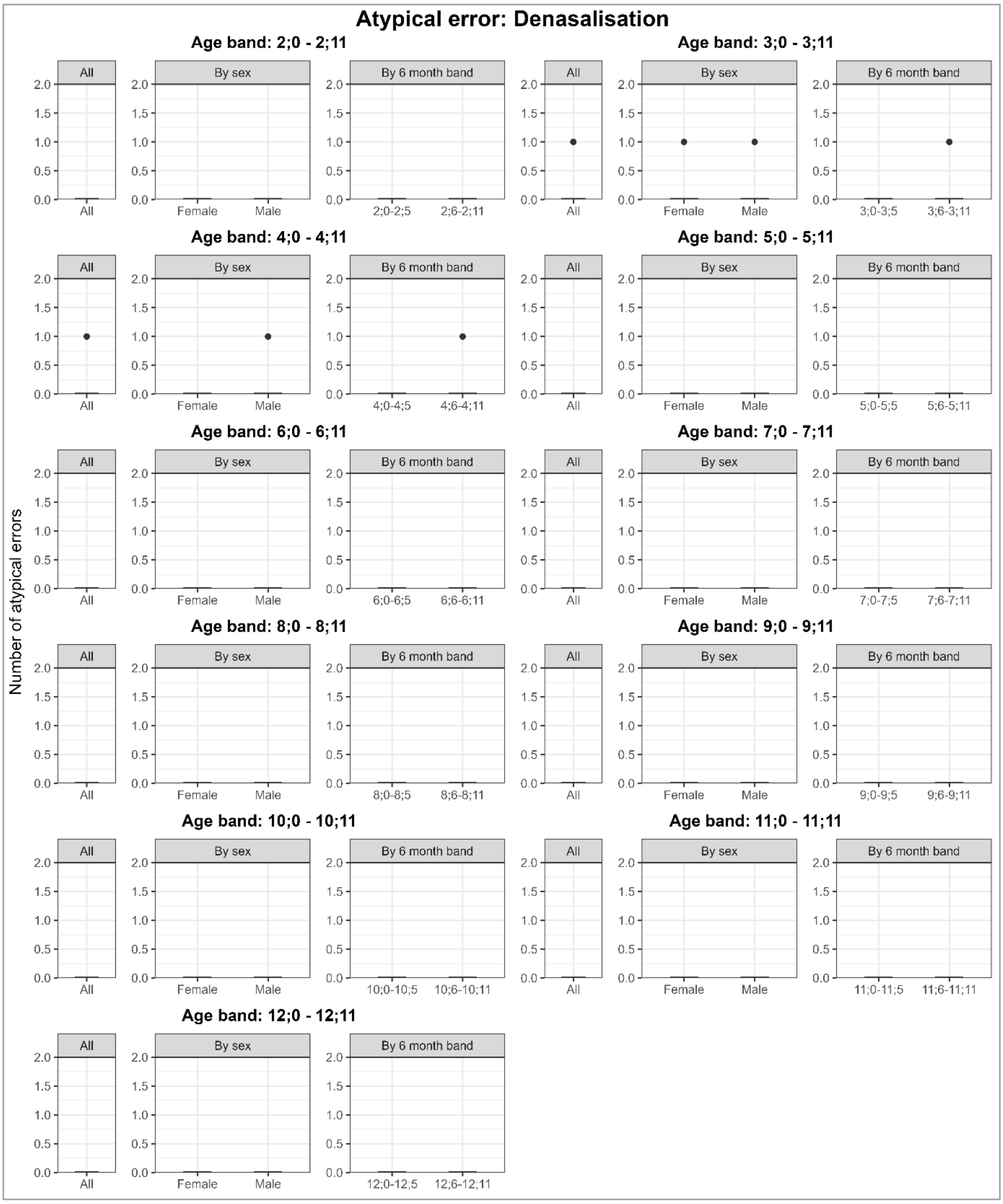

Figure S22. Frequency of denasalisation errors stratified by age, sex, and six-month age band.

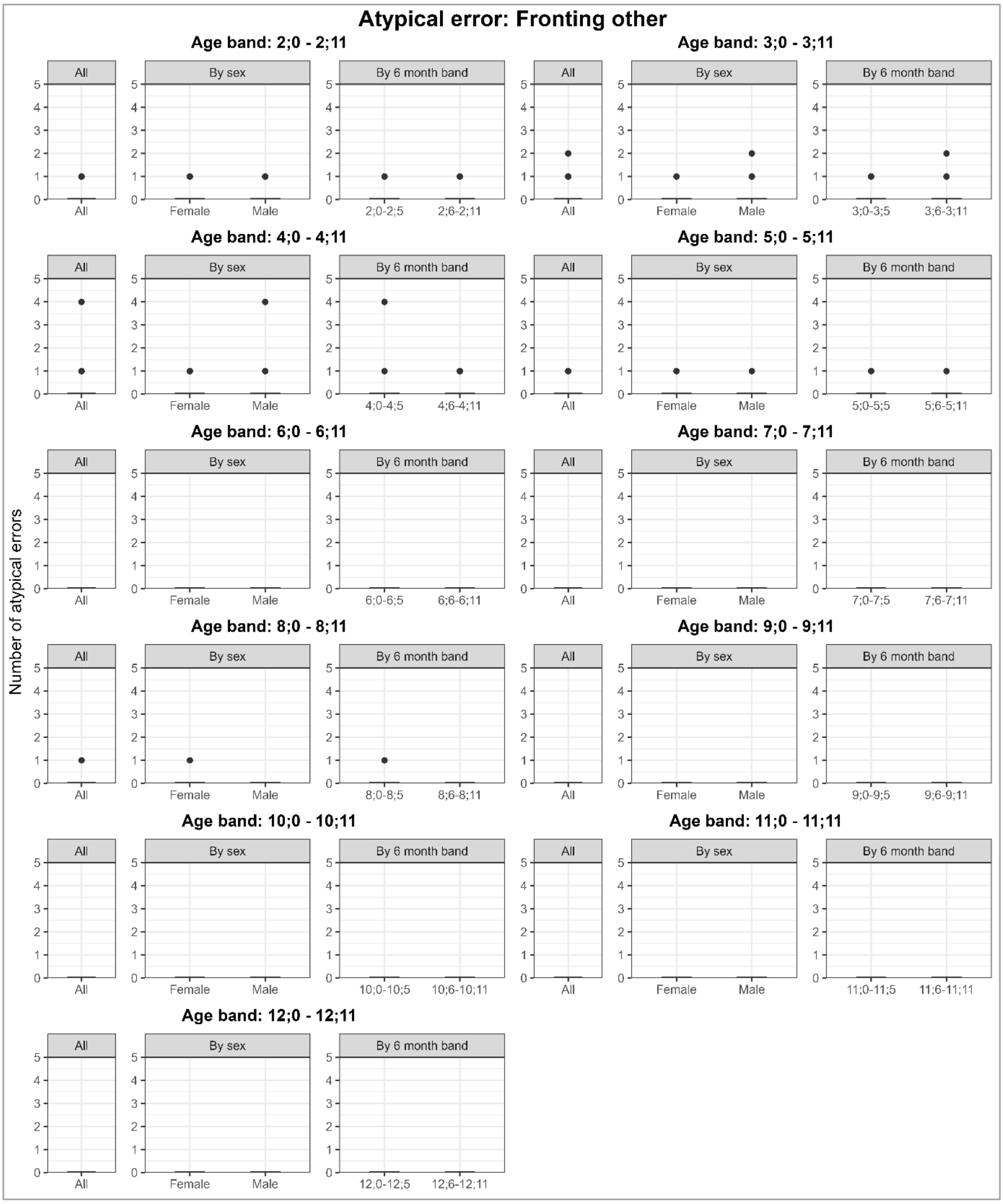

Figure S23. Frequency of fronting (other) errors stratified by age, sex, and six-month age band.

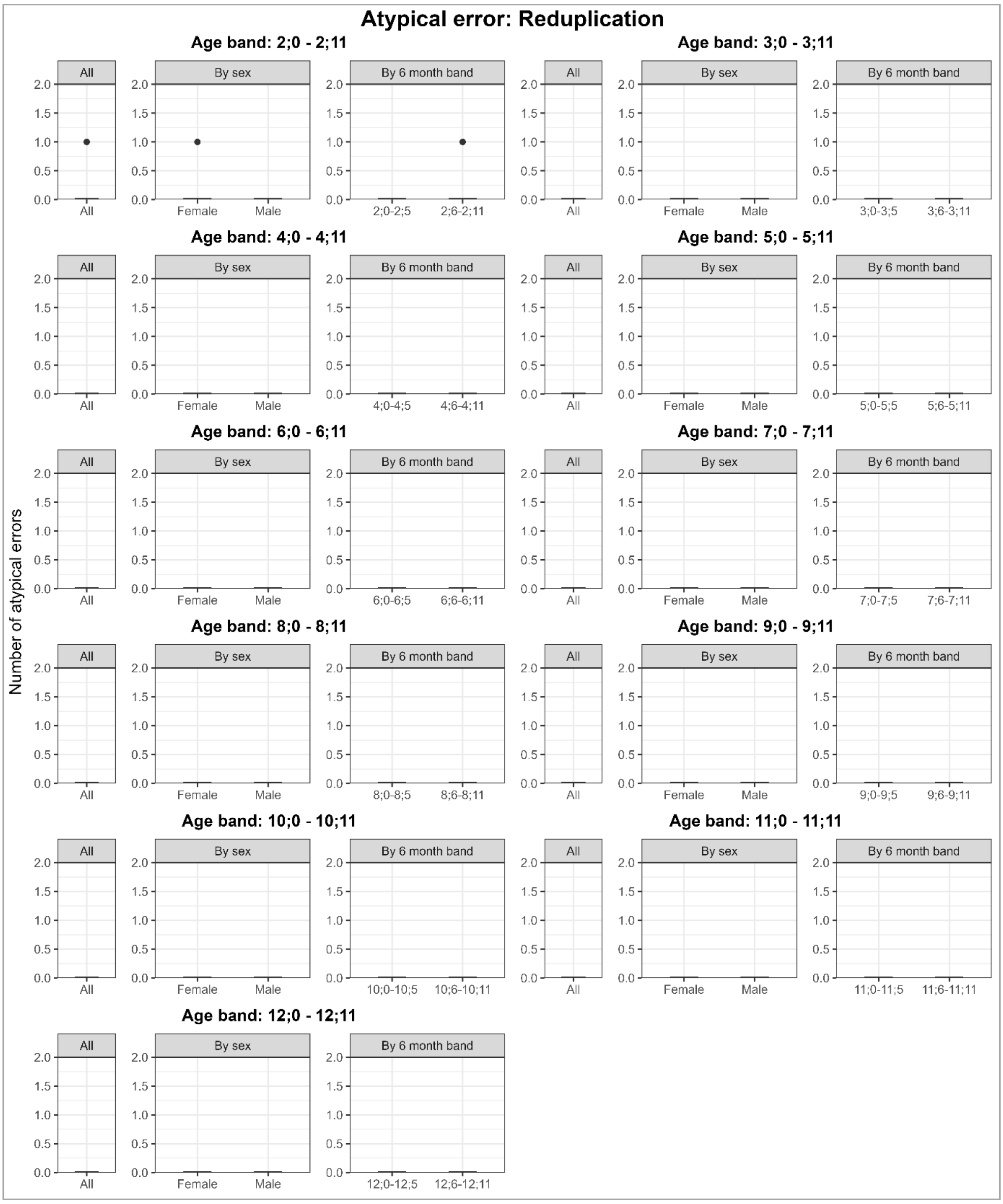

Figure S24. Frequency of reduplication errors stratified by age, sex, and six-month age band.

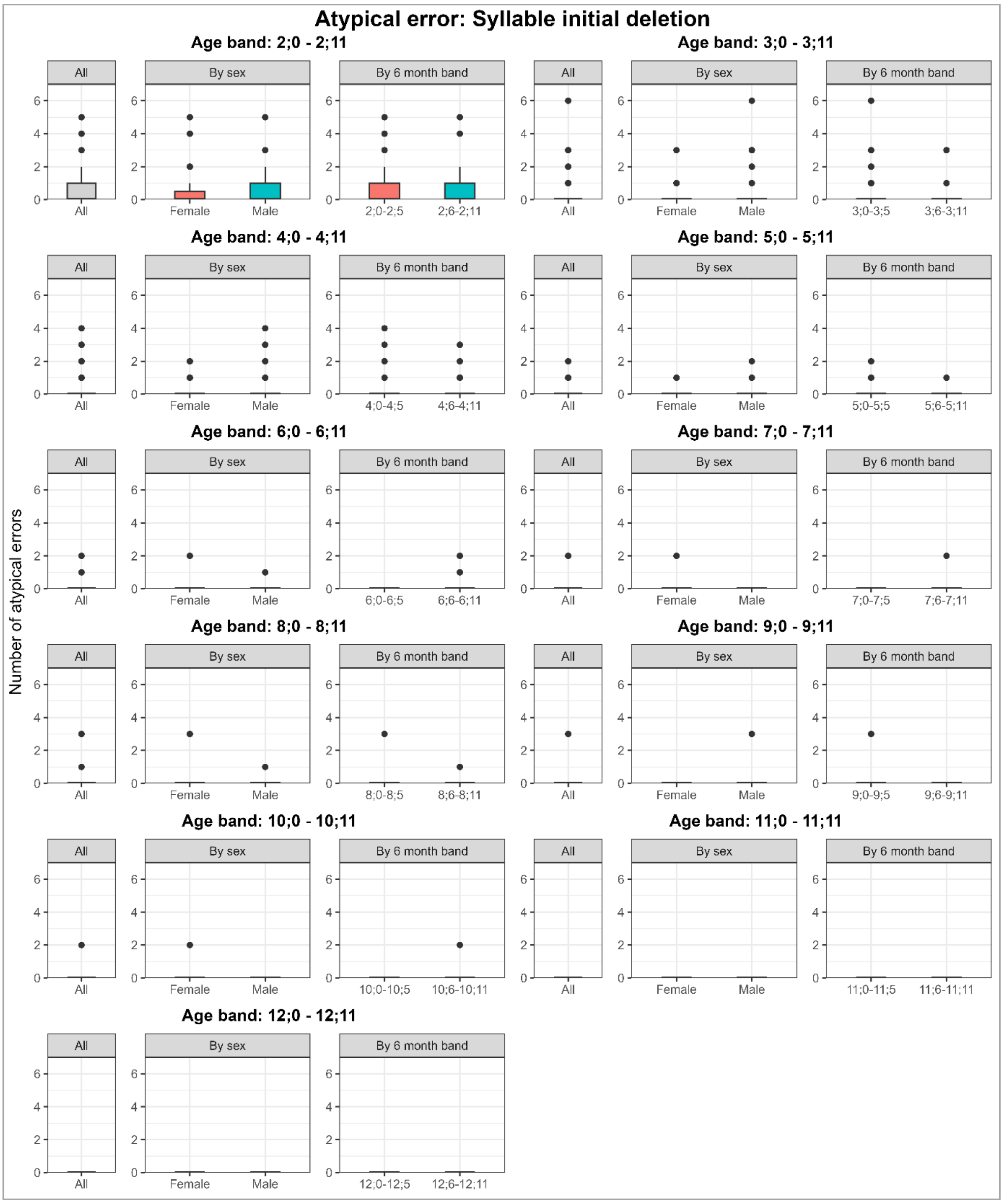

Figure S25. Frequency of syllable initial deletion errors stratified by age, sex, and six-month age band.

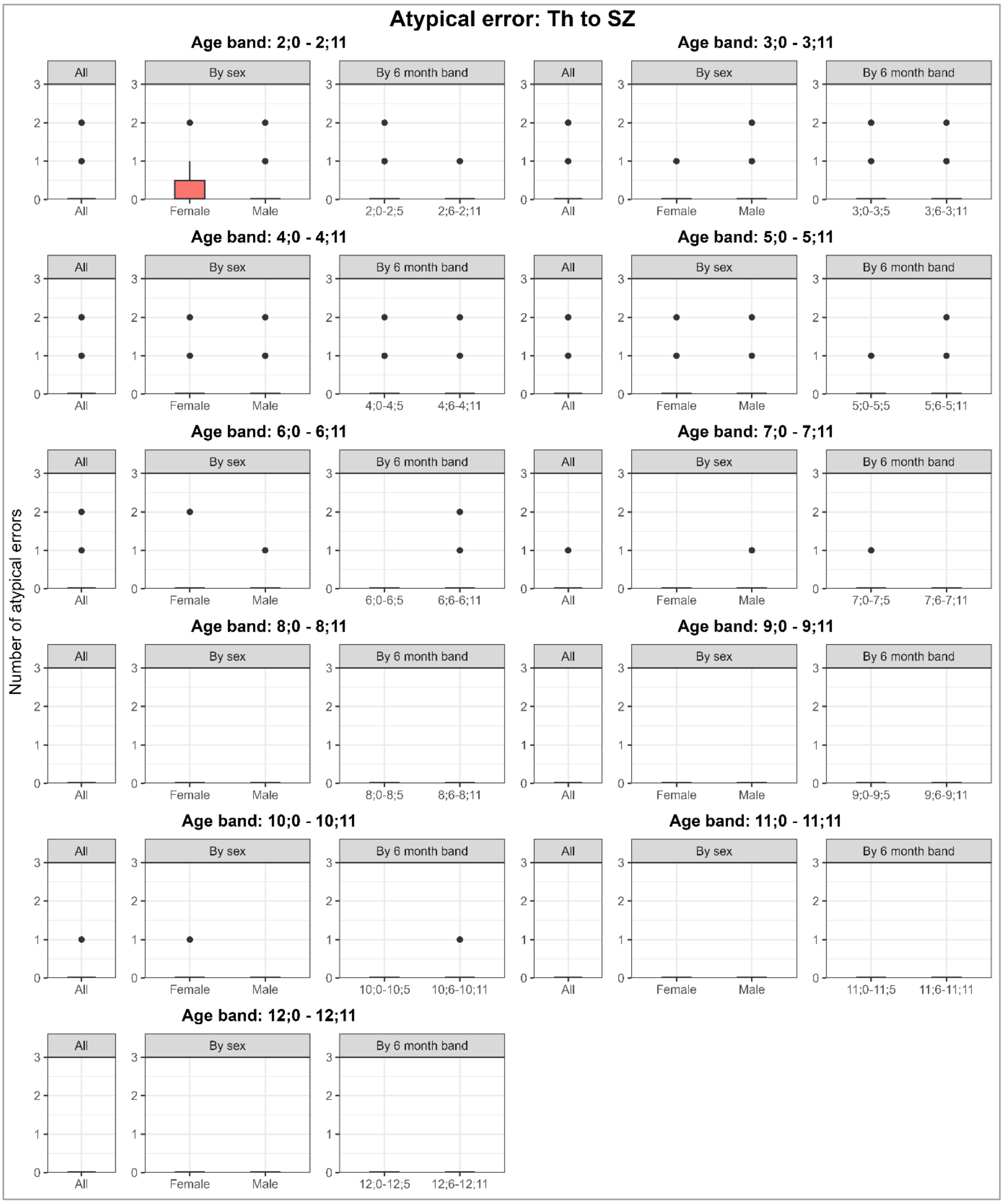

Figure S26. Frequency of Th to SZ errors stratified by age, sex, and six-month age band.

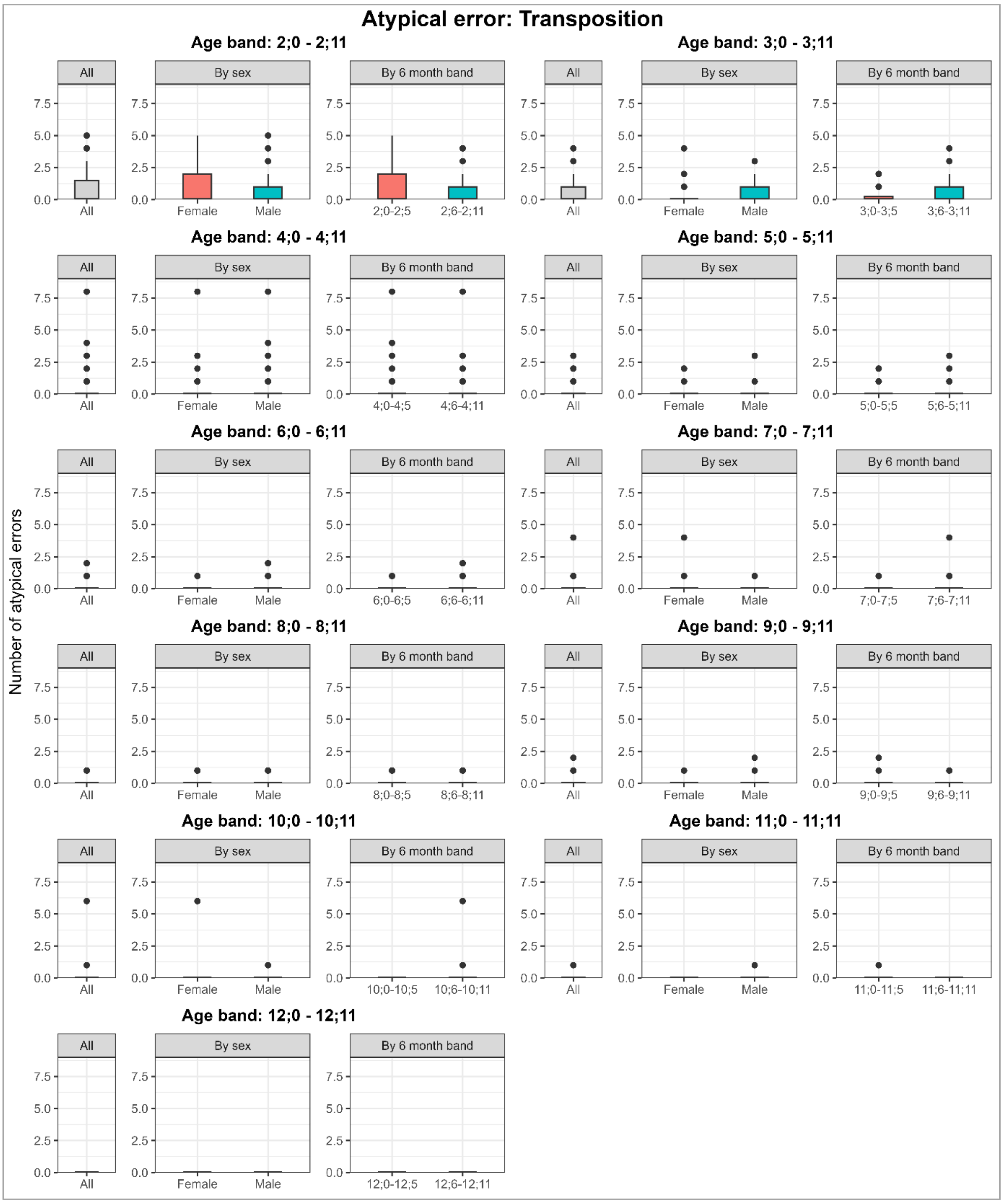

Figure S27. Frequency of transposition errors stratified by age, sex, and six-month age band.

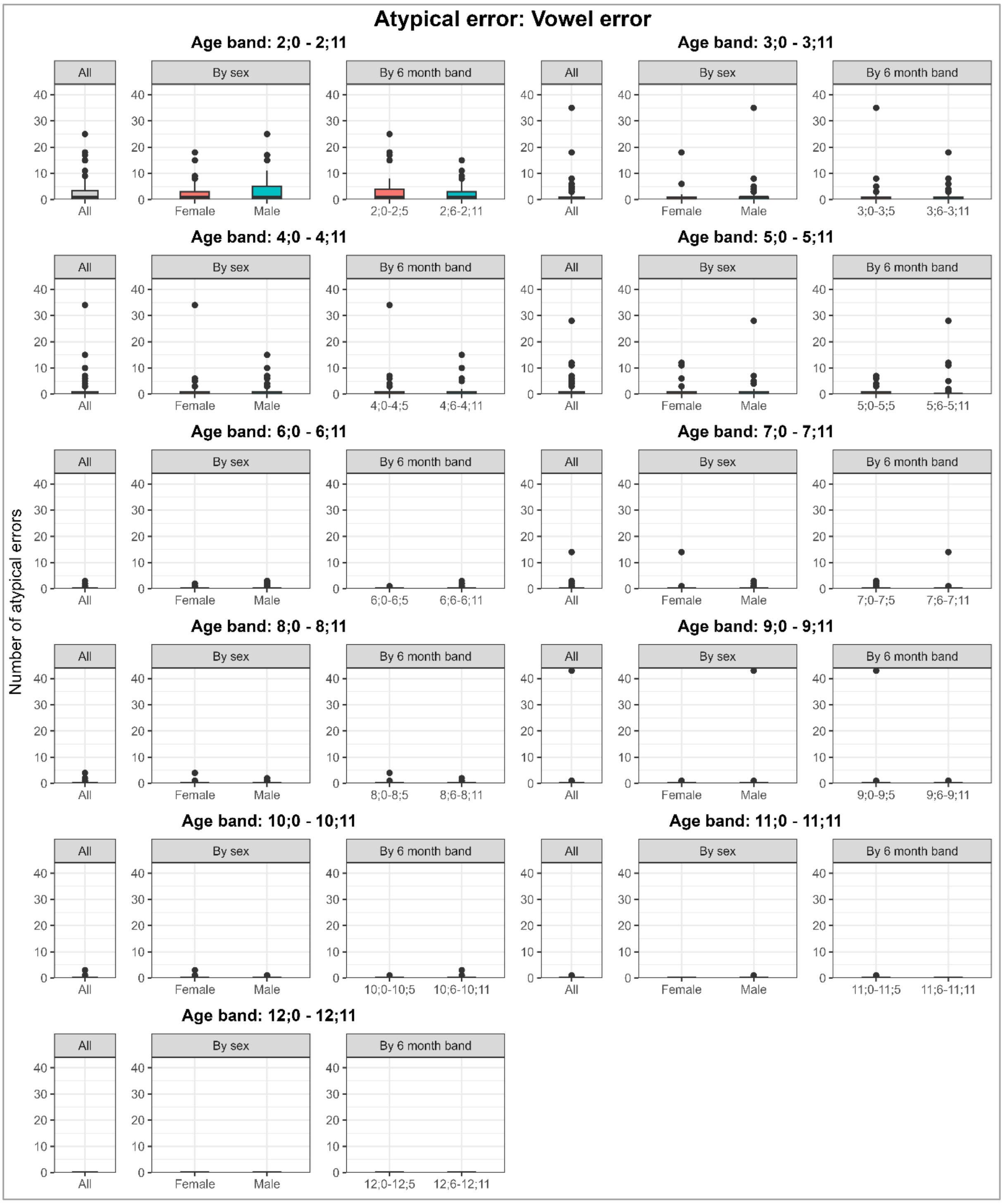

Figure S28. Frequency of vowel error errors stratified by age, sex, and six-month age band.

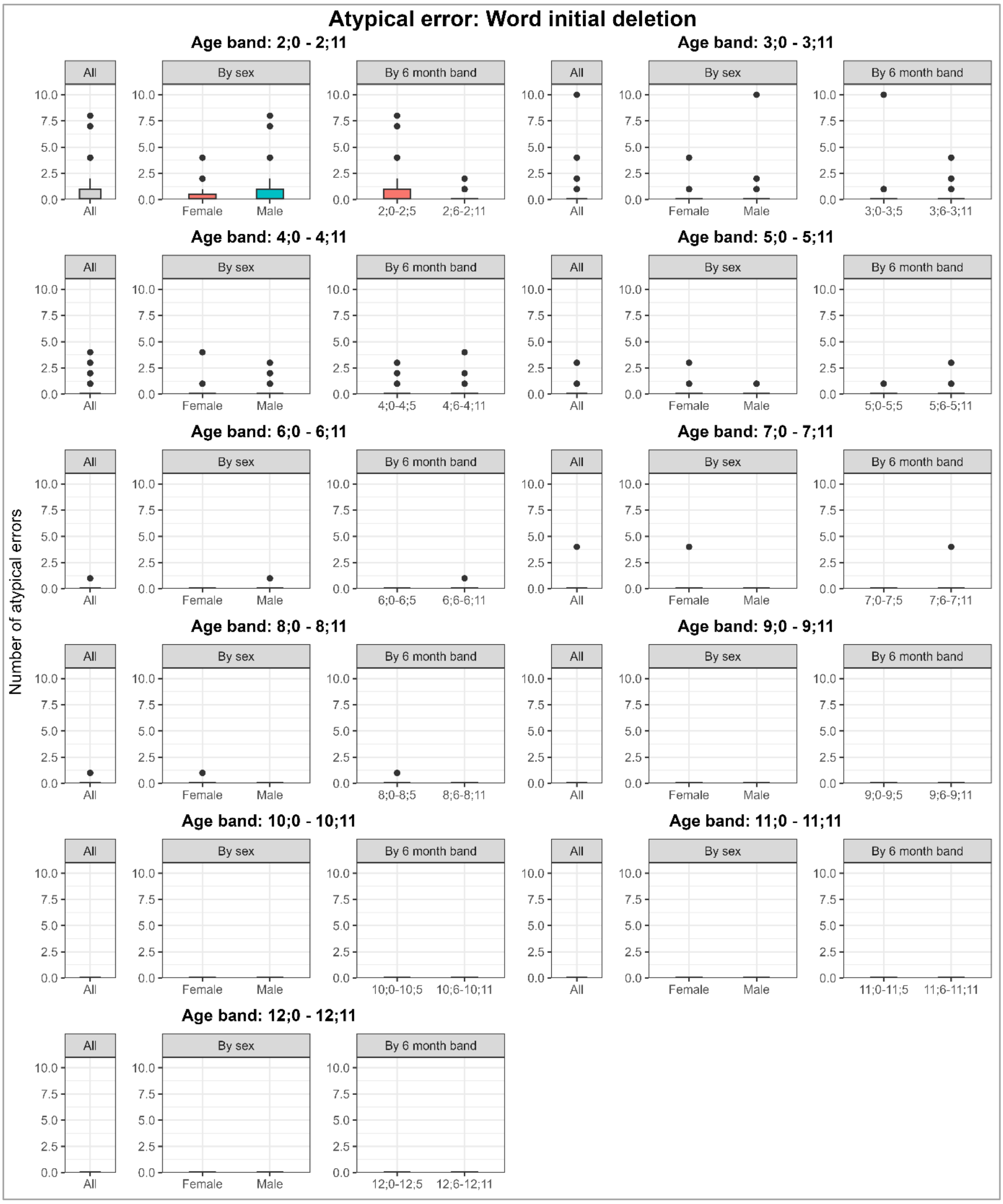

Figure S29. Frequency of word initial deletion errors stratified by age, sex, and six-month age band.

Table S19. Individual cut-points (stratified by disordered error pattern and 12-month age band) defined corresponding to the lowest performing 10% of the sample at that age (i.e., 90th percentile of number of errors). The cut-point represents the number of errors (i.e., X errors or more) that would classify the participant as falling into the lowest performing 10% of the sample.

**Disordered error pattern**	**Age group** (years; months)	**n**	**Cut-point: Number of errors (or more) to be in lowest performing 10% of sample**[a]
Addition	2;0 - 2;11	99	2
	3;0 - 3;11	107	2
	4;0 - 4;11	153	1
	5;0 - 5;11	137	1
	6;0 - 6;11	127	0
	7;0 - 7;11	138	0
	8;0 - 8;11	122	0
	9;0 - 9;11	105	0
	10;0 - 10;11	85	0
	11;0 - 11;11	69	0
	12;0 - 12;11	37	0
Frication/ affrication	2;0 - 2;11	99	2
	3;0 - 3;11	107	2
	4;0 - 4;11	153	2
	5;0 - 5;11	137	1
	6;0 - 6;11	127	1
	7;0 - 7;11	138	0
	8;0 - 8;11	122	0
	9;0 - 9;11	105	0
	10;0 - 10;11	85	0
	11;0 - 11;11	69	0
	12;0 - 12;11	37	0
Atypical gliding	2;0 - 2;11	99	1
	3;0 - 3;11	107	1
	4;0 - 4;11	153	1
	5;0 - 5;11	137	0
	6;0 - 6;11	127	0
	7;0 - 7;11	138	0
	8;0 - 8;11	122	0
	9;0 - 9;11	105	0
	10;0 - 10;11	85	0
	11;0 - 11;11	69	0
	12;0 - 12;11	37	0
Atypical sound substitution	2;0 - 2;11	99	2.2
	3;0 - 3;11	107	1
	4;0 - 4;11	153	0
	5;0 - 5;11	137	0
	6;0 - 6;11	127	0
	7;0 - 7;11	138	0

**Disordered error pattern**	**Age group** (years; months)	**n**	**Cut-point: Number of errors (or more) to be in lowest performing 10% of sample[a]**
	8;0 - 8;11	122	0
	9;0 - 9;11	105	0
	10;0 - 10;11	85	0
	11;0 - 11;11	69	0
	12;0 - 12;11	37	0
Backing	2;0 - 2;11	99	3
	3;0 - 3;11	107	1
	4;0 - 4;11	153	2
	5;0 - 5;11	137	1
	6;0 - 6;11	127	0
	7;0 - 7;11	138	0
	8;0 - 8;11	122	0
	9;0 - 9;11	105	0
	10;0 - 10;11	85	0
	11;0 - 11;11	69	0
	12;0 - 12;11	37	0
Denasalisation	2;0 - 2;11	99	0
	3;0 - 3;11	107	0
	4;0 - 4;11	153	0
	5;0 - 5;11	137	0
	6;0 - 6;11	127	0
	7;0 - 7;11	138	0
	8;0 - 8;11	122	0
	9;0 - 9;11	105	0
	10;0 - 10;11	85	0
	11;0 - 11;11	69	0
	12;0 - 12;11	37	0
Fronting (other)	2;0 - 2;11	99	0
	3;0 - 3;11	107	0
	4;0 - 4;11	153	0
	5;0 - 5;11	137	0
	6;0 - 6;11	127	0
	7;0 - 7;11	138	0
	8;0 - 8;11	122	0
	9;0 - 9;11	105	0
	10;0 - 10;11	85	0
	11;0 - 11;11	69	0
	12;0 - 12;11	37	0
Reduplication	2;0 - 2;11	99	0
	3;0 - 3;11	107	0
	4;0 - 4;11	153	0
	5;0 - 5;11	137	0
	6;0 - 6;11	127	0
	7;0 - 7;11	138	0
	8;0 - 8;11	122	0

Disordered error pattern	Age group (years; months)	n	Cut-point: Number of errors (or more) to be in lowest performing 10% of sample[a]
	9;0 - 9;11	105	0
	10;0 - 10;11	85	0
	11;0 - 11;11	69	0
	12;0 - 12;11	37	0
Syllable initial deletion	2;0 - 2;11	99	2
	3;0 - 3;11	107	1
	4;0 - 4;11	153	0
	5;0 - 5;11	137	0
	6;0 - 6;11	127	0
	7;0 - 7;11	138	0
	8;0 - 8;11	122	0
	9;0 - 9;11	105	0
	10;0 - 10;11	85	0
	11;0 - 11;11	69	0
	12;0 - 12;11	37	0
Th to SZ	2;0 - 2;11	99	1
	3;0 - 3;11	107	1
	4;0 - 4;11	153	0
	5;0 - 5;11	137	0
	6;0 - 6;11	127	0
	7;0 - 7;11	138	0
	8;0 - 8;11	122	0
	9;0 - 9;11	105	0
	10;0 - 10;11	85	0
	11;0 - 11;11	69	0
	12;0 - 12;11	37	0
Transposition	2;0 - 2;11	99	3
	3;0 - 3;11	107	1
	4;0 - 4;11	153	1
	5;0 - 5;11	137	1
	6;0 - 6;11	127	0
	7;0 - 7;11	138	0
	8;0 - 8;11	122	0
	9;0 - 9;11	105	0
	10;0 - 10;11	85	0
	11;0 - 11;11	69	0
	12;0 - 12;11	37	0
Vowel error	2;0 - 2;11	99	8
	3;0 - 3;11	107	2.4
	4;0 - 4;11	153	2
	5;0 - 5;11	137	1
	6;0 - 6;11	127	1
	7;0 - 7;11	138	1
	8;0 - 8;11	122	0.9
	9;0 - 9;11	105	0

**Disordered error pattern**	**Age group**	**n**	**Cut-point: Number of errors (or more) to be in lowest performing 10% of sample**[a]
	(years; months)		
	10;0 - 10;11	85	0.6
	11;0 - 11;11	69	0
	12;0 - 12;11	37	0
Word initial deletion	2;0 - 2;11	99	2
	3;0 - 3;11	107	1
	4;0 - 4;11	153	0
	5;0 - 5;11	137	0
	6;0 - 6;11	127	0
	7;0 - 7;11	138	0
	8;0 - 8;11	122	0
	9;0 - 9;11	105	0
	10;0 - 10;11	85	0
	11;0 - 11;11	69	0
	12;0 - 12;11	37	0

[a]When 0 is presented as the cut point, any participants making at least one error would be considered as underperforming (even lower than 10% of the sample).

Comparison to previous normative data

Table S20. Percentage of participants with the sound present (with or without stimulus) broken down by 6-month age bands. The shaded cells correspond to when the percentage is equal to or greater than 90% (the threshold from Dodd et al. (2003) paper).

Age band	n	/p/	/b/	/t/	/d/	/k/	/g/	/m/	/n/	/ŋ/	/f/	/v/	/θ/	/ð/	/s/	/z/	/ʃ/	/ʧ/	/ʤ/	/l/	/ɹ/	/w/	/j/	/h/	/ʒ/
2;0 - 2;5	53	98.1	100	98.1	98.1	96.2	88.7	100	96.2	86.8	88.7	69.8	34.0	15.1	84.9	86.8	83.0	64.2	64.2	96.2	75.5	96.2	77.4	96.2	47.2
2;6 - 2;11	46	100	100	100	97.8	97.8	89.1	97.8	100	93.5	93.5	89.1	39.1	32.6	84.8	84.8	76.1	71.7	69.6	97.8	87.0	100	80.4	100	54.3
3;0 - 3;5	52	98.1	100	100	100	98.1	98.1	98.1	100	98.1	96.2	94.2	57.7	48.1	84.6	82.7	78.8	86.5	84.6	98.1	96.2	100	92.3	98.1	63.5
3;6 - 3;11	55	100	100	100	100	100	98.2	100	100	100	100	100	58.2	43.6	87.3	83.6	92.7	89.1	90.9	100	90.9	100	100	100	65.5
4;0 - 4;5	77	100	100	100	100	100	100	100	100	100	98.7	92.2	59.7	51.9	81.8	79.2	92.2	92.2	87.0	100	90.9	100	100	98.7	75.3
4;6 - 4;11	76	100	100	100	100	100	100	100	100	98.7	100	98.7	63.2	51.3	84.2	78.9	94.7	96.1	93.4	100	93.4	100	98.7	100	80.3
5;0 - 5;5	61	100	100	100	100	100	98.4	100	100	98.4	98.4	98.4	75.4	78.7	90.2	88.5	95.1	93.4	91.8	100	88.5	100	98.4	100	83.6
5;6 - 5;11	76	100	100	100	100	100	100	100	100	97.4	100	98.7	88.2	88.2	84.2	85.5	97.4	94.7	94.7	98.7	92.1	100	100	100	85.5
6;0 - 6;5	33	100	100	100	100	100	100	100	100	97.0	100	100	97.0	97.0	93.9	93.9	100	100	100	100	90.9	100	100	100	90.9
6;6 - 6;11	94	100	100	100	100	100	100	100	100	100	100	100	88.3	87.2	98.9	97.9	97.9	100	100	100	97.9	100	100	100	87.2
7;0 - 7;5	72	100	100	100	100	100	100	100	100	100	100	100	98.6	97.2	86.1	84.7	98.6	97.2	97.2	100	98.6	100	100	100	93.1
7;6 - 7;11	66	100	100	100	100	100	100	100	100	98.5	98.5	98.5	98.5	98.5	98.5	97.0	95.5	97.0	95.5	100	98.5	100	100	100	89.4
8;0 - 8;5	62	100	100	100	100	100	100	100	100	100	100	100	98.4	100	93.5	93.5	98.4	98.4	98.4	100	98.4	100	100	100	95.2
8;6 - 8;11	60	100	100	100	100	100	100	100	100	100	100	100	95.0	95.0	98.3	96.7	98.3	100	100	100	96.7	100	100	100	98.3
9;0 - 9;5	51	100	100	98.0	98.0	100	100	100	100	100	100	100	100	100	94.1	94.1	98.0	98.0	96.1	98.0	100	100	100	100	94.1
9;6 - 9;11	54	100	100	100	100	100	100	100	100	100	100	100	98.1	100	98.1	98.1	100	100	100	100	98.1	100	100	100	100
10;0 - 10;5	41	100	100	100	100	100	100	100	100	100	100	100	100	97.6	97.6	97.6	100	100	100	100	100	100	100	100	100
10;6 - 10;11	44	100	100	100	100	100	100	100	100	100	100	100	93.2	93.2	97.7	97.7	97.7	100	100	100	100	100	100	100	95.5
11;0 - 11;5	39	100	100	100	100	100	100	100	100	100	100	100	100	100	97.4	97.4	100	97.4	97.4	100	97.4	100	100	100	100
11;6 - 11;11	30	100	100	100	100	100	100	100	100	100	100	100	100	96.7	96.7	96.7	100	100	100	100	96.7	100	100	100	100
12;0 - 12;5	30	100	100	100	100	100	100	100	100	100	100	100	100	100	100	100	96.7	96.7	96.7	100	96.7	100	100	100	96.7
12;6 - 12;11	7	100	100	100	100	100	100	100	100	100	100	100	85.7	85.7	71.4	57.1	100	100	100	100	100	100	100	100	100

Table S21. Phonetic acquisition from our study compared to the previous study by Dodd et al. (2003). Under this threshold, a sound was considered present if 90% or more of that age band could produce the sound with or without stimulus. Plosive speech sounds are produced by using the lips, teeth, or palate to stop airflow before a sudden release of air. Nasal sounds are produced when air escapes via the nose rather than mouth. Fricative sounds are produced by releasing air through two articulators close together (e.g. upper teeth on lower lip to produce /f/). Affricates combine a plosive feature to start before releasing a fricative feature. Approximant sounds are produced by two articulators coming together but not as narrowly as fricatives, without turbulent airflow.

**Age band (years; months)**		**ADAAPT**		**DEAP**	
		**Present**	**Absent**	**Present**	**Absent**
3;0 – 3;5	Plosive	/p/, /b/, /t/, /d/, /k/, /g/		/p/, /b/, /t/, /d/, /k/, /g/	
	Nasal	/m/, /n/, /ŋ/		/m/, /n/, /ŋ/	
	Fricative	/f/, /v/, /h/	/θ/, /ð/, /s/, /z/, /ʃ/, /ʒ/	/f/, /v/, /s/, /z/, /h/	/θ/, /ð/, /ʃ/, /ʒ/
	Affricative		/ʧ/, /ʤ/		/ʧ/, /ʤ/
	Approximant	/w/, /l/, /j/, /ɹ/		w, l-, j	/ɹ/
3;6 – 3;11	Plosive				
	Nasal				
	Fricative	/ʃ/	/θ/, /ð/, /s/, /z/, /ʒ/		/θ/, /ð/, /ʃ/, /ʒ/
	Affricate	/ʤ/	/ʧ/	/ʧ/	/ʤ/
	Approximant				/ɹ/
4;0 – 4;5	Plosive				
	Nasal				
	Fricative		/θ/, /ð/, /s/, /z/, /ʒ/	/ʒ/	/θ/, /ð/, /ʃ/,
	Affricate	/ʧ/	/ʤ/	/ʤ/	
	Approximant				/ɹ/
4;6 – 4;11	Plosive				
	Nasal				
	Fricative		/θ/, /ð/, /s/, /z/, /ʒ/		/θ/, /ð/, /ʃ/,
	Affricate	/ʤ/			
	Approximant				/ɹ/
5;0 – 5;5	Plosive				
	Nasal				
	Fricative	/s/	/θ/, /ð/, /z/, /ʒ/	/ʃ/	/θ/, /ð/
	Affricate				
	Approximant		/ɹ/*		/ɹ/
5;6 – 5;11	Plosive				
	Nasal				
	Fricative		/θ/, /ð/, /s/, /z/, /ʒ/		/θ/, /ð/
	Affricate				
	Approximant	/ɹ/			/ɹ/
6;0 – 6;5	Plosive				
	Nasal				
	Fricative	/θ/, /ð/, /s/, /z/, /ʒ/			/θ/, /ð/
	Affricate				
	Approximant			/ɹ/	
6;6 – 6;11	Plosive				
	Nasal				
	Fricative		/θ/, /ð/, /ʒ/		/θ/, /ð/

	Affricate			
	Approximant			
7;0 above	Plosive			
	Nasal			
	Fricative	/θ/, /ð/, /ʒ/	/s/*, /z/*	/θ/, /ð/
	Affricate			
	Approximant			

*Although absent, was still present for ≥80% participants (see Table S9).

Table S22. Proportion of participants with sounds not present (not stimulable or no response) stratified by age band (6 months) alongside the speech pathology history.

Sound	Age band	n	Not stimulable or no response		Ever seen a speech pathologist (%)		
			n	%	Yes	No	Missing
/θ/	2;0-2;5	53	35	66.0	14.3	82.9	2.9
	2;6-2;11	46	28	60.9	7.1	89.3	3.6
	3;0-3;5	52	22	42.3	0.0	63.6	36.4
	3;6-3;11	55	23	41.8	0.0	43.5	56.5
	4;0-4;5	77	31	40.3	12.9	41.9	45.2
	4;6-4;11	76	28	36.8	14.3	28.6	57.1
	5;0-5;5	61	15	24.6	6.7	20.0	73.3
	5;6-5;11	76	9	11.8	22.2	44.4	33.3
	6;0-6;5	33	1	3.0	0.0	100.0	0.0
	6;6-6;11	94	11	11.7	9.1	36.4	54.5
	7;0-7;5	72	1	1.4	100.0	0.0	0.0
	7;6-7;11	66	1	1.5	100.0	0.0	0.0
	8;0-8;5	62	1	1.6	0.0	100.0	0.0
	8;6-8;11	60	3	5.0	66.7	33.3	0.0
	9;0-9;5	51	0	0.0	—	—	—
	9;6-9;11	54	1	1.9	0.0	100.0	0.0
	10;0-10;5	41	0	0.0	—	—	—
	10;6-10;11	44	3	6.8	66.7	33.3	0.0
	11;0-11;5	39	0	0.0	—	—	—
	11;6-11;11	30	0	0.0	—	—	—
	12;0-12;5	30	0	0.0	—	—	—
	12;6-12;11	7	1	14.3	100.0	0.0	0.0
/ð/	2;0-2;5	53	45	84.9	11.1	82.2	6.7
	2;6-2;11	46	31	67.4	9.7	83.9	6.5
	3;0-3;5	52	27	51.9	0.0	66.7	33.3
	3;6-3;11	55	31	56.4	0.0	48.4	51.6
	4;0-4;5	77	37	48.1	16.2	40.5	43.2

Sound	Age band	n	Not stimulable or no response		Ever seen a speech pathologist (%)		
			n	%	Yes	No	Missing
	4;6-4;11	76	37	48.7	21.6	32.4	45.9
	5;0-5;5	61	13	21.3	15.4	23.1	61.5
	5;6-5;11	76	9	11.8	33.3	33.3	33.3
	6;0-6;5	33	1	3.0	100.0	0.0	0.0
	6;6-6;11	94	12	12.8	8.3	50.0	41.7
	7;0-7;5	72	2	2.8	50.0	50.0	0.0
	7;6-7;11	66	1	1.5	100.0	0.0	0.0
	8;0-8;5	62	0	0.0	—	—	—
	8;6-8;11	60	3	5.0	66.7	33.3	0.0
	9;0-9;5	51	0	0.0	—	—	—
	9;6-9;11	54	0	0.0	—	—	—
	10;0-10;5	41	1	2.4	0.0	100.0	0.0
	10;6-10;11	44	3	6.8	66.7	33.3	0.0
	11;0-11;5	39	0	0.0	—	—	—
	11;6-11;11	30	1	3.3	100.0	0.0	0.0
	12;0-12;5	30	0	0.0	—	—	—
	12;6-12;11	7	1	14.3	100.0	0.0	0.0
**/s/**	2;0-2;5	53	8	15.1	0.0	100.0	0.0
	2;6-2;11	46	7	15.2	0.0	100.0	0.0
	3;0-3;5	52	8	15.4	0.0	87.5	12.5
	3;6-3;11	55	7	12.7	0.0	71.4	28.6
	4;0-4;5	77	14	18.2	21.4	42.9	35.7
	4;6-4;11	76	12	15.8	8.3	75.0	16.7
	5;0-5;5	61	6	9.8	16.7	33.3	50.0
	5;6-5;11	76	12	15.8	33.3	58.3	8.3
	6;0-6;5	33	2	6.1	50.0	50.0	0.0
	6;6-6;11	94	1	1.1	0.0	100.0	0.0
	7;0-7;5	72	10	13.9	30.0	50.0	20.0
	7;6-7;11	66	1	1.5	100.0	0.0	0.0
	8;0-8;5	62	4	6.5	50.0	25.0	25.0
	8;6-8;11	60	1	1.7	100.0	0.0	0.0
	9;0-9;5	51	3	5.9	33.3	33.3	33.3

Sound	Age band	n	Not stimulable or no response		Ever seen a speech pathologist (%)		
			n	%	Yes	No	Missing
	9;6-9;11	54	1	1.9	100.0	0.0	0.0
	10;0-10;5	41	1	2.4	0.0	100.0	0.0
	10;6-10;11	44	1	2.3	100.0	0.0	0.0
	11;0-11;5	39	1	2.6	100.0	0.0	0.0
	11;6-11;11	30	1	3.3	0.0	100.0	0.0
	12;0-12;5	30	0	0.0	—	—	—
	12;6-12;11	7	2	28.6	0.0	100.0	0.0
**/z/**	2;0-2;5	53	7	13.2	14.3	85.7	0.0
	2;6-2;11	46	7	15.2	0.0	100.0	0.0
	3;0-3;5	52	9	17.3	0.0	88.9	11.1
	3;6-3;11	55	9	16.4	11.1	66.7	22.2
	4;0-4;5	77	16	20.8	31.2	37.5	31.2
	4;6-4;11	76	16	21.1	18.8	68.8	12.5
	5;0-5;5	61	7	11.5	14.3	42.9	42.9
	5;6-5;11	76	11	14.5	36.4	54.5	9.1
	6;0-6;5	33	2	6.1	50.0	50.0	0.0
	6;6-6;11	94	2	2.1	0.0	100.0	0.0
	7;0-7;5	72	11	15.3	27.3	54.5	18.2
	7;6-7;11	66	2	3.0	50.0	50.0	0.0
	8;0-8;5	62	4	6.5	50.0	25.0	25.0
	8;6-8;11	60	2	3.3	50.0	50.0	0.0
	9;0-9;5	51	3	5.9	66.7	0.0	33.3
	9;6-9;11	54	1	1.9	100.0	0.0	0.0
	10;0-10;5	41	1	2.4	0.0	100.0	0.0
	10;6-10;11	44	1	2.3	100.0	0.0	0.0
	11;0-11;5	39	1	2.6	100.0	0.0	0.0
	11;6-11;11	30	1	3.3	0.0	100.0	0.0
	12;0-12;5	30	0	0.0	—	—	—
	12;6-12;11	7	3	42.9	33.3	66.7	0.0
